# Genetic predisposition to digital device use and the risk of five psychiatric disorders

**DOI:** 10.3389/fpsyt.2025.1463212

**Published:** 2025-05-22

**Authors:** Qi Liu, Zhen Zhang

**Affiliations:** ^1^ Department of Child Developmental Behavior & Henan Provincial Medical Key Lab of Child Developmental Behavior and Learning, the Third Affiliated Hospital of Zhengzhou University, Zhengzhou, Henan, China; ^2^ Department of Medical Administration, the Affiliated Cancer Hospital of Zhengzhou University & Henan Cancer Hospital, Zhengzhou, China

**Keywords:** digital device use, psychiatric disorders, Mendelian randomization study, causality, modifiable risk factor

## Abstract

**Background:**

Psychiatric disorders were observationally related to digital device use, but causality and direction remained unclear. We aimed to uncover the causal links between digital device use and five psychiatric disorders risk utilizing the two-sample Mendelian Randomization method.

**Methods:**

We obtained genetic variants related to digital device use from the UK Biobank’s genome-wide association study and psychiatric disorders data from the Psychiatric Genomics Consortium. The primary analysis employed the inverse-variance weighted method, complemented by sensitivity analyses to determine heterogeneity and pleiotropy.

**Results:**

There were causal relationships between genetically increased mobile phone use [odds ratio (OR) = 1.75, 95% confidence interval (CI): 1.31-2.33], more television watching (OR = 3.39, 95% CI: 2.64-4.35) and a higher risk of attention-deficit/hyperactivity disorder (ADHD). Genetically determined duration of computer use was also causally related to the risk of autism spectrum disorder (ASD) (OR = 2.66, 95%CI: 1.82-3.88). Conversely, ADHD was significantly positively associated with playing computer games (*β* = 0.021, 95%CI: 0.010-0.032) and watching television (*β* = 0.030, 95%CI: 0.010-0.049). Also, a significant inverse associations of major depression disorder (MDD) with playing computer games was observed (*β* = 0.008, 95%CI: 0.003-0.013).

**Conclusions:**

Our findings indicate potential causal links between genetic disposition to use digital devices and psychiatric disorders, such as ADHD, ASD, and MDD, highlighting the importance of digital device use in both prevention and management of these disorders.

## Graphical Abstract

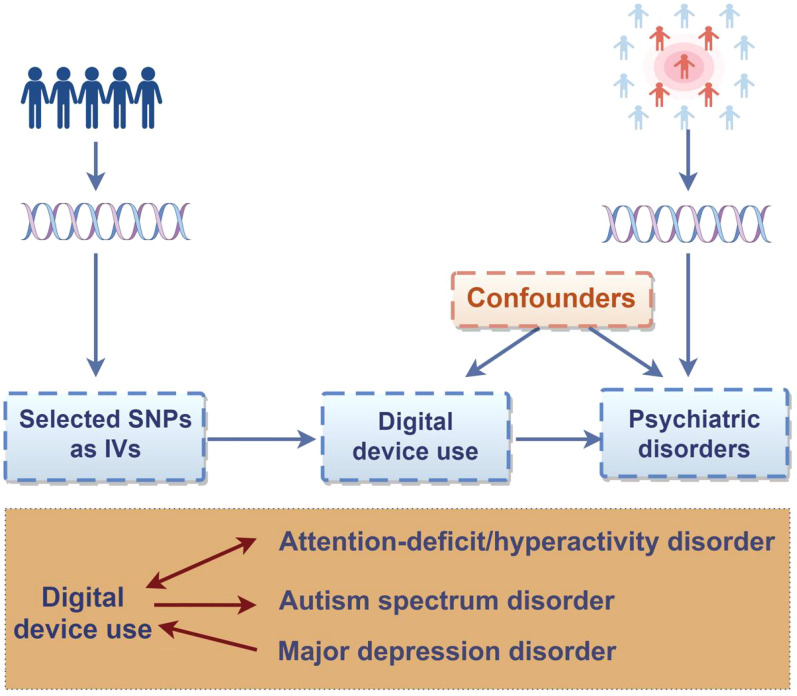

## Highlights

- Digital device use may be bidirectional causal related to the risk of ADHD.
- We found the causal effect of MDD on digital device use.
- The causal relationship was observed between digital device use and ASD risk.

## Introduction

Psychiatric disorders, widely recognized as a public health concern, remain among the leading global contributors to years lived with disability (1). It is estimated that the global number of cases due to psychiatric disorders increased from 654.8 million in 1990 to 970.1 million in 2019, and correspondingly, the global disability adjusted life-years have increased from 3.1% to 4.9% (2, 3). Furthermore, the number is predicted to rise even more (4). Facing the high global burden of psychiatric disorders, discovering modifiable causal factors for psychiatric disorders is an urgent requirement.

The proliferation of portable electronic devices has heightened concerns about their psychiatric implications (5–11). Accumulating evidence suggests that digital device use may increase the risk of psychiatric disorders, including Autism Spectrum Disorder (ASD), Attention-Deficit/Hyperactivity Disorder (ADHD), anxiety, depression, and Post-Traumatic Stress Disorder (PTSD). Specifically: ASD is characterized by impaired social communication and restricted/repetitive behaviors, with studies suggesting that early-life digital media experiences may be associated with ASD-like characteristics (7, 12). Screen exposure, such as electrical stimulation through the screen and visual light stimulation, may affect the neurodevelopment and *de novo* sequence alterations associated with ASD (9). ADHD involves inattention, hyperactivity, and impulsivity, and excessive screen time exacerbates attention deficits, as high-stimulus digital environments amplify inattention/hyperactivity, while ADHD-related impulsivity may drive compulsive device use (6, 13, 14). In addition, anxiety and depression—marked by persistent worry and pervasive low mood, respectively—are increasingly correlated with problematic social media use and nighttime screen exposure (15). The displacement hypothesis suggests that time spent on screen-based activities may replace time participating in more productive and/or active activities, especially activities involving physical movement and interpersonal communication (16). PTSD is a trauma-induced condition with intrusive memories and hyperarousal and media content related to disasters is an important correlation for PTSD (17, 18). However, these observed associations are confounded by reverse causality and environmental factors, so causal reasoning methods are needed.

Mendelian randomization (MR) is a widely recognized and robust tool for inferring the causal impact of exposures on diseases, utilizing genetic information as instrumental variables (IVs).It can effectively overcome the confounding bias of traditional epidemiological studies and thus providing more robust evidence for causal estimation. In our research, the goal was to execute a bidirectional two-sample MR study, utilizing publicly aggregated summary statistics derived from the genome-wide association study (GWAS) on digital device use behaviors, to dissect the causation between digital device use and psychiatric disorders, including ADHD, primary anxiety disorder (AD), ASD, major depression disorder (MDD), and post-traumatic stress disorder (PTSD), which would may have significant implications for public health worldwide.

Given that previous observational studies have reported associations between digital device use and mental illnesses, and theoretical and empirical evidence supports the notion that mental illnesses can influence individuals’ behavioral and lifestyle choices, we hypothesize two key relationships in this study. First, there is a causal link between genetically determined digital device use and the risk of five mental illnesses. Second, a bidirectional relationship exists between certain digital device use patterns and mental illnesses, such that digital device use may affect the development of mental illnesses, while pre - existing mental illnesses can also impact an individual’s digital device usage behavior.

## Methods

### Study aim and design

This study employed open-source two-sample MR analyses to identify the bidirectional causal effects of digital device use on the risk of five psychiatric disorders. MR validity is based on the following three principal assumptions: a) IVs should exhibit a solid relationship with digital device use; b) the exposure-outcome association of IVs must be independent of confounding factors; c) IVs must impact the outcome risk only through the exposure. With the data retrieved from public databases, the original studies had already acquired the necessary patient consent and were granted ethical approval.

### Exposure GWAS

In the analysis, data on the use of digital devices were extracted from the latest UK Biobank summary-level GWAS, which involved 422,218 participants of European descent (13). For the data from the UK Biobank, ethical approval was granted by the North West Multi - Centre Research Ethics Committee, and all participants provided informed consent prior to their enrollment. The digital devices used mainly included four categories: time spent watching television (the ID in IEU Open GWAS: ukb-b-5192), play computer games (the ID in IEU Open GWAS: ukb-b-4779), time spent using computer (the ID in IEU Open GWAS: ukb-b-4522), length of mobile phone use (the ID in IEU Open GWAS: ukb-b-4094). Participants were asked to log the time they spent on each of the four activities with responses to questions like: “In a typical DAY, how many hours do you spend watching television? (Put 0 if you do not spend any time doing it)”, “Do you play computer games?”, “In a typical DAY, how many hours do you spend using the computer? (Do not include using a computer at work; put 0 if you do not spend any time doing it)”, and “For approximately how many years have you been using a mobile phone at least once per week to make or receive calls?”. Participants reported an average of 2.92 hours per day spent watching television, having a standard deviation (SD) of 1.62 hours; for computer use, the average was 1.35 hours with a SD of 1.54 hours. Questions regarding mobile phone use were constructed based on cumulative years of use to capture long - term exposure. Gaming habits were evaluated over the past year, while questions about TV and computer use focused on daily engagement to reflect short - term habitual behavior. **Table 1** provides an overview of the phenotypic characteristics linked to digital device use.

**Table 1:** Detailed information about the phenotype feature of digital devices use.

GWAS iD	Trait	Sample size/number of SNPs	Unique name in UK Biobank	Question Stem	Responses	Validations	Hints	Mean (SD)	Manhattan plot
ukb-b-4094	Length of mobile phone use	456,972/9,851,867	**MB1**	For approximately how many years have you been using a mobile phone at least once per week to make or receive calls?	SELECT one of 7 from 0: Never used mobile phone at least once per week 1: One year or less 2: Two to four years 3: Five to eight years 4: More than eight years -1: Do not know -3: Prefer not to answer	–	Do not include time spent text messaging. If you are unsure, please provide an estimate or select Do not know.	–	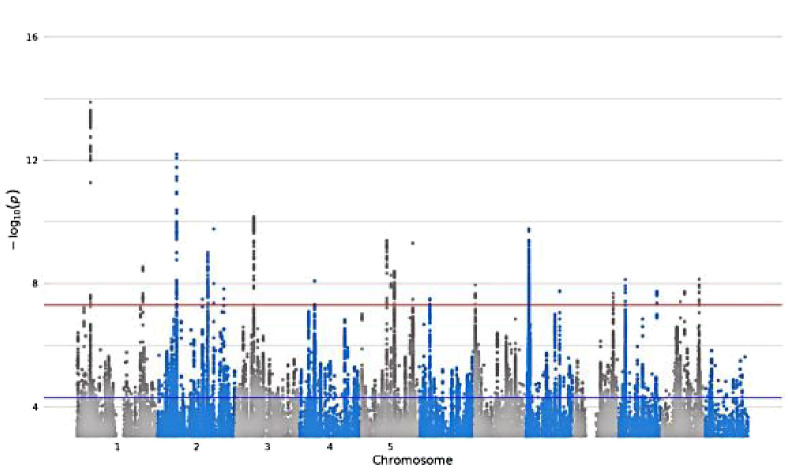
ukb-b-4522	Time spent using computer	360,895/9,851,867	**WP5A**	In a typical DAY, how many hours do you spend using the computer? (Do not include using a computer at work; put 0 if you do not spend any time doing it)	Enter INTEGER OR -10: Less than an hour a day OR -1: Do not know OR -3: Prefer not to answer	Require: ≥ 0, ≤24Expect: ≤ 6 Units: hours	If the time you spend on the computer varies a lot, give the average time for a 24 hour day in the last 4weeks. Remember not to include time spent on a computer at work.	1.35 (1.54)	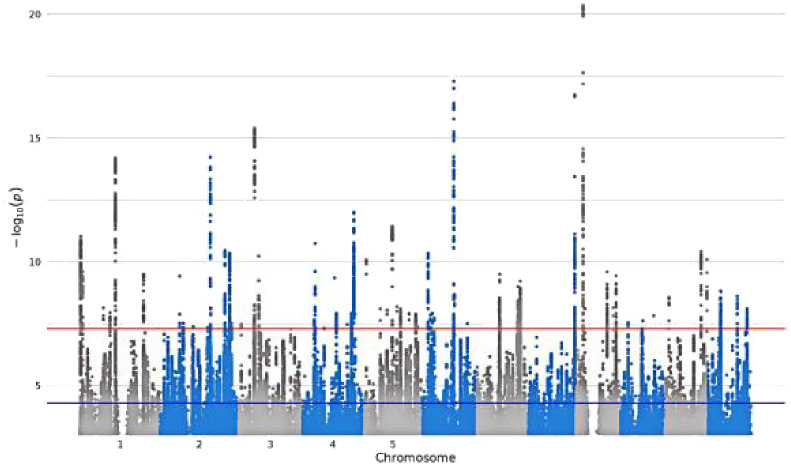
ukb-b-4779	Plays computer games	462,433/9,851,867	**F1**	Do you play computer games?	SELECT one of 4 from 0: Never/rarely 1: Sometimes 2: Often -3: Prefer not to answer	–	Answer this question thinking about the past year.	–	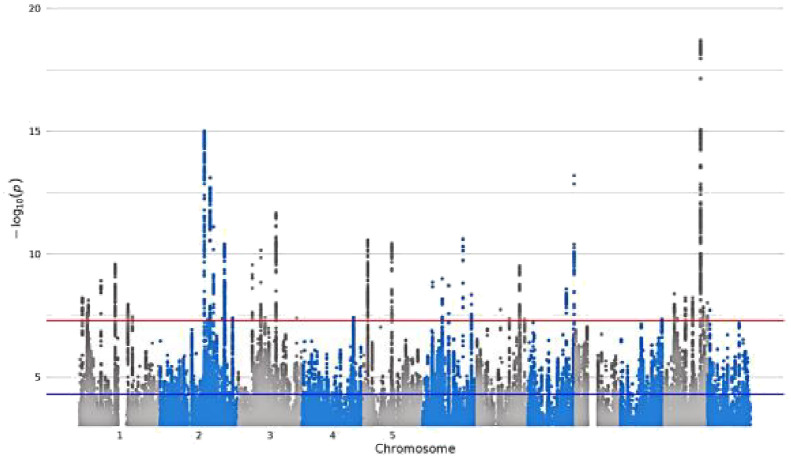
ukb-b-5192	Time spent watching television	437,887/9,851,867	**WP5**	In a typical DAY, how many hours do you spend watching TV? (Put 0 if you do not spend any time doing it)	Enter INTEGER OR -10: Less than an hour a day OR -1: Do not know OR -3: Prefer not to answer	Require: ≥ 0, ≤24 Expect: ≤ 8 Units: hours	If the time you spend watching TV varies a lot, give the average time for a 24 hour day in the last 4 weeks.	2.92 (1.62)	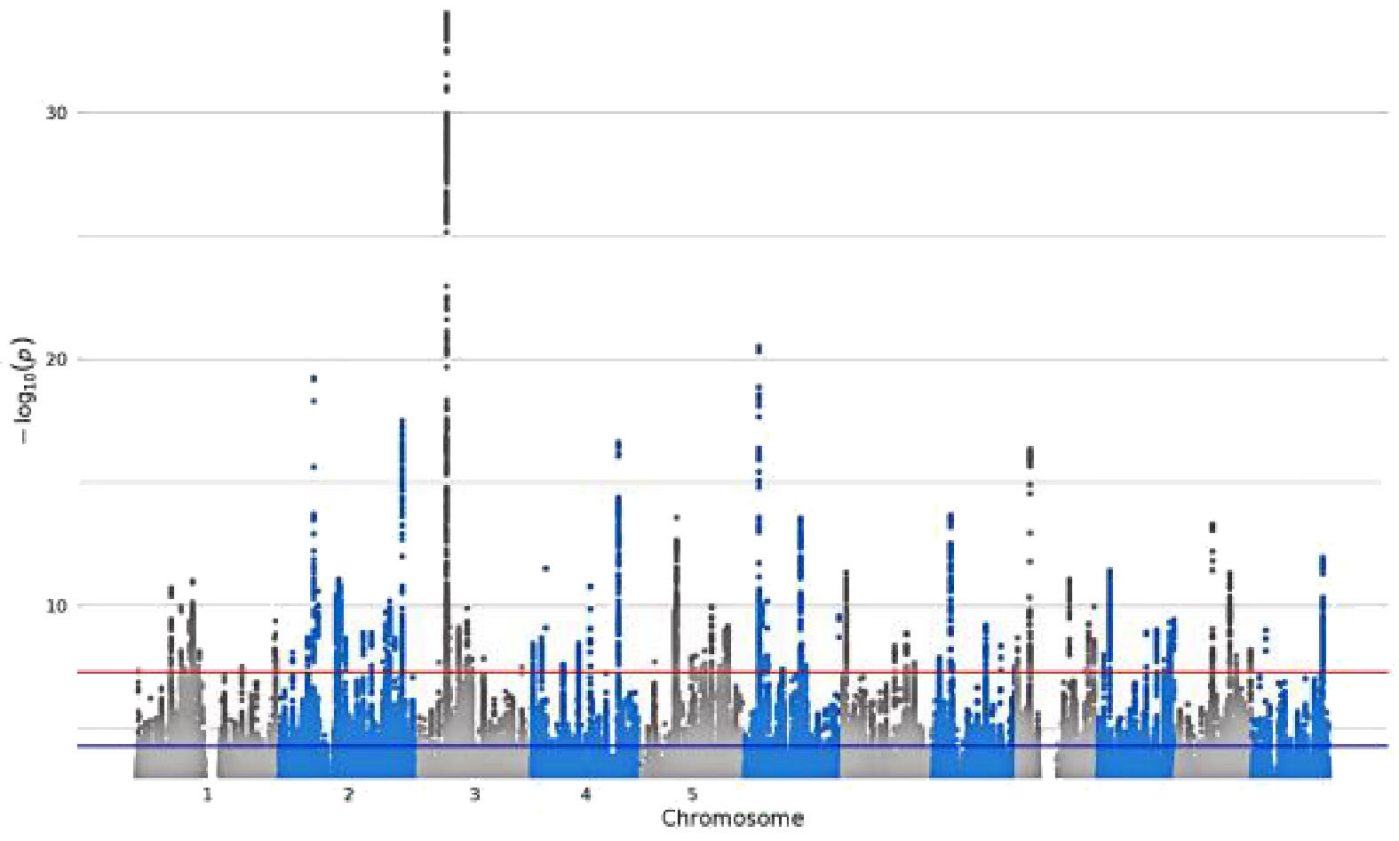

SD, standard deviation.

Bold text indicates variable coding names from the UK Biobank touch-screen questionnaire (https://biobank.ndph.ox.ac.uk/ukb/refer.cgi?id=113241; accessed May 16, 2025).

### Outcome GWAS

Permission granted, we retrieved the newest GWAS summary data pertaining to five psychiatric disorders from the Psychiatric Genomics Consortium (PGC), available online at https://pgc.unc.edu/for-researchers/download-results/. Being the foremost and most expansive consortium in psychiatric research history, the PGC aims to elucidate genetic framework of these mental disorders (19, 20). Here, we collected GWAS summary statistics from the following neuropsychiatric studies: ADHD (38,691 cases and 186,843 controls) (21), AD (5,580 cases and 11,730 controls) (22), ASD (18,381 cases and 27,969 controls) (23), MDD (45,396 cases and 97,250 controls) (24), and PTSD (23,212 cases and 151,447 controls) (25). All these studies obtained ethical approval from their respective local Institutional Review Boards (21–25). As our exposure GWAS focused mainly on European cohorts, we exclusively utilized results from GWAS that predominantly featured samples of European descent. A full list of the GWAS dataset is presented in **Table 2**.

**Table 2:** Overview of the GWAS summary statistics of the five psychiatric disorders.

Trait	PubMedID	Authors	Year	Consortium	Sample size	Number of SNPs
ADHD	36702997	Demontis et al. (21)	2023	PGC	225,534	6,774,224
AD	26754954	Otowa et al. (22)	2016	PGC	17,310	6,330,995
ASD	30804558	Grove et al. (23)	2019	PGC	46,350	9,112,386
MDD	38177345	Meng et al. (24)	2023	PGC	991,073	20,092,701
PTSD	31594949	Nievergelt et al. (25)	2019	PGC	200,000	9,766,174

ADHD, attention-deficit/hyperactivity disorder; AD, anxiety disorder; ASD, autism spectrum disorder; MDD, major depression disorder; PGC, Psychiatric Genomics consortium; PTSD, post-traumatic stress disorder; SNP, single nucleotide polymorphism.

### Instrumental SNPs selection

**Figure 1:**
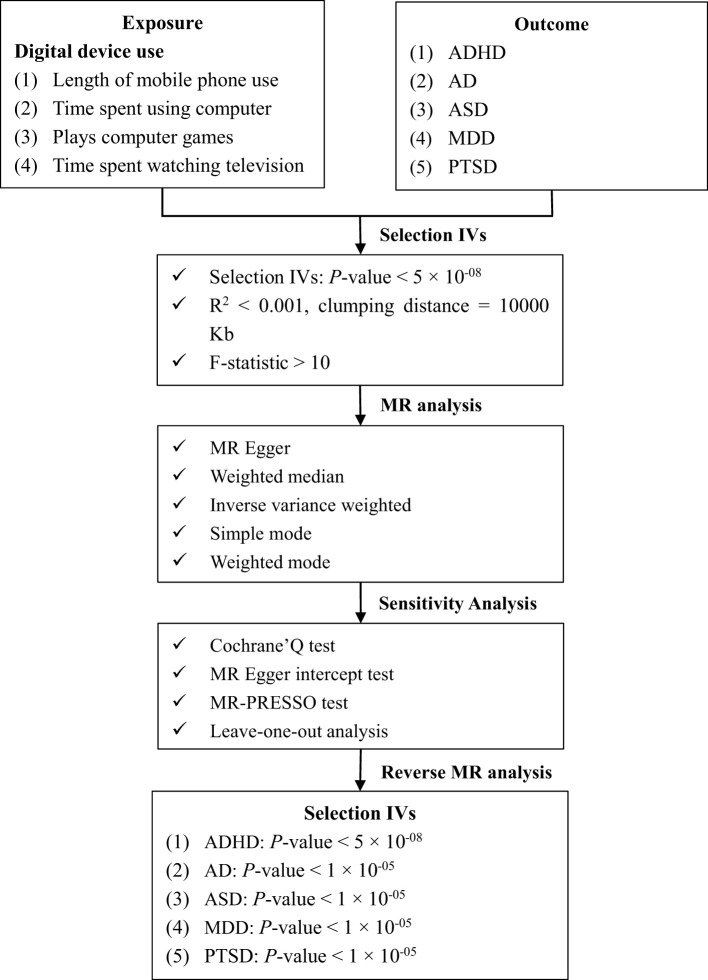
Overview of the present study design and workflow. ADHD, attention-deficit/hyperactivity disorder; AD, anxiety disorder, ASD, autism spectrum disorder; IVs, instrumental variables; MDD, major depression disorder; MR, Mendelian randomization; MR-PRESSO, MR pleiotropy residual sum and outlier test; PTSD, post traumatic stress disorder; SNP, single nucleotide polymorphism.


In addition, we executed strict filtering protocols for SNPs before engaging in the MR analysis. To begin with, ensuring significant correlations between SNPs and exposures and to evade linkage disequilibrium (LD), we defined the clump-*p* value threshold to be below 5×10^-8^, *R* ^2^ = 0.001, and a genomic distance of 10,000 kb as our qualifiers. Then, to examine the possibility of bias due to inadequate strength in IVs, we calculated the *F*-statistic (Equation 1). Here, *R* ^2^ denotes the percentage of exposure variability that can be explained by genetic factors, and is calculated as follows (Equation 2). *N* is the sample size, and *K* denotes the total number of SNPs. EAF is the frequency of the effect allele within the population, *beta* and *SE* represent the estimated genetic effect and standard error of the genetic effect. SNPs were excluded if their *F* statistic did not meet the threshold of 10. In addition, owing to the bidirectional MR design of the study, the *p*-value threshold was extended to 1×10–^5^ and included IVs strength validation when the initial qualifiers were not met following exposure inversion. **Figure 1** shows a prime description of the bidirectional MR design.

(1) $$F=\frac{R^{2}\times \left(N-1-K\right)}{\left(1-R^{2}\right)\times K}$$

(2) $$R^{2}=\frac{2\times EAF\times \left(1-EAF\right)\times beta^{2}}{2\times EAF\times \left(1-EAF\right)\times N\times SE\left(beta^{2}\right)+2\times EAF\times \left(1-EAF\right)\times beta^{2}}$$

### Two-sample MR analysis

After identifying qualified SNPs as gene IVs, we proceeded to harmonize the aggregated statistics for SNP-exposure and SNP-outcome to ensure that allele representation was consistent for each SNP when exploring the connection between digital device use and psychiatric disorders. Specifically, we checked and adjusted the effect alleles, reference alleles, and effect estimates to make sure that the genetic information was comparable across different datasets.

To test the possibility of causal relationships between digital device use and the risk of major psychiatric disorders, we employed multiple statistical methods. The inverse variance weighted (IVW) analysis served as our primary method for estimating the causal effect. This approach calculates a weighted average of the causal effect estimates from individual SNPs, with the weights being inversely proportional to the variance of the SNP - specific effect estimates. It assumes balanced pleiotropy and provides precise estimates under valid instrumental variable assumptions.

The MR-Egger regression model was applied to test for the presence of horizontal pleiotropy. It includes an intercept term that can detect directional pleiotropy, allowing us to assess whether the causal effect estimates were confounded by pleiotropic effects of the SNPs.

Furthermore, we used the weighted median approach, which gives more weight to SNPs with more precise effect estimates. This method is robust to up to 50% of invalid instruments and can provide a reliable estimate of the causal effect even when some SNPs do not fully meet the IV assumptions. Simple mode analysis and weighted mode analysis were also conducted. These methods focus on the most frequently occurring effect estimate (simple mode) or a weighted version of it (weighted mode) among the SNPs, respectively, and can offer alternative perspectives on the causal relationship, especially in the presence of outliers or heterogeneous SNP effects.

### Sensitivity analysis

The presence of heterogeneity was determined using Cochran’s Q statistic, where a *p*-value below 0.05 denote its existence. The MR-PRESSO test and MR-Egger intercept test serve as tools to identify horizontal pleiotropy. When the MR-PRESSO global test revealed substantial horizontal pleiotropy in our study, we proceeded to eliminate outliers with a *p*-value below 0.05 and subsequently reassessed the remaining SNPs using the MR analysis. And a *p*-value of more than 0.05 for the MR-Egger regression intercept suggests that horizontal pleiotropy is not detected. Additionally, to assess whether a single SNP was responsible for the causal effect in the two-sample MR analysis, we executed a leave-one-out sensitivity analysis.

To test the possibility of reverse causality between psychiatric disorders and digital device use, the investigation further extended to encompass bidirectional two-sample MR analyses. We calculated the effect size with the OR (or *beta*) and 95% confidence interval (CI). Moreover, Benjamini–Hochberg correction was implemented to adjust for multiple testing. A *p*-value below 0.05 was deemed statistically significant.

Statistical tests were two-sided and performed on the R platform, version 4.3.3 (R Foundation for Statistical Computing Vienna, Austria). The MR analyses were facilitated by the TwoSampleMR package, version 0.5.11 (https://github.com/MRCIEU/TwoSampleMR).

## Results

In the two-sample MR analysis, we first extracted independent digital device use-related SNPs that met the criteria for genome-wide significance, with a *p*-value less than 5 × 10^−8^, an *R* ^2^ less than 0.001, and located within a 10,000 kilobase interval. The SNPs that were associated with confounding factors, with palindromic or incompatible alleles, as well as pleiotropic SNPs, identified through the MR-PRESSO outlier test, were also eliminated from the analysis (**Supplementary Table 1**). After a series of data extraction and selection, the detailed information about the IVs is shown in **Supplementary Tables 2**-**6**. The *F*-statistics of all these genetic variants were above the threshold of 10 (ranged: 29.738 to 151.702), indicting no evidence of potential weak instrument bias.

**Figure 2:**
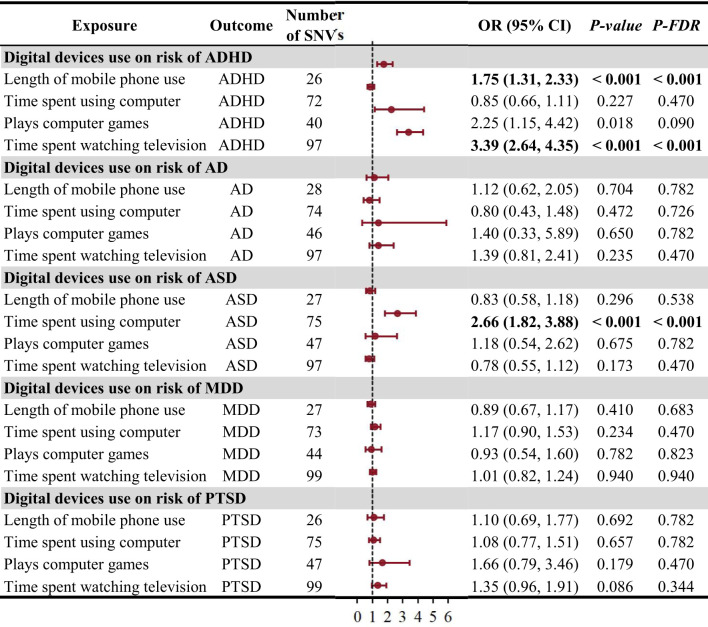
The causal effect of digital device use on the risk of psychiatric disorders using the inverse variance weighted method. ADHD, attention-deficit/hyperactivity disorder; AD, anxiety disorder; ASD, autism spectrum disorder; CI, confidence interval; MDD, major depression disorder; OR, odds ratio; PTSD, post traumatic stress disorder; SNV, single-nucleotide variant.

According to the IVW results, we found there were causal relationships between genetically increased long - term mobile phone use (in years) [odds ratio (OR) = 1.75, 95% CI: 1.31-2.33, *P_FDR_* < 0.001], time of watching television (in days) (OR = 3.39, 95% CI: 2.64-4.35, *P_FDR_* < 0.001) and the risk of ADHD. Furthermore, a longer genetically determined time spent using computer in the past year was correlated with a greater risk of ASD (OR = 2.66, 95%CI: 1.82-3.88, *P_FDR_* < 0.001). No statistically significant correlation was found between digital device use and the risk of AD, MDD, and PTSD (**Figure 2**). The MR analyses conducted to assess the potential causal relationship between digital device use and the risk of the five psychiatric disorders using MR-Egger regression method, weighted median analysis, simple mode analysis, and weighted mode analysis are outlined in **Supplementary Table 7**.

In the sensitivity analyses, the MR-PRESSO analysis detected 20 outlier SNPs, which were then removed for subsequent analysis. The Cochran’s Q test revealed that most estimates obtained from individual SNPs exhibited different degrees of heterogeneity (*p* > 0.05), but the MR-Egger regression analysis yielded no signs of horizontal pleiotropy (CI for intercept ranging from−0.021 to 0.098, all *p* > 0.05) except for the test for playing computer games and AD (**Supplementary Table 8**). Additionally, scatter plots for using mobile phone, watching television, playing computer games, and using computer had positive slopes, suggesting that they were risk factors for ADHD and ASD (**Supplementary Figures 1**-**5**). The forest plots, funnel plots, and leave-one-out sensitivity analysis plots were depicted in the **Supplementary Figures 6**-**20**, implying a stability of our results.

**Figure 3:**
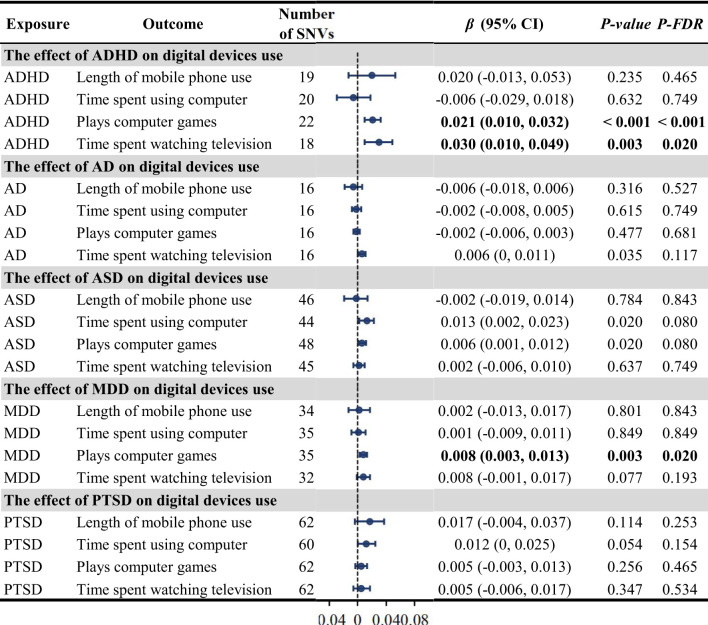
The causal effect of the five psychiatric disorders on digital device use using the inverse variance weighted method. ADHD, attention-deficit/hyperactivity disorder; AD, anxiety disorder; ASD, autism spectrum disorder; CI, confidence interval; MDD, major depression disorder; OR, odds ratio; PTSD, post traumatic stress disorder; SNV, single-nucleotide variant.

To evaluate any reverse causation effects, we used the five psychiatric disorders as exposure and digital device use as outcome. We observed that genetically predicted ADHD may be significantly positively related to play computer games in the past year (IVW, *β* = 0.021, 95%CI: 0.010-0.032, *P_FDR_* < 0.001) and the duration spent watching television (in days) (IVW, *β* = 0.030, 95%CI: 0.010-0.049, *P_FDR_* = 0.020), which indicated a bi-directional causal effect between ADHD and digital device use. Moreover, MDD was related to play computer games in the past year (IVW, *β* = 0.008, 95%CI: 0.003-0.013, *P_FDR_* = 0.020) (**Figure 3**). The MR analysis using other methods was displayed in **Supplementary Table 9**. Upon detecting potential heterogeneity and horizontal pleiotropy in the IVs (**Supplementary Table 10**), we proceeded to eliminate outlier SNPs identified by MR-PRESSO from the subsequent analysis and utilize the IVW method for the primary analysis. No significant abnormal SNVs were found in the subsequent leave-one-out analysis (**Supplementary Figures 21**-**25**).

## Discussion

The study employed a bidirectional two-sample MR analysis to explore the causal links between four digital device use traits and five psychiatric disorders. We uncovered that digital device use exhibited significant positive bidirectional causal effects with ADHD. Furthermore, higher genetically predicted digital device use was consistently associated with increased ASD risk. There were also weak reverse causal effects between genetic determinants of MDD and digital device use. However, we did not find evidence supporting causal associations between digital device use and the risk of AD and PTSD.

The significant association between digital device use and the risk of ASD and ADHD has been widely discussed in previous research. For example, several meta-analyses have revealed minor yet statistically notable pooled zero-order correlations connecting time spent on screen media with behaviors related to ADHD (26–28). Likewise, it has been suggested that high digital media time among children and youth was associated with the risk of ASD in a series of longitudinal (7) and cross-sectional studies (29–31). But, on the flip side, scholars and health professionals are also focusing on the opposite direction of this association—that is, whether some of these children with ADHD (32) or ASD like symptoms might be more likely to use digital media later on. However, the observational nature of the existing researches limits their ability to ascertain the causal direction of the association (30). In this study, we supported a bi-directional causal relationship between digital device use and the risk of ADHD via mendelian randomization. Moreover, the use of electronic devices increases the risk of ASD. These discoveries could offer novel insights for etiology research and clinical interventions of ASD and ADHD.

The underlying mechanism by which digital device use affects ASD and ADHD may be related to the structural development of brain (33–36). Recent evidence revealed that increased digital media consumption was linked to reduced cortical thickness and sulcal depth in brain areas, which was related to visual processing, executive functions, social cognition, and attention (34–36). Similar findings were also observed in early childhood, highlighting the differences in white matter microstructure when using screen at this age (34). Furthermore, as reported by a functional magnetic resonance imaging study, the network connectivity involving language, visual, cerebellar, and default-mode systems was significantly reduced during animated stories (33). Thus, the use of digital media might exert a range of impacts, both direct and indirect, on children’s skill development and developing brains (37), thereby affecting the pathogenesis of ASD and ADHD. With regard to reverse causation, it is suggested that children with ADHD behaviors would be more attracted to screen activities than their peers to avoid real-life communication challenges in these activities.

To our knowledge, a number of literature review and meta-analysis have summarized what is known about linkages between digital technology usage and mental health, especially for depression and anxiety disorder, in children and adolescents (38–41). However, most of the evidence to date is of a correlational or associative nature, and has resulted in small positive (42–46), negative (47, 48) and null outcomes (49). Hence, in this study, we initiated a two-sample MR analysis to determine the true causality and directionality underlying the shared associations. Our study demonstrated that MDD may affect the use of social media. Nevertheless, we did not support the hypotheses that social media use have an effect on the risk of MDD and AD. This suggests that it may be useful to leverage these factors as early indicators of depression disorder, rather than as direct modifiers of the risk of depression and anxiety disorder (50). Future investigations should dig deeper to clarify whether this effect is affected by factors such as video duration, content, frequency of use, media type and the number of devices.

Moreover, limited research has explored the impact of media exposure on symptoms of PTSD. As reported, the development of PTSD symptoms is linked to the intake of information from modern media sources (51). Besides, it was discovered that a greater frequency of television watching, more active media sharing, and a larger number of online friendships significantly relate to higher PTSD symptom severity (52). In contrast, our limited data did not support a causal association between the four digital device use characteristics and PTSD. The focus of future studies needs to be on investigating the effect of media content on PTSD symptoms.

This is the first comprehensive and extensive study to appraise the causal effects of digital device use on various psychiatric disorders (ADHD, ASD, AD, MDD, and PTSD) by summarizing the evidence from MR approach. However, there are several limitations. First, the analysis did not take into account the purpose, content, and context of digital device use, rather than just the duration of screen exposure. Undoubtedly, To thoroughly understand the link between media and psychiatric disorders, more in-depth analysis of screen media content and more rigorous assessments of screen media device use are required (51, 53). Even if some associations do not pass the corrected multiple-testing thresholds, they may still hold biological plausibility. These associations are worthy of further investigation in future studies, as they might represent real but subtle relationships between digital device use and psychiatric disorders. Second, the relationship between digital device use and the five psychiatric disorders may be nonlinear. Nevertheless, due to the design of the MR analysis, we have not test for non-linear associations between exposures and diseases. Third, our research includes GWAS data that is primarily focused on individuals with European ancestry; further replication in diverse ethnic populations is necessary for confirmation. Fourth, regarding the assessment of electronic devices, measurement heterogeneity may limit direct comparisons between different categories of digital device use. Future studies should strive to standardize the measurement of digital device use to enable more robust and comparable analyses. Additionally, this study is subject to recall and response biases. The self-reported usage of electronic devices relies on participants’ memory and honesty, and recall or response biases may occur, especially when recollecting events over an extended period. Moreover, certain genetic factors associated with digital device use may also be related to other lifestyle or environmental factors, which may independently influence the risk of psychiatric disorders. These unaccounted confounding factors may introduce biases into our causal estimates. Such biases have the potential to distort the relationship between digital device use and psychiatric disorders, leading to overestimation or underestimation of the associations. Finally, although we used the latest available GWAS with the largest sample size in the data analysis; the update rate in the field of genetic research is very high, and there will be more GWAS available in the future.

## Conclusions

In summary, this two-sample MR study has revealed a bidirectional relationship between digital device use and the risk of ADHD. Additionally, the use of electronic devices increases the risk of ASD, and MDD was identified as unidirectional high-risk factors for digital device use. Our findings provide valuable policies and practices guidance for the pivotal role of digital device use as a key modifiable risk factor in the prevention and management of psychiatric disorders.

## Data Availability

Publicly available datasets were analyzed in this study. This data can be found here: the UK BIOBANK (http://www.nealelab.is/uk-biobank) and the Psychiatric Genomics Consortium (https://pgc.unc.edu/).
