# Supplementary material for: Genetic predisposition to digital device use and the risk of five psychiatric disorders
Source: Front Psychiatry. 2025 May 22;16:1463212. doi: 10.3389/fpsyt.2025.1463212 (PMC12137273; doi:10.3389/fpsyt.2025.1463212)
Supplement: Supplementary file 1 [file Supplementaryfile1.docx]

**Supplementary material**

**Table S1.** Outlier SNPs identified by the MR-PRESSO test

**Table S2.** Genetic instruments for attention-deficit/hyperactivity disorder

**Table S3.** Genetic instruments for anxiety disorder

**Table S4.** Genetic instruments for autism spectrum disorder

**Table S5.** Genetic instruments for major depression disorder

**Table S6.** Genetic instruments for post traumatic stress disorder

**Table S7.** The causal effect of digital device use on the risk of the five psychiatric disorders (SNPs reaching *P* < 5×10-8)

**Table S8.** Test on pleiotropy and heterogeneity for the selected SNVs

**Fig. S1.** Scatter plots of single SNVs included in the mendelian randomization study for the effects of digital device use on the risk of attention-deficit/hyperactivity disorder

**Fig. S2.** Scatter plots of single SNVs included in the mendelian randomization study for the effects of digital device use on the risk of anxiety disorder

**Fig. S3.** Scatter plots of single SNVs included in the mendelian randomization study for the effects of digital device use on the risk of autism spectrum disorder

**Fig. S4.** Scatter plots of single SNVs included in the mendelian randomization study for the effects of digital device use on the risk of major depression disorder

**Fig. S5.** Scatter plots of single SNVs included in the mendelian randomization study for the effects of digital device use on the risk of post traumatic stress disorder

**Fig. S6.** Forest plots for the effects of digital device use on the risk of attention-deficit/hyperactivity disorder

**Fig. S7.** Forest plots for the effects of digital device use on the risk of anxiety disorder

**Fig. S8.** Forest plots for the effects of digital device use on the risk of autism spectrum disorder

**Fig. S9.** Forest plots for the effects of digital device use on the risk of major depression disorder

**Fig. S10.** Forest plots for the effects of digital device use on the risk of post traumatic stress disorder

**Fig. S11.** Leave-one-out plot for the effects of digital device use on the risk of attention-deficit/hyperactivity disorder

**Fig. S12.** Leave-one-out plot for the effects of digital device use on the risk of anxiety disorder

**Fig. S13.** Leave-one-out plot for the effects of digital device use on the risk of autism spectrum disorder

**Fig. S14.** Leave-one-out plot for the effects of digital device use on the risk of major depression disorder

**Fig. S15.** Leave-one-out plot for the effects of digital device use on the risk of post traumatic stress disorder

**Fig. S16.** Funnel plots for the effects of digital device use on the risk of attention-deficit/hyperactivity disorder

**Fig. S17.** Funnel plots for the effects of digital device use on the risk of anxiety disorder

**Fig. S18.** Funnel plots for the effects of digital device use on the risk of autism spectrum disorder

**Fig. S19.** Funnel plots for the effects of digital device use on the risk of major depression disorder

**Fig. S20.** Funnel plots for the effects of digital device use on the risk of post traumatic stress disorder

**Table S9.** The causal effect of the five psychiatric disorders on digital device use in the mendelian randomization study

**Table S10.** Test on pleiotropy and heterogeneity for the selected SNVs related to psychiatric disorders used as instruments in analysis of effect on digital device use

**Fig. S21.** Leave-one-out plot for the effects of attention-deficit/hyperactivity disorder on digital device use

**Fig. S22.** Leave-one-out plot for the effects of anxiety disorder on digital device use

**Fig. S23.** Leave-one-out plot for the effects of autism spectrum disorder on digital device use

**Fig. S24.** Leave-one-out plot for the effects of major depression disorder on digital device use

**Fig. S25.** Leave-one-out plot for the effects of post traumatic stress disorder on digital device use

**Table S1.** Outlier SNPs identified by the MR-PRESSO test

| **Exposure** | **Outcome** | **Excluded SNVs** | **RSSobs** | ***P-value*** |
| --- | --- | --- | --- | --- |
| Length of mobile phone use | ADHD | rs17156711 | 1.10E-03 | 0.029 |
| Time spent using computer | ADHD | rs11749912 | 1.62E-03 | 0.016 |
| Plays computer games | ADHD | rs11743441 | 2.09E-03 | <0.009 |
| Plays computer games | ADHD | rs411844 | 1.71E-03 | 0.028 |
| Plays computer games | ADHD | rs9842435 | 4.59E-03 | <0.009 |
| Plays computer games | ADHD | rs4938017 | 1.08E-03 | 0.018 |
| Time spent watching television | ADHD | rs34094119 | 1.33E-03 | 0.022 |
| Time spent watching television | ADHD | rs61864793 | 1.40E-03 | 0.044 |
| Length of mobile phone use | ASD | rs10107145 | 3.67E-03 | <0.006 |
| Time spent watching television | ASD | rs34094119 | 2.57E-03 | <0.022 |
| Time spent watching television | ASD | rs7921305 | 3.79E-03 | <0.022 |
| Time spent watching television | ASD | rs898751 | 2.52E-03 | <0.022 |
| Length of mobile phone use | MDD | rs17156711 | 1.88E-03 | <0.006 |
| Time spent using computer | MDD | rs2734833 | 1.13E-03 | 0.016 |
| Time spent using computer | MDD | rs2756121 | 1.62E-03 | <0.016 |
| Plays computer games | MDD | rs4938017 | 9.53E-04 | 0.019 |
| Length of mobile phone use | PTSD | rs2836920 | 3.33E-03 | <0.006 |
| Length of mobile phone use | PTSD | rs6063374 | 3.24E-03 | 0.019 |
| Time spent watching television | PTSD | rs11911112 | 2.92E-03 | 0.022 |
| Time spent watching television | PTSD | rs13107325 | 9.35E-03 | 0.045 |

**Note:** Abbreviations: ADHD, attention-deficit/hyperactivity disorder; ASD, autism spectrum disorder; MDD, major depression disorder; PTSD, post traumatic stress disorder; MR-PRESSO, the Mendelian randomization pleiotropy residual sum and outlier test; SNV, single-nucleotide variant

**Table S2.** Genetic instruments for attention-deficit/hyperactivity disorder

| **Exposure** | **SNP** | **Chromosome** | **Position** | **Effect allele** | **Other allele** | **Effect allele frequency** | **Exposure** | | |  | **Outcome** | | | R2 | ***F-Statistic*** |
| --- | --- | --- | --- | --- | --- | --- | --- | --- | --- | --- | --- | --- | --- | --- | --- |
|  |  |  |  |  |  |  | ***Beta*** | ***SE*** | ***P-value*** |  | ***Beta*** | ***SE*** | ***P-value*** |  |  |
| Length of mobile phone use | rs10107145 | 8 | 10758213 | G | A | 0.545 | -0.018 | 0.003 | 1.70E-10 |  | -0.023 | 0.01 | 0.016 | 8.92E-05 | 40.785 |
| Length of mobile phone use | rs10807124 | 6 | 33404064 | A | G | 0.274 | -0.018 | 0.003 | 3.10E-08 |  | -0.028 | 0.01 | 0.005 | 6.70E-05 | 30.623 |
| Length of mobile phone use | rs10828247 | 10 | 21822856 | G | A | 0.344 | 0.017 | 0.003 | 7.40E-09 |  | 0.019 | 0.01 | 0.049 | 7.31E-05 | 33.426 |
| Length of mobile phone use | rs11229008 | 11 | 57118840 | A | G | 0.063 | -0.033 | 0.006 | 3.90E-08 |  | 0.02 | 0.021 | 0.322 | 6.61E-05 | 30.222 |
| Length of mobile phone use | rs11236714 | 11 | 70424559 | T | C | 0.196 | -0.02 | 0.004 | 1.80E-08 |  | -0.008 | 0.012 | 0.536 | 6.93E-05 | 31.661 |
| Length of mobile phone use | rs11655813 | 17 | 2119101 | T | C | 0.355 | 0.018 | 0.003 | 1.20E-09 |  | 0.006 | 0.01 | 0.55 | 8.10E-05 | 37.04 |
| Length of mobile phone use | rs11682846 | 2 | 157008703 | T | C | 0.485 | -0.017 | 0.003 | 9.90E-10 |  | -0.009 | 0.009 | 0.329 | 8.17E-05 | 37.352 |
| Length of mobile phone use | rs12145998 | 1 | 204969419 | T | C | 0.266 | -0.019 | 0.003 | 2.90E-09 |  | -0.02 | 0.011 | 0.067 | 7.71E-05 | 35.224 |
| Length of mobile phone use | rs12437348 | 14 | 36606550 | A | G | 0.71 | 0.017 | 0.003 | 4.20E-08 |  | -0.021 | 0.01 | 0.04 | 6.57E-05 | 30.033 |
| Length of mobile phone use | rs13266457 | 8 | 106086786 | T | C | 0.33 | -0.017 | 0.003 | 1.70E-08 |  | 0.015 | 0.01 | 0.138 | 6.97E-05 | 31.856 |
| Length of mobile phone use | rs1512142 | 4 | 47004305 | A | G | 0.443 | -0.017 | 0.003 | 8.30E-09 |  | -0.022 | 0.01 | 0.022 | 7.27E-05 | 33.205 |
| Length of mobile phone use | rs17374152 | 5 | 93236948 | G | A | 0.239 | -0.019 | 0.003 | 5.30E-09 |  | -0.005 | 0.011 | 0.632 | 7.46E-05 | 34.088 |
| Length of mobile phone use | rs1892417 | 1 | 41779673 | C | T | 0.229 | 0.026 | 0.003 | 1.30E-14 |  | -0.01 | 0.011 | 0.387 | 1.30E-04 | 59.328 |
| Length of mobile phone use | rs2161220 | 5 | 80241904 | A | G | 0.248 | 0.021 | 0.003 | 4.00E-10 |  | 0.03 | 0.011 | 0.005 | 8.56E-05 | 39.118 |
| Length of mobile phone use | rs2836920 | 21 | 40512918 | G | T | 0.396 | 0.019 | 0.003 | 2.30E-10 |  | 0.014 | 0.01 | 0.144 | 8.80E-05 | 40.218 |
| Length of mobile phone use | rs28713780 | 7 | 3321415 | C | T | 0.641 | -0.017 | 0.003 | 1.10E-08 |  | -0.008 | 0.009 | 0.392 | 7.15E-05 | 32.685 |
| Length of mobile phone use | rs344868 | 2 | 140095800 | T | C | 0.247 | 0.018 | 0.003 | 3.20E-08 |  | 0.015 | 0.011 | 0.16 | 6.69E-05 | 30.553 |
| Length of mobile phone use | rs359265 | 2 | 60456410 | A | G | 0.607 | 0.021 | 0.003 | 6.40E-13 |  | 0.024 | 0.01 | 0.012 | 1.13E-04 | 51.715 |
| Length of mobile phone use | rs6063374 | 20 | 47832997 | G | A | 0.781 | 0.029 | 0.003 | 1.00E-17 |  | 0.004 | 0.011 | 0.712 | 1.61E-04 | 73.426 |
| Length of mobile phone use | rs6131703 | 20 | 15754684 | G | A | 0.386 | -0.018 | 0.003 | 1.90E-09 |  | -0.006 | 0.01 | 0.551 | 7.90E-05 | 36.121 |
| Length of mobile phone use | rs6780051 | 3 | 56193150 | T | G | 0.058 | 0.04 | 0.006 | 6.90E-11 |  | 0.038 | 0.02 | 0.052 | 9.31E-05 | 42.548 |
| Length of mobile phone use | rs78166132 | 5 | 161239986 | C | T | 0.094 | -0.031 | 0.005 | 4.90E-10 |  | 0.011 | 0.016 | 0.496 | 8.47E-05 | 38.728 |
| Length of mobile phone use | rs7859831 | 9 | 126205754 | T | C | 0.139 | -0.023 | 0.004 | 2.10E-08 |  | -0.018 | 0.014 | 0.2 | 6.87E-05 | 31.393 |
| Length of mobile phone use | rs8014346 | 14 | 46832002 | A | G | 0.535 | 0.019 | 0.003 | 3.70E-11 |  | 0.034 | 0.009 | 0 | 9.57E-05 | 43.758 |
| Length of mobile phone use | rs849527 | 2 | 206592525 | G | A | 0.547 | -0.016 | 0.003 | 1.50E-08 |  | -0.016 | 0.009 | 0.082 | 7.00E-05 | 31.995 |
| Length of mobile phone use | rs853946 | 10 | 120166472 | T | C | 0.468 | 0.016 | 0.003 | 1.80E-08 |  | 0.006 | 0.009 | 0.538 | 6.94E-05 | 31.707 |
| Plays computer games | rs10170573 | 2 | 225552916 | G | A | 0.654 | -0.006 | 0.001 | 4.00E-08 |  | -0.018 | 0.01 | 0.07 | 6.52E-05 | 30.147 |
| Plays computer games | rs10799961 | 1 | 163744838 | G | A | 0.538 | 0.006 | 0.001 | 3.50E-08 |  | 0.022 | 0.009 | 0.017 | 6.57E-05 | 30.388 |
| Plays computer games | rs10803798 | 2 | 166399307 | G | T | 0.284 | 0.008 | 0.001 | 7.80E-12 |  | -0.006 | 0.011 | 0.591 | 1.01E-04 | 46.811 |
| Plays computer games | rs11223780 | 11 | 134261150 | G | A | 0.148 | 0.008 | 0.001 | 9.50E-09 |  | 0.04 | 0.014 | 0.003 | 7.12E-05 | 32.946 |
| Plays computer games | rs113002196 | 1 | 27200156 | C | T | 0.16 | 0.008 | 0.001 | 7.50E-09 |  | 0.031 | 0.012 | 0.014 | 7.22E-05 | 33.411 |
| Plays computer games | rs11548535 | 7 | 148936878 | T | G | 0.146 | -0.008 | 0.001 | 4.50E-08 |  | -0.005 | 0.013 | 0.721 | 6.47E-05 | 29.912 |
| Plays computer games | rs11740196 | 5 | 12855333 | G | A | 0.484 | -0.007 | 0.001 | 2.60E-11 |  | -0.012 | 0.01 | 0.194 | 9.61E-05 | 44.46 |
| Plays computer games | rs12129719 | 1 | 66324512 | A | G | 0.552 | 0.006 | 0.001 | 1.20E-09 |  | 0.019 | 0.01 | 0.043 | 8.00E-05 | 37 |
| Plays computer games | rs12138787 | 1 | 150246070 | C | T | 0.124 | 0.009 | 0.002 | 1.10E-08 |  | 0.006 | 0.014 | 0.68 | 7.05E-05 | 32.605 |
| Plays computer games | rs1232205 | 11 | 31661314 | C | T | 0.302 | -0.007 | 0.001 | 4.10E-09 |  | -0.017 | 0.01 | 0.082 | 7.48E-05 | 34.595 |
| Plays computer games | rs12361956 | 11 | 40406823 | C | T | 0.39 | -0.006 | 0.001 | 4.10E-08 |  | -0.006 | 0.01 | 0.51 | 6.51E-05 | 30.107 |
| Plays computer games | rs12707117 | 7 | 133429058 | A | G | 0.555 | 0.007 | 0.001 | 3.00E-10 |  | -0.022 | 0.009 | 0.018 | 8.58E-05 | 39.698 |
| Plays computer games | rs13262595 | 8 | 143316970 | G | A | 0.561 | 0.008 | 0.001 | 6.30E-14 |  | 0.006 | 0.009 | 0.547 | 1.22E-04 | 56.287 |
| Plays computer games | rs144377835 | 3 | 70581216 | T | C | 0.039 | 0.018 | 0.003 | 6.90E-11 |  | 0.013 | 0.027 | 0.617 | 9.20E-05 | 42.556 |
| Plays computer games | rs16831510 | 2 | 200305460 | T | G | 0.128 | 0.01 | 0.002 | 3.90E-11 |  | 0.003 | 0.014 | 0.857 | 9.44E-05 | 43.674 |
| Plays computer games | rs2014653 | 20 | 50788476 | G | A | 0.298 | -0.008 | 0.001 | 3.70E-11 |  | -0.013 | 0.011 | 0.227 | 9.47E-05 | 43.774 |
| Plays computer games | rs302719 | 1 | 8490320 | G | T | 0.34 | -0.006 | 0.001 | 6.00E-09 |  | -0.008 | 0.01 | 0.411 | 7.32E-05 | 33.848 |
| Plays computer games | rs34217929 | 2 | 137485074 | A | G | 0.256 | 0.01 | 0.001 | 9.70E-16 |  | 0.006 | 0.01 | 0.53 | 1.39E-04 | 64.484 |
| Plays computer games | rs34402857 | 2 | 156109104 | A | G | 0.519 | 0.008 | 0.001 | 7.90E-14 |  | -0.004 | 0.011 | 0.684 | 1.21E-04 | 55.833 |
| Plays computer games | rs35104374 | 12 | 6739497 | C | T | 0.725 | -0.007 | 0.001 | 1.90E-08 |  | -0.003 | 0.011 | 0.811 | 6.82E-05 | 31.559 |
| Plays computer games | rs4113587 | 6 | 128311640 | G | T | 0.443 | 0.007 | 0.001 | 2.40E-11 |  | -0.011 | 0.009 | 0.266 | 9.65E-05 | 44.651 |
| Plays computer games | rs535591 | 3 | 83154118 | A | G | 0.788 | -0.007 | 0.001 | 3.80E-08 |  | -0.023 | 0.013 | 0.082 | 6.54E-05 | 30.236 |
| Plays computer games | rs580241 | 11 | 66066349 | A | G | 0.763 | -0.007 | 0.001 | 5.90E-09 |  | -0.001 | 0.011 | 0.95 | 7.32E-05 | 33.866 |
| Plays computer games | rs62241000 | 22 | 42378507 | T | C | 0.321 | 0.006 | 0.001 | 2.90E-08 |  | -0.011 | 0.01 | 0.296 | 6.66E-05 | 30.788 |
| Plays computer games | rs623863 | 11 | 88608915 | G | A | 0.651 | 0.006 | 0.001 | 5.90E-09 |  | 0.014 | 0.01 | 0.143 | 7.32E-05 | 33.854 |
| Plays computer games | rs6457740 | 6 | 33697125 | G | A | 0.703 | -0.007 | 0.001 | 1.40E-09 |  | -0.033 | 0.011 | 0.002 | 7.93E-05 | 36.664 |
| Plays computer games | rs6536378 | 4 | 159857227 | G | T | 0.653 | 0.006 | 0.001 | 3.70E-08 |  | -0.022 | 0.01 | 0.024 | 6.55E-05 | 30.279 |
| Plays computer games | rs678007 | 1 | 23600579 | T | C | 0.178 | -0.008 | 0.001 | 3.40E-08 |  | -0.018 | 0.012 | 0.131 | 6.59E-05 | 30.473 |
| Plays computer games | rs717915 | 2 | 200714766 | T | C | 0.635 | 0.006 | 0.001 | 3.50E-08 |  | -0.008 | 0.01 | 0.391 | 6.58E-05 | 30.417 |
| Plays computer games | rs7196161 | 16 | 31110981 | A | G | 0.633 | 0.008 | 0.001 | 1.90E-12 |  | 0.009 | 0.009 | 0.315 | 1.07E-04 | 49.586 |
| Plays computer games | rs7234933 | 18 | 39692681 | C | T | 0.101 | 0.01 | 0.002 | 4.00E-08 |  | 0.003 | 0.016 | 0.834 | 6.52E-05 | 30.13 |
| Plays computer games | rs7431945 | 3 | 44029095 | A | G | 0.232 | 0.008 | 0.001 | 2.70E-10 |  | 0.022 | 0.012 | 0.053 | 8.63E-05 | 39.913 |
| Plays computer games | rs76563988 | 15 | 47947718 | G | A | 0.248 | -0.007 | 0.001 | 4.50E-09 |  | -0.024 | 0.011 | 0.023 | 7.44E-05 | 34.388 |
| Plays computer games | rs78081759 | 19 | 51130909 | A | G | 0.066 | 0.012 | 0.002 | 4.00E-09 |  | 0.033 | 0.02 | 0.091 | 7.49E-05 | 34.616 |
| Plays computer games | rs7824395 | 8 | 118922172 | G | A | 0.315 | -0.007 | 0.001 | 2.60E-09 |  | 0.011 | 0.01 | 0.287 | 7.66E-05 | 35.445 |
| Plays computer games | rs7907706 | 10 | 128491019 | A | G | 0.764 | 0.007 | 0.001 | 4.50E-08 |  | -0.013 | 0.011 | 0.247 | 6.47E-05 | 29.922 |
| Plays computer games | rs8062624 | 16 | 10168497 | C | T | 0.114 | 0.011 | 0.002 | 8.00E-11 |  | -0.025 | 0.015 | 0.106 | 9.14E-05 | 42.261 |
| Plays computer games | rs9444043 | 6 | 83915414 | C | A | 0.376 | -0.006 | 0.001 | 1.90E-09 |  | 0.008 | 0.01 | 0.42 | 7.80E-05 | 36.075 |
| Plays computer games | rs9661614 | 1 | 110021837 | C | T | 0.297 | 0.007 | 0.001 | 2.50E-10 |  | -0.018 | 0.01 | 0.073 | 8.66E-05 | 40.028 |
| Plays computer games | rs9688977 | 6 | 154336892 | C | T | 0.145 | 0.009 | 0.001 | 4.50E-09 |  | 0.032 | 0.013 | 0.012 | 7.43E-05 | 34.377 |
| Time spent using computer | rs10208088 | 2 | 221055873 | T | C | 0.58 | -0.01 | 0.002 | 3.00E-08 |  | 0.01 | 0.009 | 0.234 | 8.50E-05 | 30.685 |
| Time spent using computer | rs1037091 | 2 | 155652357 | T | C | 0.327 | -0.015 | 0.002 | 5.90E-15 |  | 0.006 | 0.01 | 0.568 | 1.69E-04 | 60.918 |
| Time spent using computer | rs10518019 | 4 | 67959875 | G | A | 0.476 | 0.01 | 0.002 | 4.90E-08 |  | -0.017 | 0.01 | 0.071 | 8.24E-05 | 29.738 |
| Time spent using computer | rs10828248 | 10 | 21824619 | G | A | 0.345 | 0.011 | 0.002 | 2.80E-08 |  | 0.02 | 0.01 | 0.038 | 8.55E-05 | 30.846 |
| Time spent using computer | rs11259902 | 15 | 83886529 | A | C | 0.2 | 0.013 | 0.002 | 1.60E-08 |  | 0.01 | 0.011 | 0.365 | 8.84E-05 | 31.895 |
| Time spent using computer | rs112600282 | 2 | 156895797 | G | A | 0.11 | -0.017 | 0.003 | 7.20E-09 |  | -0.006 | 0.014 | 0.684 | 9.28E-05 | 33.481 |
| Time spent using computer | rs113851275 | 9 | 98297220 | A | G | 0.108 | 0.019 | 0.003 | 2.50E-10 |  | 0.007 | 0.014 | 0.598 | 1.11E-04 | 39.994 |
| Time spent using computer | rs11634155 | 15 | 26693096 | C | T | 0.335 | -0.012 | 0.002 | 2.50E-09 |  | 0.014 | 0.01 | 0.177 | 9.85E-05 | 35.556 |
| Time spent using computer | rs11766392 | 7 | 69838127 | T | G | 0.285 | -0.013 | 0.002 | 3.10E-10 |  | 0.022 | 0.012 | 0.063 | 1.10E-04 | 39.584 |
| Time spent using computer | rs11942953 | 4 | 163753973 | C | T | 0.537 | -0.01 | 0.002 | 1.30E-08 |  | 0.029 | 0.01 | 0.002 | 8.96E-05 | 32.324 |
| Time spent using computer | rs12128707 | 1 | 72588119 | G | A | 0.264 | 0.012 | 0.002 | 7.10E-09 |  | 0.01 | 0.011 | 0.366 | 9.29E-05 | 33.517 |
| Time spent using computer | rs12145677 | 1 | 110023610 | A | G | 0.297 | 0.016 | 0.002 | 6.40E-15 |  | -0.018 | 0.01 | 0.066 | 1.68E-04 | 60.762 |
| Time spent using computer | rs1229984 | 4 | 100239319 | C | T | 0.973 | -0.034 | 0.006 | 4.40E-10 |  | -0.072 | 0.038 | 0.059 | 1.08E-04 | 38.91 |
| Time spent using computer | rs12521638 | 5 | 166458770 | G | A | 0.447 | 0.01 | 0.002 | 4.20E-08 |  | 0.003 | 0.01 | 0.738 | 8.33E-05 | 30.056 |
| Time spent using computer | rs12706626 | 7 | 124531370 | A | G | 0.384 | 0.01 | 0.002 | 2.90E-08 |  | 0.008 | 0.01 | 0.4 | 8.52E-05 | 30.762 |
| Time spent using computer | rs13262595 | 8 | 143316970 | G | A | 0.561 | 0.016 | 0.002 | 1.80E-17 |  | 0.006 | 0.009 | 0.547 | 2.00E-04 | 72.37 |
| Time spent using computer | rs13422733 | 2 | 102010245 | T | C | 0.126 | -0.015 | 0.003 | 4.10E-08 |  | 0.004 | 0.015 | 0.804 | 8.34E-05 | 30.115 |
| Time spent using computer | rs136553 | 22 | 27255675 | T | C | 0.377 | 0.011 | 0.002 | 2.00E-09 |  | 0 | 0.01 | 0.988 | 9.97E-05 | 35.994 |
| Time spent using computer | rs1395020 | 4 | 139690326 | A | G | 0.303 | -0.011 | 0.002 | 3.40E-08 |  | 0.012 | 0.01 | 0.217 | 8.44E-05 | 30.465 |
| Time spent using computer | rs1448355 | 11 | 131286685 | T | C | 0.618 | 0.012 | 0.002 | 8.00E-11 |  | -0.004 | 0.01 | 0.673 | 1.17E-04 | 42.262 |
| Time spent using computer | rs1469249 | 5 | 113837198 | A | G | 0.211 | -0.013 | 0.002 | 8.00E-09 |  | -0.007 | 0.011 | 0.533 | 9.22E-05 | 33.277 |
| Time spent using computer | rs147543875 | 10 | 101624164 | T | C | 0.023 | -0.036 | 0.006 | 1.50E-08 |  | 0.031 | 0.04 | 0.434 | 8.86E-05 | 31.993 |
| Time spent using computer | rs1648906 | 18 | 35311651 | A | G | 0.321 | -0.011 | 0.002 | 2.10E-08 |  | 0.008 | 0.01 | 0.393 | 8.71E-05 | 31.428 |
| Time spent using computer | rs166835 | 15 | 47716037 | T | C | 0.556 | -0.011 | 0.002 | 4.30E-09 |  | 0.006 | 0.009 | 0.531 | 9.55E-05 | 34.481 |
| Time spent using computer | rs16912540 | 11 | 13271422 | G | A | 0.137 | -0.016 | 0.003 | 2.70E-09 |  | 0.001 | 0.014 | 0.96 | 9.80E-05 | 35.363 |
| Time spent using computer | rs17167210 | 7 | 133339343 | A | G | 0.436 | -0.011 | 0.002 | 6.00E-10 |  | 0.022 | 0.009 | 0.015 | 1.06E-04 | 38.306 |
| Time spent using computer | rs17789218 | 6 | 100600097 | C | T | 0.245 | 0.013 | 0.002 | 2.10E-09 |  | 0.011 | 0.011 | 0.342 | 9.94E-05 | 35.872 |
| Time spent using computer | rs17862355 | 7 | 126970135 | G | T | 0.442 | -0.011 | 0.002 | 1.00E-09 |  | -0.009 | 0.009 | 0.334 | 1.03E-04 | 37.243 |
| Time spent using computer | rs1987942 | 13 | 54004785 | C | T | 0.617 | -0.011 | 0.002 | 5.40E-09 |  | -0.02 | 0.01 | 0.034 | 9.43E-05 | 34.046 |
| Time spent using computer | rs2032780 | 2 | 215073935 | C | T | 0.406 | 0.012 | 0.002 | 4.50E-11 |  | -0.008 | 0.01 | 0.432 | 1.20E-04 | 43.374 |
| Time spent using computer | rs2041687 | 2 | 60405620 | G | T | 0.549 | 0.012 | 0.002 | 3.80E-10 |  | 0.024 | 0.01 | 0.012 | 1.09E-04 | 39.23 |
| Time spent using computer | rs2068625 | 4 | 159856739 | C | T | 0.698 | 0.014 | 0.002 | 1.00E-12 |  | -0.024 | 0.01 | 0.02 | 1.41E-04 | 50.776 |
| Time spent using computer | rs206965 | 12 | 120856332 | C | T | 0.791 | -0.013 | 0.002 | 7.90E-09 |  | 0.023 | 0.012 | 0.058 | 9.23E-05 | 33.296 |
| Time spent using computer | rs2120461 | 1 | 8447722 | T | C | 0.661 | 0.012 | 0.002 | 2.50E-10 |  | 0.008 | 0.01 | 0.441 | 1.11E-04 | 40.002 |
| Time spent using computer | rs246723 | 5 | 140519166 | G | A | 0.59 | -0.011 | 0.002 | 1.20E-08 |  | 0.016 | 0.01 | 0.104 | 9.01E-05 | 32.502 |
| Time spent using computer | rs2588543 | 4 | 37000406 | T | C | 0.672 | 0.011 | 0.002 | 2.20E-08 |  | -0.019 | 0.011 | 0.083 | 8.67E-05 | 31.287 |
| Time spent using computer | rs2734833 | 11 | 113292920 | A | G | 0.607 | 0.012 | 0.002 | 3.80E-11 |  | -0.025 | 0.01 | 0.009 | 1.21E-04 | 43.725 |
| Time spent using computer | rs2748985 | 1 | 1853184 | C | T | 0.544 | 0.012 | 0.002 | 9.40E-12 |  | 0.007 | 0.01 | 0.5 | 1.29E-04 | 46.447 |
| Time spent using computer | rs2761438 | 1 | 110752139 | G | A | 0.624 | -0.011 | 0.002 | 1.70E-09 |  | 0.021 | 0.01 | 0.029 | 1.00E-04 | 36.273 |
| Time spent using computer | rs28710456 | 4 | 152667171 | C | T | 0.49 | -0.01 | 0.002 | 1.20E-08 |  | -0.008 | 0.009 | 0.379 | 8.98E-05 | 32.426 |
| Time spent using computer | rs306755 | 20 | 3099752 | C | T | 0.475 | 0.01 | 0.002 | 1.40E-08 |  | 0.012 | 0.009 | 0.189 | 8.92E-05 | 32.178 |
| Time spent using computer | rs34238696 | 5 | 161356241 | G | A | 0.107 | -0.017 | 0.003 | 1.30E-08 |  | 0 | 0.015 | 0.984 | 8.94E-05 | 32.268 |
| Time spent using computer | rs3730399 | 16 | 67229019 | G | A | 0.064 | -0.023 | 0.004 | 9.10E-10 |  | -0.025 | 0.018 | 0.16 | 1.04E-04 | 37.507 |
| Time spent using computer | rs4704043 | 5 | 72159179 | T | C | 0.714 | 0.012 | 0.002 | 8.90E-09 |  | 0.01 | 0.01 | 0.314 | 9.16E-05 | 33.075 |
| Time spent using computer | rs4852252 | 2 | 71539301 | C | T | 0.564 | 0.01 | 0.002 | 3.00E-08 |  | 0.009 | 0.009 | 0.353 | 8.51E-05 | 30.718 |
| Time spent using computer | rs56229818 | 15 | 58662232 | C | T | 0.484 | -0.01 | 0.002 | 3.00E-08 |  | -0.005 | 0.009 | 0.589 | 8.51E-05 | 30.715 |
| Time spent using computer | rs58638214 | 19 | 31864938 | T | C | 0.398 | -0.013 | 0.002 | 6.70E-12 |  | 0.017 | 0.01 | 0.079 | 1.31E-04 | 47.117 |
| Time spent using computer | rs6028090 | 20 | 59856465 | A | G | 0.555 | 0.013 | 0.002 | 1.30E-11 |  | 0.001 | 0.01 | 0.897 | 1.27E-04 | 45.87 |
| Time spent using computer | rs613872 | 18 | 53210302 | T | G | 0.827 | -0.016 | 0.002 | 7.70E-11 |  | 0 | 0.012 | 0.977 | 1.17E-04 | 42.329 |
| Time spent using computer | rs6449708 | 5 | 50851575 | C | T | 0.532 | -0.011 | 0.002 | 7.90E-09 |  | -0.001 | 0.009 | 0.896 | 9.23E-05 | 33.302 |
| Time spent using computer | rs6780848 | 3 | 8179920 | G | T | 0.27 | 0.011 | 0.002 | 3.30E-08 |  | -0.024 | 0.011 | 0.022 | 8.46E-05 | 30.541 |
| Time spent using computer | rs6935828 | 6 | 140811367 | T | C | 0.556 | 0.01 | 0.002 | 3.10E-08 |  | -0.005 | 0.01 | 0.6 | 8.49E-05 | 30.65 |
| Time spent using computer | rs7020477 | 9 | 116827760 | G | A | 0.267 | -0.012 | 0.002 | 7.00E-09 |  | 0.002 | 0.012 | 0.878 | 9.29E-05 | 33.548 |
| Time spent using computer | rs707926 | 6 | 31748820 | A | G | 0.15 | 0.015 | 0.003 | 1.20E-08 |  | 0.015 | 0.012 | 0.219 | 9.02E-05 | 32.543 |
| Time spent using computer | rs7209653 | 17 | 19882084 | C | T | 0.295 | -0.013 | 0.002 | 2.20E-11 |  | -0.003 | 0.01 | 0.761 | 1.24E-04 | 44.772 |
| Time spent using computer | rs7281293 | 21 | 34291496 | C | A | 0.248 | 0.013 | 0.002 | 3.00E-09 |  | -0.025 | 0.011 | 0.027 | 9.75E-05 | 35.189 |
| Time spent using computer | rs72828532 | 6 | 19065342 | C | T | 0.179 | 0.016 | 0.002 | 4.50E-11 |  | 0.015 | 0.012 | 0.233 | 1.20E-04 | 43.367 |
| Time spent using computer | rs72847500 | 6 | 37643909 | C | T | 0.121 | 0.016 | 0.003 | 1.90E-08 |  | -0.028 | 0.014 | 0.049 | 8.76E-05 | 31.623 |
| Time spent using computer | rs7288455 | 22 | 39966547 | G | A | 0.567 | -0.011 | 0.002 | 1.10E-08 |  | -0.007 | 0.01 | 0.432 | 9.05E-05 | 32.647 |
| Time spent using computer | rs73578186 | 9 | 126334485 | T | C | 0.324 | -0.012 | 0.002 | 3.70E-10 |  | -0.007 | 0.01 | 0.49 | 1.09E-04 | 39.264 |
| Time spent using computer | rs7526112 | 1 | 93747683 | G | T | 0.362 | -0.011 | 0.002 | 1.10E-08 |  | -0.006 | 0.01 | 0.562 | 9.05E-05 | 32.653 |
| Time spent using computer | rs75550998 | 2 | 146486095 | T | G | 0.054 | -0.022 | 0.004 | 4.60E-08 |  | -0.008 | 0.02 | 0.684 | 8.28E-05 | 29.893 |
| Time spent using computer | rs7564844 | 2 | 215335556 | A | G | 0.701 | -0.012 | 0.002 | 5.60E-10 |  | -0.006 | 0.01 | 0.587 | 1.07E-04 | 38.453 |
| Time spent using computer | rs7630869 | 3 | 49522543 | T | C | 0.304 | 0.016 | 0.002 | 4.00E-16 |  | -0.023 | 0.01 | 0.027 | 1.84E-04 | 66.249 |
| Time spent using computer | rs76824303 | 3 | 62459819 | C | A | 0.1 | -0.02 | 0.003 | 5.80E-11 |  | -0.04 | 0.016 | 0.012 | 1.19E-04 | 42.891 |
| Time spent using computer | rs7904398 | 10 | 67954193 | T | C | 0.503 | -0.01 | 0.002 | 2.40E-08 |  | -0.01 | 0.009 | 0.287 | 8.63E-05 | 31.135 |
| Time spent using computer | rs7968738 | 12 | 90281747 | A | G | 0.264 | -0.012 | 0.002 | 2.40E-09 |  | 0.009 | 0.011 | 0.42 | 9.88E-05 | 35.659 |
| Time spent using computer | rs79720045 | 4 | 39797668 | C | T | 0.401 | -0.013 | 0.002 | 1.80E-11 |  | 0.015 | 0.011 | 0.168 | 1.25E-04 | 45.23 |
| Time spent using computer | rs806795 | 6 | 26205293 | A | G | 0.47 | 0.01 | 0.002 | 1.40E-08 |  | 0.001 | 0.009 | 0.919 | 8.93E-05 | 32.23 |
| Time spent using computer | rs8102851 | 19 | 32208909 | C | T | 0.217 | 0.013 | 0.002 | 7.60E-09 |  | 0.009 | 0.014 | 0.524 | 9.25E-05 | 33.383 |
| Time spent using computer | rs9375188 | 6 | 98555272 | T | C | 0.484 | 0.016 | 0.002 | 5.00E-18 |  | -0.005 | 0.009 | 0.622 | 2.07E-04 | 74.896 |
| Time spent using computer | rs9537571 | 13 | 57604700 | A | G | 0.096 | 0.019 | 0.003 | 1.10E-09 |  | -0.013 | 0.017 | 0.434 | 1.03E-04 | 37.22 |
| Time spent watching television | rs10109061 | 8 | 144239859 | G | A | 0.443 | -0.009 | 0.002 | 3.10E-08 |  | 0.004 | 0.01 | 0.69 | 7.00E-05 | 30.672 |
| Time spent watching television | rs10189857 | 2 | 60713235 | G | A | 0.432 | 0.015 | 0.002 | 5.70E-20 |  | 0.02 | 0.009 | 0.035 | 1.91E-04 | 83.718 |
| Time spent watching television | rs10269099 | 7 | 126371011 | T | G | 0.391 | 0.009 | 0.002 | 2.10E-08 |  | 0.014 | 0.01 | 0.141 | 7.17E-05 | 31.412 |
| Time spent watching television | rs10765777 | 11 | 95656385 | C | A | 0.392 | -0.011 | 0.002 | 4.90E-12 |  | -0.003 | 0.01 | 0.755 | 1.09E-04 | 47.73 |
| Time spent watching television | rs111901094 | 19 | 19513570 | T | G | 0.182 | 0.013 | 0.002 | 1.40E-09 |  | 0.051 | 0.014 | 0 | 8.39E-05 | 36.72 |
| Time spent watching television | rs11191129 | 10 | 103606543 | T | C | 0.423 | -0.01 | 0.002 | 9.30E-10 |  | -0.025 | 0.011 | 0.022 | 8.56E-05 | 37.466 |
| Time spent watching television | rs11222919 | 11 | 131969663 | G | T | 0.175 | -0.012 | 0.002 | 6.30E-09 |  | -0.025 | 0.012 | 0.044 | 7.71E-05 | 33.75 |
| Time spent watching television | rs11245482 | 10 | 126733546 | C | T | 0.385 | 0.01 | 0.002 | 4.80E-10 |  | -0.008 | 0.01 | 0.417 | 8.85E-05 | 38.772 |
| Time spent watching television | rs114755463 | 5 | 152503110 | A | G | 0.168 | 0.013 | 0.002 | 6.70E-10 |  | 0.012 | 0.013 | 0.35 | 8.70E-05 | 38.096 |
| Time spent watching television | rs115608101 | 11 | 107106532 | T | C | 0.13 | -0.014 | 0.002 | 1.10E-08 |  | -0.003 | 0.013 | 0.837 | 7.44E-05 | 32.599 |
| Time spent watching television | rs11662211 | 18 | 77618869 | T | C | 0.506 | -0.009 | 0.002 | 2.90E-09 |  | -0.031 | 0.011 | 0.005 | 8.05E-05 | 35.242 |
| Time spent watching television | rs11680095 | 2 | 181825956 | T | C | 0.593 | -0.009 | 0.002 | 1.90E-08 |  | -0.006 | 0.01 | 0.505 | 7.22E-05 | 31.619 |
| Time spent watching television | rs11696187 | 20 | 58891882 | T | C | 0.16 | -0.014 | 0.002 | 4.20E-10 |  | -0.021 | 0.013 | 0.094 | 8.91E-05 | 39.029 |
| Time spent watching television | rs11700249 | 20 | 11910800 | G | T | 0.409 | 0.009 | 0.002 | 5.90E-09 |  | 0.006 | 0.011 | 0.6 | 7.73E-05 | 33.86 |
| Time spent watching television | rs11714337 | 3 | 71582521 | A | G | 0.43 | -0.01 | 0.002 | 7.60E-10 |  | -0.045 | 0.01 | 0 | 8.64E-05 | 37.855 |
| Time spent watching television | rs11877758 | 18 | 35138110 | G | T | 0.313 | 0.011 | 0.002 | 1.30E-10 |  | 0.029 | 0.01 | 0.004 | 9.43E-05 | 41.285 |
| Time spent watching television | rs11911112 | 21 | 40528346 | C | A | 0.366 | -0.011 | 0.002 | 8.00E-11 |  | 0.006 | 0.01 | 0.543 | 9.65E-05 | 42.247 |
| Time spent watching television | rs12045585 | 1 | 243673099 | A | G | 0.131 | -0.015 | 0.002 | 4.10E-10 |  | -0.038 | 0.013 | 0.005 | 8.92E-05 | 39.068 |
| Time spent watching television | rs12214364 | 6 | 67556372 | G | T | 0.415 | 0.009 | 0.002 | 3.80E-08 |  | 0.012 | 0.01 | 0.204 | 6.90E-05 | 30.234 |
| Time spent watching television | rs1291871 | 10 | 11086083 | C | T | 0.514 | 0.009 | 0.002 | 9.90E-09 |  | 0.02 | 0.009 | 0.03 | 7.50E-05 | 32.862 |
| Time spent watching television | rs13014947 | 2 | 193742999 | A | G | 0.575 | 0.011 | 0.002 | 6.50E-11 |  | 0.016 | 0.01 | 0.098 | 9.74E-05 | 42.663 |
| Time spent watching television | rs13107325 | 4 | 103188709 | T | C | 0.075 | 0.02 | 0.003 | 1.70E-11 |  | 0.005 | 0.024 | 0.844 | 1.04E-04 | 45.326 |
| Time spent watching television | rs1324491 | 1 | 60350616 | A | G | 0.131 | 0.013 | 0.002 | 1.30E-08 |  | 0.026 | 0.014 | 0.059 | 7.39E-05 | 32.37 |
| Time spent watching television | rs1727332 | 12 | 123718301 | T | C | 0.754 | 0.013 | 0.002 | 1.10E-12 |  | 0.049 | 0.011 | 0 | 1.16E-04 | 50.601 |
| Time spent watching television | rs17789218 | 6 | 100600097 | C | T | 0.244 | -0.011 | 0.002 | 3.60E-09 |  | 0.011 | 0.011 | 0.342 | 7.96E-05 | 34.845 |
| Time spent watching television | rs178203 | 14 | 26959322 | C | T | 0.75 | 0.012 | 0.002 | 5.90E-11 |  | 0.015 | 0.011 | 0.165 | 9.78E-05 | 42.845 |
| Time spent watching television | rs180396 | 13 | 60437497 | T | C | 0.747 | 0.01 | 0.002 | 4.30E-08 |  | 0.011 | 0.011 | 0.296 | 6.85E-05 | 30.007 |
| Time spent watching television | rs184332798 | 18 | 53373610 | A | G | 0.026 | -0.028 | 0.005 | 2.80E-08 |  | -0.042 | 0.034 | 0.211 | 7.04E-05 | 30.815 |
| Time spent watching television | rs1889996 | 13 | 54269950 | G | T | 0.739 | 0.012 | 0.002 | 3.90E-11 |  | -0.008 | 0.011 | 0.458 | 9.97E-05 | 43.664 |
| Time spent watching television | rs1993092 | 6 | 98689604 | C | T | 0.39 | -0.01 | 0.002 | 5.40E-09 |  | -0.04 | 0.01 | 0 | 7.78E-05 | 34.051 |
| Time spent watching television | rs2073869 | 9 | 135763816 | T | C | 0.167 | -0.014 | 0.002 | 1.10E-10 |  | -0.019 | 0.013 | 0.123 | 9.50E-05 | 41.61 |
| Time spent watching television | rs2106164 | 7 | 92661753 | C | T | 0.532 | -0.009 | 0.002 | 4.20E-09 |  | -0.033 | 0.009 | 0 | 7.88E-05 | 34.513 |
| Time spent watching television | rs2185490 | 14 | 69732119 | C | A | 0.618 | 0.009 | 0.002 | 1.20E-08 |  | 0.026 | 0.01 | 0.008 | 7.41E-05 | 32.471 |
| Time spent watching television | rs2240857 | 7 | 8010634 | G | T | 0.141 | 0.016 | 0.002 | 4.50E-12 |  | 0.028 | 0.014 | 0.04 | 1.09E-04 | 47.89 |
| Time spent watching television | rs2283 | 5 | 106773623 | G | A | 0.339 | -0.009 | 0.002 | 3.20E-08 |  | -0.021 | 0.01 | 0.037 | 6.99E-05 | 30.592 |
| Time spent watching television | rs2352984 | 3 | 49948728 | C | T | 0.431 | 0.02 | 0.002 | 7.40E-35 |  | 0.039 | 0.009 | 0 | 3.46E-04 | 151.702 |
| Time spent watching television | rs2479968 | 13 | 111969328 | G | A | 0.051 | 0.021 | 0.004 | 2.70E-08 |  | 0.051 | 0.02 | 0.01 | 7.06E-05 | 30.922 |
| Time spent watching television | rs249960 | 5 | 96164771 | G | A | 0.182 | -0.012 | 0.002 | 1.10E-08 |  | -0.015 | 0.012 | 0.199 | 7.45E-05 | 32.633 |
| Time spent watching television | rs262890 | 5 | 62930015 | G | A | 0.299 | 0.013 | 0.002 | 2.60E-14 |  | 0.006 | 0.01 | 0.592 | 1.33E-04 | 58.042 |
| Time spent watching television | rs263771 | 2 | 185921692 | A | C | 0.233 | 0.012 | 0.002 | 1.90E-10 |  | 0.022 | 0.011 | 0.05 | 9.27E-05 | 40.602 |
| Time spent watching television | rs2646351 | 4 | 55701312 | A | G | 0.453 | 0.009 | 0.002 | 2.40E-08 |  | -0.008 | 0.009 | 0.382 | 7.10E-05 | 31.101 |
| Time spent watching television | rs2678662 | 2 | 104446759 | G | T | 0.608 | 0.011 | 0.002 | 8.60E-12 |  | 0.041 | 0.01 | 0 | 1.06E-04 | 46.621 |
| Time spent watching television | rs2725371 | 8 | 30854033 | G | A | 0.696 | -0.013 | 0.002 | 2.10E-14 |  | -0.019 | 0.01 | 0.063 | 1.34E-04 | 58.479 |
| Time spent watching television | rs2857693 | 6 | 31588384 | T | G | 0.366 | 0.011 | 0.002 | 9.60E-11 |  | -0.003 | 0.01 | 0.727 | 9.57E-05 | 41.906 |
| Time spent watching television | rs2906604 | 2 | 107624244 | C | T | 0.497 | 0.01 | 0.002 | 5.50E-11 |  | 0.011 | 0.01 | 0.257 | 9.82E-05 | 42.994 |
| Time spent watching television | rs3138499 | 9 | 92219921 | C | A | 0.518 | 0.011 | 0.002 | 8.50E-12 |  | -0.015 | 0.009 | 0.111 | 1.07E-04 | 46.658 |
| Time spent watching television | rs34811474 | 4 | 25408838 | A | G | 0.231 | -0.013 | 0.002 | 3.10E-12 |  | -0.027 | 0.011 | 0.016 | 1.11E-04 | 48.598 |
| Time spent watching television | rs35797019 | 3 | 93987306 | G | A | 0.392 | -0.009 | 0.002 | 1.50E-08 |  | -0.025 | 0.01 | 0.009 | 7.32E-05 | 32.04 |
| Time spent watching television | rs362312 | 4 | 3237644 | C | T | 0.424 | -0.01 | 0.002 | 3.50E-09 |  | -0.001 | 0.01 | 0.931 | 7.97E-05 | 34.892 |
| Time spent watching television | rs3754970 | 2 | 162091836 | C | T | 0.503 | 0.01 | 0.002 | 1.30E-09 |  | 0.019 | 0.01 | 0.041 | 8.41E-05 | 36.828 |
| Time spent watching television | rs3810496 | 20 | 62406886 | C | T | 0.616 | 0.009 | 0.002 | 1.20E-08 |  | 0.01 | 0.01 | 0.331 | 7.42E-05 | 32.482 |
| Time spent watching television | rs4076457 | 15 | 78007213 | T | C | 0.257 | -0.01 | 0.002 | 3.70E-08 |  | -0.035 | 0.011 | 0.002 | 6.92E-05 | 30.298 |
| Time spent watching television | rs4110177 | 5 | 88793281 | A | G | 0.367 | 0.009 | 0.002 | 1.30E-08 |  | 0.018 | 0.01 | 0.064 | 7.39E-05 | 32.375 |
| Time spent watching television | rs4303732 | 2 | 100830040 | C | T | 0.402 | -0.011 | 0.002 | 3.70E-11 |  | -0.015 | 0.009 | 0.107 | 1.00E-04 | 43.779 |
| Time spent watching television | rs4339469 | 6 | 98369230 | G | T | 0.629 | 0.013 | 0.002 | 2.80E-14 |  | 0.009 | 0.01 | 0.346 | 1.32E-04 | 57.861 |
| Time spent watching television | rs4469687 | 1 | 184679019 | G | A | 0.484 | 0.009 | 0.002 | 3.10E-08 |  | 0.03 | 0.009 | 0.001 | 6.99E-05 | 30.621 |
| Time spent watching television | rs4567133 | 9 | 22606560 | A | C | 0.81 | -0.013 | 0.002 | 1.70E-10 |  | -0.032 | 0.012 | 0.006 | 9.32E-05 | 40.821 |
| Time spent watching television | rs4747438 | 10 | 22124263 | T | C | 0.677 | -0.012 | 0.002 | 3.90E-12 |  | -0.02 | 0.01 | 0.043 | 1.10E-04 | 48.152 |
| Time spent watching television | rs4788616 | 16 | 72211984 | G | T | 0.391 | -0.01 | 0.002 | 4.30E-10 |  | -0.01 | 0.01 | 0.334 | 8.90E-05 | 38.982 |
| Time spent watching television | rs494566 | 9 | 1785717 | T | C | 0.332 | 0.01 | 0.002 | 2.00E-09 |  | 0.003 | 0.01 | 0.805 | 8.20E-05 | 35.93 |
| Time spent watching television | rs57555420 | 1 | 97783448 | T | C | 0.282 | 0.01 | 0.002 | 9.40E-09 |  | 0.009 | 0.011 | 0.376 | 7.53E-05 | 32.957 |
| Time spent watching television | rs58541850 | 6 | 166165563 | A | G | 0.059 | 0.021 | 0.003 | 2.70E-10 |  | -0.003 | 0.021 | 0.9 | 9.10E-05 | 39.872 |
| Time spent watching television | rs6102912 | 20 | 41202935 | C | T | 0.41 | -0.011 | 0.002 | 1.40E-11 |  | -0.02 | 0.009 | 0.031 | 1.04E-04 | 45.603 |
| Time spent watching television | rs6125907 | 20 | 48730315 | A | C | 0.092 | 0.016 | 0.003 | 4.50E-09 |  | -0.015 | 0.02 | 0.439 | 7.85E-05 | 34.381 |
| Time spent watching television | rs62145951 | 2 | 68399586 | C | T | 0.263 | -0.012 | 0.002 | 2.60E-11 |  | -0.026 | 0.011 | 0.018 | 1.01E-04 | 44.433 |
| Time spent watching television | rs62199883 | 2 | 215376706 | A | C | 0.486 | 0.014 | 0.002 | 3.20E-18 |  | 0.026 | 0.009 | 0.006 | 1.73E-04 | 75.768 |
| Time spent watching television | rs6511708 | 19 | 10788813 | C | T | 0.665 | -0.012 | 0.002 | 7.50E-13 |  | -0.006 | 0.01 | 0.592 | 1.17E-04 | 51.41 |
| Time spent watching television | rs68056254 | 2 | 147846855 | T | G | 0.151 | 0.013 | 0.002 | 1.30E-09 |  | 0.019 | 0.013 | 0.129 | 8.40E-05 | 36.792 |
| Time spent watching television | rs6814554 | 4 | 152454334 | A | G | 0.474 | 0.014 | 0.002 | 2.40E-17 |  | -0.012 | 0.009 | 0.221 | 1.64E-04 | 71.822 |
| Time spent watching television | rs6850494 | 4 | 82291771 | C | A | 0.386 | 0.01 | 0.002 | 3.40E-09 |  | 0.002 | 0.01 | 0.852 | 7.98E-05 | 34.938 |
| Time spent watching television | rs6895658 | 5 | 124274035 | C | T | 0.193 | -0.013 | 0.002 | 1.10E-10 |  | -0.013 | 0.012 | 0.306 | 9.50E-05 | 41.583 |
| Time spent watching television | rs6994132 | 8 | 92653740 | C | T | 0.579 | -0.01 | 0.002 | 6.20E-10 |  | -0.013 | 0.009 | 0.164 | 8.73E-05 | 38.25 |
| Time spent watching television | rs7089973 | 10 | 116569565 | A | C | 0.38 | 0.009 | 0.002 | 1.60E-08 |  | -0.013 | 0.01 | 0.174 | 7.28E-05 | 31.892 |
| Time spent watching television | rs7184800 | 16 | 53509131 | A | G | 0.303 | -0.013 | 0.002 | 7.40E-15 |  | -0.021 | 0.01 | 0.033 | 1.38E-04 | 60.476 |
| Time spent watching television | rs73571431 | 9 | 126136139 | T | C | 0.11 | 0.016 | 0.003 | 5.50E-10 |  | -0.016 | 0.015 | 0.28 | 8.79E-05 | 38.495 |
| Time spent watching television | rs73946726 | 2 | 117073427 | A | C | 0.02 | 0.032 | 0.006 | 2.20E-08 |  | 0.007 | 0.042 | 0.864 | 7.16E-05 | 31.349 |
| Time spent watching television | rs749056 | 1 | 110037838 | G | T | 0.304 | -0.01 | 0.002 | 7.20E-09 |  | -0.013 | 0.01 | 0.187 | 7.65E-05 | 33.481 |
| Time spent watching television | rs749671 | 16 | 31088347 | A | G | 0.372 | -0.011 | 0.002 | 1.60E-11 |  | -0.009 | 0.01 | 0.343 | 1.04E-04 | 45.388 |
| Time spent watching television | rs7539775 | 1 | 3109151 | A | G | 0.743 | 0.01 | 0.002 | 4.30E-08 |  | 0.005 | 0.013 | 0.697 | 6.85E-05 | 30.005 |
| Time spent watching television | rs75499503 | 6 | 26145217 | T | C | 0.22 | -0.018 | 0.002 | 3.10E-21 |  | -0.008 | 0.011 | 0.457 | 2.04E-04 | 89.463 |
| Time spent watching television | rs75641275 | 1 | 98327133 | C | A | 0.143 | 0.015 | 0.002 | 1.00E-11 |  | 0.014 | 0.013 | 0.277 | 1.06E-04 | 46.239 |
| Time spent watching television | rs7708324 | 5 | 147920094 | G | A | 0.377 | -0.01 | 0.002 | 1.60E-09 |  | 0.009 | 0.01 | 0.341 | 8.31E-05 | 36.404 |
| Time spent watching television | rs7798292 | 7 | 112974602 | A | G | 0.435 | -0.01 | 0.002 | 1.30E-09 |  | 0.002 | 0.009 | 0.832 | 8.40E-05 | 36.764 |
| Time spent watching television | rs78227853 | 15 | 44169073 | T | C | 0.025 | -0.028 | 0.005 | 3.40E-08 |  | -0.013 | 0.03 | 0.653 | 6.95E-05 | 30.452 |
| Time spent watching television | rs7899206 | 10 | 127188859 | G | T | 0.492 | -0.01 | 0.002 | 1.30E-09 |  | -0.019 | 0.01 | 0.053 | 8.42E-05 | 36.852 |
| Time spent watching television | rs7921305 | 10 | 133775196 | A | G | 0.253 | -0.011 | 0.002 | 3.70E-10 |  | -0.006 | 0.011 | 0.579 | 8.96E-05 | 39.257 |
| Time spent watching television | rs79373894 | 15 | 73369053 | C | T | 0.034 | -0.029 | 0.004 | 9.80E-11 |  | 0.01 | 0.034 | 0.775 | 9.56E-05 | 41.862 |
| Time spent watching television | rs801733 | 11 | 65934549 | C | A | 0.358 | -0.012 | 0.002 | 5.00E-14 |  | -0.006 | 0.01 | 0.525 | 1.30E-04 | 56.723 |
| Time spent watching television | rs814197 | 1 | 61092456 | G | T | 0.467 | -0.011 | 0.002 | 2.00E-11 |  | -0.02 | 0.009 | 0.035 | 1.03E-04 | 44.948 |
| Time spent watching television | rs898751 | 17 | 2291863 | T | C | 0.493 | 0.01 | 0.002 | 3.00E-10 |  | 0.019 | 0.01 | 0.057 | 9.06E-05 | 39.664 |
| Time spent watching television | rs9300594 | 13 | 100869905 | G | A | 0.254 | 0.011 | 0.002 | 9.10E-10 |  | 0.006 | 0.011 | 0.559 | 8.56E-05 | 37.504 |
| Time spent watching television | rs9471333 | 6 | 40362023 | T | C | 0.552 | -0.01 | 0.002 | 6.70E-11 |  | -0.003 | 0.009 | 0.735 | 9.73E-05 | 42.592 |
| Time spent watching television | rs9834970 | 3 | 36856030 | C | T | 0.498 | -0.009 | 0.002 | 2.00E-08 |  | -0.021 | 0.009 | 0.025 | 7.19E-05 | 31.491 |
| Time spent watching television | rs9867121 | 3 | 114631548 | A | C | 0.184 | -0.012 | 0.002 | 1.40E-08 |  | -0.013 | 0.012 | 0.284 | 7.36E-05 | 32.239 |
| Time spent watching television | rs9867437 | 3 | 85676752 | C | A | 0.46 | -0.01 | 0.002 | 1.30E-10 |  | -0.022 | 0.009 | 0.018 | 9.45E-05 | 41.366 |
| Time spent watching television | rs9880023 | 3 | 54178199 | T | G | 0.556 | 0.01 | 0.002 | 2.00E-09 |  | -0.001 | 0.009 | 0.924 | 8.22E-05 | 35.994 |
| Time spent watching television | rs996234 | 5 | 59455212 | A | G | 0.516 | -0.011 | 0.002 | 1.60E-10 |  | -0.012 | 0.009 | 0.187 | 9.34E-05 | 40.916 |

**Note:** Abbreviations: Beta, effect allele value; SE, standard error; SNP, single nucleotide polymorphism

**Table S3.** Genetic instruments for anxiety disorder

| **SNP** | **Chromosome** | **Position** | **Effect allele** | **Other allele** | **Effect allele frequency** | **Exposure** | | |  | **Outcome** | | | ***R^2^*** | ***F-Statistic*** |
| --- | --- | --- | --- | --- | --- | --- | --- | --- | --- | --- | --- | --- | --- | --- |
|  |  |  |  |  |  | ***Beta*** | ***SE*** | ***P-value*** |  | ***Beta*** | ***SE*** | ***P-value*** |  |  |
| rs10107145 | 8 | 10758213 | G | A | 0.545 | -0.018 | 0.003 | 1.70E-10 |  | 0.065 | 0.028 | 0.019 | 8.92E-05 | 40.785 |
| rs10807124 | 6 | 33404064 | A | G | 0.274 | -0.018 | 0.003 | 3.10E-08 |  | -0.024 | 0.032 | 0.452 | 6.70E-05 | 30.623 |
| rs10828247 | 10 | 21822856 | G | A | 0.344 | 0.017 | 0.003 | 7.40E-09 |  | -0.024 | 0.029 | 0.419 | 7.31E-05 | 33.426 |
| rs11229008 | 11 | 57118840 | A | G | 0.063 | -0.033 | 0.006 | 3.90E-08 |  | 0.026 | 0.064 | 0.687 | 6.61E-05 | 30.222 |
| rs11236714 | 11 | 70424559 | T | C | 0.196 | -0.02 | 0.004 | 1.80E-08 |  | 0.013 | 0.035 | 0.718 | 6.93E-05 | 31.661 |
| rs11655813 | 17 | 2119101 | T | C | 0.355 | 0.018 | 0.003 | 1.20E-09 |  | -0.023 | 0.027 | 0.406 | 8.10E-05 | 37.04 |
| rs11682846 | 2 | 157008703 | T | C | 0.485 | -0.017 | 0.003 | 9.90E-10 |  | -0.028 | 0.029 | 0.339 | 8.17E-05 | 37.352 |
| rs12145998 | 1 | 204969419 | T | C | 0.266 | -0.019 | 0.003 | 2.90E-09 |  | -0.048 | 0.03 | 0.111 | 7.71E-05 | 35.224 |
| rs12437348 | 14 | 36606550 | A | G | 0.71 | 0.017 | 0.003 | 4.20E-08 |  | 0.017 | 0.03 | 0.57 | 6.57E-05 | 30.033 |
| rs13266457 | 8 | 106086786 | T | C | 0.33 | -0.017 | 0.003 | 1.70E-08 |  | 0 | 0.028 | 0.992 | 6.97E-05 | 31.856 |
| rs1512142 | 4 | 47004305 | A | G | 0.443 | -0.017 | 0.003 | 8.30E-09 |  | -0.014 | 0.027 | 0.62 | 7.27E-05 | 33.205 |
| rs17156711 | 5 | 103924699 | G | A | 0.299 | -0.018 | 0.003 | 4.10E-09 |  | -0.033 | 0.028 | 0.251 | 7.56E-05 | 34.563 |
| rs17374152 | 5 | 93236948 | G | A | 0.239 | -0.019 | 0.003 | 5.30E-09 |  | -0.03 | 0.044 | 0.496 | 7.46E-05 | 34.088 |
| rs1892417 | 1 | 41779673 | C | T | 0.229 | 0.026 | 0.003 | 1.30E-14 |  | 0.018 | 0.032 | 0.57 | 1.30E-04 | 59.328 |
| rs2161220 | 5 | 80241904 | A | G | 0.248 | 0.021 | 0.003 | 4.00E-10 |  | 0.03 | 0.031 | 0.336 | 8.56E-05 | 39.118 |
| rs2836920 | 21 | 40512918 | G | T | 0.396 | 0.019 | 0.003 | 2.30E-10 |  | 0.006 | 0.031 | 0.859 | 8.80E-05 | 40.218 |
| rs28713780 | 7 | 3321415 | C | T | 0.641 | -0.017 | 0.003 | 1.10E-08 |  | -0.053 | 0.027 | 0.051 | 7.15E-05 | 32.685 |
| rs344868 | 2 | 140095800 | T | C | 0.247 | 0.018 | 0.003 | 3.20E-08 |  | -0.027 | 0.031 | 0.387 | 6.69E-05 | 30.553 |
| rs359265 | 2 | 60456410 | A | G | 0.607 | 0.021 | 0.003 | 6.40E-13 |  | 0.038 | 0.03 | 0.207 | 1.13E-04 | 51.715 |
| rs6063374 | 20 | 47832997 | G | A | 0.781 | 0.029 | 0.003 | 1.00E-17 |  | -0.038 | 0.036 | 0.283 | 1.61E-04 | 73.426 |
| rs6131703 | 20 | 15754684 | G | A | 0.386 | -0.018 | 0.003 | 1.90E-09 |  | 0.008 | 0.032 | 0.814 | 7.90E-05 | 36.121 |
| rs6780051 | 3 | 56193150 | T | G | 0.058 | 0.04 | 0.006 | 6.90E-11 |  | -0.038 | 0.107 | 0.72 | 9.31E-05 | 42.548 |
| rs78166132 | 5 | 161239986 | C | T | 0.094 | -0.031 | 0.005 | 4.90E-10 |  | 0.006 | 0.05 | 0.91 | 8.47E-05 | 38.728 |
| rs7859831 | 9 | 126205754 | T | C | 0.139 | -0.023 | 0.004 | 2.10E-08 |  | 0.053 | 0.036 | 0.14 | 6.87E-05 | 31.393 |
| rs8014346 | 14 | 46832002 | A | G | 0.535 | 0.019 | 0.003 | 3.70E-11 |  | 0.04 | 0.026 | 0.129 | 9.57E-05 | 43.758 |
| rs849527 | 2 | 206592525 | G | A | 0.547 | -0.016 | 0.003 | 1.50E-08 |  | 0.009 | 0.026 | 0.722 | 7.00E-05 | 31.995 |
| rs853946 | 10 | 120166472 | T | C | 0.468 | 0.016 | 0.003 | 1.80E-08 |  | -0.012 | 0.026 | 0.659 | 6.94E-05 | 31.707 |
| rs9896202 | 17 | 77778227 | C | T | 0.498 | -0.021 | 0.003 | 1.90E-13 |  | 0.029 | 0.031 | 0.352 | 1.18E-04 | 54.134 |
| rs10170573 | 2 | 225552916 | G | A | 0.654 | -0.006 | 0.001 | 4.00E-08 |  | -0.009 | 0.027 | 0.742 | 6.52E-05 | 30.147 |
| rs10799961 | 1 | 163744838 | G | A | 0.538 | 0.006 | 0.001 | 3.50E-08 |  | 0.052 | 0.027 | 0.049 | 6.57E-05 | 30.388 |
| rs10803798 | 2 | 166399307 | G | T | 0.284 | 0.008 | 0.001 | 7.80E-12 |  | 0.011 | 0.029 | 0.704 | 1.01E-04 | 46.811 |
| rs11223780 | 11 | 134261150 | G | A | 0.148 | 0.008 | 0.001 | 9.50E-09 |  | 0.032 | 0.04 | 0.425 | 7.12E-05 | 32.946 |
| rs113002196 | 1 | 27200156 | C | T | 0.16 | 0.008 | 0.001 | 7.50E-09 |  | 0.021 | 0.043 | 0.626 | 7.22E-05 | 33.411 |
| rs11740196 | 5 | 12855333 | G | A | 0.484 | -0.007 | 0.001 | 2.60E-11 |  | -0.013 | 0.037 | 0.713 | 9.61E-05 | 44.46 |
| rs11743441 | 5 | 88065637 | T | G | 0.574 | -0.007 | 0.001 | 3.60E-11 |  | -0.051 | 0.03 | 0.085 | 9.47E-05 | 43.817 |
| rs12129719 | 1 | 66324512 | A | G | 0.552 | 0.006 | 0.001 | 1.20E-09 |  | 0.058 | 0.03 | 0.054 | 8.00E-05 | 37 |
| rs12138787 | 1 | 150246070 | C | T | 0.124 | 0.009 | 0.002 | 1.10E-08 |  | -0.021 | 0.041 | 0.614 | 7.05E-05 | 32.605 |
| rs1232205 | 11 | 31661314 | C | T | 0.302 | -0.007 | 0.001 | 4.10E-09 |  | -0.071 | 0.031 | 0.023 | 7.48E-05 | 34.595 |
| rs12361956 | 11 | 40406823 | C | T | 0.39 | -0.006 | 0.001 | 4.10E-08 |  | -0.005 | 0.027 | 0.862 | 6.51E-05 | 30.107 |
| rs12707117 | 7 | 133429058 | A | G | 0.555 | 0.007 | 0.001 | 3.00E-10 |  | 0.006 | 0.026 | 0.821 | 8.58E-05 | 39.698 |
| rs13262595 | 8 | 143316970 | G | A | 0.561 | 0.008 | 0.001 | 6.30E-14 |  | -0.055 | 0.027 | 0.04 | 1.22E-04 | 56.287 |
| rs1406069 | 17 | 44225886 | A | G | 0.201 | 0.013 | 0.001 | 2.90E-21 |  | -0.054 | 0.071 | 0.446 | 1.94E-04 | 89.595 |
| rs16831510 | 2 | 200305460 | T | G | 0.128 | 0.01 | 0.002 | 3.90E-11 |  | -0.061 | 0.04 | 0.125 | 9.44E-05 | 43.674 |
| rs17057709 | 18 | 73378941 | T | C | 0.142 | -0.008 | 0.001 | 2.40E-08 |  | 0.033 | 0.051 | 0.512 | 6.73E-05 | 31.103 |
| rs2014653 | 20 | 50788476 | G | A | 0.298 | -0.008 | 0.001 | 3.70E-11 |  | 0.043 | 0.031 | 0.163 | 9.47E-05 | 43.774 |
| rs302719 | 1 | 8490320 | G | T | 0.34 | -0.006 | 0.001 | 6.00E-09 |  | 0.096 | 0.086 | 0.264 | 7.32E-05 | 33.848 |
| rs34217929 | 2 | 137485074 | A | G | 0.256 | 0.01 | 0.001 | 9.70E-16 |  | 0.037 | 0.035 | 0.294 | 1.39E-04 | 64.484 |
| rs34402857 | 2 | 156109104 | A | G | 0.519 | 0.008 | 0.001 | 7.90E-14 |  | 0.033 | 0.028 | 0.241 | 1.21E-04 | 55.833 |
| rs35104374 | 12 | 6739497 | C | T | 0.725 | -0.007 | 0.001 | 1.90E-08 |  | -0.023 | 0.043 | 0.59 | 6.82E-05 | 31.559 |
| rs3757589 | 7 | 73865349 | A | G | 0.389 | -0.006 | 0.001 | 1.80E-08 |  | -0.029 | 0.038 | 0.446 | 6.85E-05 | 31.691 |
| rs4113587 | 6 | 128311640 | G | T | 0.443 | 0.007 | 0.001 | 2.40E-11 |  | 0.004 | 0.027 | 0.897 | 9.65E-05 | 44.651 |
| rs411844 | 7 | 101692403 | A | G | 0.821 | -0.007 | 0.001 | 4.30E-08 |  | -0.018 | 0.039 | 0.649 | 6.49E-05 | 29.999 |
| rs4938017 | 11 | 113303929 | T | C | 0.393 | -0.01 | 0.001 | 2.00E-19 |  | 0.078 | 0.03 | 0.009 | 1.76E-04 | 81.22 |
| rs535591 | 3 | 83154118 | A | G | 0.788 | -0.007 | 0.001 | 3.80E-08 |  | -0.006 | 0.034 | 0.859 | 6.54E-05 | 30.236 |
| rs56275045 | 16 | 10201392 | A | C | 0.137 | 0.009 | 0.002 | 7.40E-09 |  | -0.075 | 0.044 | 0.086 | 7.23E-05 | 33.437 |
| rs580241 | 11 | 66066349 | A | G | 0.763 | -0.007 | 0.001 | 5.90E-09 |  | 0.008 | 0.036 | 0.819 | 7.32E-05 | 33.866 |
| rs62241000 | 22 | 42378507 | T | C | 0.321 | 0.006 | 0.001 | 2.90E-08 |  | 0.027 | 0.028 | 0.34 | 6.66E-05 | 30.788 |
| rs623863 | 11 | 88608915 | G | A | 0.651 | 0.006 | 0.001 | 5.90E-09 |  | -0.006 | 0.029 | 0.84 | 7.32E-05 | 33.854 |
| rs6457740 | 6 | 33697125 | G | A | 0.703 | -0.007 | 0.001 | 1.40E-09 |  | 0.007 | 0.029 | 0.812 | 7.93E-05 | 36.664 |
| rs6536378 | 4 | 159857227 | G | T | 0.653 | 0.006 | 0.001 | 3.70E-08 |  | -0.01 | 0.028 | 0.709 | 6.55E-05 | 30.279 |
| rs678007 | 1 | 23600579 | T | C | 0.178 | -0.008 | 0.001 | 3.40E-08 |  | 0.028 | 0.036 | 0.434 | 6.59E-05 | 30.473 |
| rs717915 | 2 | 200714766 | T | C | 0.635 | 0.006 | 0.001 | 3.50E-08 |  | 0.013 | 0.028 | 0.646 | 6.58E-05 | 30.417 |
| rs7196161 | 16 | 31110981 | A | G | 0.633 | 0.008 | 0.001 | 1.90E-12 |  | 0.02 | 0.03 | 0.514 | 1.07E-04 | 49.586 |
| rs7234933 | 18 | 39692681 | C | T | 0.101 | 0.01 | 0.002 | 4.00E-08 |  | -0.05 | 0.045 | 0.264 | 6.52E-05 | 30.13 |
| rs7431945 | 3 | 44029095 | A | G | 0.232 | 0.008 | 0.001 | 2.70E-10 |  | 0.061 | 0.032 | 0.055 | 8.63E-05 | 39.913 |
| rs76563988 | 15 | 47947718 | G | A | 0.248 | -0.007 | 0.001 | 4.50E-09 |  | -0.024 | 0.034 | 0.483 | 7.44E-05 | 34.388 |
| rs78081759 | 19 | 51130909 | A | G | 0.066 | 0.012 | 0.002 | 4.00E-09 |  | -0.081 | 0.087 | 0.352 | 7.49E-05 | 34.616 |
| rs7824395 | 8 | 118922172 | G | A | 0.315 | -0.007 | 0.001 | 2.60E-09 |  | 0.015 | 0.028 | 0.588 | 7.66E-05 | 35.445 |
| rs7907706 | 10 | 128491019 | A | G | 0.764 | 0.007 | 0.001 | 4.50E-08 |  | 0.039 | 0.038 | 0.311 | 6.47E-05 | 29.922 |
| rs8062624 | 16 | 10168497 | C | T | 0.114 | 0.011 | 0.002 | 8.00E-11 |  | -0.057 | 0.042 | 0.176 | 9.14E-05 | 42.261 |
| rs9444043 | 6 | 83915414 | C | A | 0.376 | -0.006 | 0.001 | 1.90E-09 |  | -0.033 | 0.028 | 0.235 | 7.80E-05 | 36.075 |
| rs9661614 | 1 | 110021837 | C | T | 0.297 | 0.007 | 0.001 | 2.50E-10 |  | 0.014 | 0.028 | 0.616 | 8.66E-05 | 40.028 |
| rs9688977 | 6 | 154336892 | C | T | 0.145 | 0.009 | 0.001 | 4.50E-09 |  | -0.036 | 0.037 | 0.326 | 7.43E-05 | 34.377 |
| rs9842435 | 3 | 117517339 | G | A | 0.876 | -0.011 | 0.002 | 2.10E-12 |  | -0.035 | 0.04 | 0.38 | 1.07E-04 | 49.397 |
| rs10208088 | 2 | 221055873 | T | C | 0.58 | -0.01 | 0.002 | 3.00E-08 |  | 0.012 | 0.027 | 0.646 | 8.50E-05 | 30.685 |
| rs1037091 | 2 | 155652357 | T | C | 0.327 | -0.015 | 0.002 | 5.90E-15 |  | -0.037 | 0.03 | 0.207 | 1.69E-04 | 60.918 |
| rs10518019 | 4 | 67959875 | G | A | 0.476 | 0.01 | 0.002 | 4.90E-08 |  | -0.008 | 0.03 | 0.802 | 8.24E-05 | 29.738 |
| rs10828248 | 10 | 21824619 | G | A | 0.345 | 0.011 | 0.002 | 2.80E-08 |  | -0.02 | 0.029 | 0.497 | 8.55E-05 | 30.846 |
| rs11259902 | 15 | 83886529 | A | C | 0.2 | 0.013 | 0.002 | 1.60E-08 |  | 0.018 | 0.033 | 0.574 | 8.84E-05 | 31.895 |
| rs112600282 | 2 | 156895797 | G | A | 0.11 | -0.017 | 0.003 | 7.20E-09 |  | -0.052 | 0.048 | 0.278 | 9.28E-05 | 33.481 |
| rs113851275 | 9 | 98297220 | A | G | 0.108 | 0.019 | 0.003 | 2.50E-10 |  | -0.005 | 0.053 | 0.919 | 1.11E-04 | 39.994 |
| rs11634155 | 15 | 26693096 | C | T | 0.335 | -0.012 | 0.002 | 2.50E-09 |  | 0.032 | 0.034 | 0.337 | 9.85E-05 | 35.556 |
| rs11652437 | 17 | 79338469 | A | C | 0.324 | 0.013 | 0.002 | 1.00E-10 |  | 0.016 | 0.053 | 0.767 | 1.16E-04 | 41.772 |
| rs11749912 | 5 | 88065628 | G | A | 0.575 | -0.013 | 0.002 | 3.70E-12 |  | -0.059 | 0.027 | 0.029 | 1.34E-04 | 48.256 |
| rs11766392 | 7 | 69838127 | T | G | 0.285 | -0.013 | 0.002 | 3.10E-10 |  | 0.084 | 0.031 | 0.006 | 1.10E-04 | 39.584 |
| rs11942953 | 4 | 163753973 | C | T | 0.537 | -0.01 | 0.002 | 1.30E-08 |  | 0.033 | 0.027 | 0.218 | 8.96E-05 | 32.324 |
| rs12128707 | 1 | 72588119 | G | A | 0.264 | 0.012 | 0.002 | 7.10E-09 |  | -0.002 | 0.032 | 0.94 | 9.29E-05 | 33.517 |
| rs12145677 | 1 | 110023610 | A | G | 0.297 | 0.016 | 0.002 | 6.40E-15 |  | 0.014 | 0.028 | 0.613 | 1.68E-04 | 60.762 |
| rs1229984 | 4 | 100239319 | C | T | 0.973 | -0.034 | 0.006 | 4.40E-10 |  | -0.031 | 0.133 | 0.814 | 1.08E-04 | 38.91 |
| rs12521638 | 5 | 166458770 | G | A | 0.447 | 0.01 | 0.002 | 4.20E-08 |  | -0.029 | 0.027 | 0.283 | 8.33E-05 | 30.056 |
| rs12706626 | 7 | 124531370 | A | G | 0.384 | 0.01 | 0.002 | 2.90E-08 |  | 0.016 | 0.029 | 0.576 | 8.52E-05 | 30.762 |
| rs12820967 | 12 | 38921745 | C | T | 0.322 | 0.012 | 0.002 | 1.50E-09 |  | 0.06 | 0.032 | 0.058 | 1.01E-04 | 36.556 |
| rs13262595 | 8 | 143316970 | G | A | 0.561 | 0.016 | 0.002 | 1.80E-17 |  | -0.055 | 0.027 | 0.04 | 2.00E-04 | 72.37 |
| rs13422733 | 2 | 102010245 | T | C | 0.126 | -0.015 | 0.003 | 4.10E-08 |  | 0.054 | 0.042 | 0.201 | 8.34E-05 | 30.115 |
| rs136553 | 22 | 27255675 | T | C | 0.377 | 0.011 | 0.002 | 2.00E-09 |  | -0.022 | 0.027 | 0.419 | 9.97E-05 | 35.994 |
| rs1395020 | 4 | 139690326 | A | G | 0.303 | -0.011 | 0.002 | 3.40E-08 |  | 0.035 | 0.029 | 0.225 | 8.44E-05 | 30.465 |
| rs1448355 | 11 | 131286685 | T | C | 0.618 | 0.012 | 0.002 | 8.00E-11 |  | 0.004 | 0.028 | 0.89 | 1.17E-04 | 42.262 |
| rs1469249 | 5 | 113837198 | A | G | 0.211 | -0.013 | 0.002 | 8.00E-09 |  | -0.025 | 0.032 | 0.446 | 9.22E-05 | 33.277 |
| rs1648906 | 18 | 35311651 | A | G | 0.321 | -0.011 | 0.002 | 2.10E-08 |  | 0.058 | 0.028 | 0.037 | 8.71E-05 | 31.428 |
| rs166835 | 15 | 47716037 | T | C | 0.556 | -0.011 | 0.002 | 4.30E-09 |  | -0.015 | 0.026 | 0.566 | 9.55E-05 | 34.481 |
| rs16912540 | 11 | 13271422 | G | A | 0.137 | -0.016 | 0.003 | 2.70E-09 |  | 0.075 | 0.041 | 0.07 | 9.80E-05 | 35.363 |
| rs17167210 | 7 | 133339343 | A | G | 0.436 | -0.011 | 0.002 | 6.00E-10 |  | -0.002 | 0.027 | 0.936 | 1.06E-04 | 38.306 |
| rs17789218 | 6 | 100600097 | C | T | 0.245 | 0.013 | 0.002 | 2.10E-09 |  | -0.049 | 0.036 | 0.175 | 9.94E-05 | 35.872 |
| rs17862355 | 7 | 126970135 | G | T | 0.442 | -0.011 | 0.002 | 1.00E-09 |  | 0.006 | 0.027 | 0.831 | 1.03E-04 | 37.243 |
| rs1987942 | 13 | 54004785 | C | T | 0.617 | -0.011 | 0.002 | 5.40E-09 |  | -0.007 | 0.027 | 0.787 | 9.43E-05 | 34.046 |
| rs2032780 | 2 | 215073935 | C | T | 0.406 | 0.012 | 0.002 | 4.50E-11 |  | -0.029 | 0.031 | 0.359 | 1.20E-04 | 43.374 |
| rs2041687 | 2 | 60405620 | G | T | 0.549 | 0.012 | 0.002 | 3.80E-10 |  | -0.022 | 0.033 | 0.497 | 1.09E-04 | 39.23 |
| rs2068625 | 4 | 159856739 | C | T | 0.698 | 0.014 | 0.002 | 1.00E-12 |  | -0.007 | 0.031 | 0.835 | 1.41E-04 | 50.776 |
| rs206965 | 12 | 120856332 | C | T | 0.791 | -0.013 | 0.002 | 7.90E-09 |  | 0.039 | 0.033 | 0.229 | 9.23E-05 | 33.296 |
| rs2120461 | 1 | 8447722 | T | C | 0.661 | 0.012 | 0.002 | 2.50E-10 |  | 0.064 | 0.029 | 0.026 | 1.11E-04 | 40.002 |
| rs246723 | 5 | 140519166 | G | A | 0.59 | -0.011 | 0.002 | 1.20E-08 |  | -0.044 | 0.029 | 0.136 | 9.01E-05 | 32.502 |
| rs2588543 | 4 | 37000406 | T | C | 0.672 | 0.011 | 0.002 | 2.20E-08 |  | -0.016 | 0.028 | 0.562 | 8.67E-05 | 31.287 |
| rs2734833 | 11 | 113292920 | A | G | 0.607 | 0.012 | 0.002 | 3.80E-11 |  | -0.079 | 0.03 | 0.008 | 1.21E-04 | 43.725 |
| rs2748985 | 1 | 1853184 | C | T | 0.544 | 0.012 | 0.002 | 9.40E-12 |  | -0.024 | 0.03 | 0.431 | 1.29E-04 | 46.447 |
| rs2761438 | 1 | 110752139 | G | A | 0.624 | -0.011 | 0.002 | 1.70E-09 |  | -0.011 | 0.032 | 0.74 | 1.00E-04 | 36.273 |
| rs28710456 | 4 | 152667171 | C | T | 0.49 | -0.01 | 0.002 | 1.20E-08 |  | -0.014 | 0.026 | 0.608 | 8.98E-05 | 32.426 |
| rs306755 | 20 | 3099752 | C | T | 0.475 | 0.01 | 0.002 | 1.40E-08 |  | 0.032 | 0.031 | 0.301 | 8.92E-05 | 32.178 |
| rs34238696 | 5 | 161356241 | G | A | 0.107 | -0.017 | 0.003 | 1.30E-08 |  | 0.031 | 0.043 | 0.473 | 8.94E-05 | 32.268 |
| rs3730399 | 16 | 67229019 | G | A | 0.064 | -0.023 | 0.004 | 9.10E-10 |  | 0.053 | 0.052 | 0.309 | 1.04E-04 | 37.507 |
| rs4704043 | 5 | 72159179 | T | C | 0.714 | 0.012 | 0.002 | 8.90E-09 |  | 0.013 | 0.029 | 0.668 | 9.16E-05 | 33.075 |
| rs4852252 | 2 | 71539301 | C | T | 0.564 | 0.01 | 0.002 | 3.00E-08 |  | 0.017 | 0.03 | 0.575 | 8.51E-05 | 30.718 |
| rs56229818 | 15 | 58662232 | C | T | 0.484 | -0.01 | 0.002 | 3.00E-08 |  | 0 | 0.029 | 0.995 | 8.51E-05 | 30.715 |
| rs58638214 | 19 | 31864938 | T | C | 0.398 | -0.013 | 0.002 | 6.70E-12 |  | 0.02 | 0.027 | 0.457 | 1.31E-04 | 47.117 |
| rs6028090 | 20 | 59856465 | A | G | 0.555 | 0.013 | 0.002 | 1.30E-11 |  | 0.025 | 0.029 | 0.385 | 1.27E-04 | 45.87 |
| rs613872 | 18 | 53210302 | T | G | 0.827 | -0.016 | 0.002 | 7.70E-11 |  | -0.031 | 0.035 | 0.367 | 1.17E-04 | 42.329 |
| rs6449708 | 5 | 50851575 | C | T | 0.532 | -0.011 | 0.002 | 7.90E-09 |  | 0.025 | 0.026 | 0.34 | 9.23E-05 | 33.302 |
| rs6780848 | 3 | 8179920 | G | T | 0.27 | 0.011 | 0.002 | 3.30E-08 |  | 0.013 | 0.033 | 0.689 | 8.46E-05 | 30.541 |
| rs6935828 | 6 | 140811367 | T | C | 0.556 | 0.01 | 0.002 | 3.10E-08 |  | -0.039 | 0.027 | 0.141 | 8.49E-05 | 30.65 |
| rs7020477 | 9 | 116827760 | G | A | 0.267 | -0.012 | 0.002 | 7.00E-09 |  | -0.06 | 0.032 | 0.061 | 9.29E-05 | 33.548 |
| rs707926 | 6 | 31748820 | A | G | 0.15 | 0.015 | 0.003 | 1.20E-08 |  | 0.049 | 0.088 | 0.575 | 9.02E-05 | 32.543 |
| rs7209653 | 17 | 19882084 | C | T | 0.295 | -0.013 | 0.002 | 2.20E-11 |  | 0.024 | 0.031 | 0.434 | 1.24E-04 | 44.772 |
| rs7281293 | 21 | 34291496 | C | A | 0.248 | 0.013 | 0.002 | 3.00E-09 |  | -0.029 | 0.03 | 0.348 | 9.75E-05 | 35.189 |
| rs72828532 | 6 | 19065342 | C | T | 0.179 | 0.016 | 0.002 | 4.50E-11 |  | -0.013 | 0.038 | 0.731 | 1.20E-04 | 43.367 |
| rs72847500 | 6 | 37643909 | C | T | 0.121 | 0.016 | 0.003 | 1.90E-08 |  | 0.004 | 0.045 | 0.934 | 8.76E-05 | 31.623 |
| rs7288455 | 22 | 39966547 | G | A | 0.567 | -0.011 | 0.002 | 1.10E-08 |  | -0.019 | 0.031 | 0.545 | 9.05E-05 | 32.647 |
| rs73578186 | 9 | 126334485 | T | C | 0.324 | -0.012 | 0.002 | 3.70E-10 |  | 0.032 | 0.031 | 0.291 | 1.09E-04 | 39.264 |
| rs7526112 | 1 | 93747683 | G | T | 0.362 | -0.011 | 0.002 | 1.10E-08 |  | -0.021 | 0.031 | 0.498 | 9.05E-05 | 32.653 |
| rs75550998 | 2 | 146486095 | T | G | 0.054 | -0.022 | 0.004 | 4.60E-08 |  | -0.204 | 0.082 | 0.012 | 8.28E-05 | 29.893 |
| rs7564844 | 2 | 215335556 | A | G | 0.701 | -0.012 | 0.002 | 5.60E-10 |  | -0.02 | 0.029 | 0.504 | 1.07E-04 | 38.453 |
| rs7630869 | 3 | 49522543 | T | C | 0.304 | 0.016 | 0.002 | 4.00E-16 |  | 0.003 | 0.029 | 0.917 | 1.84E-04 | 66.249 |
| rs76824303 | 3 | 62459819 | C | A | 0.1 | -0.02 | 0.003 | 5.80E-11 |  | -0.037 | 0.05 | 0.453 | 1.19E-04 | 42.891 |
| rs7904398 | 10 | 67954193 | T | C | 0.503 | -0.01 | 0.002 | 2.40E-08 |  | -0.002 | 0.026 | 0.926 | 8.63E-05 | 31.135 |
| rs7968738 | 12 | 90281747 | A | G | 0.264 | -0.012 | 0.002 | 2.40E-09 |  | -0.013 | 0.03 | 0.668 | 9.88E-05 | 35.659 |
| rs79720045 | 4 | 39797668 | C | T | 0.401 | -0.013 | 0.002 | 1.80E-11 |  | 0.016 | 0.038 | 0.676 | 1.25E-04 | 45.23 |
| rs806795 | 6 | 26205293 | A | G | 0.47 | 0.01 | 0.002 | 1.40E-08 |  | 0.007 | 0.03 | 0.815 | 8.93E-05 | 32.23 |
| rs8102851 | 19 | 32208909 | C | T | 0.217 | 0.013 | 0.002 | 7.60E-09 |  | 0.027 | 0.037 | 0.459 | 9.25E-05 | 33.383 |
| rs9375188 | 6 | 98555272 | T | C | 0.484 | 0.016 | 0.002 | 5.00E-18 |  | 0.002 | 0.027 | 0.946 | 2.07E-04 | 74.896 |
| rs9537571 | 13 | 57604700 | A | G | 0.096 | 0.019 | 0.003 | 1.10E-09 |  | 0.011 | 0.048 | 0.825 | 1.03E-04 | 37.22 |
| rs10109061 | 8 | 144239859 | G | A | 0.443 | -0.009 | 0.002 | 3.10E-08 |  | 0.032 | 0.03 | 0.284 | 7.00E-05 | 30.672 |
| rs10189857 | 2 | 60713235 | G | A | 0.432 | 0.015 | 0.002 | 5.70E-20 |  | 0.014 | 0.027 | 0.597 | 1.91E-04 | 83.718 |
| rs10269099 | 7 | 126371011 | T | G | 0.391 | 0.009 | 0.002 | 2.10E-08 |  | 0.045 | 0.027 | 0.095 | 7.17E-05 | 31.412 |
| rs10765777 | 11 | 95656385 | C | A | 0.392 | -0.011 | 0.002 | 4.90E-12 |  | 0.006 | 0.028 | 0.827 | 1.09E-04 | 47.73 |
| rs11191129 | 10 | 103606543 | T | C | 0.423 | -0.01 | 0.002 | 9.30E-10 |  | -0.017 | 0.027 | 0.529 | 8.56E-05 | 37.466 |
| rs11222919 | 11 | 131969663 | G | T | 0.175 | -0.012 | 0.002 | 6.30E-09 |  | 0.026 | 0.035 | 0.465 | 7.71E-05 | 33.75 |
| rs11245482 | 10 | 126733546 | C | T | 0.385 | 0.01 | 0.002 | 4.80E-10 |  | 0.014 | 0.027 | 0.617 | 8.85E-05 | 38.772 |
| rs114755463 | 5 | 152503110 | A | G | 0.168 | 0.013 | 0.002 | 6.70E-10 |  | -0.059 | 0.038 | 0.122 | 8.70E-05 | 38.096 |
| rs115608101 | 11 | 107106532 | T | C | 0.13 | -0.014 | 0.002 | 1.10E-08 |  | 0.05 | 0.044 | 0.259 | 7.44E-05 | 32.599 |
| rs11662211 | 18 | 77618869 | T | C | 0.506 | -0.009 | 0.002 | 2.90E-09 |  | -0.002 | 0.03 | 0.953 | 8.05E-05 | 35.242 |
| rs11680095 | 2 | 181825956 | T | C | 0.593 | -0.009 | 0.002 | 1.90E-08 |  | -0.045 | 0.028 | 0.101 | 7.22E-05 | 31.619 |
| rs11696187 | 20 | 58891882 | T | C | 0.16 | -0.014 | 0.002 | 4.20E-10 |  | 0.002 | 0.037 | 0.962 | 8.91E-05 | 39.029 |
| rs11700249 | 20 | 11910800 | G | T | 0.409 | 0.009 | 0.002 | 5.90E-09 |  | 0.018 | 0.028 | 0.509 | 7.73E-05 | 33.86 |
| rs11714337 | 3 | 71582521 | A | G | 0.43 | -0.01 | 0.002 | 7.60E-10 |  | -0.041 | 0.03 | 0.167 | 8.64E-05 | 37.855 |
| rs11877758 | 18 | 35138110 | G | T | 0.313 | 0.011 | 0.002 | 1.30E-10 |  | 0.041 | 0.028 | 0.149 | 9.43E-05 | 41.285 |
| rs11911112 | 21 | 40528346 | C | A | 0.366 | -0.011 | 0.002 | 8.00E-11 |  | 0.016 | 0.028 | 0.563 | 9.65E-05 | 42.247 |
| rs12045585 | 1 | 243673099 | A | G | 0.131 | -0.015 | 0.002 | 4.10E-10 |  | 0.063 | 0.04 | 0.119 | 8.92E-05 | 39.068 |
| rs12214364 | 6 | 67556372 | G | T | 0.415 | 0.009 | 0.002 | 3.80E-08 |  | -0.06 | 0.035 | 0.089 | 6.90E-05 | 30.234 |
| rs1291871 | 10 | 11086083 | C | T | 0.514 | 0.009 | 0.002 | 9.90E-09 |  | 0.033 | 0.029 | 0.249 | 7.50E-05 | 32.862 |
| rs13014947 | 2 | 193742999 | A | G | 0.575 | 0.011 | 0.002 | 6.50E-11 |  | 0.026 | 0.028 | 0.353 | 9.74E-05 | 42.663 |
| rs13107325 | 4 | 103188709 | T | C | 0.075 | 0.02 | 0.003 | 1.70E-11 |  | 0.065 | 0.072 | 0.366 | 1.04E-04 | 45.326 |
| rs1324491 | 1 | 60350616 | A | G | 0.131 | 0.013 | 0.002 | 1.30E-08 |  | 0.029 | 0.041 | 0.482 | 7.39E-05 | 32.37 |
| rs1727332 | 12 | 123718301 | T | C | 0.754 | 0.013 | 0.002 | 1.10E-12 |  | 0.022 | 0.034 | 0.528 | 1.16E-04 | 50.601 |
| rs17789218 | 6 | 100600097 | C | T | 0.244 | -0.011 | 0.002 | 3.60E-09 |  | -0.049 | 0.036 | 0.175 | 7.96E-05 | 34.845 |
| rs178203 | 14 | 26959322 | C | T | 0.75 | 0.012 | 0.002 | 5.90E-11 |  | 0.019 | 0.035 | 0.577 | 9.78E-05 | 42.845 |
| rs180396 | 13 | 60437497 | T | C | 0.747 | 0.01 | 0.002 | 4.30E-08 |  | 0.006 | 0.031 | 0.846 | 6.85E-05 | 30.007 |
| rs1826510 | 5 | 24800012 | A | G | 0.828 | -0.013 | 0.002 | 1.90E-08 |  | -0.102 | 0.058 | 0.076 | 7.22E-05 | 31.621 |
| rs1889996 | 13 | 54269950 | G | T | 0.739 | 0.012 | 0.002 | 3.90E-11 |  | 0.034 | 0.03 | 0.261 | 9.97E-05 | 43.664 |
| rs1993092 | 6 | 98689604 | C | T | 0.39 | -0.01 | 0.002 | 5.40E-09 |  | 0.001 | 0.027 | 0.959 | 7.78E-05 | 34.051 |
| rs2073869 | 9 | 135763816 | T | C | 0.167 | -0.014 | 0.002 | 1.10E-10 |  | -0.006 | 0.036 | 0.875 | 9.50E-05 | 41.61 |
| rs2106164 | 7 | 92661753 | C | T | 0.532 | -0.009 | 0.002 | 4.20E-09 |  | 0.038 | 0.03 | 0.203 | 7.88E-05 | 34.513 |
| rs2185490 | 14 | 69732119 | C | A | 0.618 | 0.009 | 0.002 | 1.20E-08 |  | -0.01 | 0.03 | 0.734 | 7.41E-05 | 32.471 |
| rs2240857 | 7 | 8010634 | G | T | 0.141 | 0.016 | 0.002 | 4.50E-12 |  | 0.028 | 0.039 | 0.472 | 1.09E-04 | 47.89 |
| rs2283 | 5 | 106773623 | G | A | 0.339 | -0.009 | 0.002 | 3.20E-08 |  | 0 | 0.028 | 0.993 | 6.99E-05 | 30.592 |
| rs2332818 | 14 | 27112432 | C | T | 0.731 | 0.01 | 0.002 | 4.00E-08 |  | 0.014 | 0.097 | 0.884 | 6.89E-05 | 30.172 |
| rs2352984 | 3 | 49948728 | C | T | 0.431 | 0.02 | 0.002 | 7.40E-35 |  | 0.001 | 0.029 | 0.974 | 3.46E-04 | 151.702 |
| rs2479968 | 13 | 111969328 | G | A | 0.051 | 0.021 | 0.004 | 2.70E-08 |  | 0.137 | 0.093 | 0.141 | 7.06E-05 | 30.922 |
| rs249960 | 5 | 96164771 | G | A | 0.182 | -0.012 | 0.002 | 1.10E-08 |  | 0.045 | 0.034 | 0.19 | 7.45E-05 | 32.633 |
| rs262890 | 5 | 62930015 | G | A | 0.299 | 0.013 | 0.002 | 2.60E-14 |  | -0.005 | 0.03 | 0.866 | 1.33E-04 | 58.042 |
| rs263771 | 2 | 185921692 | A | C | 0.233 | 0.012 | 0.002 | 1.90E-10 |  | 0.016 | 0.034 | 0.642 | 9.27E-05 | 40.602 |
| rs2646351 | 4 | 55701312 | A | G | 0.453 | 0.009 | 0.002 | 2.40E-08 |  | 0.021 | 0.026 | 0.428 | 7.10E-05 | 31.101 |
| rs2678662 | 2 | 104446759 | G | T | 0.608 | 0.011 | 0.002 | 8.60E-12 |  | -0.052 | 0.027 | 0.053 | 1.06E-04 | 46.621 |
| rs2725371 | 8 | 30854033 | G | A | 0.696 | -0.013 | 0.002 | 2.10E-14 |  | 0.005 | 0.029 | 0.875 | 1.34E-04 | 58.479 |
| rs2857693 | 6 | 31588384 | T | G | 0.366 | 0.011 | 0.002 | 9.60E-11 |  | -0.052 | 0.064 | 0.417 | 9.57E-05 | 41.906 |
| rs2906604 | 2 | 107624244 | C | T | 0.497 | 0.01 | 0.002 | 5.50E-11 |  | 0.016 | 0.026 | 0.542 | 9.82E-05 | 42.994 |
| rs3138499 | 9 | 92219921 | C | A | 0.518 | 0.011 | 0.002 | 8.50E-12 |  | -0.037 | 0.026 | 0.16 | 1.07E-04 | 46.658 |
| rs34094119 | 8 | 10935898 | G | A | 0.525 | 0.009 | 0.002 | 1.30E-08 |  | 0.037 | 0.031 | 0.232 | 7.39E-05 | 32.382 |
| rs34811474 | 4 | 25408838 | A | G | 0.231 | -0.013 | 0.002 | 3.10E-12 |  | 0.01 | 0.053 | 0.854 | 1.11E-04 | 48.598 |
| rs35797019 | 3 | 93987306 | G | A | 0.392 | -0.009 | 0.002 | 1.50E-08 |  | 0.014 | 0.027 | 0.618 | 7.32E-05 | 32.04 |
| rs362312 | 4 | 3237644 | C | T | 0.424 | -0.01 | 0.002 | 3.50E-09 |  | -0.04 | 0.028 | 0.151 | 7.97E-05 | 34.892 |
| rs3754970 | 2 | 162091836 | C | T | 0.503 | 0.01 | 0.002 | 1.30E-09 |  | 0.064 | 0.027 | 0.016 | 8.41E-05 | 36.828 |
| rs3810496 | 20 | 62406886 | C | T | 0.616 | 0.009 | 0.002 | 1.20E-08 |  | 0.019 | 0.029 | 0.524 | 7.42E-05 | 32.482 |
| rs4076457 | 15 | 78007213 | T | C | 0.257 | -0.01 | 0.002 | 3.70E-08 |  | -0.026 | 0.031 | 0.41 | 6.92E-05 | 30.298 |
| rs4110177 | 5 | 88793281 | A | G | 0.367 | 0.009 | 0.002 | 1.30E-08 |  | 0.025 | 0.027 | 0.364 | 7.39E-05 | 32.375 |
| rs4303732 | 2 | 100830040 | C | T | 0.402 | -0.011 | 0.002 | 3.70E-11 |  | -0.025 | 0.027 | 0.359 | 1.00E-04 | 43.779 |
| rs4339469 | 6 | 98369230 | G | T | 0.629 | 0.013 | 0.002 | 2.80E-14 |  | -0.01 | 0.028 | 0.711 | 1.32E-04 | 57.861 |
| rs4469687 | 1 | 184679019 | G | A | 0.484 | 0.009 | 0.002 | 3.10E-08 |  | 0.027 | 0.026 | 0.305 | 6.99E-05 | 30.621 |
| rs4567133 | 9 | 22606560 | A | C | 0.81 | -0.013 | 0.002 | 1.70E-10 |  | -0.017 | 0.034 | 0.628 | 9.32E-05 | 40.821 |
| rs4747438 | 10 | 22124263 | T | C | 0.677 | -0.012 | 0.002 | 3.90E-12 |  | -0.029 | 0.033 | 0.384 | 1.10E-04 | 48.152 |
| rs4788616 | 16 | 72211984 | G | T | 0.391 | -0.01 | 0.002 | 4.30E-10 |  | -0.006 | 0.027 | 0.833 | 8.90E-05 | 38.982 |
| rs494566 | 9 | 1785717 | T | C | 0.332 | 0.01 | 0.002 | 2.00E-09 |  | 0.006 | 0.032 | 0.854 | 8.20E-05 | 35.93 |
| rs57555420 | 1 | 97783448 | T | C | 0.282 | 0.01 | 0.002 | 9.40E-09 |  | -0.007 | 0.032 | 0.827 | 7.53E-05 | 32.957 |
| rs58541850 | 6 | 166165563 | A | G | 0.059 | 0.021 | 0.003 | 2.70E-10 |  | -0.038 | 0.074 | 0.612 | 9.10E-05 | 39.872 |
| rs6102912 | 20 | 41202935 | C | T | 0.41 | -0.011 | 0.002 | 1.40E-11 |  | 0.047 | 0.026 | 0.076 | 1.04E-04 | 45.603 |
| rs6125907 | 20 | 48730315 | A | C | 0.092 | 0.016 | 0.003 | 4.50E-09 |  | 0.042 | 0.05 | 0.396 | 7.85E-05 | 34.381 |
| rs61743199 | 19 | 50161091 | G | A | 0.072 | 0.017 | 0.003 | 1.60E-08 |  | -0.078 | 0.114 | 0.493 | 7.28E-05 | 31.874 |
| rs61864793 | 10 | 85803372 | C | T | 0.252 | -0.011 | 0.002 | 1.20E-09 |  | 0.005 | 0.03 | 0.861 | 8.42E-05 | 36.894 |
| rs62145951 | 2 | 68399586 | C | T | 0.263 | -0.012 | 0.002 | 2.60E-11 |  | 0.004 | 0.032 | 0.892 | 1.01E-04 | 44.433 |
| rs62199883 | 2 | 215376706 | A | C | 0.486 | 0.014 | 0.002 | 3.20E-18 |  | 0.013 | 0.029 | 0.655 | 1.73E-04 | 75.768 |
| rs6511708 | 19 | 10788813 | C | T | 0.665 | -0.012 | 0.002 | 7.50E-13 |  | 0.028 | 0.028 | 0.314 | 1.17E-04 | 51.41 |
| rs68056254 | 2 | 147846855 | T | G | 0.151 | 0.013 | 0.002 | 1.30E-09 |  | -0.034 | 0.04 | 0.402 | 8.40E-05 | 36.792 |
| rs6814554 | 4 | 152454334 | A | G | 0.474 | 0.014 | 0.002 | 2.40E-17 |  | -0.028 | 0.027 | 0.293 | 1.64E-04 | 71.822 |
| rs6850494 | 4 | 82291771 | C | A | 0.386 | 0.01 | 0.002 | 3.40E-09 |  | -0.025 | 0.028 | 0.375 | 7.98E-05 | 34.938 |
| rs6895658 | 5 | 124274035 | C | T | 0.193 | -0.013 | 0.002 | 1.10E-10 |  | -0.026 | 0.037 | 0.493 | 9.50E-05 | 41.583 |
| rs6994132 | 8 | 92653740 | C | T | 0.579 | -0.01 | 0.002 | 6.20E-10 |  | -0.051 | 0.03 | 0.094 | 8.73E-05 | 38.25 |
| rs7089973 | 10 | 116569565 | A | C | 0.38 | 0.009 | 0.002 | 1.60E-08 |  | -0.012 | 0.028 | 0.661 | 7.28E-05 | 31.892 |
| rs7184800 | 16 | 53509131 | A | G | 0.303 | -0.013 | 0.002 | 7.40E-15 |  | -0.03 | 0.029 | 0.301 | 1.38E-04 | 60.476 |
| rs73571431 | 9 | 126136139 | T | C | 0.11 | 0.016 | 0.003 | 5.50E-10 |  | -0.01 | 0.048 | 0.837 | 8.79E-05 | 38.495 |
| rs749056 | 1 | 110037838 | G | T | 0.304 | -0.01 | 0.002 | 7.20E-09 |  | 0.023 | 0.028 | 0.426 | 7.65E-05 | 33.481 |
| rs749671 | 16 | 31088347 | A | G | 0.372 | -0.011 | 0.002 | 1.60E-11 |  | -0.003 | 0.028 | 0.922 | 1.04E-04 | 45.388 |
| rs7539775 | 1 | 3109151 | A | G | 0.743 | 0.01 | 0.002 | 4.30E-08 |  | 0.001 | 0.033 | 0.985 | 6.85E-05 | 30.005 |
| rs75499503 | 6 | 26145217 | T | C | 0.22 | -0.018 | 0.002 | 3.10E-21 |  | 0.028 | 0.04 | 0.487 | 2.04E-04 | 89.463 |
| rs75641275 | 1 | 98327133 | C | A | 0.143 | 0.015 | 0.002 | 1.00E-11 |  | -0.04 | 0.042 | 0.339 | 1.06E-04 | 46.239 |
| rs7708324 | 5 | 147920094 | G | A | 0.377 | -0.01 | 0.002 | 1.60E-09 |  | -0.029 | 0.027 | 0.276 | 8.31E-05 | 36.404 |
| rs7798292 | 7 | 112974602 | A | G | 0.435 | -0.01 | 0.002 | 1.30E-09 |  | -0.001 | 0.026 | 0.962 | 8.40E-05 | 36.764 |
| rs7899206 | 10 | 127188859 | G | T | 0.492 | -0.01 | 0.002 | 1.30E-09 |  | 0.021 | 0.032 | 0.502 | 8.42E-05 | 36.852 |
| rs7921305 | 10 | 133775196 | A | G | 0.253 | -0.011 | 0.002 | 3.70E-10 |  | -0.043 | 0.036 | 0.228 | 8.96E-05 | 39.257 |
| rs801733 | 11 | 65934549 | C | A | 0.358 | -0.012 | 0.002 | 5.00E-14 |  | -0.013 | 0.028 | 0.634 | 1.30E-04 | 56.723 |
| rs814197 | 1 | 61092456 | G | T | 0.467 | -0.011 | 0.002 | 2.00E-11 |  | 0.012 | 0.026 | 0.645 | 1.03E-04 | 44.948 |
| rs898751 | 17 | 2291863 | T | C | 0.493 | 0.01 | 0.002 | 3.00E-10 |  | -0.041 | 0.028 | 0.14 | 9.06E-05 | 39.664 |
| rs9300594 | 13 | 100869905 | G | A | 0.254 | 0.011 | 0.002 | 9.10E-10 |  | 0.002 | 0.031 | 0.945 | 8.56E-05 | 37.504 |
| rs9471333 | 6 | 40362023 | T | C | 0.552 | -0.01 | 0.002 | 6.70E-11 |  | -0.033 | 0.026 | 0.21 | 9.73E-05 | 42.592 |
| rs9834970 | 3 | 36856030 | C | T | 0.498 | -0.009 | 0.002 | 2.00E-08 |  | 0.033 | 0.027 | 0.217 | 7.19E-05 | 31.491 |
| rs9867121 | 3 | 114631548 | A | C | 0.184 | -0.012 | 0.002 | 1.40E-08 |  | 0.017 | 0.036 | 0.644 | 7.36E-05 | 32.239 |
| rs9867437 | 3 | 85676752 | C | A | 0.46 | -0.01 | 0.002 | 1.30E-10 |  | -0.01 | 0.027 | 0.71 | 9.45E-05 | 41.366 |
| rs9880023 | 3 | 54178199 | T | G | 0.556 | 0.01 | 0.002 | 2.00E-09 |  | 0.038 | 0.029 | 0.185 | 8.22E-05 | 35.994 |
| rs996234 | 5 | 59455212 | A | G | 0.516 | -0.011 | 0.002 | 1.60E-10 |  | -0.02 | 0.029 | 0.486 | 9.34E-05 | 40.916 |

**Note:** Abbreviations: Beta, effect allele value; SE, standard error; SNP, single nucleotide polymorphism

**Table S4.** Genetic instruments for autism spectrum disorder

| **Exposure** | **SNP** | **Chromosome** | **Position** | **Effect allele** | **Other allele** | **Effect allele frequency** | **Exposure** | | |  | **Outcome** | | | ***R^2^*** | ***F-Statistic*** |
| --- | --- | --- | --- | --- | --- | --- | --- | --- | --- | --- | --- | --- | --- | --- | --- |
|  |  |  |  |  |  |  | ***Beta*** | ***SE*** | ***P-value*** |  | ***Beta*** | ***SE*** | ***P-value*** |  |  |
| Length of mobile phone use | rs10807124 | 6 | 33404064 | A | G | 0.274 | -0.018 | 0.003 | 3.10E-08 |  | 0.022 | 0.015 | 0.135 | 6.70E-05 | 30.623 |
| Length of mobile phone use | rs10828247 | 10 | 21822856 | G | A | 0.344 | 0.017 | 0.003 | 7.40E-09 |  | -0.023 | 0.015 | 0.115 | 7.31E-05 | 33.426 |
| Length of mobile phone use | rs11229008 | 11 | 57118840 | A | G | 0.063 | -0.033 | 0.006 | 3.90E-08 |  | -0.064 | 0.031 | 0.04 | 6.61E-05 | 30.222 |
| Length of mobile phone use | rs11236714 | 11 | 70424559 | T | C | 0.196 | -0.02 | 0.004 | 1.80E-08 |  | -0.003 | 0.018 | 0.882 | 6.93E-05 | 31.661 |
| Length of mobile phone use | rs11655813 | 17 | 2119101 | T | C | 0.355 | 0.018 | 0.003 | 1.20E-09 |  | -0.008 | 0.014 | 0.582 | 8.10E-05 | 37.04 |
| Length of mobile phone use | rs11682846 | 2 | 157008703 | T | C | 0.485 | -0.017 | 0.003 | 9.90E-10 |  | 0.008 | 0.014 | 0.565 | 8.17E-05 | 37.352 |
| Length of mobile phone use | rs12145998 | 1 | 204969419 | T | C | 0.266 | -0.019 | 0.003 | 2.90E-09 |  | -0.005 | 0.016 | 0.729 | 7.71E-05 | 35.224 |
| Length of mobile phone use | rs12437348 | 14 | 36606550 | A | G | 0.71 | 0.017 | 0.003 | 4.20E-08 |  | 0.012 | 0.015 | 0.423 | 6.57E-05 | 30.033 |
| Length of mobile phone use | rs13266457 | 8 | 106086786 | T | C | 0.33 | -0.017 | 0.003 | 1.70E-08 |  | 0.019 | 0.015 | 0.196 | 6.97E-05 | 31.856 |
| Length of mobile phone use | rs1512142 | 4 | 47004305 | A | G | 0.443 | -0.017 | 0.003 | 8.30E-09 |  | 0.004 | 0.014 | 0.786 | 7.27E-05 | 33.205 |
| Length of mobile phone use | rs17156711 | 5 | 103924699 | G | A | 0.299 | -0.018 | 0.003 | 4.10E-09 |  | -0.027 | 0.015 | 0.074 | 7.56E-05 | 34.563 |
| Length of mobile phone use | rs17374152 | 5 | 93236948 | G | A | 0.239 | -0.019 | 0.003 | 5.30E-09 |  | 0.015 | 0.016 | 0.365 | 7.46E-05 | 34.088 |
| Length of mobile phone use | rs1892417 | 1 | 41779673 | C | T | 0.229 | 0.026 | 0.003 | 1.30E-14 |  | -0.034 | 0.016 | 0.037 | 1.30E-04 | 59.328 |
| Length of mobile phone use | rs2161220 | 5 | 80241904 | A | G | 0.248 | 0.021 | 0.003 | 4.00E-10 |  | -0.035 | 0.016 | 0.028 | 8.56E-05 | 39.118 |
| Length of mobile phone use | rs2836920 | 21 | 40512918 | G | T | 0.396 | 0.019 | 0.003 | 2.30E-10 |  | 0.03 | 0.015 | 0.042 | 8.80E-05 | 40.218 |
| Length of mobile phone use | rs28713780 | 7 | 3321415 | C | T | 0.641 | -0.017 | 0.003 | 1.10E-08 |  | -0.009 | 0.014 | 0.511 | 7.15E-05 | 32.685 |
| Length of mobile phone use | rs344868 | 2 | 140095800 | T | C | 0.247 | 0.018 | 0.003 | 3.20E-08 |  | 0.002 | 0.016 | 0.898 | 6.69E-05 | 30.553 |
| Length of mobile phone use | rs359265 | 2 | 60456410 | A | G | 0.607 | 0.021 | 0.003 | 6.40E-13 |  | 0.008 | 0.014 | 0.59 | 1.13E-04 | 51.715 |
| Length of mobile phone use | rs6063374 | 20 | 47832997 | G | A | 0.781 | 0.029 | 0.003 | 1.00E-17 |  | -0.007 | 0.016 | 0.661 | 1.61E-04 | 73.426 |
| Length of mobile phone use | rs6131703 | 20 | 15754684 | G | A | 0.386 | -0.018 | 0.003 | 1.90E-09 |  | 0.009 | 0.014 | 0.539 | 7.90E-05 | 36.121 |
| Length of mobile phone use | rs6780051 | 3 | 56193150 | T | G | 0.058 | 0.04 | 0.006 | 6.90E-11 |  | 0.001 | 0.03 | 0.985 | 9.31E-05 | 42.548 |
| Length of mobile phone use | rs78166132 | 5 | 161239986 | C | T | 0.094 | -0.031 | 0.005 | 4.90E-10 |  | 0.016 | 0.023 | 0.474 | 8.47E-05 | 38.728 |
| Length of mobile phone use | rs7859831 | 9 | 126205754 | T | C | 0.139 | -0.023 | 0.004 | 2.10E-08 |  | -0.023 | 0.02 | 0.252 | 6.87E-05 | 31.393 |
| Length of mobile phone use | rs8014346 | 14 | 46832002 | A | G | 0.535 | 0.019 | 0.003 | 3.70E-11 |  | 0.007 | 0.014 | 0.592 | 9.57E-05 | 43.758 |
| Length of mobile phone use | rs849527 | 2 | 206592525 | G | A | 0.547 | -0.016 | 0.003 | 1.50E-08 |  | 0.016 | 0.014 | 0.255 | 7.00E-05 | 31.995 |
| Length of mobile phone use | rs853946 | 10 | 120166472 | T | C | 0.468 | 0.016 | 0.003 | 1.80E-08 |  | -0.029 | 0.014 | 0.04 | 6.94E-05 | 31.707 |
| Length of mobile phone use | rs9896202 | 17 | 77778227 | C | T | 0.498 | -0.021 | 0.003 | 1.90E-13 |  | 0.001 | 0.014 | 0.974 | 1.18E-04 | 54.134 |
| Plays computer games | rs10170573 | 2 | 225552916 | G | A | 0.654 | -0.006 | 0.001 | 4.00E-08 |  | -0.01 | 0.015 | 0.485 | 6.52E-05 | 30.147 |
| Plays computer games | rs10799961 | 1 | 163744838 | G | A | 0.538 | 0.006 | 0.001 | 3.50E-08 |  | -0.039 | 0.014 | 0.005 | 6.57E-05 | 30.388 |
| Plays computer games | rs10803798 | 2 | 166399307 | G | T | 0.284 | 0.008 | 0.001 | 7.80E-12 |  | -0.029 | 0.016 | 0.066 | 1.01E-04 | 46.811 |
| Plays computer games | rs11223780 | 11 | 134261150 | G | A | 0.148 | 0.008 | 0.001 | 9.50E-09 |  | 0.001 | 0.02 | 0.978 | 7.12E-05 | 32.946 |
| Plays computer games | rs113002196 | 1 | 27200156 | C | T | 0.16 | 0.008 | 0.001 | 7.50E-09 |  | -0.008 | 0.019 | 0.664 | 7.22E-05 | 33.411 |
| Plays computer games | rs11548535 | 7 | 148936878 | T | G | 0.146 | -0.008 | 0.001 | 4.50E-08 |  | 0.013 | 0.019 | 0.483 | 6.47E-05 | 29.912 |
| Plays computer games | rs11740196 | 5 | 12855333 | G | A | 0.484 | -0.007 | 0.001 | 2.60E-11 |  | -0.017 | 0.014 | 0.233 | 9.61E-05 | 44.46 |
| Plays computer games | rs11743441 | 5 | 88065637 | T | G | 0.574 | -0.007 | 0.001 | 3.60E-11 |  | -0.011 | 0.014 | 0.432 | 9.47E-05 | 43.817 |
| Plays computer games | rs12129719 | 1 | 66324512 | A | G | 0.552 | 0.006 | 0.001 | 1.20E-09 |  | 0.026 | 0.014 | 0.069 | 8.00E-05 | 37 |
| Plays computer games | rs12138787 | 1 | 150246070 | C | T | 0.124 | 0.009 | 0.002 | 1.10E-08 |  | 0.041 | 0.021 | 0.051 | 7.05E-05 | 32.605 |
| Plays computer games | rs1232205 | 11 | 31661314 | C | T | 0.302 | -0.007 | 0.001 | 4.10E-09 |  | -0.006 | 0.015 | 0.701 | 7.48E-05 | 34.595 |
| Plays computer games | rs12361956 | 11 | 40406823 | C | T | 0.39 | -0.006 | 0.001 | 4.10E-08 |  | -0.005 | 0.014 | 0.724 | 6.51E-05 | 30.107 |
| Plays computer games | rs12707117 | 7 | 133429058 | A | G | 0.555 | 0.007 | 0.001 | 3.00E-10 |  | 0.014 | 0.014 | 0.312 | 8.58E-05 | 39.698 |
| Plays computer games | rs13262595 | 8 | 143316970 | G | A | 0.561 | 0.008 | 0.001 | 6.30E-14 |  | -0.008 | 0.014 | 0.587 | 1.22E-04 | 56.287 |
| Plays computer games | rs144377835 | 3 | 70581216 | T | C | 0.039 | 0.018 | 0.003 | 6.90E-11 |  | 0.018 | 0.04 | 0.653 | 9.20E-05 | 42.556 |
| Plays computer games | rs16831510 | 2 | 200305460 | T | G | 0.128 | 0.01 | 0.002 | 3.90E-11 |  | 0.02 | 0.021 | 0.342 | 9.44E-05 | 43.674 |
| Plays computer games | rs17057709 | 18 | 73378941 | T | C | 0.142 | -0.008 | 0.001 | 2.40E-08 |  | -0.038 | 0.022 | 0.08 | 6.73E-05 | 31.103 |
| Plays computer games | rs2014653 | 20 | 50788476 | G | A | 0.298 | -0.008 | 0.001 | 3.70E-11 |  | -0.018 | 0.016 | 0.25 | 9.47E-05 | 43.774 |
| Plays computer games | rs302719 | 1 | 8490320 | G | T | 0.34 | -0.006 | 0.001 | 6.00E-09 |  | 0.012 | 0.015 | 0.396 | 7.32E-05 | 33.848 |
| Plays computer games | rs34217929 | 2 | 137485074 | A | G | 0.256 | 0.01 | 0.001 | 9.70E-16 |  | -0.009 | 0.016 | 0.565 | 1.39E-04 | 64.484 |
| Plays computer games | rs34402857 | 2 | 156109104 | A | G | 0.519 | 0.008 | 0.001 | 7.90E-14 |  | 0.027 | 0.014 | 0.048 | 1.21E-04 | 55.833 |
| Plays computer games | rs35104374 | 12 | 6739497 | C | T | 0.725 | -0.007 | 0.001 | 1.90E-08 |  | -0.003 | 0.017 | 0.84 | 6.82E-05 | 31.559 |
| Plays computer games | rs3757589 | 7 | 73865349 | A | G | 0.389 | -0.006 | 0.001 | 1.80E-08 |  | -0.002 | 0.016 | 0.89 | 6.85E-05 | 31.691 |
| Plays computer games | rs4113587 | 6 | 128311640 | G | T | 0.443 | 0.007 | 0.001 | 2.40E-11 |  | 0.011 | 0.014 | 0.411 | 9.65E-05 | 44.651 |
| Plays computer games | rs411844 | 7 | 101692403 | A | G | 0.821 | -0.007 | 0.001 | 4.30E-08 |  | 0.016 | 0.018 | 0.396 | 6.49E-05 | 29.999 |
| Plays computer games | rs4938017 | 11 | 113303929 | T | C | 0.393 | -0.01 | 0.001 | 2.00E-19 |  | -0.018 | 0.014 | 0.21 | 1.76E-04 | 81.22 |
| Plays computer games | rs535591 | 3 | 83154118 | A | G | 0.788 | -0.007 | 0.001 | 3.80E-08 |  | -0.007 | 0.017 | 0.685 | 6.54E-05 | 30.236 |
| Plays computer games | rs56275045 | 16 | 10201392 | A | C | 0.137 | 0.009 | 0.002 | 7.40E-09 |  | -0.012 | 0.021 | 0.551 | 7.23E-05 | 33.437 |
| Plays computer games | rs580241 | 11 | 66066349 | A | G | 0.763 | -0.007 | 0.001 | 5.90E-09 |  | 0.026 | 0.017 | 0.123 | 7.32E-05 | 33.866 |
| Plays computer games | rs62241000 | 22 | 42378507 | T | C | 0.321 | 0.006 | 0.001 | 2.90E-08 |  | -0.036 | 0.015 | 0.016 | 6.66E-05 | 30.788 |
| Plays computer games | rs623863 | 11 | 88608915 | G | A | 0.651 | 0.006 | 0.001 | 5.90E-09 |  | 0.013 | 0.015 | 0.369 | 7.32E-05 | 33.854 |
| Plays computer games | rs6457740 | 6 | 33697125 | G | A | 0.703 | -0.007 | 0.001 | 1.40E-09 |  | 0.004 | 0.016 | 0.791 | 7.93E-05 | 36.664 |
| Plays computer games | rs6536378 | 4 | 159857227 | G | T | 0.653 | 0.006 | 0.001 | 3.70E-08 |  | -0.031 | 0.015 | 0.036 | 6.55E-05 | 30.279 |
| Plays computer games | rs678007 | 1 | 23600579 | T | C | 0.178 | -0.008 | 0.001 | 3.40E-08 |  | -0.039 | 0.018 | 0.028 | 6.59E-05 | 30.473 |
| Plays computer games | rs717915 | 2 | 200714766 | T | C | 0.635 | 0.006 | 0.001 | 3.50E-08 |  | 0.015 | 0.014 | 0.296 | 6.58E-05 | 30.417 |
| Plays computer games | rs7196161 | 16 | 31110981 | A | G | 0.633 | 0.008 | 0.001 | 1.90E-12 |  | -0.011 | 0.014 | 0.449 | 1.07E-04 | 49.586 |
| Plays computer games | rs7234933 | 18 | 39692681 | C | T | 0.101 | 0.01 | 0.002 | 4.00E-08 |  | 0.016 | 0.023 | 0.496 | 6.52E-05 | 30.13 |
| Plays computer games | rs7431945 | 3 | 44029095 | A | G | 0.232 | 0.008 | 0.001 | 2.70E-10 |  | 0.027 | 0.017 | 0.108 | 8.63E-05 | 39.913 |
| Plays computer games | rs76563988 | 15 | 47947718 | G | A | 0.248 | -0.007 | 0.001 | 4.50E-09 |  | 0.005 | 0.016 | 0.774 | 7.44E-05 | 34.388 |
| Plays computer games | rs78081759 | 19 | 51130909 | A | G | 0.066 | 0.012 | 0.002 | 4.00E-09 |  | 0.039 | 0.028 | 0.159 | 7.49E-05 | 34.616 |
| Plays computer games | rs7824395 | 8 | 118922172 | G | A | 0.315 | -0.007 | 0.001 | 2.60E-09 |  | 0.025 | 0.015 | 0.088 | 7.66E-05 | 35.445 |
| Plays computer games | rs7907706 | 10 | 128491019 | A | G | 0.764 | 0.007 | 0.001 | 4.50E-08 |  | -0.012 | 0.017 | 0.454 | 6.47E-05 | 29.922 |
| Plays computer games | rs8062624 | 16 | 10168497 | C | T | 0.114 | 0.011 | 0.002 | 8.00E-11 |  | -0.036 | 0.022 | 0.105 | 9.14E-05 | 42.261 |
| Plays computer games | rs9444043 | 6 | 83915414 | C | A | 0.376 | -0.006 | 0.001 | 1.90E-09 |  | 0.033 | 0.014 | 0.022 | 7.80E-05 | 36.075 |
| Plays computer games | rs9661614 | 1 | 110021837 | C | T | 0.297 | 0.007 | 0.001 | 2.50E-10 |  | 0.004 | 0.015 | 0.793 | 8.66E-05 | 40.028 |
| Plays computer games | rs9688977 | 6 | 154336892 | C | T | 0.145 | 0.009 | 0.001 | 4.50E-09 |  | 0.02 | 0.019 | 0.288 | 7.43E-05 | 34.377 |
| Plays computer games | rs9842435 | 3 | 117517339 | G | A | 0.876 | -0.011 | 0.002 | 2.10E-12 |  | 0.014 | 0.021 | 0.504 | 1.07E-04 | 49.397 |
| Time spent using computer | rs10208088 | 2 | 221055873 | T | C | 0.58 | -0.01 | 0.002 | 3.00E-08 |  | -0.004 | 0.014 | 0.783 | 8.50E-05 | 30.685 |
| Time spent using computer | rs1037091 | 2 | 155652357 | T | C | 0.327 | -0.015 | 0.002 | 5.90E-15 |  | -0.015 | 0.015 | 0.319 | 1.69E-04 | 60.918 |
| Time spent using computer | rs10518019 | 4 | 67959875 | G | A | 0.476 | 0.01 | 0.002 | 4.90E-08 |  | 0 | 0.014 | 0.992 | 8.24E-05 | 29.738 |
| Time spent using computer | rs10828248 | 10 | 21824619 | G | A | 0.345 | 0.011 | 0.002 | 2.80E-08 |  | -0.022 | 0.014 | 0.121 | 8.55E-05 | 30.846 |
| Time spent using computer | rs11259902 | 15 | 83886529 | A | C | 0.2 | 0.013 | 0.002 | 1.60E-08 |  | 0.015 | 0.017 | 0.359 | 8.84E-05 | 31.895 |
| Time spent using computer | rs112600282 | 2 | 156895797 | G | A | 0.11 | -0.017 | 0.003 | 7.20E-09 |  | -0.018 | 0.023 | 0.423 | 9.28E-05 | 33.481 |
| Time spent using computer | rs113851275 | 9 | 98297220 | A | G | 0.108 | 0.019 | 0.003 | 2.50E-10 |  | 0.007 | 0.022 | 0.74 | 1.11E-04 | 39.994 |
| Time spent using computer | rs11634155 | 15 | 26693096 | C | T | 0.335 | -0.012 | 0.002 | 2.50E-09 |  | 0.002 | 0.015 | 0.876 | 9.85E-05 | 35.556 |
| Time spent using computer | rs11652437 | 17 | 79338469 | A | C | 0.324 | 0.013 | 0.002 | 1.00E-10 |  | 0.008 | 0.017 | 0.652 | 1.16E-04 | 41.772 |
| Time spent using computer | rs11749912 | 5 | 88065628 | G | A | 0.575 | -0.013 | 0.002 | 3.70E-12 |  | -0.011 | 0.014 | 0.431 | 1.34E-04 | 48.256 |
| Time spent using computer | rs11766392 | 7 | 69838127 | T | G | 0.285 | -0.013 | 0.002 | 3.10E-10 |  | -0.005 | 0.016 | 0.758 | 1.10E-04 | 39.584 |
| Time spent using computer | rs11942953 | 4 | 163753973 | C | T | 0.537 | -0.01 | 0.002 | 1.30E-08 |  | 0.007 | 0.014 | 0.594 | 8.96E-05 | 32.324 |
| Time spent using computer | rs12128707 | 1 | 72588119 | G | A | 0.264 | 0.012 | 0.002 | 7.10E-09 |  | 0.062 | 0.016 | 0 | 9.29E-05 | 33.517 |
| Time spent using computer | rs12145677 | 1 | 110023610 | A | G | 0.297 | 0.016 | 0.002 | 6.40E-15 |  | 0.004 | 0.015 | 0.815 | 1.68E-04 | 60.762 |
| Time spent using computer | rs1229984 | 4 | 100239319 | C | T | 0.973 | -0.034 | 0.006 | 4.40E-10 |  | 0.013 | 0.044 | 0.765 | 1.08E-04 | 38.91 |
| Time spent using computer | rs12521638 | 5 | 166458770 | G | A | 0.447 | 0.01 | 0.002 | 4.20E-08 |  | 0.021 | 0.014 | 0.139 | 8.33E-05 | 30.056 |
| Time spent using computer | rs12706626 | 7 | 124531370 | A | G | 0.384 | 0.01 | 0.002 | 2.90E-08 |  | 0.036 | 0.014 | 0.013 | 8.52E-05 | 30.762 |
| Time spent using computer | rs12820967 | 12 | 38921745 | C | T | 0.322 | 0.012 | 0.002 | 1.50E-09 |  | -0.008 | 0.016 | 0.632 | 1.01E-04 | 36.556 |
| Time spent using computer | rs13262595 | 8 | 143316970 | G | A | 0.561 | 0.016 | 0.002 | 1.80E-17 |  | -0.008 | 0.014 | 0.587 | 2.00E-04 | 72.37 |
| Time spent using computer | rs13422733 | 2 | 102010245 | T | C | 0.126 | -0.015 | 0.003 | 4.10E-08 |  | -0.001 | 0.022 | 0.961 | 8.34E-05 | 30.115 |
| Time spent using computer | rs136553 | 22 | 27255675 | T | C | 0.377 | 0.011 | 0.002 | 2.00E-09 |  | 0.004 | 0.014 | 0.783 | 9.97E-05 | 35.994 |
| Time spent using computer | rs1395020 | 4 | 139690326 | A | G | 0.303 | -0.011 | 0.002 | 3.40E-08 |  | 0.024 | 0.015 | 0.111 | 8.44E-05 | 30.465 |
| Time spent using computer | rs1448355 | 11 | 131286685 | T | C | 0.618 | 0.012 | 0.002 | 8.00E-11 |  | 0.021 | 0.014 | 0.142 | 1.17E-04 | 42.262 |
| Time spent using computer | rs1469249 | 5 | 113837198 | A | G | 0.211 | -0.013 | 0.002 | 8.00E-09 |  | -0.069 | 0.017 | 0 | 9.22E-05 | 33.277 |
| Time spent using computer | rs147543875 | 10 | 101624164 | T | C | 0.023 | -0.036 | 0.006 | 1.50E-08 |  | -0.055 | 0.059 | 0.352 | 8.86E-05 | 31.993 |
| Time spent using computer | rs1648906 | 18 | 35311651 | A | G | 0.321 | -0.011 | 0.002 | 2.10E-08 |  | 0.009 | 0.015 | 0.527 | 8.71E-05 | 31.428 |
| Time spent using computer | rs166835 | 15 | 47716037 | T | C | 0.556 | -0.011 | 0.002 | 4.30E-09 |  | -0.027 | 0.014 | 0.052 | 9.55E-05 | 34.481 |
| Time spent using computer | rs16912540 | 11 | 13271422 | G | A | 0.137 | -0.016 | 0.003 | 2.70E-09 |  | 0.006 | 0.021 | 0.769 | 9.80E-05 | 35.363 |
| Time spent using computer | rs17167210 | 7 | 133339343 | A | G | 0.436 | -0.011 | 0.002 | 6.00E-10 |  | -0.013 | 0.014 | 0.34 | 1.06E-04 | 38.306 |
| Time spent using computer | rs17789218 | 6 | 100600097 | C | T | 0.245 | 0.013 | 0.002 | 2.10E-09 |  | -0.005 | 0.017 | 0.77 | 9.94E-05 | 35.872 |
| Time spent using computer | rs17862355 | 7 | 126970135 | G | T | 0.442 | -0.011 | 0.002 | 1.00E-09 |  | 0.007 | 0.014 | 0.593 | 1.03E-04 | 37.243 |
| Time spent using computer | rs1987942 | 13 | 54004785 | C | T | 0.617 | -0.011 | 0.002 | 5.40E-09 |  | -0.017 | 0.014 | 0.242 | 9.43E-05 | 34.046 |
| Time spent using computer | rs2032780 | 2 | 215073935 | C | T | 0.406 | 0.012 | 0.002 | 4.50E-11 |  | 0.028 | 0.014 | 0.052 | 1.20E-04 | 43.374 |
| Time spent using computer | rs2041687 | 2 | 60405620 | G | T | 0.549 | 0.012 | 0.002 | 3.80E-10 |  | 0.008 | 0.015 | 0.591 | 1.09E-04 | 39.23 |
| Time spent using computer | rs2068625 | 4 | 159856739 | C | T | 0.698 | 0.014 | 0.002 | 1.00E-12 |  | -0.029 | 0.015 | 0.056 | 1.41E-04 | 50.776 |
| Time spent using computer | rs206965 | 12 | 120856332 | C | T | 0.791 | -0.013 | 0.002 | 7.90E-09 |  | -0.032 | 0.018 | 0.07 | 9.23E-05 | 33.296 |
| Time spent using computer | rs2120461 | 1 | 8447722 | T | C | 0.661 | 0.012 | 0.002 | 2.50E-10 |  | -0.01 | 0.015 | 0.503 | 1.11E-04 | 40.002 |
| Time spent using computer | rs246723 | 5 | 140519166 | G | A | 0.59 | -0.011 | 0.002 | 1.20E-08 |  | 0.004 | 0.014 | 0.777 | 9.01E-05 | 32.502 |
| Time spent using computer | rs2588543 | 4 | 37000406 | T | C | 0.672 | 0.011 | 0.002 | 2.20E-08 |  | 0.01 | 0.015 | 0.493 | 8.67E-05 | 31.287 |
| Time spent using computer | rs2734833 | 11 | 113292920 | A | G | 0.607 | 0.012 | 0.002 | 3.80E-11 |  | 0.019 | 0.014 | 0.176 | 1.21E-04 | 43.725 |
| Time spent using computer | rs2748985 | 1 | 1853184 | C | T | 0.544 | 0.012 | 0.002 | 9.40E-12 |  | 0.011 | 0.014 | 0.428 | 1.29E-04 | 46.447 |
| Time spent using computer | rs2761438 | 1 | 110752139 | G | A | 0.624 | -0.011 | 0.002 | 1.70E-09 |  | 0.02 | 0.015 | 0.168 | 1.00E-04 | 36.273 |
| Time spent using computer | rs28710456 | 4 | 152667171 | C | T | 0.49 | -0.01 | 0.002 | 1.20E-08 |  | -0.042 | 0.014 | 0.003 | 8.98E-05 | 32.426 |
| Time spent using computer | rs306755 | 20 | 3099752 | C | T | 0.475 | 0.01 | 0.002 | 1.40E-08 |  | 0.024 | 0.014 | 0.079 | 8.92E-05 | 32.178 |
| Time spent using computer | rs34238696 | 5 | 161356241 | G | A | 0.107 | -0.017 | 0.003 | 1.30E-08 |  | 0.011 | 0.022 | 0.606 | 8.94E-05 | 32.268 |
| Time spent using computer | rs3730399 | 16 | 67229019 | G | A | 0.064 | -0.023 | 0.004 | 9.10E-10 |  | 0.017 | 0.026 | 0.524 | 1.04E-04 | 37.507 |
| Time spent using computer | rs4704043 | 5 | 72159179 | T | C | 0.714 | 0.012 | 0.002 | 8.90E-09 |  | 0.02 | 0.015 | 0.183 | 9.16E-05 | 33.075 |
| Time spent using computer | rs4852252 | 2 | 71539301 | C | T | 0.564 | 0.01 | 0.002 | 3.00E-08 |  | 0.028 | 0.014 | 0.047 | 8.51E-05 | 30.718 |
| Time spent using computer | rs56229818 | 15 | 58662232 | C | T | 0.484 | -0.01 | 0.002 | 3.00E-08 |  | 0 | 0.014 | 0.983 | 8.51E-05 | 30.715 |
| Time spent using computer | rs58638214 | 19 | 31864938 | T | C | 0.398 | -0.013 | 0.002 | 6.70E-12 |  | -0.008 | 0.014 | 0.571 | 1.31E-04 | 47.117 |
| Time spent using computer | rs6028090 | 20 | 59856465 | A | G | 0.555 | 0.013 | 0.002 | 1.30E-11 |  | 0.02 | 0.015 | 0.156 | 1.27E-04 | 45.87 |
| Time spent using computer | rs613872 | 18 | 53210302 | T | G | 0.827 | -0.016 | 0.002 | 7.70E-11 |  | -0.021 | 0.018 | 0.244 | 1.17E-04 | 42.329 |
| Time spent using computer | rs6449708 | 5 | 50851575 | C | T | 0.532 | -0.011 | 0.002 | 7.90E-09 |  | -0.001 | 0.014 | 0.962 | 9.23E-05 | 33.302 |
| Time spent using computer | rs6780848 | 3 | 8179920 | G | T | 0.27 | 0.011 | 0.002 | 3.30E-08 |  | -0.019 | 0.016 | 0.215 | 8.46E-05 | 30.541 |
| Time spent using computer | rs6935828 | 6 | 140811367 | T | C | 0.556 | 0.01 | 0.002 | 3.10E-08 |  | 0.008 | 0.014 | 0.563 | 8.49E-05 | 30.65 |
| Time spent using computer | rs7020477 | 9 | 116827760 | G | A | 0.267 | -0.012 | 0.002 | 7.00E-09 |  | -0.011 | 0.017 | 0.515 | 9.29E-05 | 33.548 |
| Time spent using computer | rs707926 | 6 | 31748820 | A | G | 0.15 | 0.015 | 0.003 | 1.20E-08 |  | 0.005 | 0.018 | 0.77 | 9.02E-05 | 32.543 |
| Time spent using computer | rs7209653 | 17 | 19882084 | C | T | 0.295 | -0.013 | 0.002 | 2.20E-11 |  | -0.008 | 0.015 | 0.582 | 1.24E-04 | 44.772 |
| Time spent using computer | rs7281293 | 21 | 34291496 | C | A | 0.248 | 0.013 | 0.002 | 3.00E-09 |  | 0.006 | 0.016 | 0.696 | 9.75E-05 | 35.189 |
| Time spent using computer | rs72828532 | 6 | 19065342 | C | T | 0.179 | 0.016 | 0.002 | 4.50E-11 |  | 0.062 | 0.018 | 0.001 | 1.20E-04 | 43.367 |
| Time spent using computer | rs72847500 | 6 | 37643909 | C | T | 0.121 | 0.016 | 0.003 | 1.90E-08 |  | 0.036 | 0.021 | 0.088 | 8.76E-05 | 31.623 |
| Time spent using computer | rs7288455 | 22 | 39966547 | G | A | 0.567 | -0.011 | 0.002 | 1.10E-08 |  | -0.015 | 0.014 | 0.277 | 9.05E-05 | 32.647 |
| Time spent using computer | rs73578186 | 9 | 126334485 | T | C | 0.324 | -0.012 | 0.002 | 3.70E-10 |  | -0.015 | 0.015 | 0.316 | 1.09E-04 | 39.264 |
| Time spent using computer | rs7526112 | 1 | 93747683 | G | T | 0.362 | -0.011 | 0.002 | 1.10E-08 |  | -0.017 | 0.015 | 0.251 | 9.05E-05 | 32.653 |
| Time spent using computer | rs75550998 | 2 | 146486095 | T | G | 0.054 | -0.022 | 0.004 | 4.60E-08 |  | -0.025 | 0.033 | 0.456 | 8.28E-05 | 29.893 |
| Time spent using computer | rs7564844 | 2 | 215335556 | A | G | 0.701 | -0.012 | 0.002 | 5.60E-10 |  | -0.007 | 0.015 | 0.66 | 1.07E-04 | 38.453 |
| Time spent using computer | rs7630869 | 3 | 49522543 | T | C | 0.304 | 0.016 | 0.002 | 4.00E-16 |  | 0.027 | 0.015 | 0.078 | 1.84E-04 | 66.249 |
| Time spent using computer | rs76824303 | 3 | 62459819 | C | A | 0.1 | -0.02 | 0.003 | 5.80E-11 |  | -0.075 | 0.024 | 0.002 | 1.19E-04 | 42.891 |
| Time spent using computer | rs7904398 | 10 | 67954193 | T | C | 0.503 | -0.01 | 0.002 | 2.40E-08 |  | -0.029 | 0.014 | 0.036 | 8.63E-05 | 31.135 |
| Time spent using computer | rs7968738 | 12 | 90281747 | A | G | 0.264 | -0.012 | 0.002 | 2.40E-09 |  | -0.019 | 0.016 | 0.243 | 9.88E-05 | 35.659 |
| Time spent using computer | rs79720045 | 4 | 39797668 | C | T | 0.401 | -0.013 | 0.002 | 1.80E-11 |  | 0.023 | 0.015 | 0.125 | 1.25E-04 | 45.23 |
| Time spent using computer | rs806795 | 6 | 26205293 | A | G | 0.47 | 0.01 | 0.002 | 1.40E-08 |  | 0.037 | 0.014 | 0.007 | 8.93E-05 | 32.23 |
| Time spent using computer | rs8102851 | 19 | 32208909 | C | T | 0.217 | 0.013 | 0.002 | 7.60E-09 |  | 0.06 | 0.018 | 0.001 | 9.25E-05 | 33.383 |
| Time spent using computer | rs9375188 | 6 | 98555272 | T | C | 0.484 | 0.016 | 0.002 | 5.00E-18 |  | 0.065 | 0.014 | 0 | 2.07E-04 | 74.896 |
| Time spent using computer | rs9537571 | 13 | 57604700 | A | G | 0.096 | 0.019 | 0.003 | 1.10E-09 |  | 0.007 | 0.025 | 0.765 | 1.03E-04 | 37.22 |
| Time spent watching television | rs10109061 | 8 | 144239859 | G | A | 0.443 | -0.009 | 0.002 | 3.10E-08 |  | 0.043 | 0.014 | 0.002 | 7.00E-05 | 30.672 |
| Time spent watching television | rs10189857 | 2 | 60713235 | G | A | 0.432 | 0.015 | 0.002 | 5.70E-20 |  | -0.004 | 0.014 | 0.758 | 1.91E-04 | 83.718 |
| Time spent watching television | rs10269099 | 7 | 126371011 | T | G | 0.391 | 0.009 | 0.002 | 2.10E-08 |  | -0.002 | 0.014 | 0.869 | 7.17E-05 | 31.412 |
| Time spent watching television | rs10765777 | 11 | 95656385 | C | A | 0.392 | -0.011 | 0.002 | 4.90E-12 |  | 0.02 | 0.014 | 0.151 | 1.09E-04 | 47.73 |
| Time spent watching television | rs111901094 | 19 | 19513570 | T | G | 0.182 | 0.013 | 0.002 | 1.40E-09 |  | 0.009 | 0.021 | 0.675 | 8.39E-05 | 36.72 |
| Time spent watching television | rs11191129 | 10 | 103606543 | T | C | 0.423 | -0.01 | 0.002 | 9.30E-10 |  | 0.005 | 0.014 | 0.707 | 8.56E-05 | 37.466 |
| Time spent watching television | rs11222919 | 11 | 131969663 | G | T | 0.175 | -0.012 | 0.002 | 6.30E-09 |  | -0.036 | 0.019 | 0.055 | 7.71E-05 | 33.75 |
| Time spent watching television | rs11245482 | 10 | 126733546 | C | T | 0.385 | 0.01 | 0.002 | 4.80E-10 |  | 0.023 | 0.015 | 0.119 | 8.85E-05 | 38.772 |
| Time spent watching television | rs114755463 | 5 | 152503110 | A | G | 0.168 | 0.013 | 0.002 | 6.70E-10 |  | -0.018 | 0.018 | 0.331 | 8.70E-05 | 38.096 |
| Time spent watching television | rs115608101 | 11 | 107106532 | T | C | 0.13 | -0.014 | 0.002 | 1.10E-08 |  | 0.007 | 0.02 | 0.744 | 7.44E-05 | 32.599 |
| Time spent watching television | rs11662211 | 18 | 77618869 | T | C | 0.506 | -0.009 | 0.002 | 2.90E-09 |  | 0.004 | 0.014 | 0.744 | 8.05E-05 | 35.242 |
| Time spent watching television | rs11680095 | 2 | 181825956 | T | C | 0.593 | -0.009 | 0.002 | 1.90E-08 |  | -0.001 | 0.014 | 0.93 | 7.22E-05 | 31.619 |
| Time spent watching television | rs11696187 | 20 | 58891882 | T | C | 0.16 | -0.014 | 0.002 | 4.20E-10 |  | 0.006 | 0.019 | 0.767 | 8.91E-05 | 39.029 |
| Time spent watching television | rs11700249 | 20 | 11910800 | G | T | 0.409 | 0.009 | 0.002 | 5.90E-09 |  | 0.019 | 0.014 | 0.179 | 7.73E-05 | 33.86 |
| Time spent watching television | rs11714337 | 3 | 71582521 | A | G | 0.43 | -0.01 | 0.002 | 7.60E-10 |  | -0.039 | 0.014 | 0.006 | 8.64E-05 | 37.855 |
| Time spent watching television | rs11877758 | 18 | 35138110 | G | T | 0.313 | 0.011 | 0.002 | 1.30E-10 |  | -0.002 | 0.015 | 0.89 | 9.43E-05 | 41.285 |
| Time spent watching television | rs11911112 | 21 | 40528346 | C | A | 0.366 | -0.011 | 0.002 | 8.00E-11 |  | 0.036 | 0.015 | 0.014 | 9.65E-05 | 42.247 |
| Time spent watching television | rs12045585 | 1 | 243673099 | A | G | 0.131 | -0.015 | 0.002 | 4.10E-10 |  | 0.001 | 0.02 | 0.978 | 8.92E-05 | 39.068 |
| Time spent watching television | rs12214364 | 6 | 67556372 | G | T | 0.415 | 0.009 | 0.002 | 3.80E-08 |  | 0.002 | 0.015 | 0.889 | 6.90E-05 | 30.234 |
| Time spent watching television | rs1291871 | 10 | 11086083 | C | T | 0.514 | 0.009 | 0.002 | 9.90E-09 |  | -0.018 | 0.014 | 0.188 | 7.50E-05 | 32.862 |
| Time spent watching television | rs13014947 | 2 | 193742999 | A | G | 0.575 | 0.011 | 0.002 | 6.50E-11 |  | -0.013 | 0.014 | 0.364 | 9.74E-05 | 42.663 |
| Time spent watching television | rs13107325 | 4 | 103188709 | T | C | 0.075 | 0.02 | 0.003 | 1.70E-11 |  | 0.049 | 0.031 | 0.109 | 1.04E-04 | 45.326 |
| Time spent watching television | rs1324491 | 1 | 60350616 | A | G | 0.131 | 0.013 | 0.002 | 1.30E-08 |  | 0.01 | 0.021 | 0.64 | 7.39E-05 | 32.37 |
| Time spent watching television | rs1727332 | 12 | 123718301 | T | C | 0.754 | 0.013 | 0.002 | 1.10E-12 |  | 0.009 | 0.016 | 0.572 | 1.16E-04 | 50.601 |
| Time spent watching television | rs17789218 | 6 | 100600097 | C | T | 0.244 | -0.011 | 0.002 | 3.60E-09 |  | -0.005 | 0.017 | 0.77 | 7.96E-05 | 34.845 |
| Time spent watching television | rs178203 | 14 | 26959322 | C | T | 0.75 | 0.012 | 0.002 | 5.90E-11 |  | -0.041 | 0.016 | 0.011 | 9.78E-05 | 42.845 |
| Time spent watching television | rs180396 | 13 | 60437497 | T | C | 0.747 | 0.01 | 0.002 | 4.30E-08 |  | -0.001 | 0.016 | 0.962 | 6.85E-05 | 30.007 |
| Time spent watching television | rs184332798 | 18 | 53373610 | A | G | 0.026 | -0.028 | 0.005 | 2.80E-08 |  | 0.071 | 0.045 | 0.111 | 7.04E-05 | 30.815 |
| Time spent watching television | rs1889996 | 13 | 54269950 | G | T | 0.739 | 0.012 | 0.002 | 3.90E-11 |  | -0.012 | 0.016 | 0.445 | 9.97E-05 | 43.664 |
| Time spent watching television | rs1993092 | 6 | 98689604 | C | T | 0.39 | -0.01 | 0.002 | 5.40E-09 |  | 0.003 | 0.014 | 0.824 | 7.78E-05 | 34.051 |
| Time spent watching television | rs2073869 | 9 | 135763816 | T | C | 0.167 | -0.014 | 0.002 | 1.10E-10 |  | -0.036 | 0.019 | 0.053 | 9.50E-05 | 41.61 |
| Time spent watching television | rs2106164 | 7 | 92661753 | C | T | 0.532 | -0.009 | 0.002 | 4.20E-09 |  | -0.036 | 0.014 | 0.009 | 7.88E-05 | 34.513 |
| Time spent watching television | rs2185490 | 14 | 69732119 | C | A | 0.618 | 0.009 | 0.002 | 1.20E-08 |  | 0.002 | 0.014 | 0.865 | 7.41E-05 | 32.471 |
| Time spent watching television | rs2240857 | 7 | 8010634 | G | T | 0.141 | 0.016 | 0.002 | 4.50E-12 |  | 0.027 | 0.02 | 0.176 | 1.09E-04 | 47.89 |
| Time spent watching television | rs2283 | 5 | 106773623 | G | A | 0.339 | -0.009 | 0.002 | 3.20E-08 |  | 0.028 | 0.015 | 0.064 | 6.99E-05 | 30.592 |
| Time spent watching television | rs2352984 | 3 | 49948728 | C | T | 0.431 | 0.02 | 0.002 | 7.40E-35 |  | -0.032 | 0.014 | 0.021 | 3.46E-04 | 151.702 |
| Time spent watching television | rs2479968 | 13 | 111969328 | G | A | 0.051 | 0.021 | 0.004 | 2.70E-08 |  | 0.008 | 0.033 | 0.818 | 7.06E-05 | 30.922 |
| Time spent watching television | rs249960 | 5 | 96164771 | G | A | 0.182 | -0.012 | 0.002 | 1.10E-08 |  | -0.038 | 0.018 | 0.036 | 7.45E-05 | 32.633 |
| Time spent watching television | rs262890 | 5 | 62930015 | G | A | 0.299 | 0.013 | 0.002 | 2.60E-14 |  | -0.009 | 0.015 | 0.572 | 1.33E-04 | 58.042 |
| Time spent watching television | rs263771 | 2 | 185921692 | A | C | 0.233 | 0.012 | 0.002 | 1.90E-10 |  | -0.023 | 0.017 | 0.179 | 9.27E-05 | 40.602 |
| Time spent watching television | rs2646351 | 4 | 55701312 | A | G | 0.453 | 0.009 | 0.002 | 2.40E-08 |  | 0.012 | 0.014 | 0.403 | 7.10E-05 | 31.101 |
| Time spent watching television | rs2678662 | 2 | 104446759 | G | T | 0.608 | 0.011 | 0.002 | 8.60E-12 |  | -0.008 | 0.014 | 0.558 | 1.06E-04 | 46.621 |
| Time spent watching television | rs2725371 | 8 | 30854033 | G | A | 0.696 | -0.013 | 0.002 | 2.10E-14 |  | -0.039 | 0.015 | 0.01 | 1.34E-04 | 58.479 |
| Time spent watching television | rs2857693 | 6 | 31588384 | T | G | 0.366 | 0.011 | 0.002 | 9.60E-11 |  | 0.016 | 0.014 | 0.264 | 9.57E-05 | 41.906 |
| Time spent watching television | rs2906604 | 2 | 107624244 | C | T | 0.497 | 0.01 | 0.002 | 5.50E-11 |  | 0.014 | 0.014 | 0.316 | 9.82E-05 | 42.994 |
| Time spent watching television | rs3138499 | 9 | 92219921 | C | A | 0.518 | 0.011 | 0.002 | 8.50E-12 |  | -0.019 | 0.014 | 0.179 | 1.07E-04 | 46.658 |
| Time spent watching television | rs34811474 | 4 | 25408838 | A | G | 0.231 | -0.013 | 0.002 | 3.10E-12 |  | 0.041 | 0.017 | 0.014 | 1.11E-04 | 48.598 |
| Time spent watching television | rs35797019 | 3 | 93987306 | G | A | 0.392 | -0.009 | 0.002 | 1.50E-08 |  | -0.001 | 0.014 | 0.942 | 7.32E-05 | 32.04 |
| Time spent watching television | rs362312 | 4 | 3237644 | C | T | 0.424 | -0.01 | 0.002 | 3.50E-09 |  | 0.011 | 0.014 | 0.442 | 7.97E-05 | 34.892 |
| Time spent watching television | rs3754970 | 2 | 162091836 | C | T | 0.503 | 0.01 | 0.002 | 1.30E-09 |  | -0.009 | 0.014 | 0.527 | 8.41E-05 | 36.828 |
| Time spent watching television | rs3810496 | 20 | 62406886 | C | T | 0.616 | 0.009 | 0.002 | 1.20E-08 |  | 0.021 | 0.014 | 0.15 | 7.42E-05 | 32.482 |
| Time spent watching television | rs4076457 | 15 | 78007213 | T | C | 0.257 | -0.01 | 0.002 | 3.70E-08 |  | 0.008 | 0.016 | 0.602 | 6.92E-05 | 30.298 |
| Time spent watching television | rs4110177 | 5 | 88793281 | A | G | 0.367 | 0.009 | 0.002 | 1.30E-08 |  | 0.001 | 0.015 | 0.916 | 7.39E-05 | 32.375 |
| Time spent watching television | rs4303732 | 2 | 100830040 | C | T | 0.402 | -0.011 | 0.002 | 3.70E-11 |  | 0.007 | 0.014 | 0.637 | 1.00E-04 | 43.779 |
| Time spent watching television | rs4339469 | 6 | 98369230 | G | T | 0.629 | 0.013 | 0.002 | 2.80E-14 |  | -0.034 | 0.014 | 0.017 | 1.32E-04 | 57.861 |
| Time spent watching television | rs4469687 | 1 | 184679019 | G | A | 0.484 | 0.009 | 0.002 | 3.10E-08 |  | 0.001 | 0.014 | 0.918 | 6.99E-05 | 30.621 |
| Time spent watching television | rs4567133 | 9 | 22606560 | A | C | 0.81 | -0.013 | 0.002 | 1.70E-10 |  | -0.015 | 0.018 | 0.408 | 9.32E-05 | 40.821 |
| Time spent watching television | rs4747438 | 10 | 22124263 | T | C | 0.677 | -0.012 | 0.002 | 3.90E-12 |  | 0.015 | 0.015 | 0.294 | 1.10E-04 | 48.152 |
| Time spent watching television | rs4788616 | 16 | 72211984 | G | T | 0.391 | -0.01 | 0.002 | 4.30E-10 |  | 0.035 | 0.014 | 0.013 | 8.90E-05 | 38.982 |
| Time spent watching television | rs494566 | 9 | 1785717 | T | C | 0.332 | 0.01 | 0.002 | 2.00E-09 |  | -0.011 | 0.015 | 0.464 | 8.20E-05 | 35.93 |
| Time spent watching television | rs57555420 | 1 | 97783448 | T | C | 0.282 | 0.01 | 0.002 | 9.40E-09 |  | -0.047 | 0.016 | 0.003 | 7.53E-05 | 32.957 |
| Time spent watching television | rs58541850 | 6 | 166165563 | A | G | 0.059 | 0.021 | 0.003 | 2.70E-10 |  | 0.036 | 0.03 | 0.238 | 9.10E-05 | 39.872 |
| Time spent watching television | rs6102912 | 20 | 41202935 | C | T | 0.41 | -0.011 | 0.002 | 1.40E-11 |  | -0.018 | 0.014 | 0.187 | 1.04E-04 | 45.603 |
| Time spent watching television | rs6125907 | 20 | 48730315 | A | C | 0.092 | 0.016 | 0.003 | 4.50E-09 |  | 0.011 | 0.026 | 0.661 | 7.85E-05 | 34.381 |
| Time spent watching television | rs61743199 | 19 | 50161091 | G | A | 0.072 | 0.017 | 0.003 | 1.60E-08 |  | 0.013 | 0.028 | 0.629 | 7.28E-05 | 31.874 |
| Time spent watching television | rs61864793 | 10 | 85803372 | C | T | 0.252 | -0.011 | 0.002 | 1.20E-09 |  | 0.027 | 0.016 | 0.089 | 8.42E-05 | 36.894 |
| Time spent watching television | rs62145951 | 2 | 68399586 | C | T | 0.263 | -0.012 | 0.002 | 2.60E-11 |  | 0.009 | 0.016 | 0.562 | 1.01E-04 | 44.433 |
| Time spent watching television | rs62199883 | 2 | 215376706 | A | C | 0.486 | 0.014 | 0.002 | 3.20E-18 |  | 0.024 | 0.014 | 0.078 | 1.73E-04 | 75.768 |
| Time spent watching television | rs6511708 | 19 | 10788813 | C | T | 0.665 | -0.012 | 0.002 | 7.50E-13 |  | -0.022 | 0.015 | 0.149 | 1.17E-04 | 51.41 |
| Time spent watching television | rs68056254 | 2 | 147846855 | T | G | 0.151 | 0.013 | 0.002 | 1.30E-09 |  | 0.029 | 0.019 | 0.137 | 8.40E-05 | 36.792 |
| Time spent watching television | rs6814554 | 4 | 152454334 | A | G | 0.474 | 0.014 | 0.002 | 2.40E-17 |  | -0.043 | 0.014 | 0.002 | 1.64E-04 | 71.822 |
| Time spent watching television | rs6850494 | 4 | 82291771 | C | A | 0.386 | 0.01 | 0.002 | 3.40E-09 |  | -0.008 | 0.014 | 0.571 | 7.98E-05 | 34.938 |
| Time spent watching television | rs6895658 | 5 | 124274035 | C | T | 0.193 | -0.013 | 0.002 | 1.10E-10 |  | 0.003 | 0.018 | 0.86 | 9.50E-05 | 41.583 |
| Time spent watching television | rs6994132 | 8 | 92653740 | C | T | 0.579 | -0.01 | 0.002 | 6.20E-10 |  | 0.017 | 0.014 | 0.232 | 8.73E-05 | 38.25 |
| Time spent watching television | rs7089973 | 10 | 116569565 | A | C | 0.38 | 0.009 | 0.002 | 1.60E-08 |  | 0.007 | 0.014 | 0.608 | 7.28E-05 | 31.892 |
| Time spent watching television | rs7184800 | 16 | 53509131 | A | G | 0.303 | -0.013 | 0.002 | 7.40E-15 |  | -0.002 | 0.015 | 0.919 | 1.38E-04 | 60.476 |
| Time spent watching television | rs73571431 | 9 | 126136139 | T | C | 0.11 | 0.016 | 0.003 | 5.50E-10 |  | -0.026 | 0.022 | 0.236 | 8.79E-05 | 38.495 |
| Time spent watching television | rs73946726 | 2 | 117073427 | A | C | 0.02 | 0.032 | 0.006 | 2.20E-08 |  | 0.085 | 0.066 | 0.196 | 7.16E-05 | 31.349 |
| Time spent watching television | rs749056 | 1 | 110037838 | G | T | 0.304 | -0.01 | 0.002 | 7.20E-09 |  | 0.009 | 0.015 | 0.535 | 7.65E-05 | 33.481 |
| Time spent watching television | rs749671 | 16 | 31088347 | A | G | 0.372 | -0.011 | 0.002 | 1.60E-11 |  | 0.014 | 0.014 | 0.326 | 1.04E-04 | 45.388 |
| Time spent watching television | rs7539775 | 1 | 3109151 | A | G | 0.743 | 0.01 | 0.002 | 4.30E-08 |  | -0.002 | 0.016 | 0.89 | 6.85E-05 | 30.005 |
| Time spent watching television | rs75499503 | 6 | 26145217 | T | C | 0.22 | -0.018 | 0.002 | 3.10E-21 |  | 0.027 | 0.017 | 0.11 | 2.04E-04 | 89.463 |
| Time spent watching television | rs75641275 | 1 | 98327133 | C | A | 0.143 | 0.015 | 0.002 | 1.00E-11 |  | -0.02 | 0.02 | 0.31 | 1.06E-04 | 46.239 |
| Time spent watching television | rs7708324 | 5 | 147920094 | G | A | 0.377 | -0.01 | 0.002 | 1.60E-09 |  | -0.015 | 0.014 | 0.299 | 8.31E-05 | 36.404 |
| Time spent watching television | rs7798292 | 7 | 112974602 | A | G | 0.435 | -0.01 | 0.002 | 1.30E-09 |  | -0.017 | 0.014 | 0.215 | 8.40E-05 | 36.764 |
| Time spent watching television | rs78227853 | 15 | 44169073 | T | C | 0.025 | -0.028 | 0.005 | 3.40E-08 |  | 0.004 | 0.043 | 0.918 | 6.95E-05 | 30.452 |
| Time spent watching television | rs7899206 | 10 | 127188859 | G | T | 0.492 | -0.01 | 0.002 | 1.30E-09 |  | -0.002 | 0.014 | 0.881 | 8.42E-05 | 36.852 |
| Time spent watching television | rs79373894 | 15 | 73369053 | C | T | 0.034 | -0.029 | 0.004 | 9.80E-11 |  | 0.017 | 0.045 | 0.707 | 9.56E-05 | 41.862 |
| Time spent watching television | rs801733 | 11 | 65934549 | C | A | 0.358 | -0.012 | 0.002 | 5.00E-14 |  | 0.004 | 0.015 | 0.774 | 1.30E-04 | 56.723 |
| Time spent watching television | rs814197 | 1 | 61092456 | G | T | 0.467 | -0.011 | 0.002 | 2.00E-11 |  | -0.017 | 0.014 | 0.222 | 1.03E-04 | 44.948 |
| Time spent watching television | rs9300594 | 13 | 100869905 | G | A | 0.254 | 0.011 | 0.002 | 9.10E-10 |  | -0.037 | 0.016 | 0.018 | 8.56E-05 | 37.504 |
| Time spent watching television | rs9471333 | 6 | 40362023 | T | C | 0.552 | -0.01 | 0.002 | 6.70E-11 |  | 0.013 | 0.014 | 0.339 | 9.73E-05 | 42.592 |
| Time spent watching television | rs9834970 | 3 | 36856030 | C | T | 0.498 | -0.009 | 0.002 | 2.00E-08 |  | -0.013 | 0.014 | 0.359 | 7.19E-05 | 31.491 |
| Time spent watching television | rs9867121 | 3 | 114631548 | A | C | 0.184 | -0.012 | 0.002 | 1.40E-08 |  | 0.005 | 0.018 | 0.766 | 7.36E-05 | 32.239 |
| Time spent watching television | rs9867437 | 3 | 85676752 | C | A | 0.46 | -0.01 | 0.002 | 1.30E-10 |  | -0.022 | 0.014 | 0.117 | 9.45E-05 | 41.366 |
| Time spent watching television | rs9880023 | 3 | 54178199 | T | G | 0.556 | 0.01 | 0.002 | 2.00E-09 |  | -0.007 | 0.014 | 0.598 | 8.22E-05 | 35.994 |
| Time spent watching television | rs996234 | 5 | 59455212 | A | G | 0.516 | -0.011 | 0.002 | 1.60E-10 |  | -0.003 | 0.014 | 0.83 | 9.34E-05 | 40.916 |

**Note:** Abbreviations: Beta, effect allele value; SE, standard error; SNP, single nucleotide polymorphism

**Table S5.** Genetic instruments for major depression disorder

| **Exposure** | **SNP** | **Chromosome** | **Position** | **Effect allele** | **Other allele** | **Effect allele frequency** | **Exposure** | | |  | **Outcome** | | | R2 | ***F-Statistic*** |
| --- | --- | --- | --- | --- | --- | --- | --- | --- | --- | --- | --- | --- | --- | --- | --- |
|  |  |  |  |  |  |  | ***Beta*** | ***SE*** | ***P-value*** |  | ***Beta*** | ***SE*** | ***P-value*** |  |  |
| Length of mobile phone use | rs10107145 | 8 | 10758213 | G | A | 0.545 | -0.018 | 0.003 | 1.70E-10 |  | 0.001 | 0.009 | 0.89 | 8.92E-05 | 40.785 |
| Length of mobile phone use | rs10807124 | 6 | 33404064 | A | G | 0.274 | -0.018 | 0.003 | 3.10E-08 |  | 0.006 | 0.01 | 0.556 | 6.70E-05 | 30.623 |
| Length of mobile phone use | rs10828247 | 10 | 21822856 | G | A | 0.344 | 0.017 | 0.003 | 7.40E-09 |  | 0.002 | 0.009 | 0.858 | 7.31E-05 | 33.426 |
| Length of mobile phone use | rs11229008 | 11 | 57118840 | A | G | 0.063 | -0.033 | 0.006 | 3.90E-08 |  | 0.064 | 0.021 | 0.002 | 6.61E-05 | 30.222 |
| Length of mobile phone use | rs11236714 | 11 | 70424559 | T | C | 0.196 | -0.02 | 0.004 | 1.80E-08 |  | -0.001 | 0.012 | 0.963 | 6.93E-05 | 31.661 |
| Length of mobile phone use | rs11655813 | 17 | 2119101 | T | C | 0.355 | 0.018 | 0.003 | 1.20E-09 |  | -0.017 | 0.009 | 0.063 | 8.10E-05 | 37.04 |
| Length of mobile phone use | rs11682846 | 2 | 157008703 | T | C | 0.485 | -0.017 | 0.003 | 9.90E-10 |  | 0.007 | 0.009 | 0.463 | 8.17E-05 | 37.352 |
| Length of mobile phone use | rs12145998 | 1 | 204969419 | T | C | 0.266 | -0.019 | 0.003 | 2.90E-09 |  | -0.023 | 0.01 | 0.024 | 7.71E-05 | 35.224 |
| Length of mobile phone use | rs12437348 | 14 | 36606550 | A | G | 0.71 | 0.017 | 0.003 | 4.20E-08 |  | -0.018 | 0.01 | 0.074 | 6.57E-05 | 30.033 |
| Length of mobile phone use | rs13266457 | 8 | 106086786 | T | C | 0.33 | -0.017 | 0.003 | 1.70E-08 |  | 0.009 | 0.01 | 0.331 | 6.97E-05 | 31.856 |
| Length of mobile phone use | rs1512142 | 4 | 47004305 | A | G | 0.443 | -0.017 | 0.003 | 8.30E-09 |  | -0.009 | 0.009 | 0.325 | 7.27E-05 | 33.205 |
| Length of mobile phone use | rs17374152 | 5 | 93236948 | G | A | 0.239 | -0.019 | 0.003 | 5.30E-09 |  | 0.019 | 0.011 | 0.086 | 7.46E-05 | 34.088 |
| Length of mobile phone use | rs1892417 | 1 | 41779673 | C | T | 0.229 | 0.026 | 0.003 | 1.30E-14 |  | -0.008 | 0.011 | 0.451 | 1.30E-04 | 59.328 |
| Length of mobile phone use | rs2161220 | 5 | 80241904 | A | G | 0.248 | 0.021 | 0.003 | 4.00E-10 |  | 0.006 | 0.01 | 0.575 | 8.56E-05 | 39.118 |
| Length of mobile phone use | rs2836920 | 21 | 40512918 | G | T | 0.396 | 0.019 | 0.003 | 2.30E-10 |  | 0.009 | 0.009 | 0.35 | 8.80E-05 | 40.218 |
| Length of mobile phone use | rs28713780 | 7 | 3321415 | C | T | 0.641 | -0.017 | 0.003 | 1.10E-08 |  | -0.014 | 0.009 | 0.126 | 7.15E-05 | 32.685 |
| Length of mobile phone use | rs344868 | 2 | 140095800 | T | C | 0.247 | 0.018 | 0.003 | 3.20E-08 |  | 0.01 | 0.01 | 0.332 | 6.69E-05 | 30.553 |
| Length of mobile phone use | rs359265 | 2 | 60456410 | A | G | 0.607 | 0.021 | 0.003 | 6.40E-13 |  | 0.025 | 0.009 | 0.006 | 1.13E-04 | 51.715 |
| Length of mobile phone use | rs6063374 | 20 | 47832997 | G | A | 0.781 | 0.029 | 0.003 | 1.00E-17 |  | -0.015 | 0.011 | 0.159 | 1.61E-04 | 73.426 |
| Length of mobile phone use | rs6131703 | 20 | 15754684 | G | A | 0.386 | -0.018 | 0.003 | 1.90E-09 |  | 0.004 | 0.009 | 0.673 | 7.90E-05 | 36.121 |
| Length of mobile phone use | rs6780051 | 3 | 56193150 | T | G | 0.058 | 0.04 | 0.006 | 6.90E-11 |  | 0.031 | 0.02 | 0.109 | 9.31E-05 | 42.548 |
| Length of mobile phone use | rs78166132 | 5 | 161239986 | C | T | 0.094 | -0.031 | 0.005 | 4.90E-10 |  | 0.016 | 0.015 | 0.296 | 8.47E-05 | 38.728 |
| Length of mobile phone use | rs7859831 | 9 | 126205754 | T | C | 0.139 | -0.023 | 0.004 | 2.10E-08 |  | 0.012 | 0.013 | 0.339 | 6.87E-05 | 31.393 |
| Length of mobile phone use | rs8014346 | 14 | 46832002 | A | G | 0.535 | 0.019 | 0.003 | 3.70E-11 |  | 0.009 | 0.009 | 0.329 | 9.57E-05 | 43.758 |
| Length of mobile phone use | rs849527 | 2 | 206592525 | G | A | 0.547 | -0.016 | 0.003 | 1.50E-08 |  | 0.014 | 0.009 | 0.112 | 7.00E-05 | 31.995 |
| Length of mobile phone use | rs853946 | 10 | 120166472 | T | C | 0.468 | 0.016 | 0.003 | 1.80E-08 |  | -0.021 | 0.009 | 0.016 | 6.94E-05 | 31.707 |
| Length of mobile phone use | rs9896202 | 17 | 77778227 | C | T | 0.498 | -0.021 | 0.003 | 1.90E-13 |  | 0.004 | 0.01 | 0.708 | 1.18E-04 | 54.134 |
| Plays computer games | rs10170573 | 2 | 225552916 | G | A | 0.654 | -0.006 | 0.001 | 4.00E-08 |  | 0.003 | 0.009 | 0.704 | 6.52E-05 | 30.147 |
| Plays computer games | rs10799961 | 1 | 163744838 | G | A | 0.538 | 0.006 | 0.001 | 3.50E-08 |  | -0.009 | 0.009 | 0.302 | 6.57E-05 | 30.388 |
| Plays computer games | rs10803798 | 2 | 166399307 | G | T | 0.284 | 0.008 | 0.001 | 7.80E-12 |  | -0.003 | 0.01 | 0.731 | 1.01E-04 | 46.811 |
| Plays computer games | rs11223780 | 11 | 134261150 | G | A | 0.148 | 0.008 | 0.001 | 9.50E-09 |  | -0.002 | 0.013 | 0.866 | 7.12E-05 | 32.946 |
| Plays computer games | rs113002196 | 1 | 27200156 | C | T | 0.16 | 0.008 | 0.001 | 7.50E-09 |  | -0.009 | 0.012 | 0.486 | 7.22E-05 | 33.411 |
| Plays computer games | rs11740196 | 5 | 12855333 | G | A | 0.484 | -0.007 | 0.001 | 2.60E-11 |  | -0.006 | 0.009 | 0.541 | 9.61E-05 | 44.46 |
| Plays computer games | rs11743441 | 5 | 88065637 | T | G | 0.574 | -0.007 | 0.001 | 3.60E-11 |  | -0.027 | 0.009 | 0.003 | 9.47E-05 | 43.817 |
| Plays computer games | rs12129719 | 1 | 66324512 | A | G | 0.552 | 0.006 | 0.001 | 1.20E-09 |  | 0 | 0.009 | 0.969 | 8.00E-05 | 37 |
| Plays computer games | rs12138787 | 1 | 150246070 | C | T | 0.124 | 0.009 | 0.002 | 1.10E-08 |  | 0.009 | 0.014 | 0.525 | 7.05E-05 | 32.605 |
| Plays computer games | rs1232205 | 11 | 31661314 | C | T | 0.302 | -0.007 | 0.001 | 4.10E-09 |  | -0.008 | 0.01 | 0.431 | 7.48E-05 | 34.595 |
| Plays computer games | rs12361956 | 11 | 40406823 | C | T | 0.39 | -0.006 | 0.001 | 4.10E-08 |  | -0.004 | 0.009 | 0.656 | 6.51E-05 | 30.107 |
| Plays computer games | rs12707117 | 7 | 133429058 | A | G | 0.555 | 0.007 | 0.001 | 3.00E-10 |  | -0.013 | 0.009 | 0.159 | 8.58E-05 | 39.698 |
| Plays computer games | rs13262595 | 8 | 143316970 | G | A | 0.561 | 0.008 | 0.001 | 6.30E-14 |  | -0.011 | 0.009 | 0.23 | 1.22E-04 | 56.287 |
| Plays computer games | rs144377835 | 3 | 70581216 | T | C | 0.039 | 0.018 | 0.003 | 6.90E-11 |  | -0.01 | 0.024 | 0.681 | 9.20E-05 | 42.556 |
| Plays computer games | rs16831510 | 2 | 200305460 | T | G | 0.128 | 0.01 | 0.002 | 3.90E-11 |  | 0.002 | 0.014 | 0.861 | 9.44E-05 | 43.674 |
| Plays computer games | rs17057709 | 18 | 73378941 | T | C | 0.142 | -0.008 | 0.001 | 2.40E-08 |  | -0.005 | 0.014 | 0.687 | 6.73E-05 | 31.103 |
| Plays computer games | rs2014653 | 20 | 50788476 | G | A | 0.298 | -0.008 | 0.001 | 3.70E-11 |  | -0.012 | 0.01 | 0.223 | 9.47E-05 | 43.774 |
| Plays computer games | rs34217929 | 2 | 137485074 | A | G | 0.256 | 0.01 | 0.001 | 9.70E-16 |  | -0.006 | 0.01 | 0.575 | 1.39E-04 | 64.484 |
| Plays computer games | rs34402857 | 2 | 156109104 | A | G | 0.519 | 0.008 | 0.001 | 7.90E-14 |  | 0.008 | 0.009 | 0.375 | 1.21E-04 | 55.833 |
| Plays computer games | rs35104374 | 12 | 6739497 | C | T | 0.725 | -0.007 | 0.001 | 1.90E-08 |  | 0.005 | 0.011 | 0.634 | 6.82E-05 | 31.559 |
| Plays computer games | rs3757589 | 7 | 73865349 | A | G | 0.389 | -0.006 | 0.001 | 1.80E-08 |  | 0.001 | 0.01 | 0.908 | 6.85E-05 | 31.691 |
| Plays computer games | rs4113587 | 6 | 128311640 | G | T | 0.443 | 0.007 | 0.001 | 2.40E-11 |  | -0.007 | 0.009 | 0.441 | 9.65E-05 | 44.651 |
| Plays computer games | rs411844 | 7 | 101692403 | A | G | 0.821 | -0.007 | 0.001 | 4.30E-08 |  | 0.008 | 0.012 | 0.488 | 6.49E-05 | 29.999 |
| Plays computer games | rs535591 | 3 | 83154118 | A | G | 0.788 | -0.007 | 0.001 | 3.80E-08 |  | 0.017 | 0.011 | 0.115 | 6.54E-05 | 30.236 |
| Plays computer games | rs56275045 | 16 | 10201392 | A | C | 0.137 | 0.009 | 0.002 | 7.40E-09 |  | -0.018 | 0.014 | 0.195 | 7.23E-05 | 33.437 |
| Plays computer games | rs580241 | 11 | 66066349 | A | G | 0.763 | -0.007 | 0.001 | 5.90E-09 |  | 0.003 | 0.011 | 0.786 | 7.32E-05 | 33.866 |
| Plays computer games | rs62241000 | 22 | 42378507 | T | C | 0.321 | 0.006 | 0.001 | 2.90E-08 |  | -0.007 | 0.01 | 0.434 | 6.66E-05 | 30.788 |
| Plays computer games | rs623863 | 11 | 88608915 | G | A | 0.651 | 0.006 | 0.001 | 5.90E-09 |  | 0.007 | 0.009 | 0.452 | 7.32E-05 | 33.854 |
| Plays computer games | rs6457740 | 6 | 33697125 | G | A | 0.703 | -0.007 | 0.001 | 1.40E-09 |  | 0.022 | 0.01 | 0.026 | 7.93E-05 | 36.664 |
| Plays computer games | rs6536378 | 4 | 159857227 | G | T | 0.653 | 0.006 | 0.001 | 3.70E-08 |  | -0.008 | 0.009 | 0.401 | 6.55E-05 | 30.279 |
| Plays computer games | rs678007 | 1 | 23600579 | T | C | 0.178 | -0.008 | 0.001 | 3.40E-08 |  | -0.012 | 0.012 | 0.28 | 6.59E-05 | 30.473 |
| Plays computer games | rs717915 | 2 | 200714766 | T | C | 0.635 | 0.006 | 0.001 | 3.50E-08 |  | 0.004 | 0.009 | 0.671 | 6.58E-05 | 30.417 |
| Plays computer games | rs7196161 | 16 | 31110981 | A | G | 0.633 | 0.008 | 0.001 | 1.90E-12 |  | -0.011 | 0.009 | 0.221 | 1.07E-04 | 49.586 |
| Plays computer games | rs7234933 | 18 | 39692681 | C | T | 0.101 | 0.01 | 0.002 | 4.00E-08 |  | -0.006 | 0.015 | 0.689 | 6.52E-05 | 30.13 |
| Plays computer games | rs7431945 | 3 | 44029095 | A | G | 0.232 | 0.008 | 0.001 | 2.70E-10 |  | 0.024 | 0.011 | 0.024 | 8.63E-05 | 39.913 |
| Plays computer games | rs76563988 | 15 | 47947718 | G | A | 0.248 | -0.007 | 0.001 | 4.50E-09 |  | -0.026 | 0.01 | 0.011 | 7.44E-05 | 34.388 |
| Plays computer games | rs78081759 | 19 | 51130909 | A | G | 0.066 | 0.012 | 0.002 | 4.00E-09 |  | 0.015 | 0.02 | 0.456 | 7.49E-05 | 34.616 |
| Plays computer games | rs7824395 | 8 | 118922172 | G | A | 0.315 | -0.007 | 0.001 | 2.60E-09 |  | 0.007 | 0.01 | 0.449 | 7.66E-05 | 35.445 |
| Plays computer games | rs7907706 | 10 | 128491019 | A | G | 0.764 | 0.007 | 0.001 | 4.50E-08 |  | -0.013 | 0.011 | 0.237 | 6.47E-05 | 29.922 |
| Plays computer games | rs8062624 | 16 | 10168497 | C | T | 0.114 | 0.011 | 0.002 | 8.00E-11 |  | -0.042 | 0.014 | 0.004 | 9.14E-05 | 42.261 |
| Plays computer games | rs9444043 | 6 | 83915414 | C | A | 0.376 | -0.006 | 0.001 | 1.90E-09 |  | -0.015 | 0.009 | 0.101 | 7.80E-05 | 36.075 |
| Plays computer games | rs9661614 | 1 | 110021837 | C | T | 0.297 | 0.007 | 0.001 | 2.50E-10 |  | 0.024 | 0.01 | 0.011 | 8.66E-05 | 40.028 |
| Plays computer games | rs9688977 | 6 | 154336892 | C | T | 0.145 | 0.009 | 0.001 | 4.50E-09 |  | 0.019 | 0.013 | 0.124 | 7.43E-05 | 34.377 |
| Plays computer games | rs9842435 | 3 | 117517339 | G | A | 0.876 | -0.011 | 0.002 | 2.10E-12 |  | 0.03 | 0.014 | 0.027 | 1.07E-04 | 49.397 |
| Time spent using computer | rs10208088 | 2 | 221055873 | T | C | 0.58 | -0.01 | 0.002 | 3.00E-08 |  | -0.007 | 0.009 | 0.408 | 8.50E-05 | 30.685 |
| Time spent using computer | rs1037091 | 2 | 155652357 | T | C | 0.327 | -0.015 | 0.002 | 5.90E-15 |  | 0.001 | 0.01 | 0.891 | 1.69E-04 | 60.918 |
| Time spent using computer | rs10518019 | 4 | 67959875 | G | A | 0.476 | 0.01 | 0.002 | 4.90E-08 |  | -0.024 | 0.009 | 0.008 | 8.24E-05 | 29.738 |
| Time spent using computer | rs10828248 | 10 | 21824619 | G | A | 0.345 | 0.011 | 0.002 | 2.80E-08 |  | 0.003 | 0.009 | 0.769 | 8.55E-05 | 30.846 |
| Time spent using computer | rs11259902 | 15 | 83886529 | A | C | 0.2 | 0.013 | 0.002 | 1.60E-08 |  | 0.006 | 0.011 | 0.582 | 8.84E-05 | 31.895 |
| Time spent using computer | rs112600282 | 2 | 156895797 | G | A | 0.11 | -0.017 | 0.003 | 7.20E-09 |  | -0.002 | 0.014 | 0.886 | 9.28E-05 | 33.481 |
| Time spent using computer | rs113851275 | 9 | 98297220 | A | G | 0.108 | 0.019 | 0.003 | 2.50E-10 |  | 0.045 | 0.014 | 0.001 | 1.11E-04 | 39.994 |
| Time spent using computer | rs11634155 | 15 | 26693096 | C | T | 0.335 | -0.012 | 0.002 | 2.50E-09 |  | 0.005 | 0.01 | 0.601 | 9.85E-05 | 35.556 |
| Time spent using computer | rs11652437 | 17 | 79338469 | A | C | 0.324 | 0.013 | 0.002 | 1.00E-10 |  | -0.003 | 0.012 | 0.836 | 1.16E-04 | 41.772 |
| Time spent using computer | rs11749912 | 5 | 88065628 | G | A | 0.575 | -0.013 | 0.002 | 3.70E-12 |  | -0.027 | 0.009 | 0.003 | 1.34E-04 | 48.256 |
| Time spent using computer | rs11766392 | 7 | 69838127 | T | G | 0.285 | -0.013 | 0.002 | 3.10E-10 |  | 0.01 | 0.011 | 0.32 | 1.10E-04 | 39.584 |
| Time spent using computer | rs11942953 | 4 | 163753973 | C | T | 0.537 | -0.01 | 0.002 | 1.30E-08 |  | 0.01 | 0.009 | 0.28 | 8.96E-05 | 32.324 |
| Time spent using computer | rs12128707 | 1 | 72588119 | G | A | 0.264 | 0.012 | 0.002 | 7.10E-09 |  | 0.026 | 0.011 | 0.013 | 9.29E-05 | 33.517 |
| Time spent using computer | rs12145677 | 1 | 110023610 | A | G | 0.297 | 0.016 | 0.002 | 6.40E-15 |  | 0.025 | 0.01 | 0.009 | 1.68E-04 | 60.762 |
| Time spent using computer | rs1229984 | 4 | 100239319 | C | T | 0.973 | -0.034 | 0.006 | 4.40E-10 |  | -0.01 | 0.026 | 0.71 | 1.08E-04 | 38.91 |
| Time spent using computer | rs12521638 | 5 | 166458770 | G | A | 0.447 | 0.01 | 0.002 | 4.20E-08 |  | 0.003 | 0.009 | 0.724 | 8.33E-05 | 30.056 |
| Time spent using computer | rs12706626 | 7 | 124531370 | A | G | 0.384 | 0.01 | 0.002 | 2.90E-08 |  | 0.005 | 0.009 | 0.599 | 8.52E-05 | 30.762 |
| Time spent using computer | rs12820967 | 12 | 38921745 | C | T | 0.322 | 0.012 | 0.002 | 1.50E-09 |  | 0.011 | 0.01 | 0.274 | 1.01E-04 | 36.556 |
| Time spent using computer | rs13262595 | 8 | 143316970 | G | A | 0.561 | 0.016 | 0.002 | 1.80E-17 |  | -0.011 | 0.009 | 0.23 | 2.00E-04 | 72.37 |
| Time spent using computer | rs13422733 | 2 | 102010245 | T | C | 0.126 | -0.015 | 0.003 | 4.10E-08 |  | -0.002 | 0.015 | 0.879 | 8.34E-05 | 30.115 |
| Time spent using computer | rs136553 | 22 | 27255675 | T | C | 0.377 | 0.011 | 0.002 | 2.00E-09 |  | -0.011 | 0.009 | 0.249 | 9.97E-05 | 35.994 |
| Time spent using computer | rs1395020 | 4 | 139690326 | A | G | 0.303 | -0.011 | 0.002 | 3.40E-08 |  | 0.003 | 0.01 | 0.723 | 8.44E-05 | 30.465 |
| Time spent using computer | rs1448355 | 11 | 131286685 | T | C | 0.618 | 0.012 | 0.002 | 8.00E-11 |  | 0.006 | 0.009 | 0.555 | 1.17E-04 | 42.262 |
| Time spent using computer | rs1469249 | 5 | 113837198 | A | G | 0.211 | -0.013 | 0.002 | 8.00E-09 |  | -0.007 | 0.011 | 0.499 | 9.22E-05 | 33.277 |
| Time spent using computer | rs147543875 | 10 | 101624164 | T | C | 0.023 | -0.036 | 0.006 | 1.50E-08 |  | 0.01 | 0.04 | 0.808 | 8.86E-05 | 31.993 |
| Time spent using computer | rs1648906 | 18 | 35311651 | A | G | 0.321 | -0.011 | 0.002 | 2.10E-08 |  | 0.006 | 0.009 | 0.528 | 8.71E-05 | 31.428 |
| Time spent using computer | rs166835 | 15 | 47716037 | T | C | 0.556 | -0.011 | 0.002 | 4.30E-09 |  | -0.009 | 0.009 | 0.333 | 9.55E-05 | 34.481 |
| Time spent using computer | rs16912540 | 11 | 13271422 | G | A | 0.137 | -0.016 | 0.003 | 2.70E-09 |  | 0.035 | 0.013 | 0.008 | 9.80E-05 | 35.363 |
| Time spent using computer | rs17167210 | 7 | 133339343 | A | G | 0.436 | -0.011 | 0.002 | 6.00E-10 |  | 0.015 | 0.009 | 0.094 | 1.06E-04 | 38.306 |
| Time spent using computer | rs17789218 | 6 | 100600097 | C | T | 0.245 | 0.013 | 0.002 | 2.10E-09 |  | -0.006 | 0.011 | 0.563 | 9.94E-05 | 35.872 |
| Time spent using computer | rs17862355 | 7 | 126970135 | G | T | 0.442 | -0.011 | 0.002 | 1.00E-09 |  | -0.01 | 0.009 | 0.261 | 1.03E-04 | 37.243 |
| Time spent using computer | rs1987942 | 13 | 54004785 | C | T | 0.617 | -0.011 | 0.002 | 5.40E-09 |  | -0.028 | 0.009 | 0.002 | 9.43E-05 | 34.046 |
| Time spent using computer | rs2032780 | 2 | 215073935 | C | T | 0.406 | 0.012 | 0.002 | 4.50E-11 |  | 0.018 | 0.009 | 0.053 | 1.20E-04 | 43.374 |
| Time spent using computer | rs2041687 | 2 | 60405620 | G | T | 0.549 | 0.012 | 0.002 | 3.80E-10 |  | 0.018 | 0.009 | 0.057 | 1.09E-04 | 39.23 |
| Time spent using computer | rs2068625 | 4 | 159856739 | C | T | 0.698 | 0.014 | 0.002 | 1.00E-12 |  | -0.011 | 0.01 | 0.284 | 1.41E-04 | 50.776 |
| Time spent using computer | rs206965 | 12 | 120856332 | C | T | 0.791 | -0.013 | 0.002 | 7.90E-09 |  | -0.018 | 0.011 | 0.107 | 9.23E-05 | 33.296 |
| Time spent using computer | rs2120461 | 1 | 8447722 | T | C | 0.661 | 0.012 | 0.002 | 2.50E-10 |  | 0.025 | 0.01 | 0.008 | 1.11E-04 | 40.002 |
| Time spent using computer | rs246723 | 5 | 140519166 | G | A | 0.59 | -0.011 | 0.002 | 1.20E-08 |  | -0.002 | 0.009 | 0.783 | 9.01E-05 | 32.502 |
| Time spent using computer | rs2588543 | 4 | 37000406 | T | C | 0.672 | 0.011 | 0.002 | 2.20E-08 |  | 0.013 | 0.01 | 0.185 | 8.67E-05 | 31.287 |
| Time spent using computer | rs2748985 | 1 | 1853184 | C | T | 0.544 | 0.012 | 0.002 | 9.40E-12 |  | 0.001 | 0.009 | 0.891 | 1.29E-04 | 46.447 |
| Time spent using computer | rs2761438 | 1 | 110752139 | G | A | 0.624 | -0.011 | 0.002 | 1.70E-09 |  | 0.016 | 0.01 | 0.107 | 1.00E-04 | 36.273 |
| Time spent using computer | rs28710456 | 4 | 152667171 | C | T | 0.49 | -0.01 | 0.002 | 1.20E-08 |  | 0.012 | 0.009 | 0.187 | 8.98E-05 | 32.426 |
| Time spent using computer | rs306755 | 20 | 3099752 | C | T | 0.475 | 0.01 | 0.002 | 1.40E-08 |  | -0.007 | 0.009 | 0.436 | 8.92E-05 | 32.178 |
| Time spent using computer | rs34238696 | 5 | 161356241 | G | A | 0.107 | -0.017 | 0.003 | 1.30E-08 |  | 0.023 | 0.014 | 0.102 | 8.94E-05 | 32.268 |
| Time spent using computer | rs3730399 | 16 | 67229019 | G | A | 0.064 | -0.023 | 0.004 | 9.10E-10 |  | -0.006 | 0.018 | 0.742 | 1.04E-04 | 37.507 |
| Time spent using computer | rs4704043 | 5 | 72159179 | T | C | 0.714 | 0.012 | 0.002 | 8.90E-09 |  | 0.018 | 0.01 | 0.071 | 9.16E-05 | 33.075 |
| Time spent using computer | rs4852252 | 2 | 71539301 | C | T | 0.564 | 0.01 | 0.002 | 3.00E-08 |  | 0.008 | 0.009 | 0.394 | 8.51E-05 | 30.718 |
| Time spent using computer | rs56229818 | 15 | 58662232 | C | T | 0.484 | -0.01 | 0.002 | 3.00E-08 |  | 0.013 | 0.009 | 0.135 | 8.51E-05 | 30.715 |
| Time spent using computer | rs58638214 | 19 | 31864938 | T | C | 0.398 | -0.013 | 0.002 | 6.70E-12 |  | -0.005 | 0.009 | 0.623 | 1.31E-04 | 47.117 |
| Time spent using computer | rs6028090 | 20 | 59856465 | A | G | 0.555 | 0.013 | 0.002 | 1.30E-11 |  | -0.005 | 0.009 | 0.608 | 1.27E-04 | 45.87 |
| Time spent using computer | rs613872 | 18 | 53210302 | T | G | 0.827 | -0.016 | 0.002 | 7.70E-11 |  | 0.023 | 0.012 | 0.056 | 1.17E-04 | 42.329 |
| Time spent using computer | rs6449708 | 5 | 50851575 | C | T | 0.532 | -0.011 | 0.002 | 7.90E-09 |  | 0.001 | 0.009 | 0.898 | 9.23E-05 | 33.302 |
| Time spent using computer | rs6780848 | 3 | 8179920 | G | T | 0.27 | 0.011 | 0.002 | 3.30E-08 |  | -0.009 | 0.01 | 0.401 | 8.46E-05 | 30.541 |
| Time spent using computer | rs6935828 | 6 | 140811367 | T | C | 0.556 | 0.01 | 0.002 | 3.10E-08 |  | -0.003 | 0.009 | 0.707 | 8.49E-05 | 30.65 |
| Time spent using computer | rs7020477 | 9 | 116827760 | G | A | 0.267 | -0.012 | 0.002 | 7.00E-09 |  | -0.014 | 0.011 | 0.203 | 9.29E-05 | 33.548 |
| Time spent using computer | rs7209653 | 17 | 19882084 | C | T | 0.295 | -0.013 | 0.002 | 2.20E-11 |  | 0.006 | 0.01 | 0.542 | 1.24E-04 | 44.772 |
| Time spent using computer | rs7281293 | 21 | 34291496 | C | A | 0.248 | 0.013 | 0.002 | 3.00E-09 |  | -0.012 | 0.01 | 0.24 | 9.75E-05 | 35.189 |
| Time spent using computer | rs72828532 | 6 | 19065342 | C | T | 0.179 | 0.016 | 0.002 | 4.50E-11 |  | -0.01 | 0.012 | 0.405 | 1.20E-04 | 43.367 |
| Time spent using computer | rs72847500 | 6 | 37643909 | C | T | 0.121 | 0.016 | 0.003 | 1.90E-08 |  | 0.005 | 0.014 | 0.721 | 8.76E-05 | 31.623 |
| Time spent using computer | rs7288455 | 22 | 39966547 | G | A | 0.567 | -0.011 | 0.002 | 1.10E-08 |  | 0.009 | 0.009 | 0.328 | 9.05E-05 | 32.647 |
| Time spent using computer | rs73578186 | 9 | 126334485 | T | C | 0.324 | -0.012 | 0.002 | 3.70E-10 |  | 0.001 | 0.01 | 0.889 | 1.09E-04 | 39.264 |
| Time spent using computer | rs7526112 | 1 | 93747683 | G | T | 0.362 | -0.011 | 0.002 | 1.10E-08 |  | 0.025 | 0.009 | 0.006 | 9.05E-05 | 32.653 |
| Time spent using computer | rs75550998 | 2 | 146486095 | T | G | 0.054 | -0.022 | 0.004 | 4.60E-08 |  | -0.033 | 0.021 | 0.119 | 8.28E-05 | 29.893 |
| Time spent using computer | rs7564844 | 2 | 215335556 | A | G | 0.701 | -0.012 | 0.002 | 5.60E-10 |  | -0.005 | 0.01 | 0.618 | 1.07E-04 | 38.453 |
| Time spent using computer | rs7630869 | 3 | 49522543 | T | C | 0.304 | 0.016 | 0.002 | 4.00E-16 |  | -0.022 | 0.01 | 0.024 | 1.84E-04 | 66.249 |
| Time spent using computer | rs76824303 | 3 | 62459819 | C | A | 0.1 | -0.02 | 0.003 | 5.80E-11 |  | -0.026 | 0.015 | 0.079 | 1.19E-04 | 42.891 |
| Time spent using computer | rs7904398 | 10 | 67954193 | T | C | 0.503 | -0.01 | 0.002 | 2.40E-08 |  | -0.019 | 0.009 | 0.028 | 8.63E-05 | 31.135 |
| Time spent using computer | rs7968738 | 12 | 90281747 | A | G | 0.264 | -0.012 | 0.002 | 2.40E-09 |  | -0.01 | 0.01 | 0.324 | 9.88E-05 | 35.659 |
| Time spent using computer | rs79720045 | 4 | 39797668 | C | T | 0.401 | -0.013 | 0.002 | 1.80E-11 |  | 0.006 | 0.01 | 0.562 | 1.25E-04 | 45.23 |
| Time spent using computer | rs806795 | 6 | 26205293 | A | G | 0.47 | 0.01 | 0.002 | 1.40E-08 |  | 0.015 | 0.009 | 0.094 | 8.93E-05 | 32.23 |
| Time spent using computer | rs8102851 | 19 | 32208909 | C | T | 0.217 | 0.013 | 0.002 | 7.60E-09 |  | -0.002 | 0.011 | 0.849 | 9.25E-05 | 33.383 |
| Time spent using computer | rs9375188 | 6 | 98555272 | T | C | 0.484 | 0.016 | 0.002 | 5.00E-18 |  | 0.011 | 0.009 | 0.217 | 2.07E-04 | 74.896 |
| Time spent using computer | rs9537571 | 13 | 57604700 | A | G | 0.096 | 0.019 | 0.003 | 1.10E-09 |  | 0.038 | 0.015 | 0.012 | 1.03E-04 | 37.22 |
| Time spent watching television | rs10109061 | 8 | 144239859 | G | A | 0.443 | -0.009 | 0.002 | 3.10E-08 |  | -0.004 | 0.01 | 0.651 | 7.00E-05 | 30.672 |
| Time spent watching television | rs10189857 | 2 | 60713235 | G | A | 0.432 | 0.015 | 0.002 | 5.70E-20 |  | -0.004 | 0.009 | 0.645 | 1.91E-04 | 83.718 |
| Time spent watching television | rs10269099 | 7 | 126371011 | T | G | 0.391 | 0.009 | 0.002 | 2.10E-08 |  | -0.002 | 0.009 | 0.841 | 7.17E-05 | 31.412 |
| Time spent watching television | rs10765777 | 11 | 95656385 | C | A | 0.392 | -0.011 | 0.002 | 4.90E-12 |  | -0.01 | 0.009 | 0.272 | 1.09E-04 | 47.73 |
| Time spent watching television | rs11191129 | 10 | 103606543 | T | C | 0.423 | -0.01 | 0.002 | 9.30E-10 |  | 0.001 | 0.009 | 0.868 | 8.56E-05 | 37.466 |
| Time spent watching television | rs11222919 | 11 | 131969663 | G | T | 0.175 | -0.012 | 0.002 | 6.30E-09 |  | -0.005 | 0.012 | 0.668 | 7.71E-05 | 33.75 |
| Time spent watching television | rs11245482 | 10 | 126733546 | C | T | 0.385 | 0.01 | 0.002 | 4.80E-10 |  | 0.031 | 0.009 | 0.001 | 8.85E-05 | 38.772 |
| Time spent watching television | rs114755463 | 5 | 152503110 | A | G | 0.168 | 0.013 | 0.002 | 6.70E-10 |  | 0.028 | 0.012 | 0.019 | 8.70E-05 | 38.096 |
| Time spent watching television | rs115608101 | 11 | 107106532 | T | C | 0.13 | -0.014 | 0.002 | 1.10E-08 |  | 0.014 | 0.013 | 0.29 | 7.44E-05 | 32.599 |
| Time spent watching television | rs11662211 | 18 | 77618869 | T | C | 0.506 | -0.009 | 0.002 | 2.90E-09 |  | -0.023 | 0.009 | 0.011 | 8.05E-05 | 35.242 |
| Time spent watching television | rs11680095 | 2 | 181825956 | T | C | 0.593 | -0.009 | 0.002 | 1.90E-08 |  | 0.009 | 0.009 | 0.32 | 7.22E-05 | 31.619 |
| Time spent watching television | rs11696187 | 20 | 58891882 | T | C | 0.16 | -0.014 | 0.002 | 4.20E-10 |  | -0.015 | 0.012 | 0.202 | 8.91E-05 | 39.029 |
| Time spent watching television | rs11700249 | 20 | 11910800 | G | T | 0.409 | 0.009 | 0.002 | 5.90E-09 |  | 0.002 | 0.009 | 0.858 | 7.73E-05 | 33.86 |
| Time spent watching television | rs11714337 | 3 | 71582521 | A | G | 0.43 | -0.01 | 0.002 | 7.60E-10 |  | -0.007 | 0.009 | 0.427 | 8.64E-05 | 37.855 |
| Time spent watching television | rs11877758 | 18 | 35138110 | G | T | 0.313 | 0.011 | 0.002 | 1.30E-10 |  | 0.024 | 0.01 | 0.011 | 9.43E-05 | 41.285 |
| Time spent watching television | rs11911112 | 21 | 40528346 | C | A | 0.366 | -0.011 | 0.002 | 8.00E-11 |  | 0.01 | 0.009 | 0.277 | 9.65E-05 | 42.247 |
| Time spent watching television | rs12045585 | 1 | 243673099 | A | G | 0.131 | -0.015 | 0.002 | 4.10E-10 |  | -0.001 | 0.013 | 0.927 | 8.92E-05 | 39.068 |
| Time spent watching television | rs12214364 | 6 | 67556372 | G | T | 0.415 | 0.009 | 0.002 | 3.80E-08 |  | 0.009 | 0.01 | 0.352 | 6.90E-05 | 30.234 |
| Time spent watching television | rs1291871 | 10 | 11086083 | C | T | 0.514 | 0.009 | 0.002 | 9.90E-09 |  | -0.008 | 0.009 | 0.359 | 7.50E-05 | 32.862 |
| Time spent watching television | rs13014947 | 2 | 193742999 | A | G | 0.575 | 0.011 | 0.002 | 6.50E-11 |  | 0.008 | 0.009 | 0.392 | 9.74E-05 | 42.663 |
| Time spent watching television | rs13107325 | 4 | 103188709 | T | C | 0.075 | 0.02 | 0.003 | 1.70E-11 |  | -0.027 | 0.019 | 0.162 | 1.04E-04 | 45.326 |
| Time spent watching television | rs1324491 | 1 | 60350616 | A | G | 0.131 | 0.013 | 0.002 | 1.30E-08 |  | 0.005 | 0.014 | 0.706 | 7.39E-05 | 32.37 |
| Time spent watching television | rs1727332 | 12 | 123718301 | T | C | 0.754 | 0.013 | 0.002 | 1.10E-12 |  | 0.02 | 0.01 | 0.056 | 1.16E-04 | 50.601 |
| Time spent watching television | rs17789218 | 6 | 100600097 | C | T | 0.244 | -0.011 | 0.002 | 3.60E-09 |  | -0.006 | 0.011 | 0.563 | 7.96E-05 | 34.845 |
| Time spent watching television | rs178203 | 14 | 26959322 | C | T | 0.75 | 0.012 | 0.002 | 5.90E-11 |  | -0.022 | 0.01 | 0.032 | 9.78E-05 | 42.845 |
| Time spent watching television | rs180396 | 13 | 60437497 | T | C | 0.747 | 0.01 | 0.002 | 4.30E-08 |  | 0.011 | 0.01 | 0.302 | 6.85E-05 | 30.007 |
| Time spent watching television | rs1826510 | 5 | 24800012 | A | G | 0.828 | -0.013 | 0.002 | 1.90E-08 |  | -0.007 | 0.014 | 0.633 | 7.22E-05 | 31.621 |
| Time spent watching television | rs184332798 | 18 | 53373610 | A | G | 0.026 | -0.028 | 0.005 | 2.80E-08 |  | -0.003 | 0.029 | 0.917 | 7.04E-05 | 30.815 |
| Time spent watching television | rs1889996 | 13 | 54269950 | G | T | 0.739 | 0.012 | 0.002 | 3.90E-11 |  | 0.006 | 0.01 | 0.532 | 9.97E-05 | 43.664 |
| Time spent watching television | rs1993092 | 6 | 98689604 | C | T | 0.39 | -0.01 | 0.002 | 5.40E-09 |  | 0.01 | 0.009 | 0.293 | 7.78E-05 | 34.051 |
| Time spent watching television | rs2073869 | 9 | 135763816 | T | C | 0.167 | -0.014 | 0.002 | 1.10E-10 |  | 0.01 | 0.012 | 0.408 | 9.50E-05 | 41.61 |
| Time spent watching television | rs2106164 | 7 | 92661753 | C | T | 0.532 | -0.009 | 0.002 | 4.20E-09 |  | -0.003 | 0.009 | 0.76 | 7.88E-05 | 34.513 |
| Time spent watching television | rs2185490 | 14 | 69732119 | C | A | 0.618 | 0.009 | 0.002 | 1.20E-08 |  | 0.004 | 0.009 | 0.629 | 7.41E-05 | 32.471 |
| Time spent watching television | rs2240857 | 7 | 8010634 | G | T | 0.141 | 0.016 | 0.002 | 4.50E-12 |  | -0.007 | 0.013 | 0.596 | 1.09E-04 | 47.89 |
| Time spent watching television | rs2283 | 5 | 106773623 | G | A | 0.339 | -0.009 | 0.002 | 3.20E-08 |  | 0.003 | 0.01 | 0.793 | 6.99E-05 | 30.592 |
| Time spent watching television | rs2352984 | 3 | 49948728 | C | T | 0.431 | 0.02 | 0.002 | 7.40E-35 |  | 0.014 | 0.009 | 0.114 | 3.46E-04 | 151.702 |
| Time spent watching television | rs2479968 | 13 | 111969328 | G | A | 0.051 | 0.021 | 0.004 | 2.70E-08 |  | 0.023 | 0.022 | 0.289 | 7.06E-05 | 30.922 |
| Time spent watching television | rs249960 | 5 | 96164771 | G | A | 0.182 | -0.012 | 0.002 | 1.10E-08 |  | -0.009 | 0.012 | 0.457 | 7.45E-05 | 32.633 |
| Time spent watching television | rs262890 | 5 | 62930015 | G | A | 0.299 | 0.013 | 0.002 | 2.60E-14 |  | -0.021 | 0.01 | 0.032 | 1.33E-04 | 58.042 |
| Time spent watching television | rs263771 | 2 | 185921692 | A | C | 0.233 | 0.012 | 0.002 | 1.90E-10 |  | 0.022 | 0.011 | 0.045 | 9.27E-05 | 40.602 |
| Time spent watching television | rs2646351 | 4 | 55701312 | A | G | 0.453 | 0.009 | 0.002 | 2.40E-08 |  | -0.003 | 0.009 | 0.784 | 7.10E-05 | 31.101 |
| Time spent watching television | rs2678662 | 2 | 104446759 | G | T | 0.608 | 0.011 | 0.002 | 8.60E-12 |  | -0.002 | 0.009 | 0.824 | 1.06E-04 | 46.621 |
| Time spent watching television | rs2725371 | 8 | 30854033 | G | A | 0.696 | -0.013 | 0.002 | 2.10E-14 |  | -0.006 | 0.01 | 0.559 | 1.34E-04 | 58.479 |
| Time spent watching television | rs2906604 | 2 | 107624244 | C | T | 0.497 | 0.01 | 0.002 | 5.50E-11 |  | 0.006 | 0.009 | 0.504 | 9.82E-05 | 42.994 |
| Time spent watching television | rs3138499 | 9 | 92219921 | C | A | 0.518 | 0.011 | 0.002 | 8.50E-12 |  | -0.006 | 0.009 | 0.537 | 1.07E-04 | 46.658 |
| Time spent watching television | rs34094119 | 8 | 10935898 | G | A | 0.525 | 0.009 | 0.002 | 1.30E-08 |  | 0 | 0.009 | 0.98 | 7.39E-05 | 32.382 |
| Time spent watching television | rs34811474 | 4 | 25408838 | A | G | 0.231 | -0.013 | 0.002 | 3.10E-12 |  | -0.004 | 0.011 | 0.733 | 1.11E-04 | 48.598 |
| Time spent watching television | rs35797019 | 3 | 93987306 | G | A | 0.392 | -0.009 | 0.002 | 1.50E-08 |  | -0.009 | 0.009 | 0.324 | 7.32E-05 | 32.04 |
| Time spent watching television | rs362312 | 4 | 3237644 | C | T | 0.424 | -0.01 | 0.002 | 3.50E-09 |  | -0.01 | 0.009 | 0.278 | 7.97E-05 | 34.892 |
| Time spent watching television | rs3754970 | 2 | 162091836 | C | T | 0.503 | 0.01 | 0.002 | 1.30E-09 |  | 0.002 | 0.009 | 0.828 | 8.41E-05 | 36.828 |
| Time spent watching television | rs3810496 | 20 | 62406886 | C | T | 0.616 | 0.009 | 0.002 | 1.20E-08 |  | 0.008 | 0.01 | 0.415 | 7.42E-05 | 32.482 |
| Time spent watching television | rs4076457 | 15 | 78007213 | T | C | 0.257 | -0.01 | 0.002 | 3.70E-08 |  | -0.016 | 0.01 | 0.12 | 6.92E-05 | 30.298 |
| Time spent watching television | rs4110177 | 5 | 88793281 | A | G | 0.367 | 0.009 | 0.002 | 1.30E-08 |  | -0.006 | 0.009 | 0.548 | 7.39E-05 | 32.375 |
| Time spent watching television | rs4303732 | 2 | 100830040 | C | T | 0.402 | -0.011 | 0.002 | 3.70E-11 |  | -0.014 | 0.009 | 0.126 | 1.00E-04 | 43.779 |
| Time spent watching television | rs4339469 | 6 | 98369230 | G | T | 0.629 | 0.013 | 0.002 | 2.80E-14 |  | -0.002 | 0.009 | 0.828 | 1.32E-04 | 57.861 |
| Time spent watching television | rs4469687 | 1 | 184679019 | G | A | 0.484 | 0.009 | 0.002 | 3.10E-08 |  | 0.009 | 0.009 | 0.305 | 6.99E-05 | 30.621 |
| Time spent watching television | rs4567133 | 9 | 22606560 | A | C | 0.81 | -0.013 | 0.002 | 1.70E-10 |  | 0.003 | 0.011 | 0.776 | 9.32E-05 | 40.821 |
| Time spent watching television | rs4747438 | 10 | 22124263 | T | C | 0.677 | -0.012 | 0.002 | 3.90E-12 |  | 0.004 | 0.01 | 0.698 | 1.10E-04 | 48.152 |
| Time spent watching television | rs4788616 | 16 | 72211984 | G | T | 0.391 | -0.01 | 0.002 | 4.30E-10 |  | 0.016 | 0.009 | 0.077 | 8.90E-05 | 38.982 |
| Time spent watching television | rs494566 | 9 | 1785717 | T | C | 0.332 | 0.01 | 0.002 | 2.00E-09 |  | -0.006 | 0.01 | 0.506 | 8.20E-05 | 35.93 |
| Time spent watching television | rs57555420 | 1 | 97783448 | T | C | 0.282 | 0.01 | 0.002 | 9.40E-09 |  | -0.018 | 0.01 | 0.075 | 7.53E-05 | 32.957 |
| Time spent watching television | rs58541850 | 6 | 166165563 | A | G | 0.059 | 0.021 | 0.003 | 2.70E-10 |  | 0.004 | 0.019 | 0.853 | 9.10E-05 | 39.872 |
| Time spent watching television | rs6102912 | 20 | 41202935 | C | T | 0.41 | -0.011 | 0.002 | 1.40E-11 |  | 0.008 | 0.009 | 0.383 | 1.04E-04 | 45.603 |
| Time spent watching television | rs6125907 | 20 | 48730315 | A | C | 0.092 | 0.016 | 0.003 | 4.50E-09 |  | 0.024 | 0.016 | 0.141 | 7.85E-05 | 34.381 |
| Time spent watching television | rs61743199 | 19 | 50161091 | G | A | 0.072 | 0.017 | 0.003 | 1.60E-08 |  | 0.013 | 0.02 | 0.511 | 7.28E-05 | 31.874 |
| Time spent watching television | rs61864793 | 10 | 85803372 | C | T | 0.252 | -0.011 | 0.002 | 1.20E-09 |  | 0.012 | 0.01 | 0.237 | 8.42E-05 | 36.894 |
| Time spent watching television | rs62145951 | 2 | 68399586 | C | T | 0.263 | -0.012 | 0.002 | 2.60E-11 |  | 0.005 | 0.01 | 0.617 | 1.01E-04 | 44.433 |
| Time spent watching television | rs62199883 | 2 | 215376706 | A | C | 0.486 | 0.014 | 0.002 | 3.20E-18 |  | 0.007 | 0.009 | 0.46 | 1.73E-04 | 75.768 |
| Time spent watching television | rs6511708 | 19 | 10788813 | C | T | 0.665 | -0.012 | 0.002 | 7.50E-13 |  | 0.003 | 0.01 | 0.731 | 1.17E-04 | 51.41 |
| Time spent watching television | rs68056254 | 2 | 147846855 | T | G | 0.151 | 0.013 | 0.002 | 1.30E-09 |  | 0.001 | 0.012 | 0.919 | 8.40E-05 | 36.792 |
| Time spent watching television | rs6814554 | 4 | 152454334 | A | G | 0.474 | 0.014 | 0.002 | 2.40E-17 |  | 0.01 | 0.009 | 0.248 | 1.64E-04 | 71.822 |
| Time spent watching television | rs6850494 | 4 | 82291771 | C | A | 0.386 | 0.01 | 0.002 | 3.40E-09 |  | 0 | 0.009 | 0.994 | 7.98E-05 | 34.938 |
| Time spent watching television | rs6895658 | 5 | 124274035 | C | T | 0.193 | -0.013 | 0.002 | 1.10E-10 |  | 0.017 | 0.011 | 0.145 | 9.50E-05 | 41.583 |
| Time spent watching television | rs6994132 | 8 | 92653740 | C | T | 0.579 | -0.01 | 0.002 | 6.20E-10 |  | -0.004 | 0.009 | 0.685 | 8.73E-05 | 38.25 |
| Time spent watching television | rs7089973 | 10 | 116569565 | A | C | 0.38 | 0.009 | 0.002 | 1.60E-08 |  | -0.024 | 0.009 | 0.011 | 7.28E-05 | 31.892 |
| Time spent watching television | rs7184800 | 16 | 53509131 | A | G | 0.303 | -0.013 | 0.002 | 7.40E-15 |  | 0.005 | 0.01 | 0.587 | 1.38E-04 | 60.476 |
| Time spent watching television | rs73571431 | 9 | 126136139 | T | C | 0.11 | 0.016 | 0.003 | 5.50E-10 |  | 0.003 | 0.015 | 0.857 | 8.79E-05 | 38.495 |
| Time spent watching television | rs73946726 | 2 | 117073427 | A | C | 0.02 | 0.032 | 0.006 | 2.20E-08 |  | 0.067 | 0.035 | 0.056 | 7.16E-05 | 31.349 |
| Time spent watching television | rs749056 | 1 | 110037838 | G | T | 0.304 | -0.01 | 0.002 | 7.20E-09 |  | 0.028 | 0.01 | 0.004 | 7.65E-05 | 33.481 |
| Time spent watching television | rs749671 | 16 | 31088347 | A | G | 0.372 | -0.011 | 0.002 | 1.60E-11 |  | 0.014 | 0.009 | 0.125 | 1.04E-04 | 45.388 |
| Time spent watching television | rs7539775 | 1 | 3109151 | A | G | 0.743 | 0.01 | 0.002 | 4.30E-08 |  | 0.007 | 0.011 | 0.556 | 6.85E-05 | 30.005 |
| Time spent watching television | rs75499503 | 6 | 26145217 | T | C | 0.22 | -0.018 | 0.002 | 3.10E-21 |  | 0.021 | 0.011 | 0.053 | 2.04E-04 | 89.463 |
| Time spent watching television | rs75641275 | 1 | 98327133 | C | A | 0.143 | 0.015 | 0.002 | 1.00E-11 |  | -0.018 | 0.013 | 0.148 | 1.06E-04 | 46.239 |
| Time spent watching television | rs7708324 | 5 | 147920094 | G | A | 0.377 | -0.01 | 0.002 | 1.60E-09 |  | 0.004 | 0.009 | 0.653 | 8.31E-05 | 36.404 |
| Time spent watching television | rs7798292 | 7 | 112974602 | A | G | 0.435 | -0.01 | 0.002 | 1.30E-09 |  | -0.014 | 0.009 | 0.119 | 8.40E-05 | 36.764 |
| Time spent watching television | rs78227853 | 15 | 44169073 | T | C | 0.025 | -0.028 | 0.005 | 3.40E-08 |  | -0.008 | 0.027 | 0.758 | 6.95E-05 | 30.452 |
| Time spent watching television | rs7899206 | 10 | 127188859 | G | T | 0.492 | -0.01 | 0.002 | 1.30E-09 |  | 0.008 | 0.009 | 0.359 | 8.42E-05 | 36.852 |
| Time spent watching television | rs7921305 | 10 | 133775196 | A | G | 0.253 | -0.011 | 0.002 | 3.70E-10 |  | -0.012 | 0.011 | 0.277 | 8.96E-05 | 39.257 |
| Time spent watching television | rs79373894 | 15 | 73369053 | C | T | 0.034 | -0.029 | 0.004 | 9.80E-11 |  | -0.011 | 0.03 | 0.725 | 9.56E-05 | 41.862 |
| Time spent watching television | rs801733 | 11 | 65934549 | C | A | 0.358 | -0.012 | 0.002 | 5.00E-14 |  | 0.024 | 0.009 | 0.01 | 1.30E-04 | 56.723 |
| Time spent watching television | rs814197 | 1 | 61092456 | G | T | 0.467 | -0.011 | 0.002 | 2.00E-11 |  | 0.007 | 0.009 | 0.43 | 1.03E-04 | 44.948 |
| Time spent watching television | rs898751 | 17 | 2291863 | T | C | 0.493 | 0.01 | 0.002 | 3.00E-10 |  | -0.004 | 0.009 | 0.664 | 9.06E-05 | 39.664 |
| Time spent watching television | rs9300594 | 13 | 100869905 | G | A | 0.254 | 0.011 | 0.002 | 9.10E-10 |  | -0.006 | 0.01 | 0.586 | 8.56E-05 | 37.504 |
| Time spent watching television | rs9471333 | 6 | 40362023 | T | C | 0.552 | -0.01 | 0.002 | 6.70E-11 |  | 0.009 | 0.009 | 0.302 | 9.73E-05 | 42.592 |
| Time spent watching television | rs9834970 | 3 | 36856030 | C | T | 0.498 | -0.009 | 0.002 | 2.00E-08 |  | 0.005 | 0.009 | 0.56 | 7.19E-05 | 31.491 |
| Time spent watching television | rs9867121 | 3 | 114631548 | A | C | 0.184 | -0.012 | 0.002 | 1.40E-08 |  | 0.001 | 0.012 | 0.948 | 7.36E-05 | 32.239 |
| Time spent watching television | rs9867437 | 3 | 85676752 | C | A | 0.46 | -0.01 | 0.002 | 1.30E-10 |  | 0.002 | 0.009 | 0.835 | 9.45E-05 | 41.366 |
| Time spent watching television | rs9880023 | 3 | 54178199 | T | G | 0.556 | 0.01 | 0.002 | 2.00E-09 |  | -0.003 | 0.009 | 0.753 | 8.22E-05 | 35.994 |
| Time spent watching television | rs996234 | 5 | 59455212 | A | G | 0.516 | -0.011 | 0.002 | 1.60E-10 |  | 0.017 | 0.009 | 0.063 | 9.34E-05 | 40.916 |

**Note:** Abbreviations: Beta, effect allele value; SE, standard error; SNP, single nucleotide polymorphism

**Table S6.** Genetic instruments for post traumatic stress disorder

| **Exposure** | **SNP** | **Chromosome** | **Position** | **Effect allele** | **Other allele** | **Effect allele frequency** | **Exposure** | | |  | **Outcome** | | | ***R^2^*** | ***F-Statistic*** |
| --- | --- | --- | --- | --- | --- | --- | --- | --- | --- | --- | --- | --- | --- | --- | --- |
|  |  |  |  |  |  |  | ***Beta*** | ***SE*** | ***P-value*** |  | ***Beta*** | ***SE*** | ***P-value*** |  |  |
| Length of mobile phone use | rs10107145 | 8 | 10758213 | G | A | 0.545 | -0.018 | 0.003 | 1.70E-10 |  | -0.005 | 0.015 | 0.715 | 8.92E-05 | 40.785 |
| Length of mobile phone use | rs10807124 | 6 | 33404064 | A | G | 0.274 | -0.018 | 0.003 | 3.10E-08 |  | 0.013 | 0.016 | 0.427 | 6.70E-05 | 30.623 |
| Length of mobile phone use | rs10828247 | 10 | 21822856 | G | A | 0.344 | 0.017 | 0.003 | 7.40E-09 |  | 0.004 | 0.017 | 0.835 | 7.31E-05 | 33.426 |
| Length of mobile phone use | rs11229008 | 11 | 57118840 | A | G | 0.063 | -0.033 | 0.006 | 3.90E-08 |  | 0.039 | 0.032 | 0.211 | 6.61E-05 | 30.222 |
| Length of mobile phone use | rs11236714 | 11 | 70424559 | T | C | 0.196 | -0.02 | 0.004 | 1.80E-08 |  | 0.002 | 0.019 | 0.93 | 6.93E-05 | 31.661 |
| Length of mobile phone use | rs11655813 | 17 | 2119101 | T | C | 0.355 | 0.018 | 0.003 | 1.20E-09 |  | -0.019 | 0.016 | 0.218 | 8.10E-05 | 37.04 |
| Length of mobile phone use | rs11682846 | 2 | 157008703 | T | C | 0.485 | -0.017 | 0.003 | 9.90E-10 |  | 0.025 | 0.015 | 0.094 | 8.17E-05 | 37.352 |
| Length of mobile phone use | rs12145998 | 1 | 204969419 | T | C | 0.266 | -0.019 | 0.003 | 2.90E-09 |  | -0.004 | 0.017 | 0.797 | 7.71E-05 | 35.224 |
| Length of mobile phone use | rs12437348 | 14 | 36606550 | A | G | 0.71 | 0.017 | 0.003 | 4.20E-08 |  | -0.004 | 0.016 | 0.811 | 6.57E-05 | 30.033 |
| Length of mobile phone use | rs13266457 | 8 | 106086786 | T | C | 0.33 | -0.017 | 0.003 | 1.70E-08 |  | 0.042 | 0.016 | 0.007 | 6.97E-05 | 31.856 |
| Length of mobile phone use | rs1512142 | 4 | 47004305 | A | G | 0.443 | -0.017 | 0.003 | 8.30E-09 |  | -0.018 | 0.015 | 0.236 | 7.27E-05 | 33.205 |
| Length of mobile phone use | rs17156711 | 5 | 103924699 | G | A | 0.299 | -0.018 | 0.003 | 4.10E-09 |  | -0.019 | 0.016 | 0.238 | 7.56E-05 | 34.563 |
| Length of mobile phone use | rs17374152 | 5 | 93236948 | G | A | 0.239 | -0.019 | 0.003 | 5.30E-09 |  | 0 | 0.017 | 0.987 | 7.46E-05 | 34.088 |
| Length of mobile phone use | rs1892417 | 1 | 41779673 | C | T | 0.229 | 0.026 | 0.003 | 1.30E-14 |  | -0.043 | 0.018 | 0.017 | 1.30E-04 | 59.328 |
| Length of mobile phone use | rs2161220 | 5 | 80241904 | A | G | 0.248 | 0.021 | 0.003 | 4.00E-10 |  | 0.006 | 0.017 | 0.747 | 8.56E-05 | 39.118 |
| Length of mobile phone use | rs28713780 | 7 | 3321415 | C | T | 0.641 | -0.017 | 0.003 | 1.10E-08 |  | -0.031 | 0.015 | 0.04 | 7.15E-05 | 32.685 |
| Length of mobile phone use | rs344868 | 2 | 140095800 | T | C | 0.247 | 0.018 | 0.003 | 3.20E-08 |  | 0.032 | 0.017 | 0.062 | 6.69E-05 | 30.553 |
| Length of mobile phone use | rs359265 | 2 | 60456410 | A | G | 0.607 | 0.021 | 0.003 | 6.40E-13 |  | 0.046 | 0.015 | 0.003 | 1.13E-04 | 51.715 |
| Length of mobile phone use | rs6131703 | 20 | 15754684 | G | A | 0.386 | -0.018 | 0.003 | 1.90E-09 |  | 0 | 0.015 | 0.971 | 7.90E-05 | 36.121 |
| Length of mobile phone use | rs6780051 | 3 | 56193150 | T | G | 0.058 | 0.04 | 0.006 | 6.90E-11 |  | 0.06 | 0.032 | 0.062 | 9.31E-05 | 42.548 |
| Length of mobile phone use | rs78166132 | 5 | 161239986 | C | T | 0.094 | -0.031 | 0.005 | 4.90E-10 |  | 0.017 | 0.026 | 0.517 | 8.47E-05 | 38.728 |
| Length of mobile phone use | rs7859831 | 9 | 126205754 | T | C | 0.139 | -0.023 | 0.004 | 2.10E-08 |  | 0.004 | 0.022 | 0.852 | 6.87E-05 | 31.393 |
| Length of mobile phone use | rs8014346 | 14 | 46832002 | A | G | 0.535 | 0.019 | 0.003 | 3.70E-11 |  | 0.038 | 0.015 | 0.009 | 9.57E-05 | 43.758 |
| Length of mobile phone use | rs849527 | 2 | 206592525 | G | A | 0.547 | -0.016 | 0.003 | 1.50E-08 |  | 0.01 | 0.015 | 0.517 | 7.00E-05 | 31.995 |
| Length of mobile phone use | rs853946 | 10 | 120166472 | T | C | 0.468 | 0.016 | 0.003 | 1.80E-08 |  | -0.017 | 0.015 | 0.263 | 6.94E-05 | 31.707 |
| Length of mobile phone use | rs9896202 | 17 | 77778227 | C | T | 0.498 | -0.021 | 0.003 | 1.90E-13 |  | -0.006 | 0.015 | 0.679 | 1.18E-04 | 54.134 |
| Plays computer games | rs10170573 | 2 | 225552916 | G | A | 0.654 | -0.006 | 0.001 | 4.00E-08 |  | 0.01 | 0.016 | 0.509 | 6.52E-05 | 30.147 |
| Plays computer games | rs10799961 | 1 | 163744838 | G | A | 0.538 | 0.006 | 0.001 | 3.50E-08 |  | -0.021 | 0.015 | 0.152 | 6.57E-05 | 30.388 |
| Plays computer games | rs10803798 | 2 | 166399307 | G | T | 0.284 | 0.008 | 0.001 | 7.80E-12 |  | 0.017 | 0.017 | 0.305 | 1.01E-04 | 46.811 |
| Plays computer games | rs11223780 | 11 | 134261150 | G | A | 0.148 | 0.008 | 0.001 | 9.50E-09 |  | 0.029 | 0.021 | 0.163 | 7.12E-05 | 32.946 |
| Plays computer games | rs113002196 | 1 | 27200156 | C | T | 0.16 | 0.008 | 0.001 | 7.50E-09 |  | -0.009 | 0.02 | 0.651 | 7.22E-05 | 33.411 |
| Plays computer games | rs11548535 | 7 | 148936878 | T | G | 0.146 | -0.008 | 0.001 | 4.50E-08 |  | 0.021 | 0.021 | 0.307 | 6.47E-05 | 29.912 |
| Plays computer games | rs11740196 | 5 | 12855333 | G | A | 0.484 | -0.007 | 0.001 | 2.60E-11 |  | -0.028 | 0.015 | 0.057 | 9.61E-05 | 44.46 |
| Plays computer games | rs11743441 | 5 | 88065637 | T | G | 0.574 | -0.007 | 0.001 | 3.60E-11 |  | 0.012 | 0.015 | 0.44 | 9.47E-05 | 43.817 |
| Plays computer games | rs12129719 | 1 | 66324512 | A | G | 0.552 | 0.006 | 0.001 | 1.20E-09 |  | 0.008 | 0.015 | 0.606 | 8.00E-05 | 37 |
| Plays computer games | rs12138787 | 1 | 150246070 | C | T | 0.124 | 0.009 | 0.002 | 1.10E-08 |  | 0.018 | 0.022 | 0.433 | 7.05E-05 | 32.605 |
| Plays computer games | rs1232205 | 11 | 31661314 | C | T | 0.302 | -0.007 | 0.001 | 4.10E-09 |  | -0.022 | 0.016 | 0.176 | 7.48E-05 | 34.595 |
| Plays computer games | rs12361956 | 11 | 40406823 | C | T | 0.39 | -0.006 | 0.001 | 4.10E-08 |  | -0.016 | 0.015 | 0.304 | 6.51E-05 | 30.107 |
| Plays computer games | rs12707117 | 7 | 133429058 | A | G | 0.555 | 0.007 | 0.001 | 3.00E-10 |  | -0.024 | 0.015 | 0.109 | 8.58E-05 | 39.698 |
| Plays computer games | rs13262595 | 8 | 143316970 | G | A | 0.561 | 0.008 | 0.001 | 6.30E-14 |  | -0.033 | 0.015 | 0.027 | 1.22E-04 | 56.287 |
| Plays computer games | rs144377835 | 3 | 70581216 | T | C | 0.039 | 0.018 | 0.003 | 6.90E-11 |  | 0.026 | 0.042 | 0.53 | 9.20E-05 | 42.556 |
| Plays computer games | rs16831510 | 2 | 200305460 | T | G | 0.128 | 0.01 | 0.002 | 3.90E-11 |  | -0.001 | 0.022 | 0.972 | 9.44E-05 | 43.674 |
| Plays computer games | rs17057709 | 18 | 73378941 | T | C | 0.142 | -0.008 | 0.001 | 2.40E-08 |  | -0.015 | 0.023 | 0.509 | 6.73E-05 | 31.103 |
| Plays computer games | rs2014653 | 20 | 50788476 | G | A | 0.298 | -0.008 | 0.001 | 3.70E-11 |  | -0.012 | 0.017 | 0.472 | 9.47E-05 | 43.774 |
| Plays computer games | rs302719 | 1 | 8490320 | G | T | 0.34 | -0.006 | 0.001 | 6.00E-09 |  | -0.01 | 0.016 | 0.536 | 7.32E-05 | 33.848 |
| Plays computer games | rs34217929 | 2 | 137485074 | A | G | 0.256 | 0.01 | 0.001 | 9.70E-16 |  | 0.019 | 0.018 | 0.278 | 1.39E-04 | 64.484 |
| Plays computer games | rs34402857 | 2 | 156109104 | A | G | 0.519 | 0.008 | 0.001 | 7.90E-14 |  | 0.019 | 0.015 | 0.205 | 1.21E-04 | 55.833 |
| Plays computer games | rs35104374 | 12 | 6739497 | C | T | 0.725 | -0.007 | 0.001 | 1.90E-08 |  | -0.011 | 0.018 | 0.548 | 6.82E-05 | 31.559 |
| Plays computer games | rs3757589 | 7 | 73865349 | A | G | 0.389 | -0.006 | 0.001 | 1.80E-08 |  | -0.021 | 0.016 | 0.178 | 6.85E-05 | 31.691 |
| Plays computer games | rs4113587 | 6 | 128311640 | G | T | 0.443 | 0.007 | 0.001 | 2.40E-11 |  | -0.037 | 0.015 | 0.014 | 9.65E-05 | 44.651 |
| Plays computer games | rs411844 | 7 | 101692403 | A | G | 0.821 | -0.007 | 0.001 | 4.30E-08 |  | -0.033 | 0.019 | 0.083 | 6.49E-05 | 29.999 |
| Plays computer games | rs4938017 | 11 | 113303929 | T | C | 0.393 | -0.01 | 0.001 | 2.00E-19 |  | 0.017 | 0.015 | 0.249 | 1.76E-04 | 81.22 |
| Plays computer games | rs535591 | 3 | 83154118 | A | G | 0.788 | -0.007 | 0.001 | 3.80E-08 |  | -0.001 | 0.018 | 0.974 | 6.54E-05 | 30.236 |
| Plays computer games | rs56275045 | 16 | 10201392 | A | C | 0.137 | 0.009 | 0.002 | 7.40E-09 |  | -0.01 | 0.024 | 0.673 | 7.23E-05 | 33.437 |
| Plays computer games | rs580241 | 11 | 66066349 | A | G | 0.763 | -0.007 | 0.001 | 5.90E-09 |  | -0.021 | 0.018 | 0.23 | 7.32E-05 | 33.866 |
| Plays computer games | rs62241000 | 22 | 42378507 | T | C | 0.321 | 0.006 | 0.001 | 2.90E-08 |  | 0.005 | 0.016 | 0.766 | 6.66E-05 | 30.788 |
| Plays computer games | rs623863 | 11 | 88608915 | G | A | 0.651 | 0.006 | 0.001 | 5.90E-09 |  | 0.04 | 0.016 | 0.01 | 7.32E-05 | 33.854 |
| Plays computer games | rs6457740 | 6 | 33697125 | G | A | 0.703 | -0.007 | 0.001 | 1.40E-09 |  | -0.001 | 0.017 | 0.963 | 7.93E-05 | 36.664 |
| Plays computer games | rs6536378 | 4 | 159857227 | G | T | 0.653 | 0.006 | 0.001 | 3.70E-08 |  | -0.03 | 0.016 | 0.052 | 6.55E-05 | 30.279 |
| Plays computer games | rs678007 | 1 | 23600579 | T | C | 0.178 | -0.008 | 0.001 | 3.40E-08 |  | 0.014 | 0.02 | 0.462 | 6.59E-05 | 30.473 |
| Plays computer games | rs717915 | 2 | 200714766 | T | C | 0.635 | 0.006 | 0.001 | 3.50E-08 |  | 0.013 | 0.015 | 0.394 | 6.58E-05 | 30.417 |
| Plays computer games | rs7196161 | 16 | 31110981 | A | G | 0.633 | 0.008 | 0.001 | 1.90E-12 |  | 0.003 | 0.015 | 0.857 | 1.07E-04 | 49.586 |
| Plays computer games | rs7234933 | 18 | 39692681 | C | T | 0.101 | 0.01 | 0.002 | 4.00E-08 |  | -0.013 | 0.025 | 0.614 | 6.52E-05 | 30.13 |
| Plays computer games | rs7431945 | 3 | 44029095 | A | G | 0.232 | 0.008 | 0.001 | 2.70E-10 |  | 0.012 | 0.018 | 0.499 | 8.63E-05 | 39.913 |
| Plays computer games | rs76563988 | 15 | 47947718 | G | A | 0.248 | -0.007 | 0.001 | 4.50E-09 |  | -0.004 | 0.017 | 0.824 | 7.44E-05 | 34.388 |
| Plays computer games | rs78081759 | 19 | 51130909 | A | G | 0.066 | 0.012 | 0.002 | 4.00E-09 |  | 0.003 | 0.03 | 0.909 | 7.49E-05 | 34.616 |
| Plays computer games | rs7824395 | 8 | 118922172 | G | A | 0.315 | -0.007 | 0.001 | 2.60E-09 |  | -0.008 | 0.016 | 0.616 | 7.66E-05 | 35.445 |
| Plays computer games | rs7907706 | 10 | 128491019 | A | G | 0.764 | 0.007 | 0.001 | 4.50E-08 |  | 0.013 | 0.018 | 0.473 | 6.47E-05 | 29.922 |
| Plays computer games | rs8062624 | 16 | 10168497 | C | T | 0.114 | 0.011 | 0.002 | 8.00E-11 |  | -0.007 | 0.024 | 0.772 | 9.14E-05 | 42.261 |
| Plays computer games | rs9444043 | 6 | 83915414 | C | A | 0.376 | -0.006 | 0.001 | 1.90E-09 |  | -0.02 | 0.015 | 0.19 | 7.80E-05 | 36.075 |
| Plays computer games | rs9661614 | 1 | 110021837 | C | T | 0.297 | 0.007 | 0.001 | 2.50E-10 |  | 0.025 | 0.016 | 0.12 | 8.66E-05 | 40.028 |
| Plays computer games | rs9688977 | 6 | 154336892 | C | T | 0.145 | 0.009 | 0.001 | 4.50E-09 |  | 0.01 | 0.021 | 0.624 | 7.43E-05 | 34.377 |
| Plays computer games | rs9842435 | 3 | 117517339 | G | A | 0.876 | -0.011 | 0.002 | 2.10E-12 |  | 0.01 | 0.023 | 0.666 | 1.07E-04 | 49.397 |
| Time spent using computer | rs10208088 | 2 | 221055873 | T | C | 0.58 | -0.01 | 0.002 | 3.00E-08 |  | -0.016 | 0.015 | 0.283 | 8.50E-05 | 30.685 |
| Time spent using computer | rs1037091 | 2 | 155652357 | T | C | 0.327 | -0.015 | 0.002 | 5.90E-15 |  | -0.018 | 0.016 | 0.265 | 1.69E-04 | 60.918 |
| Time spent using computer | rs10518019 | 4 | 67959875 | G | A | 0.476 | 0.01 | 0.002 | 4.90E-08 |  | -0.023 | 0.015 | 0.125 | 8.24E-05 | 29.738 |
| Time spent using computer | rs10828248 | 10 | 21824619 | G | A | 0.345 | 0.011 | 0.002 | 2.80E-08 |  | 0.018 | 0.015 | 0.252 | 8.55E-05 | 30.846 |
| Time spent using computer | rs11259902 | 15 | 83886529 | A | C | 0.2 | 0.013 | 0.002 | 1.60E-08 |  | 0.017 | 0.018 | 0.35 | 8.84E-05 | 31.895 |
| Time spent using computer | rs112600282 | 2 | 156895797 | G | A | 0.11 | -0.017 | 0.003 | 7.20E-09 |  | -0.02 | 0.024 | 0.406 | 9.28E-05 | 33.481 |
| Time spent using computer | rs113851275 | 9 | 98297220 | A | G | 0.108 | 0.019 | 0.003 | 2.50E-10 |  | 0.015 | 0.024 | 0.54 | 1.11E-04 | 39.994 |
| Time spent using computer | rs11634155 | 15 | 26693096 | C | T | 0.335 | -0.012 | 0.002 | 2.50E-09 |  | 0.005 | 0.016 | 0.756 | 9.85E-05 | 35.556 |
| Time spent using computer | rs11652437 | 17 | 79338469 | A | C | 0.324 | 0.013 | 0.002 | 1.00E-10 |  | 0.021 | 0.018 | 0.231 | 1.16E-04 | 41.772 |
| Time spent using computer | rs11749912 | 5 | 88065628 | G | A | 0.575 | -0.013 | 0.002 | 3.70E-12 |  | 0.012 | 0.015 | 0.44 | 1.34E-04 | 48.256 |
| Time spent using computer | rs11766392 | 7 | 69838127 | T | G | 0.285 | -0.013 | 0.002 | 3.10E-10 |  | -0.025 | 0.017 | 0.134 | 1.10E-04 | 39.584 |
| Time spent using computer | rs11942953 | 4 | 163753973 | C | T | 0.537 | -0.01 | 0.002 | 1.30E-08 |  | -0.008 | 0.015 | 0.584 | 8.96E-05 | 32.324 |
| Time spent using computer | rs12128707 | 1 | 72588119 | G | A | 0.264 | 0.012 | 0.002 | 7.10E-09 |  | 0.033 | 0.017 | 0.053 | 9.29E-05 | 33.517 |
| Time spent using computer | rs12145677 | 1 | 110023610 | A | G | 0.297 | 0.016 | 0.002 | 6.40E-15 |  | 0.024 | 0.016 | 0.14 | 1.68E-04 | 60.762 |
| Time spent using computer | rs1229984 | 4 | 100239319 | C | T | 0.973 | -0.034 | 0.006 | 4.40E-10 |  | 0.015 | 0.047 | 0.757 | 1.08E-04 | 38.91 |
| Time spent using computer | rs12521638 | 5 | 166458770 | G | A | 0.447 | 0.01 | 0.002 | 4.20E-08 |  | -0.013 | 0.015 | 0.408 | 8.33E-05 | 30.056 |
| Time spent using computer | rs12706626 | 7 | 124531370 | A | G | 0.384 | 0.01 | 0.002 | 2.90E-08 |  | 0.026 | 0.015 | 0.094 | 8.52E-05 | 30.762 |
| Time spent using computer | rs12820967 | 12 | 38921745 | C | T | 0.322 | 0.012 | 0.002 | 1.50E-09 |  | 0.029 | 0.017 | 0.086 | 1.01E-04 | 36.556 |
| Time spent using computer | rs13262595 | 8 | 143316970 | G | A | 0.561 | 0.016 | 0.002 | 1.80E-17 |  | -0.033 | 0.015 | 0.027 | 2.00E-04 | 72.37 |
| Time spent using computer | rs13422733 | 2 | 102010245 | T | C | 0.126 | -0.015 | 0.003 | 4.10E-08 |  | -0.007 | 0.024 | 0.762 | 8.34E-05 | 30.115 |
| Time spent using computer | rs136553 | 22 | 27255675 | T | C | 0.377 | 0.011 | 0.002 | 2.00E-09 |  | -0.034 | 0.015 | 0.027 | 9.97E-05 | 35.994 |
| Time spent using computer | rs1395020 | 4 | 139690326 | A | G | 0.303 | -0.011 | 0.002 | 3.40E-08 |  | -0.016 | 0.016 | 0.315 | 8.44E-05 | 30.465 |
| Time spent using computer | rs1448355 | 11 | 131286685 | T | C | 0.618 | 0.012 | 0.002 | 8.00E-11 |  | 0.01 | 0.015 | 0.532 | 1.17E-04 | 42.262 |
| Time spent using computer | rs1469249 | 5 | 113837198 | A | G | 0.211 | -0.013 | 0.002 | 8.00E-09 |  | -0.021 | 0.018 | 0.247 | 9.22E-05 | 33.277 |
| Time spent using computer | rs147543875 | 10 | 101624164 | T | C | 0.023 | -0.036 | 0.006 | 1.50E-08 |  | 0.029 | 0.061 | 0.633 | 8.86E-05 | 31.993 |
| Time spent using computer | rs1648906 | 18 | 35311651 | A | G | 0.321 | -0.011 | 0.002 | 2.10E-08 |  | 0.018 | 0.016 | 0.246 | 8.71E-05 | 31.428 |
| Time spent using computer | rs166835 | 15 | 47716037 | T | C | 0.556 | -0.011 | 0.002 | 4.30E-09 |  | -0.006 | 0.015 | 0.7 | 9.55E-05 | 34.481 |
| Time spent using computer | rs16912540 | 11 | 13271422 | G | A | 0.137 | -0.016 | 0.003 | 2.70E-09 |  | 0.013 | 0.021 | 0.541 | 9.80E-05 | 35.363 |
| Time spent using computer | rs17167210 | 7 | 133339343 | A | G | 0.436 | -0.011 | 0.002 | 6.00E-10 |  | 0.016 | 0.015 | 0.284 | 1.06E-04 | 38.306 |
| Time spent using computer | rs17789218 | 6 | 100600097 | C | T | 0.245 | 0.013 | 0.002 | 2.10E-09 |  | -0.006 | 0.018 | 0.719 | 9.94E-05 | 35.872 |
| Time spent using computer | rs17862355 | 7 | 126970135 | G | T | 0.442 | -0.011 | 0.002 | 1.00E-09 |  | -0.017 | 0.015 | 0.252 | 1.03E-04 | 37.243 |
| Time spent using computer | rs1987942 | 13 | 54004785 | C | T | 0.617 | -0.011 | 0.002 | 5.40E-09 |  | -0.032 | 0.015 | 0.033 | 9.43E-05 | 34.046 |
| Time spent using computer | rs2032780 | 2 | 215073935 | C | T | 0.406 | 0.012 | 0.002 | 4.50E-11 |  | 0.002 | 0.015 | 0.877 | 1.20E-04 | 43.374 |
| Time spent using computer | rs2041687 | 2 | 60405620 | G | T | 0.549 | 0.012 | 0.002 | 3.80E-10 |  | 0.036 | 0.016 | 0.022 | 1.09E-04 | 39.23 |
| Time spent using computer | rs2068625 | 4 | 159856739 | C | T | 0.698 | 0.014 | 0.002 | 1.00E-12 |  | -0.015 | 0.016 | 0.351 | 1.41E-04 | 50.776 |
| Time spent using computer | rs206965 | 12 | 120856332 | C | T | 0.791 | -0.013 | 0.002 | 7.90E-09 |  | 0.008 | 0.018 | 0.671 | 9.23E-05 | 33.296 |
| Time spent using computer | rs2120461 | 1 | 8447722 | T | C | 0.661 | 0.012 | 0.002 | 2.50E-10 |  | 0.009 | 0.016 | 0.561 | 1.11E-04 | 40.002 |
| Time spent using computer | rs246723 | 5 | 140519166 | G | A | 0.59 | -0.011 | 0.002 | 1.20E-08 |  | -0.028 | 0.015 | 0.058 | 9.01E-05 | 32.502 |
| Time spent using computer | rs2588543 | 4 | 37000406 | T | C | 0.672 | 0.011 | 0.002 | 2.20E-08 |  | 0.031 | 0.016 | 0.051 | 8.67E-05 | 31.287 |
| Time spent using computer | rs2734833 | 11 | 113292920 | A | G | 0.607 | 0.012 | 0.002 | 3.80E-11 |  | -0.017 | 0.015 | 0.256 | 1.21E-04 | 43.725 |
| Time spent using computer | rs2748985 | 1 | 1853184 | C | T | 0.544 | 0.012 | 0.002 | 9.40E-12 |  | -0.006 | 0.015 | 0.678 | 1.29E-04 | 46.447 |
| Time spent using computer | rs2761438 | 1 | 110752139 | G | A | 0.624 | -0.011 | 0.002 | 1.70E-09 |  | 0.014 | 0.015 | 0.359 | 1.00E-04 | 36.273 |
| Time spent using computer | rs28710456 | 4 | 152667171 | C | T | 0.49 | -0.01 | 0.002 | 1.20E-08 |  | 0.029 | 0.015 | 0.053 | 8.98E-05 | 32.426 |
| Time spent using computer | rs306755 | 20 | 3099752 | C | T | 0.475 | 0.01 | 0.002 | 1.40E-08 |  | -0.007 | 0.015 | 0.652 | 8.92E-05 | 32.178 |
| Time spent using computer | rs34238696 | 5 | 161356241 | G | A | 0.107 | -0.017 | 0.003 | 1.30E-08 |  | 0.012 | 0.024 | 0.613 | 8.94E-05 | 32.268 |
| Time spent using computer | rs3730399 | 16 | 67229019 | G | A | 0.064 | -0.023 | 0.004 | 9.10E-10 |  | 0.038 | 0.03 | 0.202 | 1.04E-04 | 37.507 |
| Time spent using computer | rs4704043 | 5 | 72159179 | T | C | 0.714 | 0.012 | 0.002 | 8.90E-09 |  | 0.01 | 0.016 | 0.556 | 9.16E-05 | 33.075 |
| Time spent using computer | rs4852252 | 2 | 71539301 | C | T | 0.564 | 0.01 | 0.002 | 3.00E-08 |  | 0.026 | 0.015 | 0.082 | 8.51E-05 | 30.718 |
| Time spent using computer | rs56229818 | 15 | 58662232 | C | T | 0.484 | -0.01 | 0.002 | 3.00E-08 |  | 0.006 | 0.015 | 0.679 | 8.51E-05 | 30.715 |
| Time spent using computer | rs58638214 | 19 | 31864938 | T | C | 0.398 | -0.013 | 0.002 | 6.70E-12 |  | -0.006 | 0.015 | 0.696 | 1.31E-04 | 47.117 |
| Time spent using computer | rs6028090 | 20 | 59856465 | A | G | 0.555 | 0.013 | 0.002 | 1.30E-11 |  | 0.001 | 0.015 | 0.925 | 1.27E-04 | 45.87 |
| Time spent using computer | rs613872 | 18 | 53210302 | T | G | 0.827 | -0.016 | 0.002 | 7.70E-11 |  | 0.023 | 0.02 | 0.255 | 1.17E-04 | 42.329 |
| Time spent using computer | rs6449708 | 5 | 50851575 | C | T | 0.532 | -0.011 | 0.002 | 7.90E-09 |  | 0.012 | 0.015 | 0.416 | 9.23E-05 | 33.302 |
| Time spent using computer | rs6780848 | 3 | 8179920 | G | T | 0.27 | 0.011 | 0.002 | 3.30E-08 |  | -0.017 | 0.017 | 0.3 | 8.46E-05 | 30.541 |
| Time spent using computer | rs6935828 | 6 | 140811367 | T | C | 0.556 | 0.01 | 0.002 | 3.10E-08 |  | -0.002 | 0.015 | 0.88 | 8.49E-05 | 30.65 |
| Time spent using computer | rs7020477 | 9 | 116827760 | G | A | 0.267 | -0.012 | 0.002 | 7.00E-09 |  | 0.001 | 0.018 | 0.948 | 9.29E-05 | 33.548 |
| Time spent using computer | rs707926 | 6 | 31748820 | A | G | 0.15 | 0.015 | 0.003 | 1.20E-08 |  | 0.028 | 0.02 | 0.162 | 9.02E-05 | 32.543 |
| Time spent using computer | rs7209653 | 17 | 19882084 | C | T | 0.295 | -0.013 | 0.002 | 2.20E-11 |  | 0.013 | 0.016 | 0.426 | 1.24E-04 | 44.772 |
| Time spent using computer | rs7281293 | 21 | 34291496 | C | A | 0.248 | 0.013 | 0.002 | 3.00E-09 |  | -0.009 | 0.017 | 0.588 | 9.75E-05 | 35.189 |
| Time spent using computer | rs72828532 | 6 | 19065342 | C | T | 0.179 | 0.016 | 0.002 | 4.50E-11 |  | 0.005 | 0.019 | 0.81 | 1.20E-04 | 43.367 |
| Time spent using computer | rs72847500 | 6 | 37643909 | C | T | 0.121 | 0.016 | 0.003 | 1.90E-08 |  | -0.004 | 0.023 | 0.864 | 8.76E-05 | 31.623 |
| Time spent using computer | rs7288455 | 22 | 39966547 | G | A | 0.567 | -0.011 | 0.002 | 1.10E-08 |  | 0.036 | 0.015 | 0.018 | 9.05E-05 | 32.647 |
| Time spent using computer | rs73578186 | 9 | 126334485 | T | C | 0.324 | -0.012 | 0.002 | 3.70E-10 |  | 0.013 | 0.016 | 0.389 | 1.09E-04 | 39.264 |
| Time spent using computer | rs7526112 | 1 | 93747683 | G | T | 0.362 | -0.011 | 0.002 | 1.10E-08 |  | 0.015 | 0.015 | 0.316 | 9.05E-05 | 32.653 |
| Time spent using computer | rs75550998 | 2 | 146486095 | T | G | 0.054 | -0.022 | 0.004 | 4.60E-08 |  | 0.002 | 0.035 | 0.948 | 8.28E-05 | 29.893 |
| Time spent using computer | rs7564844 | 2 | 215335556 | A | G | 0.701 | -0.012 | 0.002 | 5.60E-10 |  | 0.014 | 0.016 | 0.4 | 1.07E-04 | 38.453 |
| Time spent using computer | rs7630869 | 3 | 49522543 | T | C | 0.304 | 0.016 | 0.002 | 4.00E-16 |  | 0.009 | 0.016 | 0.588 | 1.84E-04 | 66.249 |
| Time spent using computer | rs76824303 | 3 | 62459819 | C | A | 0.1 | -0.02 | 0.003 | 5.80E-11 |  | 0.002 | 0.026 | 0.928 | 1.19E-04 | 42.891 |
| Time spent using computer | rs7904398 | 10 | 67954193 | T | C | 0.503 | -0.01 | 0.002 | 2.40E-08 |  | -0.021 | 0.015 | 0.148 | 8.63E-05 | 31.135 |
| Time spent using computer | rs7968738 | 12 | 90281747 | A | G | 0.264 | -0.012 | 0.002 | 2.40E-09 |  | 0 | 0.017 | 0.985 | 9.88E-05 | 35.659 |
| Time spent using computer | rs79720045 | 4 | 39797668 | C | T | 0.401 | -0.013 | 0.002 | 1.80E-11 |  | 0.017 | 0.016 | 0.297 | 1.25E-04 | 45.23 |
| Time spent using computer | rs806795 | 6 | 26205293 | A | G | 0.47 | 0.01 | 0.002 | 1.40E-08 |  | 0.02 | 0.015 | 0.179 | 8.93E-05 | 32.23 |
| Time spent using computer | rs8102851 | 19 | 32208909 | C | T | 0.217 | 0.013 | 0.002 | 7.60E-09 |  | 0.002 | 0.019 | 0.935 | 9.25E-05 | 33.383 |
| Time spent using computer | rs9375188 | 6 | 98555272 | T | C | 0.484 | 0.016 | 0.002 | 5.00E-18 |  | -0.005 | 0.015 | 0.726 | 2.07E-04 | 74.896 |
| Time spent using computer | rs9537571 | 13 | 57604700 | A | G | 0.096 | 0.019 | 0.003 | 1.10E-09 |  | -0.003 | 0.026 | 0.9 | 1.03E-04 | 37.22 |
| Time spent watching television | rs10109061 | 8 | 144239859 | G | A | 0.443 | -0.009 | 0.002 | 3.10E-08 |  | 0.012 | 0.015 | 0.406 | 7.00E-05 | 30.672 |
| Time spent watching television | rs10189857 | 2 | 60713235 | G | A | 0.432 | 0.015 | 0.002 | 5.70E-20 |  | 0.012 | 0.015 | 0.41 | 1.91E-04 | 83.718 |
| Time spent watching television | rs10269099 | 7 | 126371011 | T | G | 0.391 | 0.009 | 0.002 | 2.10E-08 |  | 0.027 | 0.015 | 0.072 | 7.17E-05 | 31.412 |
| Time spent watching television | rs10765777 | 11 | 95656385 | C | A | 0.392 | -0.011 | 0.002 | 4.90E-12 |  | -0.002 | 0.015 | 0.875 | 1.09E-04 | 47.73 |
| Time spent watching television | rs111901094 | 19 | 19513570 | T | G | 0.182 | 0.013 | 0.002 | 1.40E-09 |  | 0.004 | 0.022 | 0.87 | 8.39E-05 | 36.72 |
| Time spent watching television | rs11191129 | 10 | 103606543 | T | C | 0.423 | -0.01 | 0.002 | 9.30E-10 |  | -0.002 | 0.015 | 0.902 | 8.56E-05 | 37.466 |
| Time spent watching television | rs11222919 | 11 | 131969663 | G | T | 0.175 | -0.012 | 0.002 | 6.30E-09 |  | 0.003 | 0.02 | 0.889 | 7.71E-05 | 33.75 |
| Time spent watching television | rs11245482 | 10 | 126733546 | C | T | 0.385 | 0.01 | 0.002 | 4.80E-10 |  | -0.025 | 0.015 | 0.103 | 8.85E-05 | 38.772 |
| Time spent watching television | rs114755463 | 5 | 152503110 | A | G | 0.168 | 0.013 | 0.002 | 6.70E-10 |  | 0.004 | 0.02 | 0.861 | 8.70E-05 | 38.096 |
| Time spent watching television | rs115608101 | 11 | 107106532 | T | C | 0.13 | -0.014 | 0.002 | 1.10E-08 |  | 0.057 | 0.022 | 0.009 | 7.44E-05 | 32.599 |
| Time spent watching television | rs11662211 | 18 | 77618869 | T | C | 0.506 | -0.009 | 0.002 | 2.90E-09 |  | 0.023 | 0.015 | 0.126 | 8.05E-05 | 35.242 |
| Time spent watching television | rs11680095 | 2 | 181825956 | T | C | 0.593 | -0.009 | 0.002 | 1.90E-08 |  | -0.002 | 0.015 | 0.903 | 7.22E-05 | 31.619 |
| Time spent watching television | rs11696187 | 20 | 58891882 | T | C | 0.16 | -0.014 | 0.002 | 4.20E-10 |  | -0.013 | 0.02 | 0.532 | 8.91E-05 | 39.029 |
| Time spent watching television | rs11700249 | 20 | 11910800 | G | T | 0.409 | 0.009 | 0.002 | 5.90E-09 |  | 0.018 | 0.015 | 0.226 | 7.73E-05 | 33.86 |
| Time spent watching television | rs11714337 | 3 | 71582521 | A | G | 0.43 | -0.01 | 0.002 | 7.60E-10 |  | -0.032 | 0.015 | 0.031 | 8.64E-05 | 37.855 |
| Time spent watching television | rs11877758 | 18 | 35138110 | G | T | 0.313 | 0.011 | 0.002 | 1.30E-10 |  | 0.008 | 0.016 | 0.609 | 9.43E-05 | 41.285 |
| Time spent watching television | rs12045585 | 1 | 243673099 | A | G | 0.131 | -0.015 | 0.002 | 4.10E-10 |  | -0.074 | 0.022 | 0.001 | 8.92E-05 | 39.068 |
| Time spent watching television | rs12214364 | 6 | 67556372 | G | T | 0.415 | 0.009 | 0.002 | 3.80E-08 |  | 0.015 | 0.016 | 0.339 | 6.90E-05 | 30.234 |
| Time spent watching television | rs1291871 | 10 | 11086083 | C | T | 0.514 | 0.009 | 0.002 | 9.90E-09 |  | 0.006 | 0.015 | 0.678 | 7.50E-05 | 32.862 |
| Time spent watching television | rs13014947 | 2 | 193742999 | A | G | 0.575 | 0.011 | 0.002 | 6.50E-11 |  | 0 | 0.015 | 0.985 | 9.74E-05 | 42.663 |
| Time spent watching television | rs1324491 | 1 | 60350616 | A | G | 0.131 | 0.013 | 0.002 | 1.30E-08 |  | 0.061 | 0.022 | 0.006 | 7.39E-05 | 32.37 |
| Time spent watching television | rs1727332 | 12 | 123718301 | T | C | 0.754 | 0.013 | 0.002 | 1.10E-12 |  | 0.009 | 0.017 | 0.602 | 1.16E-04 | 50.601 |
| Time spent watching television | rs17789218 | 6 | 100600097 | C | T | 0.244 | -0.011 | 0.002 | 3.60E-09 |  | -0.006 | 0.018 | 0.719 | 7.96E-05 | 34.845 |
| Time spent watching television | rs178203 | 14 | 26959322 | C | T | 0.75 | 0.012 | 0.002 | 5.90E-11 |  | 0.03 | 0.017 | 0.081 | 9.78E-05 | 42.845 |
| Time spent watching television | rs180396 | 13 | 60437497 | T | C | 0.747 | 0.01 | 0.002 | 4.30E-08 |  | 0.015 | 0.017 | 0.365 | 6.85E-05 | 30.007 |
| Time spent watching television | rs1826510 | 5 | 24800012 | A | G | 0.828 | -0.013 | 0.002 | 1.90E-08 |  | 0.001 | 0.024 | 0.955 | 7.22E-05 | 31.621 |
| Time spent watching television | rs184332798 | 18 | 53373610 | A | G | 0.026 | -0.028 | 0.005 | 2.80E-08 |  | 0.006 | 0.046 | 0.89 | 7.04E-05 | 30.815 |
| Time spent watching television | rs1889996 | 13 | 54269950 | G | T | 0.739 | 0.012 | 0.002 | 3.90E-11 |  | -0.034 | 0.018 | 0.062 | 9.97E-05 | 43.664 |
| Time spent watching television | rs1993092 | 6 | 98689604 | C | T | 0.39 | -0.01 | 0.002 | 5.40E-09 |  | -0.015 | 0.015 | 0.327 | 7.78E-05 | 34.051 |
| Time spent watching television | rs2073869 | 9 | 135763816 | T | C | 0.167 | -0.014 | 0.002 | 1.10E-10 |  | -0.032 | 0.02 | 0.105 | 9.50E-05 | 41.61 |
| Time spent watching television | rs2106164 | 7 | 92661753 | C | T | 0.532 | -0.009 | 0.002 | 4.20E-09 |  | 0.022 | 0.015 | 0.132 | 7.88E-05 | 34.513 |
| Time spent watching television | rs2185490 | 14 | 69732119 | C | A | 0.618 | 0.009 | 0.002 | 1.20E-08 |  | 0.008 | 0.015 | 0.613 | 7.41E-05 | 32.471 |
| Time spent watching television | rs2240857 | 7 | 8010634 | G | T | 0.141 | 0.016 | 0.002 | 4.50E-12 |  | -0.029 | 0.022 | 0.186 | 1.09E-04 | 47.89 |
| Time spent watching television | rs2283 | 5 | 106773623 | G | A | 0.339 | -0.009 | 0.002 | 3.20E-08 |  | 0.014 | 0.016 | 0.38 | 6.99E-05 | 30.592 |
| Time spent watching television | rs2352984 | 3 | 49948728 | C | T | 0.431 | 0.02 | 0.002 | 7.40E-35 |  | -0.003 | 0.015 | 0.825 | 3.46E-04 | 151.702 |
| Time spent watching television | rs2479968 | 13 | 111969328 | G | A | 0.051 | 0.021 | 0.004 | 2.70E-08 |  | 0.055 | 0.035 | 0.121 | 7.06E-05 | 30.922 |
| Time spent watching television | rs249960 | 5 | 96164771 | G | A | 0.182 | -0.012 | 0.002 | 1.10E-08 |  | 0.048 | 0.019 | 0.013 | 7.45E-05 | 32.633 |
| Time spent watching television | rs262890 | 5 | 62930015 | G | A | 0.299 | 0.013 | 0.002 | 2.60E-14 |  | -0.017 | 0.016 | 0.304 | 1.33E-04 | 58.042 |
| Time spent watching television | rs263771 | 2 | 185921692 | A | C | 0.233 | 0.012 | 0.002 | 1.90E-10 |  | 0.002 | 0.018 | 0.921 | 9.27E-05 | 40.602 |
| Time spent watching television | rs2646351 | 4 | 55701312 | A | G | 0.453 | 0.009 | 0.002 | 2.40E-08 |  | 0.012 | 0.015 | 0.429 | 7.10E-05 | 31.101 |
| Time spent watching television | rs2678662 | 2 | 104446759 | G | T | 0.608 | 0.011 | 0.002 | 8.60E-12 |  | 0.011 | 0.015 | 0.464 | 1.06E-04 | 46.621 |
| Time spent watching television | rs2725371 | 8 | 30854033 | G | A | 0.696 | -0.013 | 0.002 | 2.10E-14 |  | -0.013 | 0.016 | 0.402 | 1.34E-04 | 58.479 |
| Time spent watching television | rs2857693 | 6 | 31588384 | T | G | 0.366 | 0.011 | 0.002 | 9.60E-11 |  | 0.027 | 0.015 | 0.072 | 9.57E-05 | 41.906 |
| Time spent watching television | rs2906604 | 2 | 107624244 | C | T | 0.497 | 0.01 | 0.002 | 5.50E-11 |  | 0.026 | 0.015 | 0.077 | 9.82E-05 | 42.994 |
| Time spent watching television | rs3138499 | 9 | 92219921 | C | A | 0.518 | 0.011 | 0.002 | 8.50E-12 |  | 0.013 | 0.015 | 0.388 | 1.07E-04 | 46.658 |
| Time spent watching television | rs34094119 | 8 | 10935898 | G | A | 0.525 | 0.009 | 0.002 | 1.30E-08 |  | -0.014 | 0.015 | 0.342 | 7.39E-05 | 32.382 |
| Time spent watching television | rs34811474 | 4 | 25408838 | A | G | 0.231 | -0.013 | 0.002 | 3.10E-12 |  | -0.037 | 0.018 | 0.043 | 1.11E-04 | 48.598 |
| Time spent watching television | rs35797019 | 3 | 93987306 | G | A | 0.392 | -0.009 | 0.002 | 1.50E-08 |  | 0.013 | 0.015 | 0.368 | 7.32E-05 | 32.04 |
| Time spent watching television | rs362312 | 4 | 3237644 | C | T | 0.424 | -0.01 | 0.002 | 3.50E-09 |  | -0.016 | 0.015 | 0.285 | 7.97E-05 | 34.892 |
| Time spent watching television | rs3754970 | 2 | 162091836 | C | T | 0.503 | 0.01 | 0.002 | 1.30E-09 |  | -0.018 | 0.015 | 0.231 | 8.41E-05 | 36.828 |
| Time spent watching television | rs3810496 | 20 | 62406886 | C | T | 0.616 | 0.009 | 0.002 | 1.20E-08 |  | -0.005 | 0.015 | 0.753 | 7.42E-05 | 32.482 |
| Time spent watching television | rs4076457 | 15 | 78007213 | T | C | 0.257 | -0.01 | 0.002 | 3.70E-08 |  | -0.037 | 0.017 | 0.031 | 6.92E-05 | 30.298 |
| Time spent watching television | rs4110177 | 5 | 88793281 | A | G | 0.367 | 0.009 | 0.002 | 1.30E-08 |  | 0.02 | 0.015 | 0.183 | 7.39E-05 | 32.375 |
| Time spent watching television | rs4303732 | 2 | 100830040 | C | T | 0.402 | -0.011 | 0.002 | 3.70E-11 |  | 0.025 | 0.015 | 0.096 | 1.00E-04 | 43.779 |
| Time spent watching television | rs4339469 | 6 | 98369230 | G | T | 0.629 | 0.013 | 0.002 | 2.80E-14 |  | 0.016 | 0.015 | 0.29 | 1.32E-04 | 57.861 |
| Time spent watching television | rs4469687 | 1 | 184679019 | G | A | 0.484 | 0.009 | 0.002 | 3.10E-08 |  | -0.02 | 0.015 | 0.181 | 6.99E-05 | 30.621 |
| Time spent watching television | rs4567133 | 9 | 22606560 | A | C | 0.81 | -0.013 | 0.002 | 1.70E-10 |  | 0.007 | 0.02 | 0.727 | 9.32E-05 | 40.821 |
| Time spent watching television | rs4747438 | 10 | 22124263 | T | C | 0.677 | -0.012 | 0.002 | 3.90E-12 |  | -0.017 | 0.016 | 0.268 | 1.10E-04 | 48.152 |
| Time spent watching television | rs4788616 | 16 | 72211984 | G | T | 0.391 | -0.01 | 0.002 | 4.30E-10 |  | 0.013 | 0.015 | 0.375 | 8.90E-05 | 38.982 |
| Time spent watching television | rs494566 | 9 | 1785717 | T | C | 0.332 | 0.01 | 0.002 | 2.00E-09 |  | 0.015 | 0.016 | 0.358 | 8.20E-05 | 35.93 |
| Time spent watching television | rs57555420 | 1 | 97783448 | T | C | 0.282 | 0.01 | 0.002 | 9.40E-09 |  | 0.002 | 0.017 | 0.902 | 7.53E-05 | 32.957 |
| Time spent watching television | rs58541850 | 6 | 166165563 | A | G | 0.059 | 0.021 | 0.003 | 2.70E-10 |  | 0.02 | 0.033 | 0.55 | 9.10E-05 | 39.872 |
| Time spent watching television | rs6102912 | 20 | 41202935 | C | T | 0.41 | -0.011 | 0.002 | 1.40E-11 |  | 0.029 | 0.015 | 0.05 | 1.04E-04 | 45.603 |
| Time spent watching television | rs6125907 | 20 | 48730315 | A | C | 0.092 | 0.016 | 0.003 | 4.50E-09 |  | -0.019 | 0.026 | 0.474 | 7.85E-05 | 34.381 |
| Time spent watching television | rs61743199 | 19 | 50161091 | G | A | 0.072 | 0.017 | 0.003 | 1.60E-08 |  | 0.036 | 0.03 | 0.222 | 7.28E-05 | 31.874 |
| Time spent watching television | rs61864793 | 10 | 85803372 | C | T | 0.252 | -0.011 | 0.002 | 1.20E-09 |  | 0.005 | 0.017 | 0.761 | 8.42E-05 | 36.894 |
| Time spent watching television | rs62145951 | 2 | 68399586 | C | T | 0.263 | -0.012 | 0.002 | 2.60E-11 |  | -0.009 | 0.017 | 0.58 | 1.01E-04 | 44.433 |
| Time spent watching television | rs62199883 | 2 | 215376706 | A | C | 0.486 | 0.014 | 0.002 | 3.20E-18 |  | 0.031 | 0.015 | 0.034 | 1.73E-04 | 75.768 |
| Time spent watching television | rs6511708 | 19 | 10788813 | C | T | 0.665 | -0.012 | 0.002 | 7.50E-13 |  | 0.028 | 0.016 | 0.071 | 1.17E-04 | 51.41 |
| Time spent watching television | rs68056254 | 2 | 147846855 | T | G | 0.151 | 0.013 | 0.002 | 1.30E-09 |  | 0.025 | 0.02 | 0.229 | 8.40E-05 | 36.792 |
| Time spent watching television | rs6814554 | 4 | 152454334 | A | G | 0.474 | 0.014 | 0.002 | 2.40E-17 |  | 0.018 | 0.015 | 0.217 | 1.64E-04 | 71.822 |
| Time spent watching television | rs6850494 | 4 | 82291771 | C | A | 0.386 | 0.01 | 0.002 | 3.40E-09 |  | -0.013 | 0.015 | 0.406 | 7.98E-05 | 34.938 |
| Time spent watching television | rs6895658 | 5 | 124274035 | C | T | 0.193 | -0.013 | 0.002 | 1.10E-10 |  | 0.001 | 0.019 | 0.949 | 9.50E-05 | 41.583 |
| Time spent watching television | rs6994132 | 8 | 92653740 | C | T | 0.579 | -0.01 | 0.002 | 6.20E-10 |  | 0.001 | 0.015 | 0.962 | 8.73E-05 | 38.25 |
| Time spent watching television | rs7089973 | 10 | 116569565 | A | C | 0.38 | 0.009 | 0.002 | 1.60E-08 |  | -0.012 | 0.015 | 0.442 | 7.28E-05 | 31.892 |
| Time spent watching television | rs7184800 | 16 | 53509131 | A | G | 0.303 | -0.013 | 0.002 | 7.40E-15 |  | -0.007 | 0.016 | 0.653 | 1.38E-04 | 60.476 |
| Time spent watching television | rs73571431 | 9 | 126136139 | T | C | 0.11 | 0.016 | 0.003 | 5.50E-10 |  | 0.026 | 0.024 | 0.273 | 8.79E-05 | 38.495 |
| Time spent watching television | rs73946726 | 2 | 117073427 | A | C | 0.02 | 0.032 | 0.006 | 2.20E-08 |  | 0.063 | 0.056 | 0.26 | 7.16E-05 | 31.349 |
| Time spent watching television | rs749056 | 1 | 110037838 | G | T | 0.304 | -0.01 | 0.002 | 7.20E-09 |  | 0.021 | 0.016 | 0.192 | 7.65E-05 | 33.481 |
| Time spent watching television | rs749671 | 16 | 31088347 | A | G | 0.372 | -0.011 | 0.002 | 1.60E-11 |  | -0.011 | 0.015 | 0.468 | 1.04E-04 | 45.388 |
| Time spent watching television | rs7539775 | 1 | 3109151 | A | G | 0.743 | 0.01 | 0.002 | 4.30E-08 |  | -0.01 | 0.017 | 0.553 | 6.85E-05 | 30.005 |
| Time spent watching television | rs75499503 | 6 | 26145217 | T | C | 0.22 | -0.018 | 0.002 | 3.10E-21 |  | -0.017 | 0.018 | 0.339 | 2.04E-04 | 89.463 |
| Time spent watching television | rs75641275 | 1 | 98327133 | C | A | 0.143 | 0.015 | 0.002 | 1.00E-11 |  | 0.014 | 0.021 | 0.513 | 1.06E-04 | 46.239 |
| Time spent watching television | rs7708324 | 5 | 147920094 | G | A | 0.377 | -0.01 | 0.002 | 1.60E-09 |  | -0.014 | 0.015 | 0.373 | 8.31E-05 | 36.404 |
| Time spent watching television | rs7798292 | 7 | 112974602 | A | G | 0.435 | -0.01 | 0.002 | 1.30E-09 |  | -0.001 | 0.015 | 0.96 | 8.40E-05 | 36.764 |
| Time spent watching television | rs78227853 | 15 | 44169073 | T | C | 0.025 | -0.028 | 0.005 | 3.40E-08 |  | -0.01 | 0.046 | 0.829 | 6.95E-05 | 30.452 |
| Time spent watching television | rs7899206 | 10 | 127188859 | G | T | 0.492 | -0.01 | 0.002 | 1.30E-09 |  | 0.009 | 0.015 | 0.556 | 8.42E-05 | 36.852 |
| Time spent watching television | rs7921305 | 10 | 133775196 | A | G | 0.253 | -0.011 | 0.002 | 3.70E-10 |  | -0.006 | 0.017 | 0.746 | 8.96E-05 | 39.257 |
| Time spent watching television | rs79373894 | 15 | 73369053 | C | T | 0.034 | -0.029 | 0.004 | 9.80E-11 |  | 0.008 | 0.043 | 0.849 | 9.56E-05 | 41.862 |
| Time spent watching television | rs801733 | 11 | 65934549 | C | A | 0.358 | -0.012 | 0.002 | 5.00E-14 |  | -0.025 | 0.016 | 0.11 | 1.30E-04 | 56.723 |
| Time spent watching television | rs814197 | 1 | 61092456 | G | T | 0.467 | -0.011 | 0.002 | 2.00E-11 |  | 0.015 | 0.015 | 0.301 | 1.03E-04 | 44.948 |
| Time spent watching television | rs898751 | 17 | 2291863 | T | C | 0.493 | 0.01 | 0.002 | 3.00E-10 |  | -0.013 | 0.015 | 0.401 | 9.06E-05 | 39.664 |
| Time spent watching television | rs9300594 | 13 | 100869905 | G | A | 0.254 | 0.011 | 0.002 | 9.10E-10 |  | 0.005 | 0.017 | 0.761 | 8.56E-05 | 37.504 |
| Time spent watching television | rs9471333 | 6 | 40362023 | T | C | 0.552 | -0.01 | 0.002 | 6.70E-11 |  | 0.02 | 0.015 | 0.184 | 9.73E-05 | 42.592 |
| Time spent watching television | rs9834970 | 3 | 36856030 | C | T | 0.498 | -0.009 | 0.002 | 2.00E-08 |  | 0.012 | 0.015 | 0.432 | 7.19E-05 | 31.491 |
| Time spent watching television | rs9867121 | 3 | 114631548 | A | C | 0.184 | -0.012 | 0.002 | 1.40E-08 |  | -0.058 | 0.019 | 0.003 | 7.36E-05 | 32.239 |
| Time spent watching television | rs9867437 | 3 | 85676752 | C | A | 0.46 | -0.01 | 0.002 | 1.30E-10 |  | 0.013 | 0.015 | 0.378 | 9.45E-05 | 41.366 |
| Time spent watching television | rs9880023 | 3 | 54178199 | T | G | 0.556 | 0.01 | 0.002 | 2.00E-09 |  | -0.012 | 0.015 | 0.427 | 8.22E-05 | 35.994 |
| Time spent watching television | rs996234 | 5 | 59455212 | A | G | 0.516 | -0.011 | 0.002 | 1.60E-10 |  | 0.013 | 0.015 | 0.389 | 9.34E-05 | 40.916 |

**Note:** Abbreviations: Beta, effect allele value; SE, standard error; SNP, single nucleotide polymorphism

**Table S7.** The causal effect of digital device use on the risk of the five psychiatric disorders (SNPs reaching *P* < 5×10-8)

| **Exposure** | **Outcome** | **Method** | **Number of SNVs** | **OR (95% CI)** | ***P-value*** | ***P-FDR*** |
| --- | --- | --- | --- | --- | --- | --- |
| **Digital devices use on risk of ADHD** |  |  |  |  |  |  |
| Length of mobile phone use | ADHD | MR Egger | 26 | 0.72 (0.18, 2.82) | 0.639 | 0.825 |
|  |  | **Weighted median** | **26** | **1.66 (1.19, 2.31)** | **0.003** | **0.040** |
|  |  | Simple mode | 26 | 2.49 (1.23, 5.00) | 0.017 | 0.194 |
|  |  | Weighted mode | 26 | 1.58 (0.73, 3.46) | 0.260 | 0.618 |
| Time spent using computer | ADHD | MR Egger | 72 | 1.43 (0.39, 5.27) | 0.594 | 0.792 |
|  |  | Weighted median | 72 | 1.03 (0.77, 1.39) | 0.832 | 0.937 |
|  |  | Simple mode | 72 | 1.65 (0.68, 3.97) | 0.271 | 0.618 |
|  |  | Weighted mode | 72 | 1.55 (0.69, 3.51) | 0.294 | 0.618 |
| Plays computer games | ADHD | MR Egger | 40 | 4.73 (0.12, 189.98) | 0.414 | 0.637 |
|  |  | Weighted median | 40 | 2.01 (0.97, 4.14) | 0.059 | 0.429 |
|  |  | Simple mode | 40 | 2.44 (0.38, 15.49) | 0.351 | 0.618 |
|  |  | Weighted mode | 40 | 2.24 (0.41, 12.23) | 0.358 | 0.618 |
| Time spent watching television | ADHD | MR Egger | 97 | 2.20 (0.69, 7.00) | 0.184 | 0.618 |
|  |  | **Weighted median** | **97** | **3.64 (2.72, 4.87)** | **< 0.001** | **< 0.001** |
|  |  | **Simple mode** | **97** | **4.34 (1.87, 10.07)** | **< 0.001** | **< 0.001** |
|  |  | **Weighted mode** | **97** | **4.64 (2.08, 10.37)** | **< 0.001** | **< 0.001** |
| **Digital devices use on risk of AD** |  |  |  |  |  |  |
| Length of mobile phone use | AD | MR Egger | 28 | 0.21 (0.01, 5.96) | 0.371 | 0.618 |
|  |  | Weighted median | 28 | 1.00 (0.42, 2.39) | 0.994 | 0.994 |
|  |  | Simple mode | 28 | 0.47 (0.07, 3.27) | 0.448 | 0.664 |
|  |  | Weighted mode | 28 | 0.44 (0.07, 2.87) | 0.396 | 0.621 |
| Time spent using computer | AD | MR Egger | 74 | 2.02 (0.07, 61.55) | 0.688 | 0.847 |
|  |  | Weighted median | 74 | 1.17 (0.50, 2.75) | 0.719 | 0.872 |
|  |  | Simple mode | 74 | 2.48 (0.33, 18.79) | 0.382 | 0.618 |
|  |  | Weighted mode | 74 | 2.18 (0.32, 14.88) | 0.428 | 0.646 |
| **Plays computer games** | **AD** | **MR Egger** | **46** | **0 (0, 0.01)** | **0.001** | **0.016** |
|  |  | Weighted median | 46 | 2.83 (0.42, 19.11) | 0.286 | 0.618 |
|  |  | Simple mode | 46 | 10.58 (0.11, 1030.14) | 0.318 | 0.618 |
|  |  | Weighted mode | 46 | 9.12 (0.11, 776.91) | 0.335 | 0.618 |
| Time spent watching television | AD | MR Egger | 97 | 0.22 (0.01, 4.08) | 0.311 | 0.618 |
|  |  | Weighted median | 97 | 1.25 (0.56, 2.79) | 0.585 | 0.792 |
|  |  | Simple mode | 97 | 2.78 (0.38, 20.19) | 0.315 | 0.618 |
|  |  | Weighted mode | 97 | 1.71 (0.27, 10.80) | 0.572 | 0.789 |
| **Digital devices use on risk of ASD** |  |  |  |  |  |  |
| Length of mobile phone use | ASD | MR Egger | 27 | 1.45 (0.25, 8.42) | 0.683 | 0.847 |
|  |  | Weighted median | 27 | 0.79 (0.50, 1.23) | 0.294 | 0.618 |
|  |  | Simple mode | 27 | 0.90 (0.36, 2.25) | 0.823 | 0.937 |
|  |  | Weighted mode | 27 | 0.89 (0.38, 2.07) | 0.787 | 0.917 |
| Time spent using computer | ASD | MR Egger | 75 | 2.55 (0.37, 17.58) | 0.345 | 0.618 |
|  |  | **Weighted median** | **75** | **2.10 (1.37, 3.21)** | **< 0.001** | **< 0.001** |
|  |  | Simple mode | 75 | 2.22 (0.75, 6.52) | 0.153 | 0.618 |
|  |  | Weighted mode | 75 | 2.13 (0.76, 5.92) | 0.153 | 0.618 |
| Plays computer games | ASD | MR Egger | 47 | 19.22 (0.3, 1241.74) | 0.171 | 0.618 |
|  |  | Weighted median | 47 | 2.05 (0.82, 5.15) | 0.126 | 0.618 |
|  |  | Simple mode | 47 | 4.71 (0.39, 56.64) | 0.229 | 0.618 |
|  |  | Weighted mode | 47 | 5.25 (0.60, 46.36) | 0.142 | 0.618 |
| Time spent watching television | ASD | MR Egger | 97 | 0.60 (0.12, 3.04) | 0.535 | 0.764 |
|  |  | Weighted median | 97 | 0.72 (0.47, 1.12) | 0.143 | 0.618 |
|  |  | Simple mode | 97 | 0.65 (0.18, 2.39) | 0.521 | 0.758 |
|  |  | Weighted mode | 97 | 0.57 (0.16, 1.97) | 0.375 | 0.618 |
| **Digital devices use on risk of PTSD** |  |  |  |  |  |  |
| Length of mobile phone use | PTSD | MR Egger | 26 | 1.14 (0.09, 14.88) | 0.924 | 0.965 |
|  |  | Weighted median | 26 | 1.05 (0.62, 1.76) | 0.869 | 0.965 |
|  |  | Simple mode | 26 | 0.92 (0.29, 2.93) | 0.890 | 0.965 |
|  |  | Weighted mode | 26 | 0.97 (0.31, 3.06) | 0.953 | 0.977 |
| Time spent using computer | PTSD | MR Egger | 75 | 0.46 (0.08, 2.52) | 0.373 | 0.618 |
|  |  | Weighted median | 75 | 0.89 (0.57, 1.40) | 0.620 | 0.813 |
|  |  | Simple mode | 75 | 0.54 (0.16, 1.90) | 0.341 | 0.618 |
|  |  | Weighted mode | 75 | 0.55 (0.16, 1.91) | 0.351 | 0.618 |
| Plays computer games | PTSD | MR Egger | 47 | 0.59 (0.01, 29.67) | 0.791 | 0.917 |
|  |  | Weighted median | 47 | 3.26 (1.21, 8.82) | 0.020 | 0.200 |
|  |  | Simple mode | 47 | 5.11 (0.57, 45.43) | 0.150 | 0.618 |
|  |  | Weighted mode | 47 | 5.11 (0.53, 49.40) | 0.166 | 0.618 |
| Time spent watching television | PTSD | MR Egger | 99 | 6.05 (1.29, 28.36) | 0.025 | 0.222 |
|  |  | Weighted median | 99 | 1.56 (1.00, 2.41) | 0.049 | 0.392 |
|  |  | Simple mode | 99 | 1.99 (0.59, 6.65) | 0.268 | 0.618 |
|  |  | Weighted mode | 99 | 2.04 (0.66, 6.33) | 0.220 | 0.618 |
| **Digital devices use on risk of MDD** |  |  |  |  |  |  |
| Length of mobile phone use | MDD | MR Egger | 27 | 0.80 (0.20, 3.23) | 0.755 | 0.901 |
|  |  | Weighted median | 27 | 0.80 (0.59, 1.09) | 0.158 | 0.618 |
|  |  | Simple mode | 27 | 0.72 (0.36, 1.42) | 0.349 | 0.618 |
|  |  | Weighted mode | 27 | 0.72 (0.41, 1.26) | 0.259 | 0.618 |
| Time spent using computer | MDD | MR Egger | 73 | 1.86 (0.51, 6.87) | 0.353 | 0.618 |
|  |  | Weighted median | 73 | 1.14 (0.85, 1.55) | 0.386 | 0.618 |
|  |  | Simple mode | 73 | 0.96 (0.39, 2.37) | 0.929 | 0.965 |
|  |  | Weighted mode | 73 | 0.96 (0.41, 2.23) | 0.924 | 0.965 |
| Plays computer games | MDD | MR Egger | 44 | 0.27 (0.02, 4.85) | 0.379 | 0.618 |
|  |  | Weighted median | 44 | 0.65 (0.34, 1.23) | 0.183 | 0.618 |
|  |  | Simple mode | 44 | 0.46 (0.11, 1.88) | 0.282 | 0.618 |
|  |  | Weighted mode | 44 | 0.44 (0.11, 1.84) | 0.270 | 0.618 |
| Time spent watching television | MDD | MR Egger | 99 | 1.33 (0.52, 3.37) | 0.554 | 0.778 |
|  |  | Weighted median | 99 | 0.98 (0.75, 1.29) | 0.897 | 0.965 |
|  |  | Simple mode | 99 | 1.01 (0.45, 2.28) | 0.983 | 0.994 |
|  |  | Weighted mode | 99 | 0.85 (0.40, 1.84) | 0.684 | 0.847 |

**Note:** Abbreviations: ADHD, attention-deficit/hyperactivity disorder; AD, Anxiety disorder; ASD, autism spectrum disorder; CI, confidence interval; MDD, major depression disorder; OR, odds ratio; PTSD, post traumatic stress disorder; SNV, single-nucleotide variant

**Table S8.** Test on pleiotropy and heterogeneity for the selected SNVs

| **Exposure** | **Outcome** | **Pleiotropy** | | |  | **Heterogeneity (IVW)** | | |
| --- | --- | --- | --- | --- | --- | --- | --- | --- |
|  |  | **MR-Egger intercept** | **SE** | ***P-***value |  | ***Cochran’s Q*** | ***df*** | ***P-***value |
| Length of mobile phone use | ADHD | 0.018 | 0.014 | 0.204 |  | 49.928 | 25 | 2.18E-03 |
| Time spent using computer | ADHD | -0.007 | 0.008 | 0.431 |  | 127.114 | 71 | 4.90E-05 |
| Plays computer games | ADHD | -0.006 | 0.014 | 0.69 |  | 84.006 | 39 | 3.85E-05 |
| Time spent watching television | ADHD | 0.005 | 0.007 | 0.457 |  | 179.462 | 96 | 5.22E-07 |
| Length of mobile phone use | AD | 0.033 | 0.033 | 0.329 |  | 27.812 | 27 | 4.21E-01 |
| Time spent using computer | AD | -0.012 | 0.022 | 0.59 |  | 84.537 | 73 | 1.68E-01 |
| Plays computer games | AD | 0.098 | 0.028 | 0.001 |  | 56.438 | 45 | 1.18E-01 |
| Time spent watching television | AD | 0.021 | 0.017 | 0.21 |  | 86.011 | 96 | 7.58E-01 |
| Length of mobile phone use | ASD | -0.011 | 0.018 | 0.53 |  | 36.863 | 26 | 7.69E-02 |
| Time spent using computer | ASD | 0.001 | 0.012 | 0.967 |  | 134.464 | 74 | 2.20E-05 |
| Plays computer games | ASD | -0.021 | 0.016 | 0.189 |  | 74.933 | 46 | 4.48E-03 |
| Time spent watching television | ASD | 0.003 | 0.01 | 0.74 |  | 167.868 | 96 | 8.01E-06 |
| Length of mobile phone use | MDD | 0.002 | 0.014 | 0.879 |  | 54.457 | 26 | 8.89E-04 |
| Time spent using computer | MDD | -0.006 | 0.008 | 0.48 |  | 146.341 | 72 | 5.41E-07 |
| Plays computer games | MDD | 0.009 | 0.011 | 0.399 |  | 72.77 | 43 | 3.05E-03 |
| Time spent watching television | MDD | -0.003 | 0.005 | 0.555 |  | 138.241 | 98 | 4.65E-03 |
| Length of mobile phone use | PTSD | -0.001 | 0.026 | 0.981 |  | 51.543 | 25 | 1.37E-03 |
| Time spent using computer | PTSD | 0.011 | 0.011 | 0.319 |  | 92.301 | 74 | 7.36E-02 |
| Plays computer games | PTSD | 0.008 | 0.015 | 0.6 |  | 56.676 | 46 | 1.35E-01 |
| Time spent watching television | PTSD | -0.018 | 0.009 | 0.054 |  | 142.329 | 98 | 2.32E-03 |

**Note:** Abbreviations: ADHD, attention-deficit/hyperactivity disorder; AD, Anxiety disorder; ASD, autism spectrum disorder; IVW, the inverse variance weighted; MDD, major depression disorder; MR, Mendelian randomization; PTSD, post traumatic stress disorder; SE, standard error; SNV, single-nucleotide variant

| 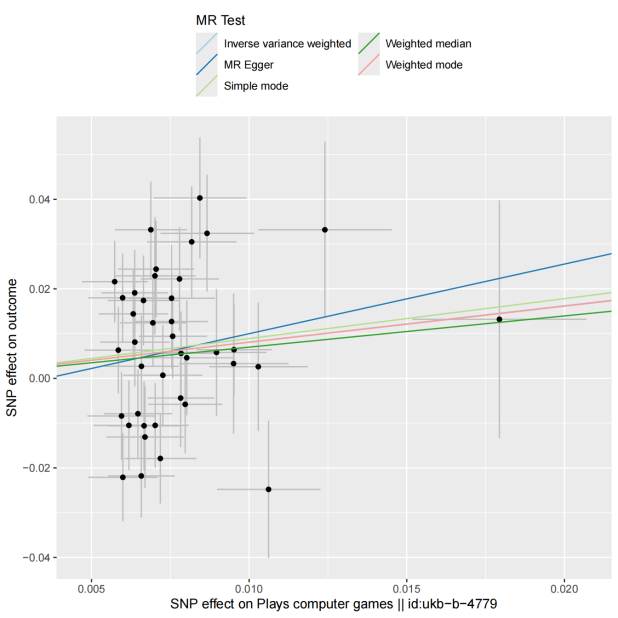  **B**  **A** | 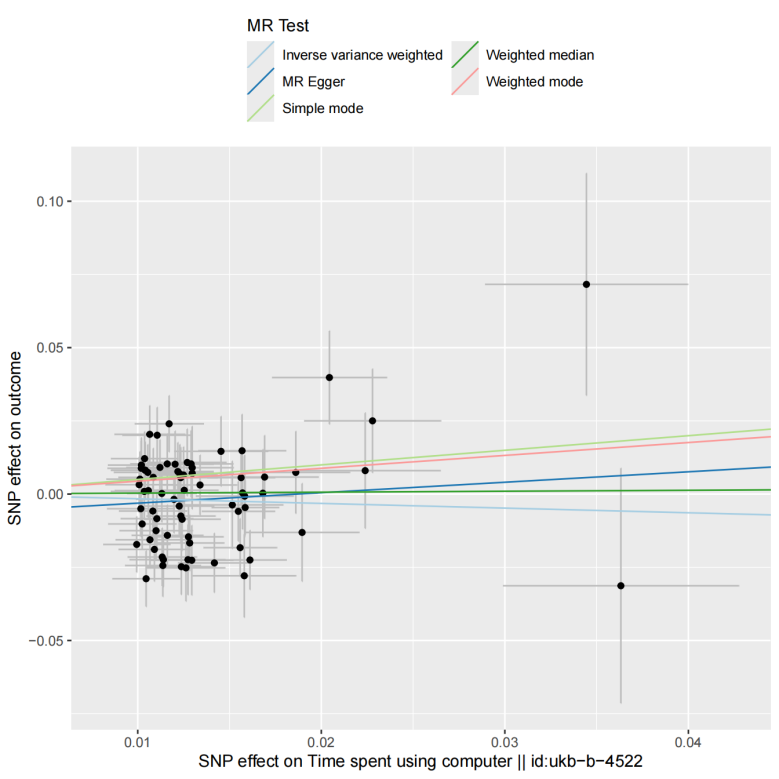  **D** |
| --- | --- |
| 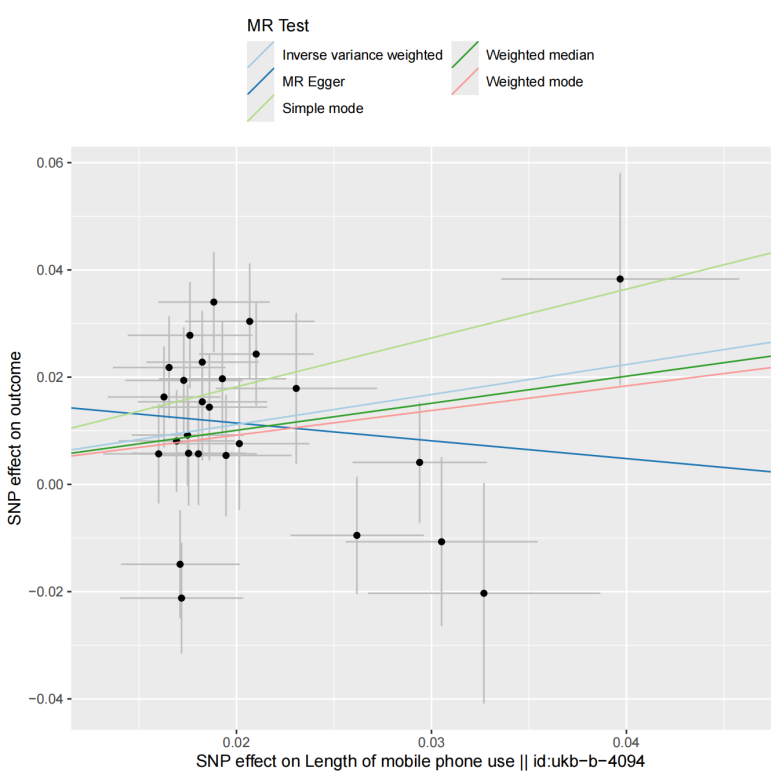  **C** | 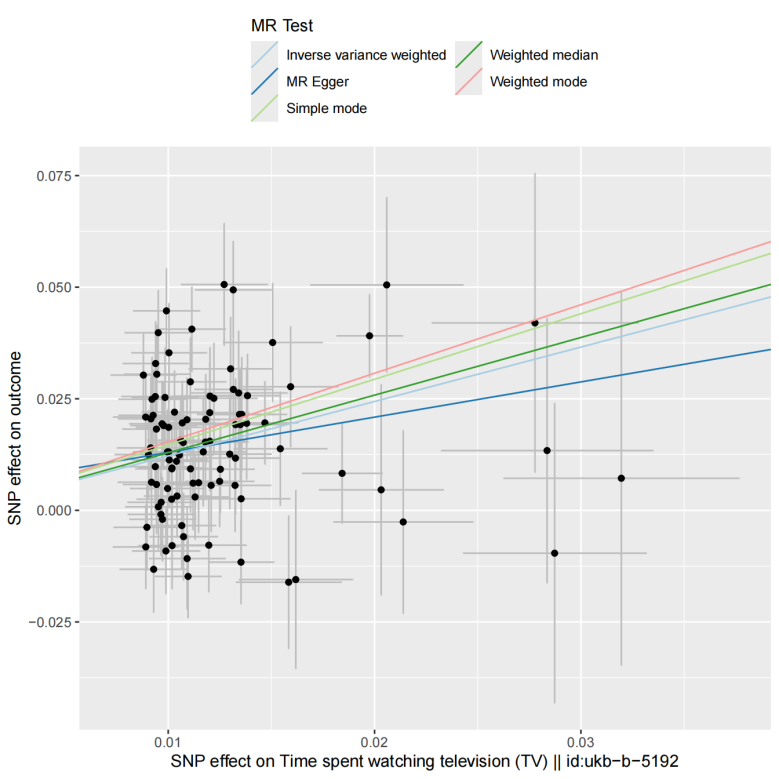 |

**Fig. S1.** Scatter plots of single SNVs included in the mendelian randomization study for the effects of digital device use on the risk of attention-deficit/hyperactivity disorder

**Note:** Each point in the scatter plot represents an SNV, and the slope of the regression line indicates the magnitude of the causal association using the five MR methods.

1. Length of mobile phone use, (B) Time spent using computer, (C) Plays computer games, and (D) Time spent watching television

Abbreviations: MR, Mendelian randomization; SNV, single-nucleotide variant

| 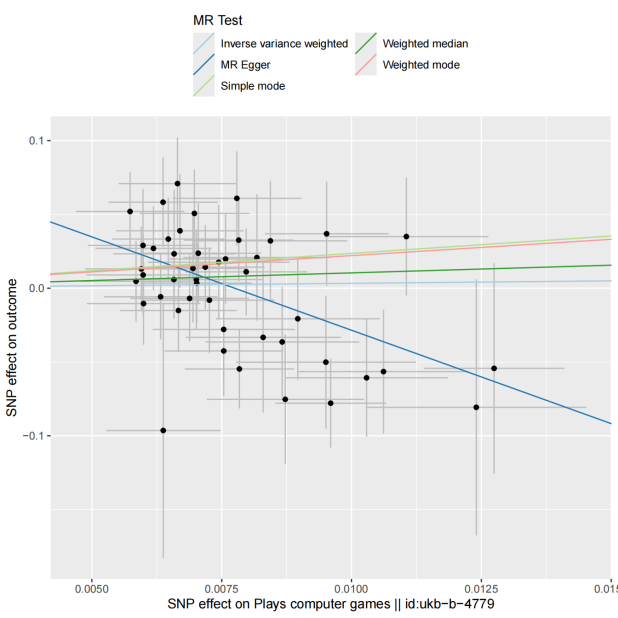  **B**  **A** | 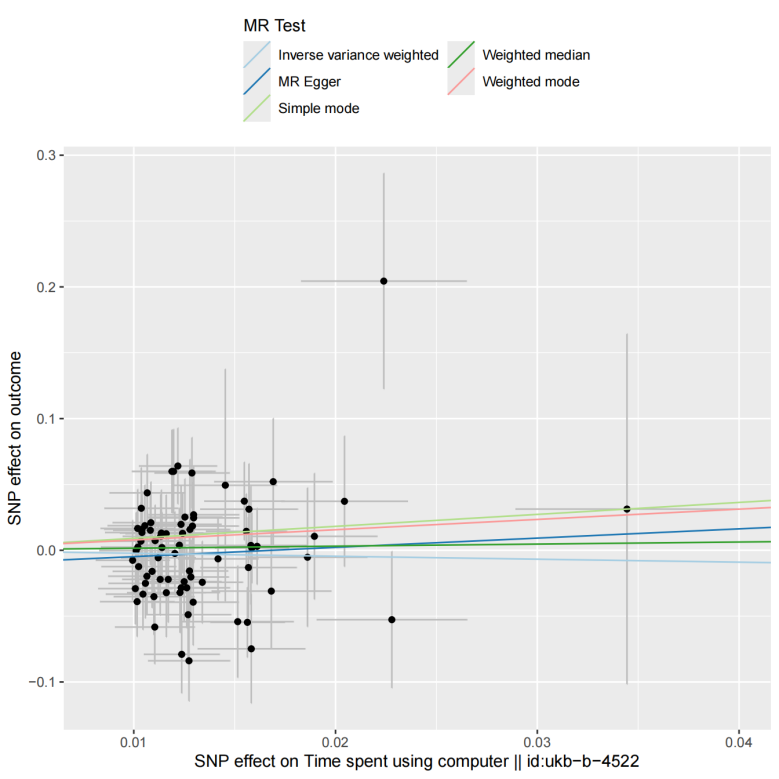  **D** |
| --- | --- |
| 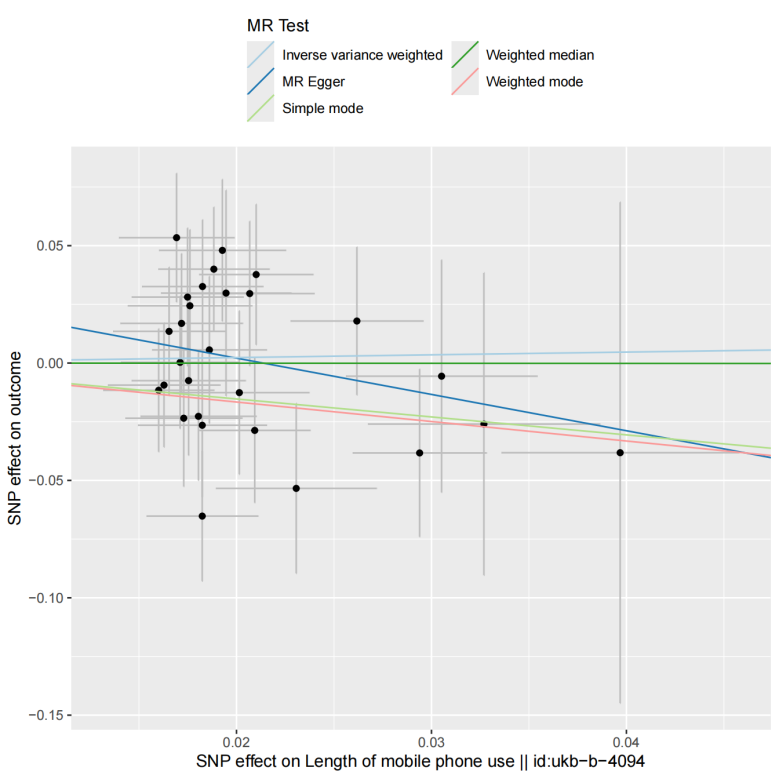  **C** | 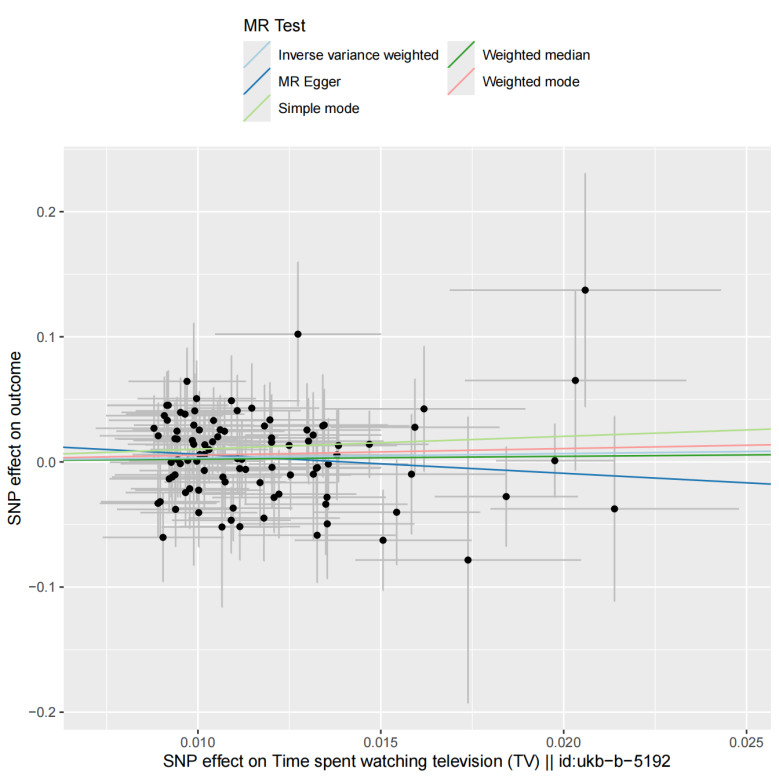 |

**Fig. S2.** Scatter plots of single SNVs included in the mendelian randomization study for the effects of digital device use on the risk of anxiety disorder

**Note:** Each point in the scatter plot represents an SNV, and the slope of the regression line indicates the magnitude of the causal association using the five MR methods.

(A) Length of mobile phone use, (B) Time spent using computer, (C) Plays computer games, and (D) Time spent watching television

Abbreviations: MR, Mendelian randomization; SNV, single-nucleotide variant

| 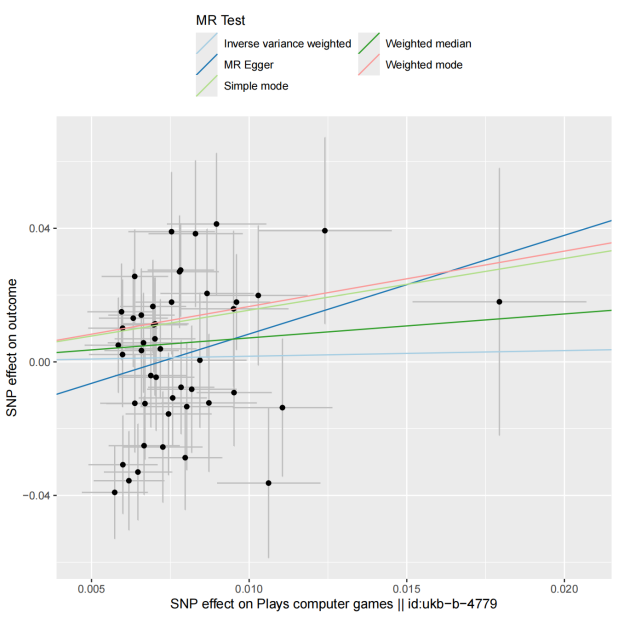  **A**  **B** | 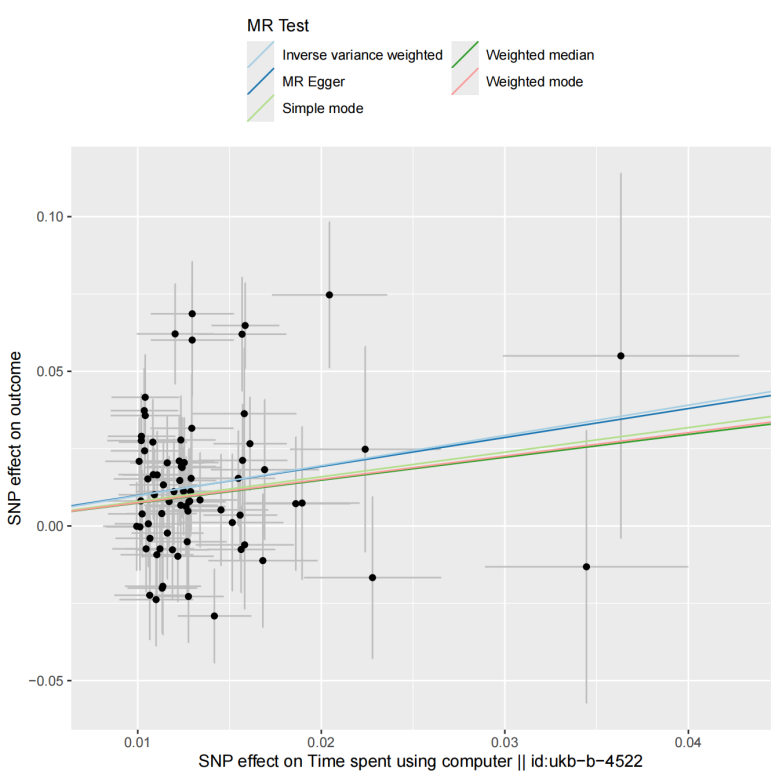  **D** |
| --- | --- |
| 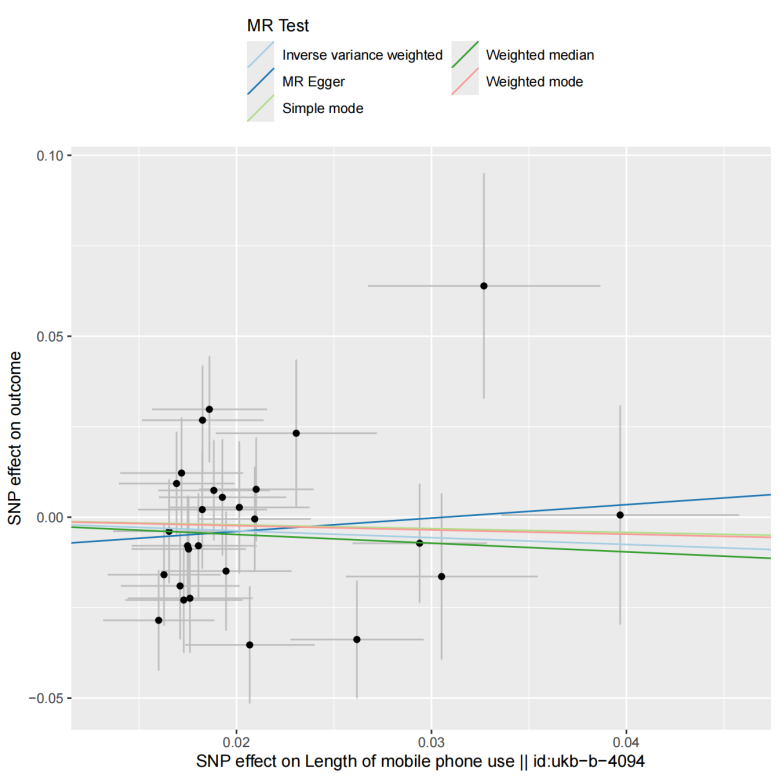  **C** | 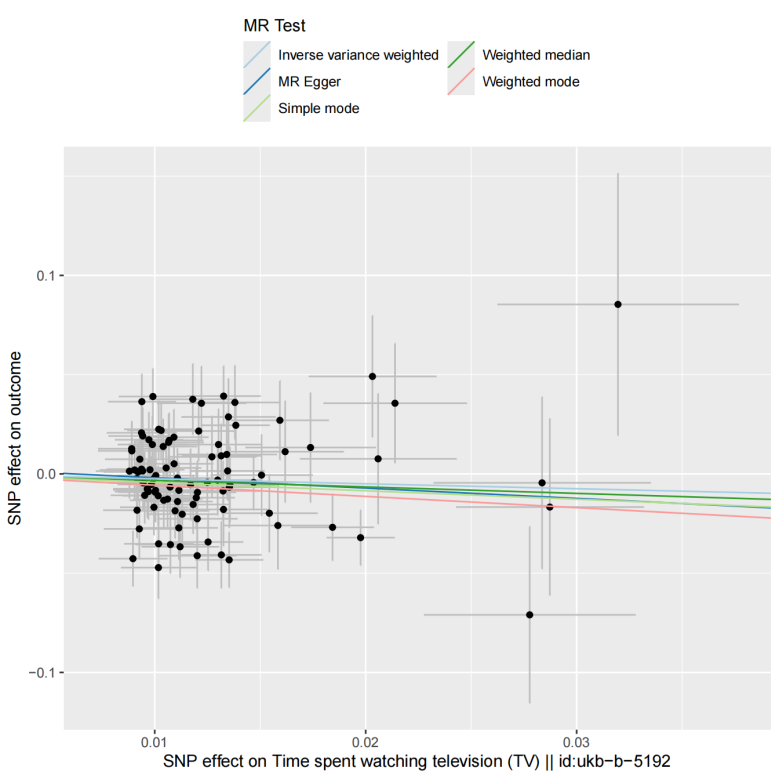 |

**Fig. S3.** Scatter plots of single SNVs included in the mendelian randomization study for the effects of digital device use on the risk of autism spectrum disorder

**Note:** Each point in the scatter plot represents an SNV, and the slope of the regression line indicates the magnitude of the causal association using the five MR methods.

(A) Length of mobile phone use, (B) Time spent using computer, (C) Plays computer games, and (D) Time spent watching television

Abbreviations: MR, Mendelian randomization; SNV, single-nucleotide variant

| 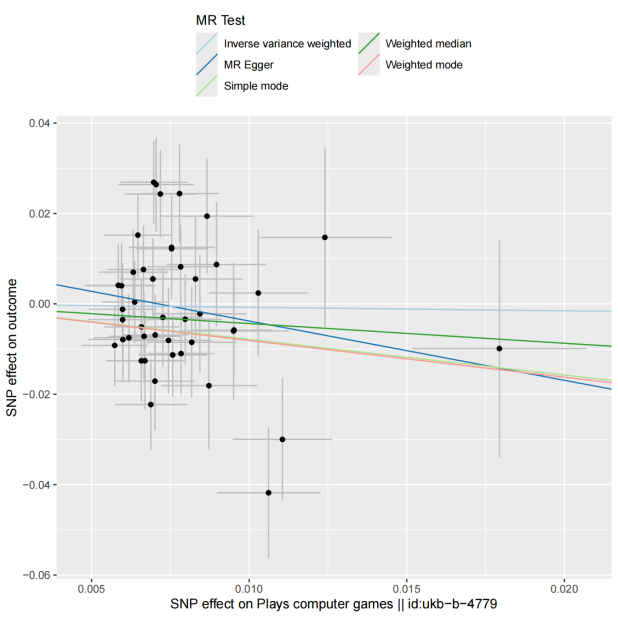  **D**  **B**  **A** | 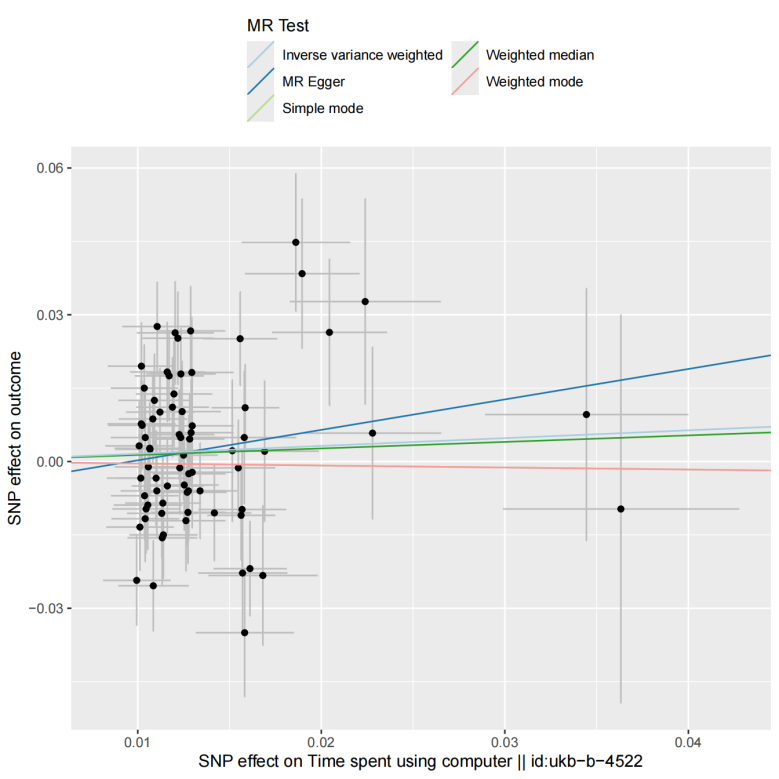 |
| --- | --- |
| 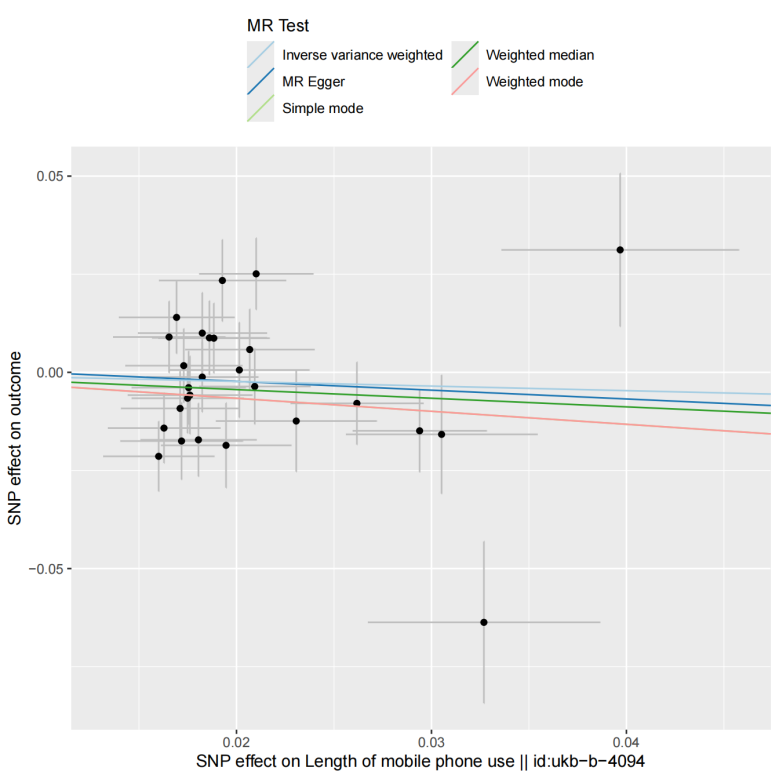  **C** | 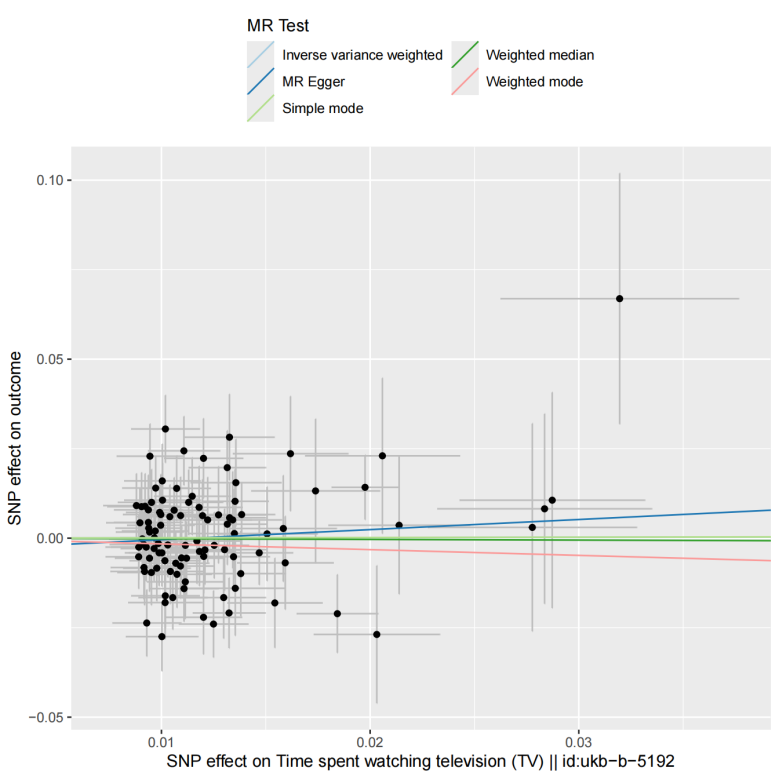 |

**Fig. S4.** Scatter plots of single SNVs included in the mendelian randomization study for the effects of digital device use on the risk of major depression disorder

**Note:** Each point in the scatter plot represents an SNV, and the slope of the regression line indicates the magnitude of the causal association using the five MR methods.

(A) Length of mobile phone use, (B) Time spent using computer, (C) Plays computer games, and (D) Time spent watching television

Abbreviations: MR, Mendelian randomization; SNV, single-nucleotide variant

| 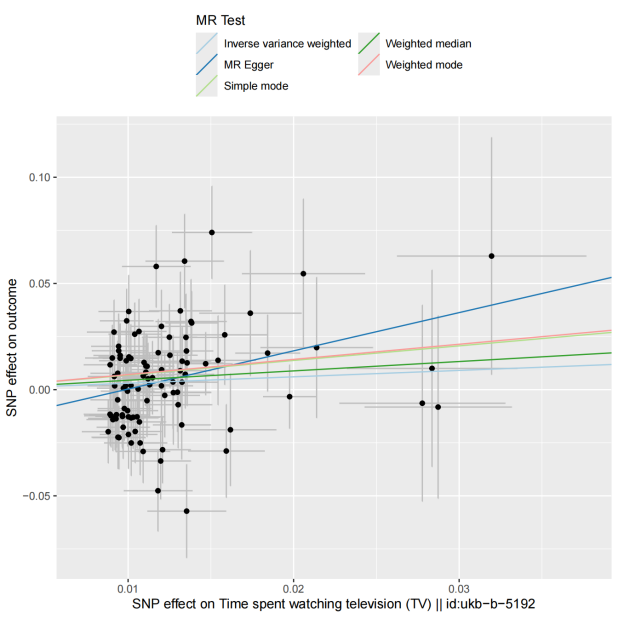  **D**  **A**  **B** | 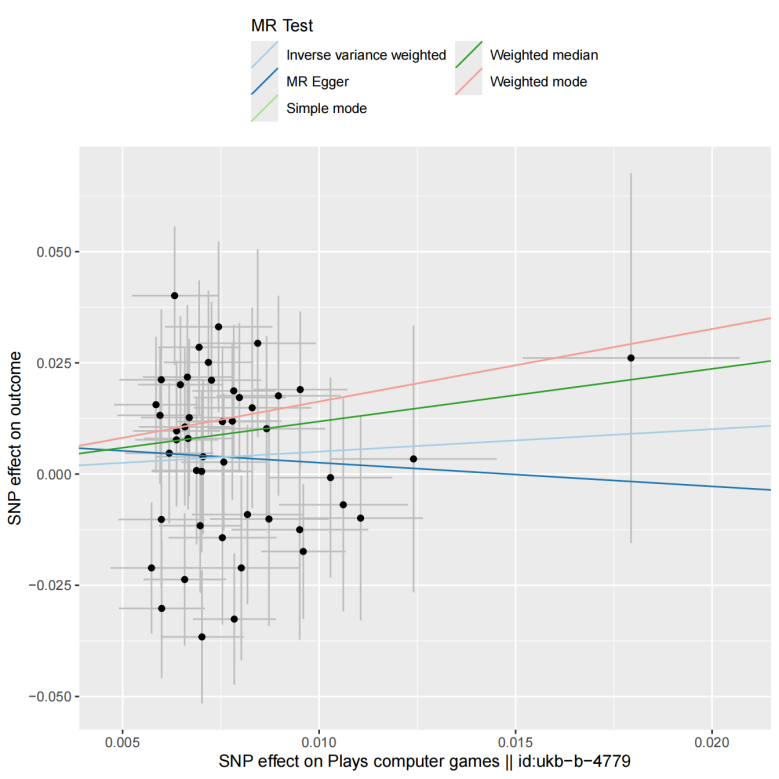 |
| --- | --- |
| 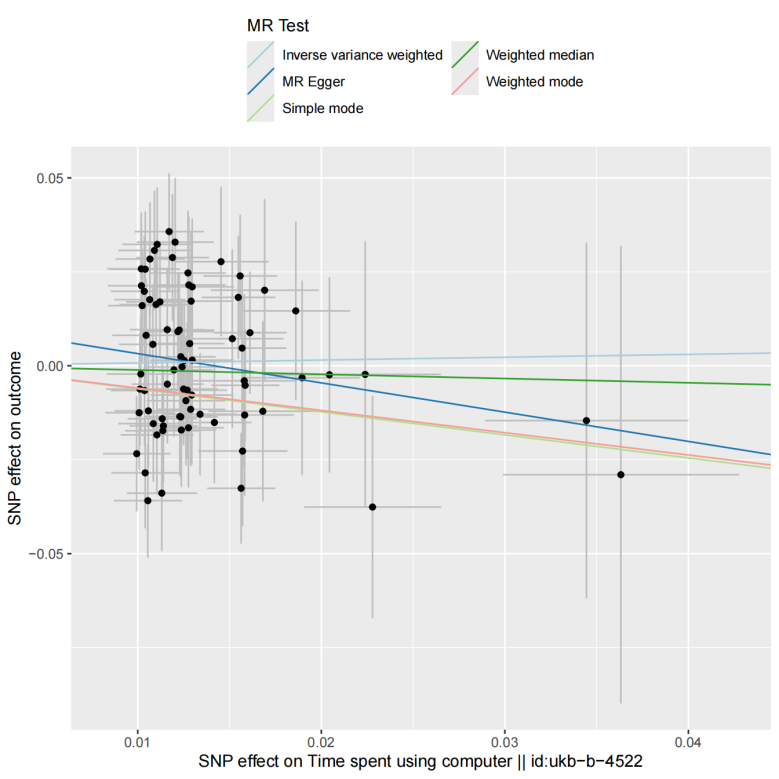  **C** | 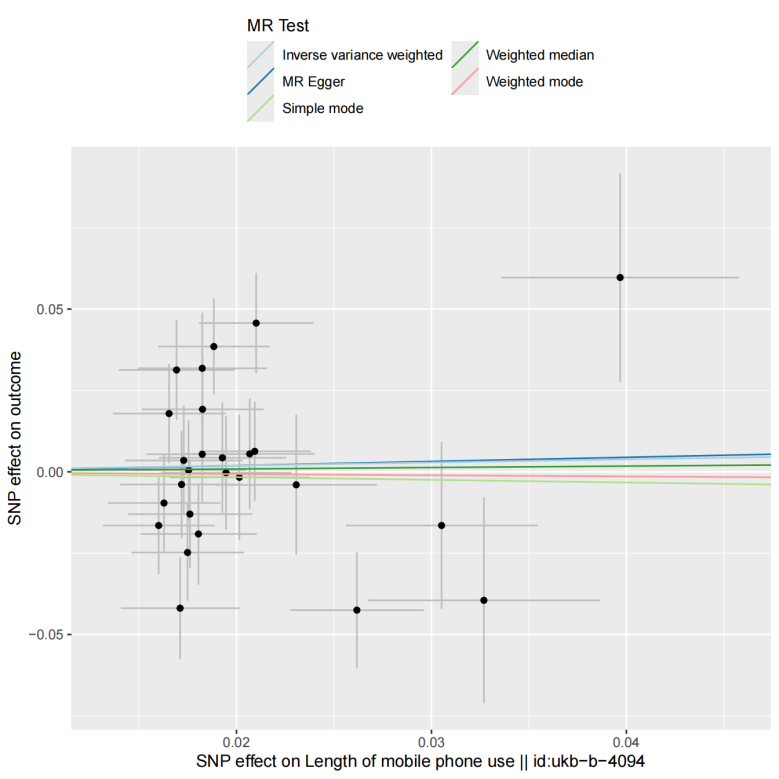 |

**Fig. S5.** Scatter plots of single SNVs included in the mendelian randomization study for the effects of digital device use on the risk of post traumatic stress disorder

**Note:** Each point in the scatter plot represents an SNV, and the slope of the regression line indicates the magnitude of the causal association using the five MR methods.

(A) Length of mobile phone use, (B) Time spent using computer, (C) Plays computer games, and (D) Time spent watching television

Abbreviations: MR, Mendelian randomization; SNV, single-nucleotide variant

| 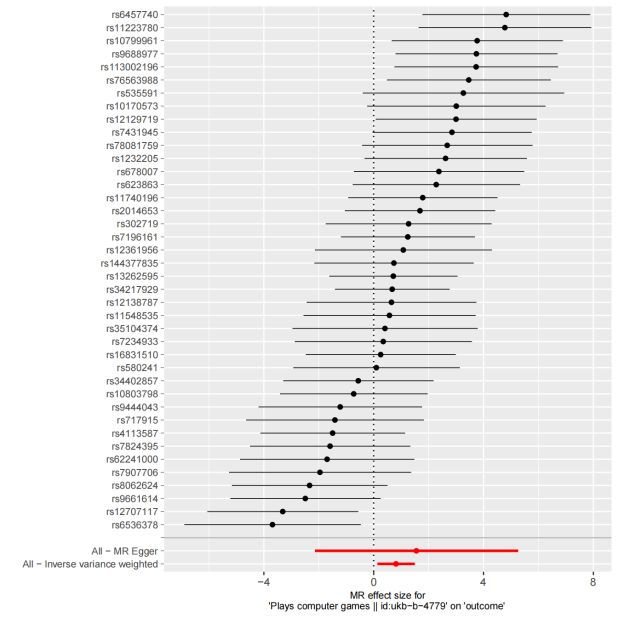  **A**  **B** | 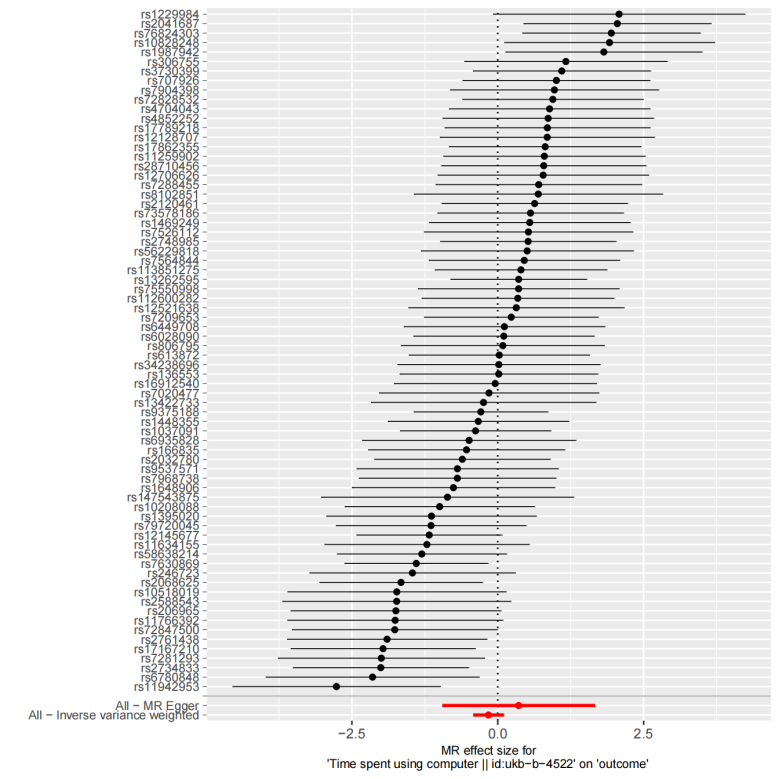 |
| --- | --- |
| 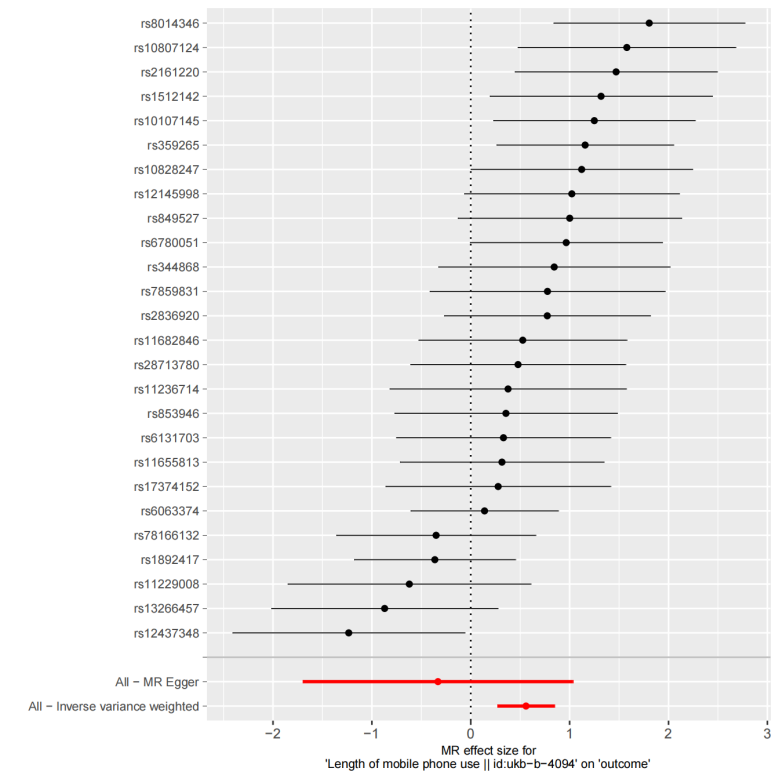  **C** | 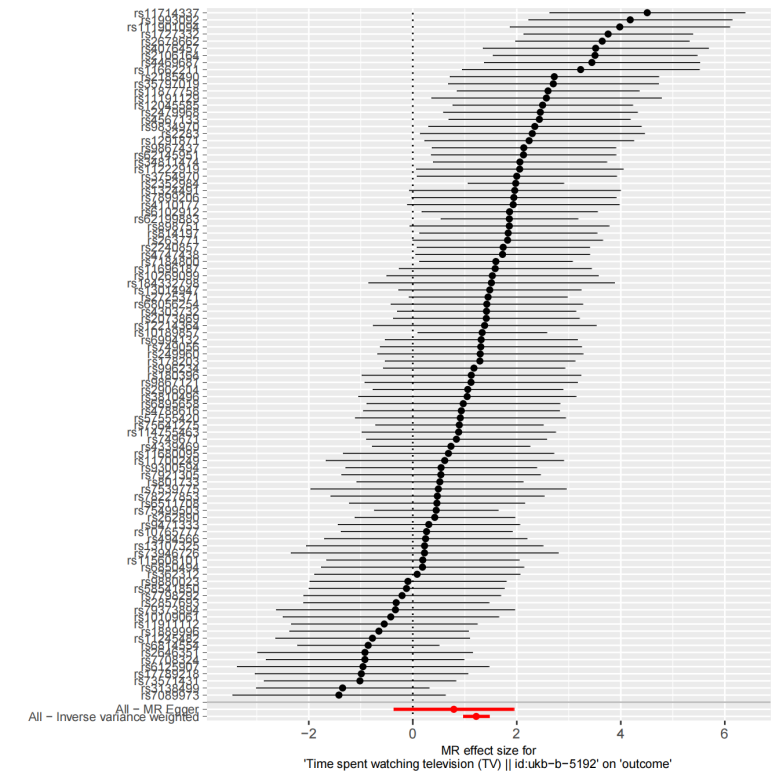  **D** |

**Fig. S6.** Forest plots for the effects of digital device use on the risk of attention-deficit/hyperactivity disorder

(A) Length of mobile phone use, (B) Time spent using computer, (C) Plays computer games, and (D) Time spent watching television

| 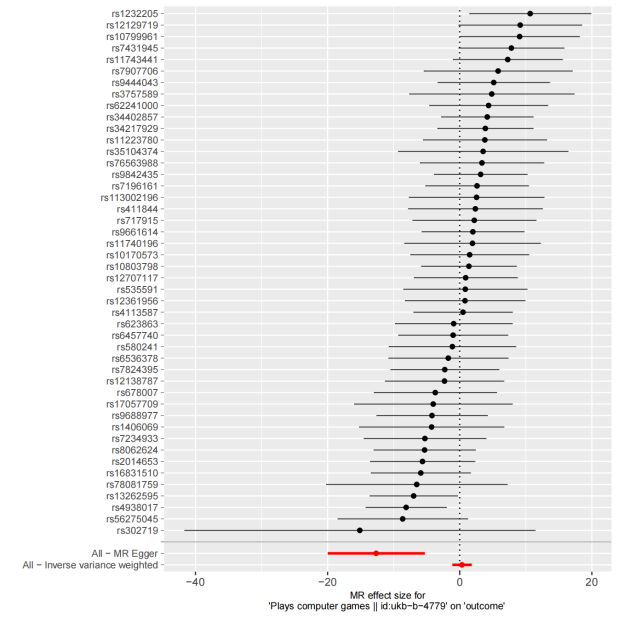  **A** | 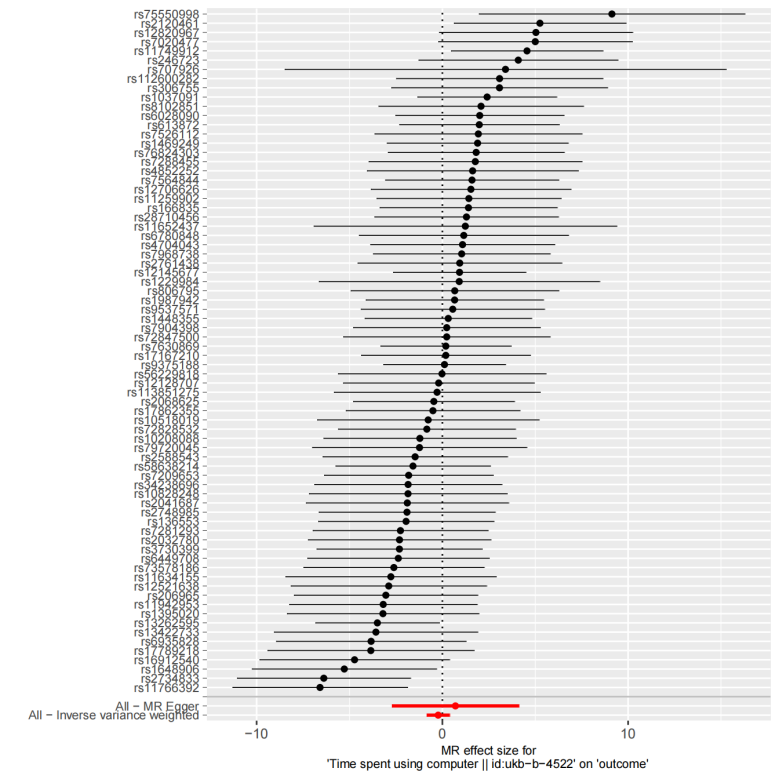  **B** |
| --- | --- |
| 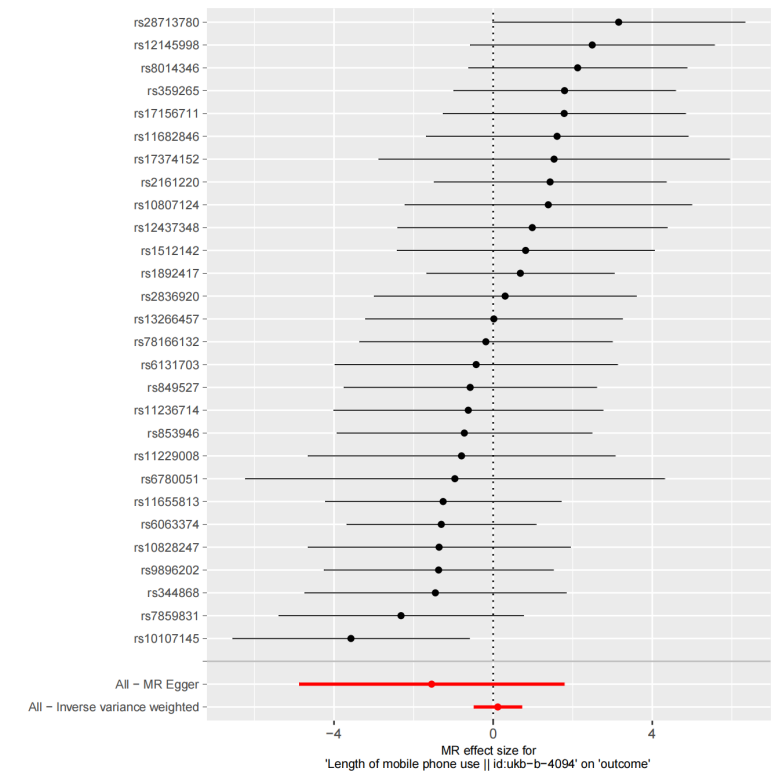  **C** | 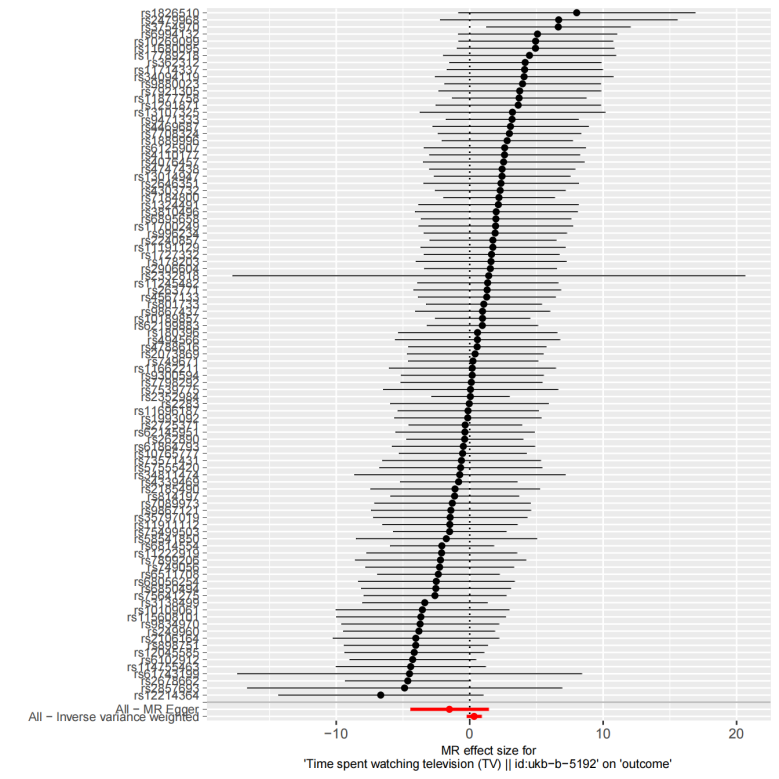  **D** |

**Fig. S7.** Forest plots for the effects of digital device use on the risk of anxiety disorder

(A) Length of mobile phone use, (B) Time spent using computer, (C) Plays computer games, and (D) Time spent watching television

| 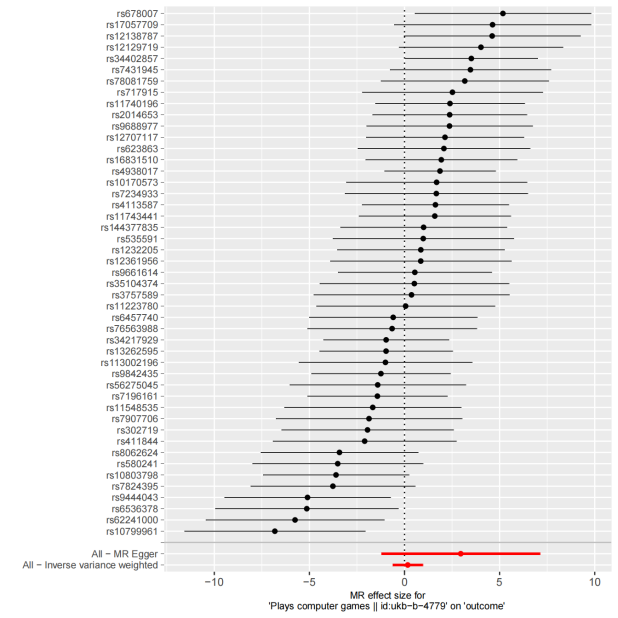  **A** | 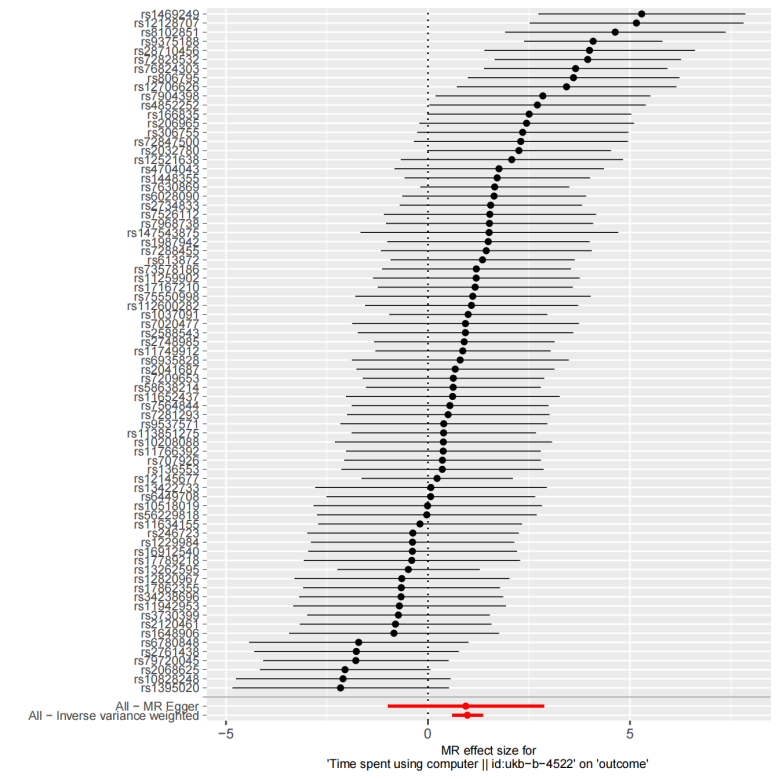  **B** |
| --- | --- |
| 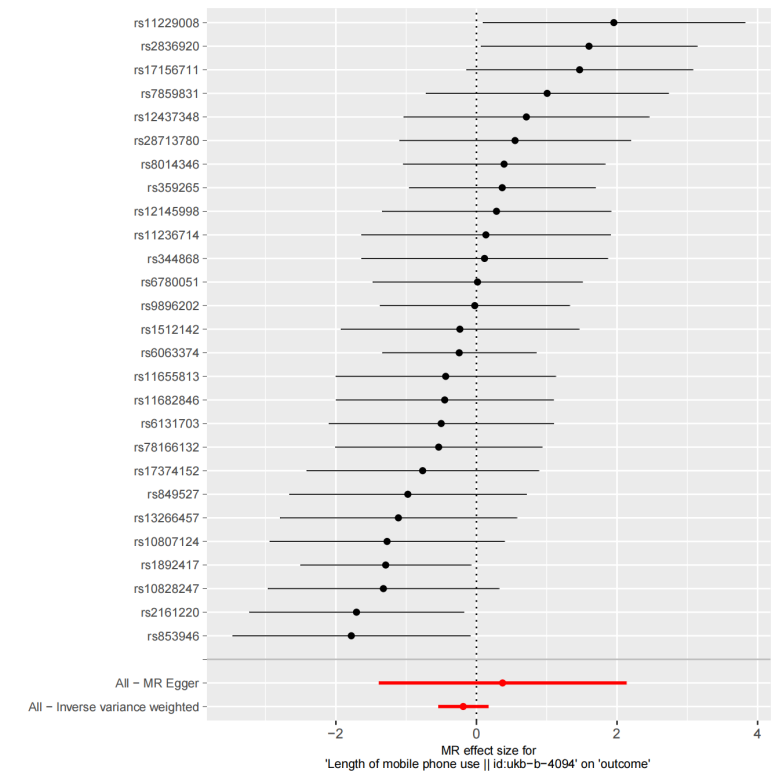  **C** | 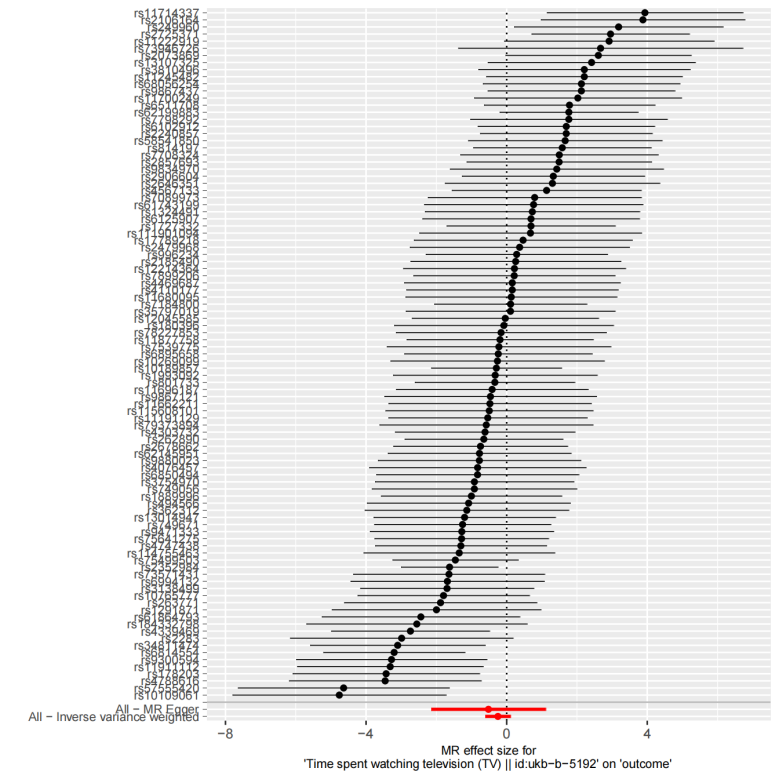  **D** |

**Fig. S8.** Forest plots for the effects of digital device use on the risk of autism spectrum disorder

(A) Length of mobile phone use, (B) Time spent using computer, (C) Plays computer games, and (D) Time spent watching television

| 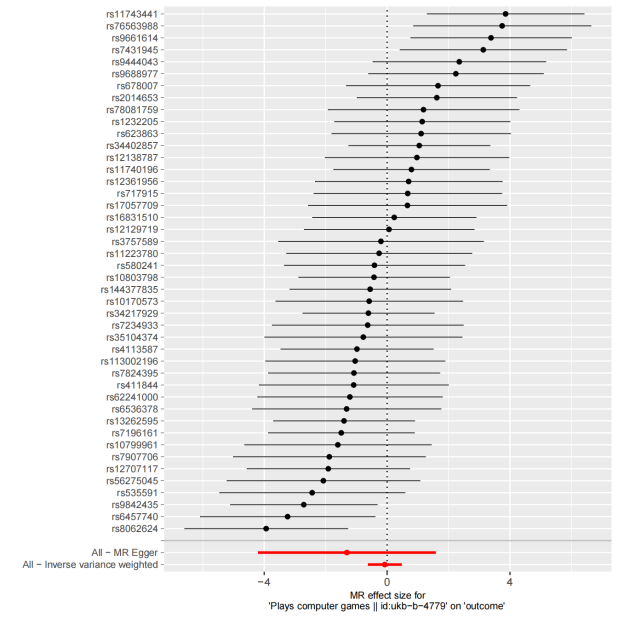  **A** | 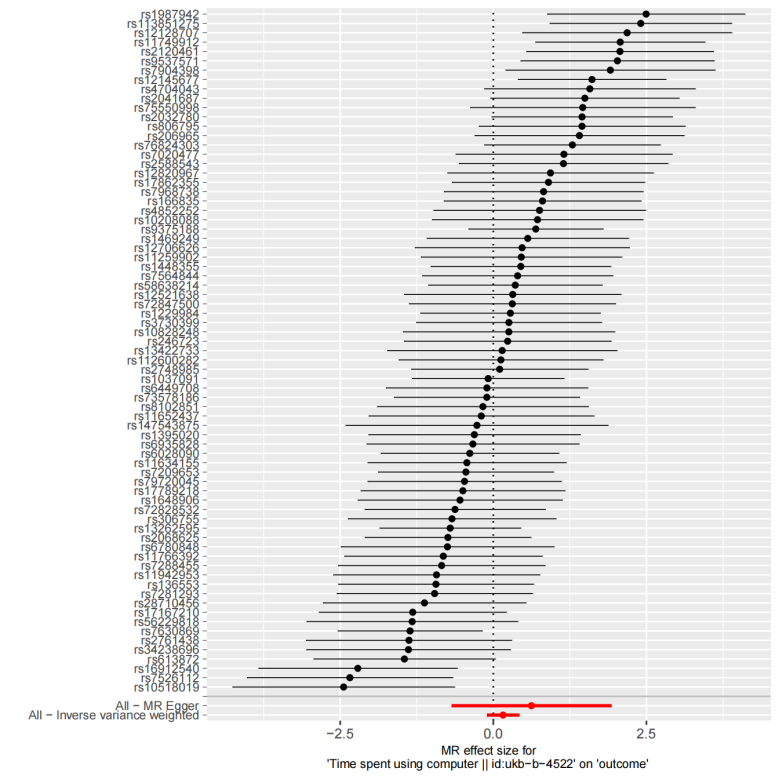  **B** |
| --- | --- |
| 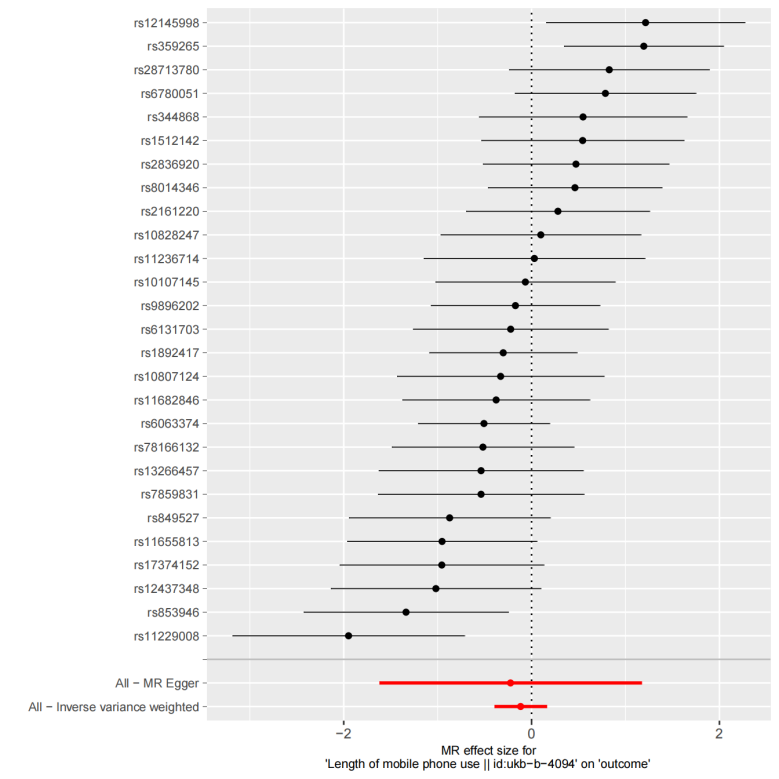  **C** | 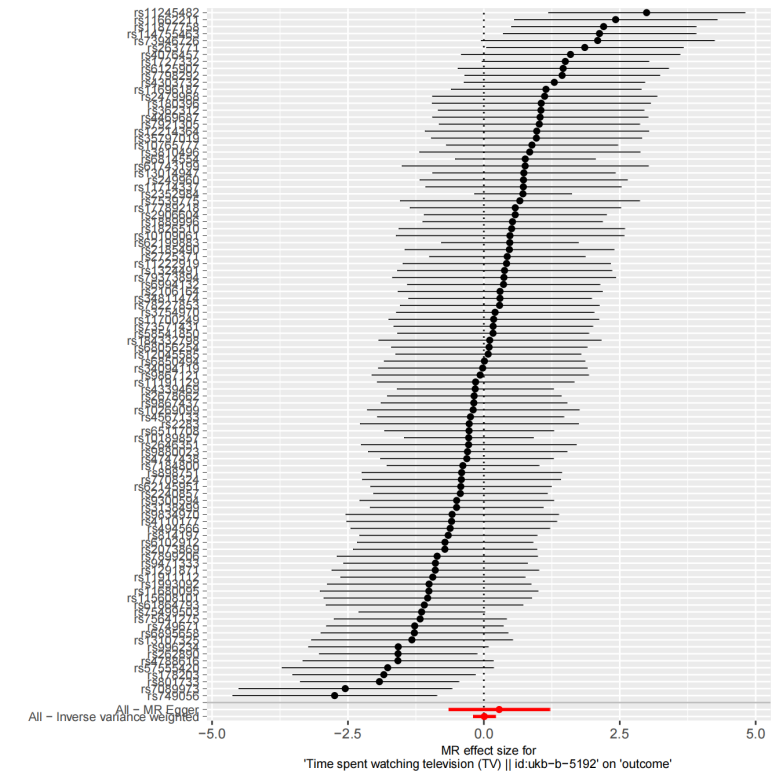  **D** |

**Fig. S9.** Forest plots for the effects of digital device use on the risk of major depression disorder

(A) Length of mobile phone use, (B) Time spent using computer, (C) Plays computer games, and (D) Time spent watching television

| 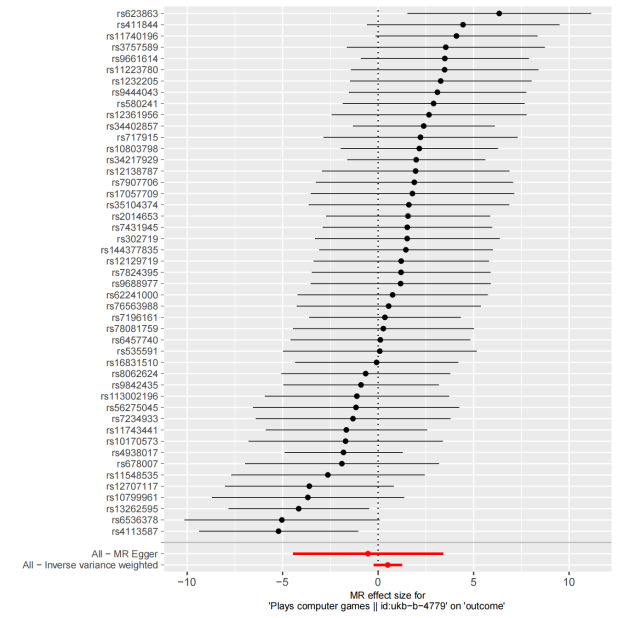  **A** | 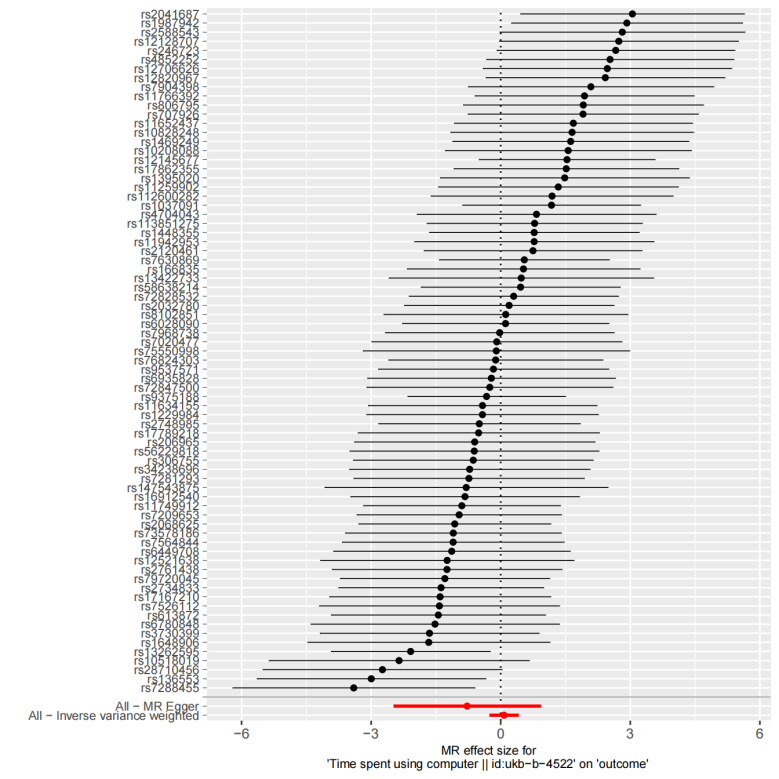  **B** |
| --- | --- |
| 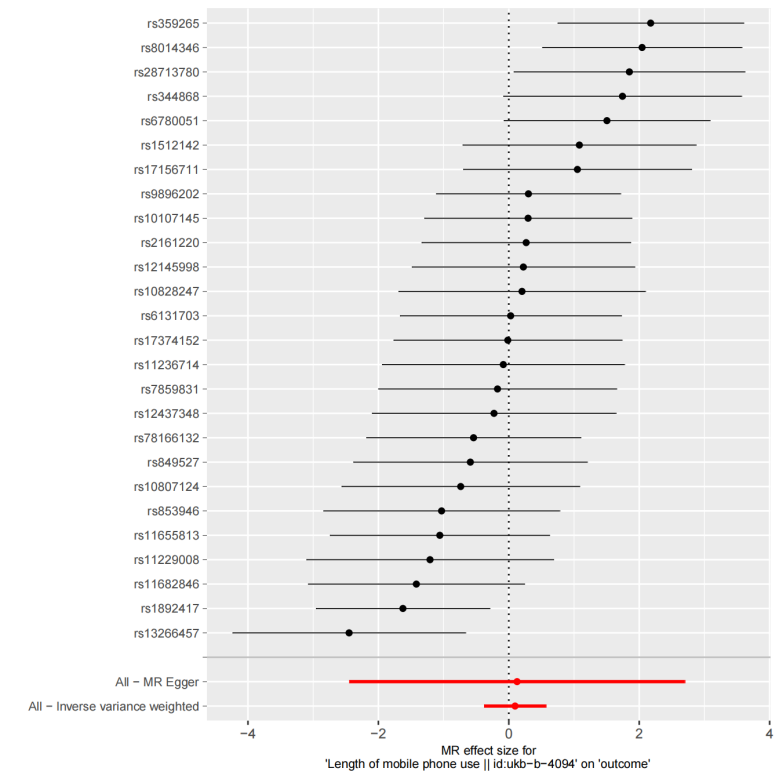  **C** | 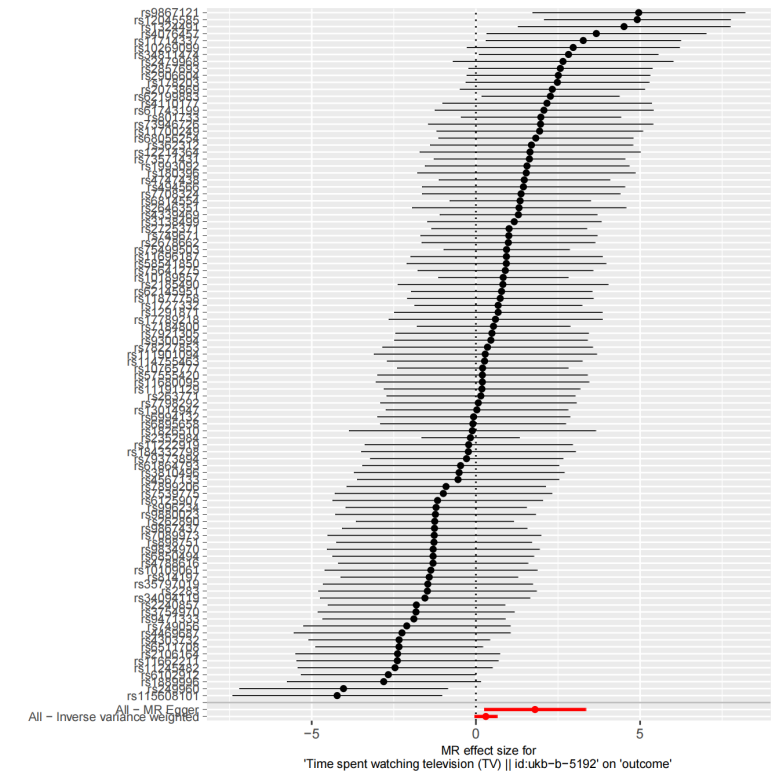  **D** |

**Fig. S10.** Forest plots for the effects of digital device use on the risk of post traumatic stress disorder

(A) Length of mobile phone use, (B) Time spent using computer, (C) Plays computer games, and (D) Time spent watching television

| 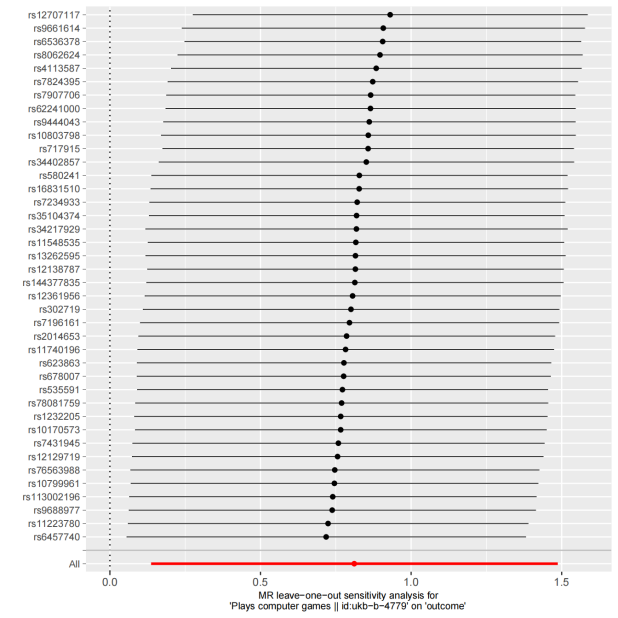  **A**  **B** | 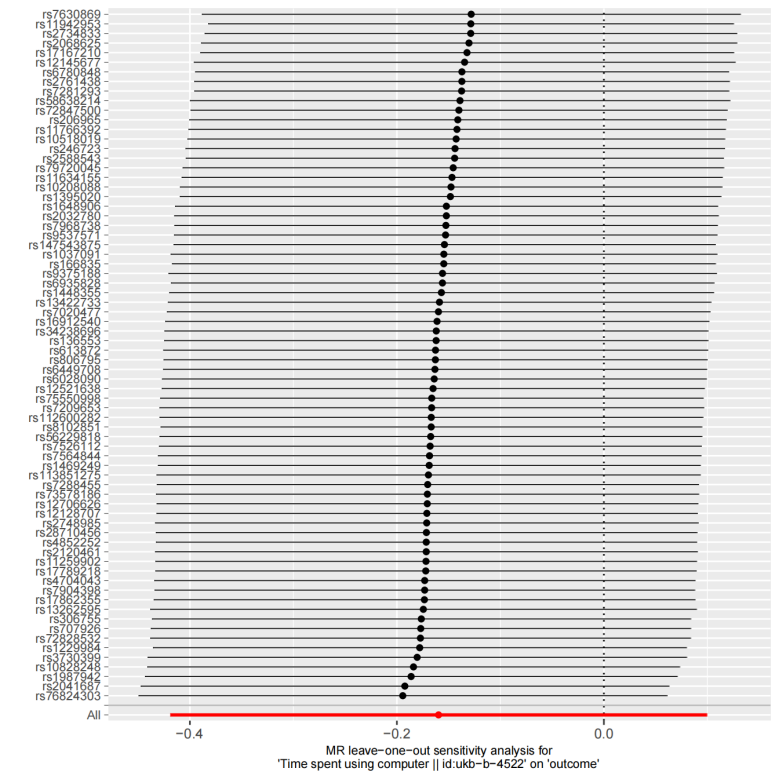 |
| --- | --- |
| 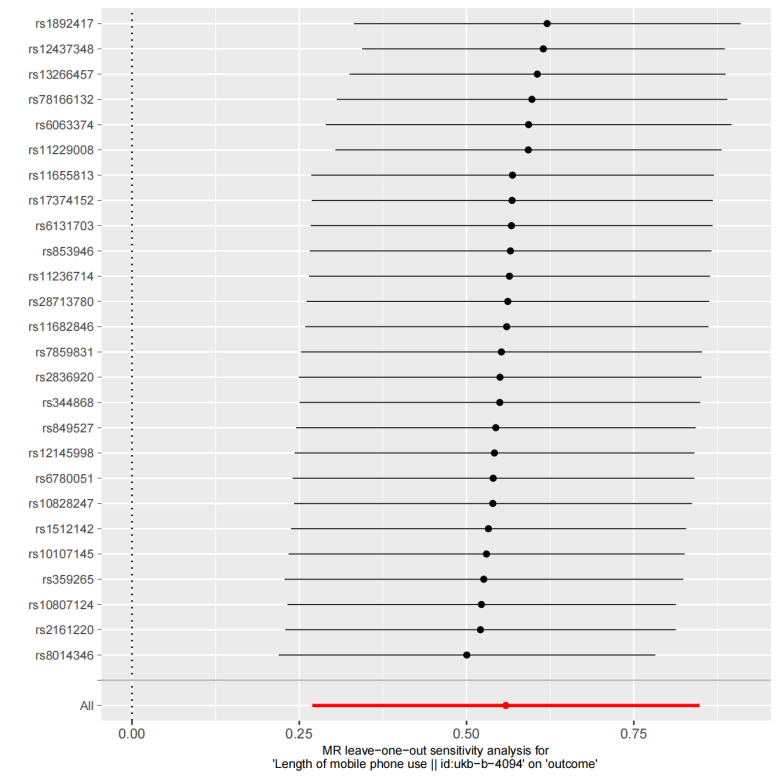  **D**  **C** | 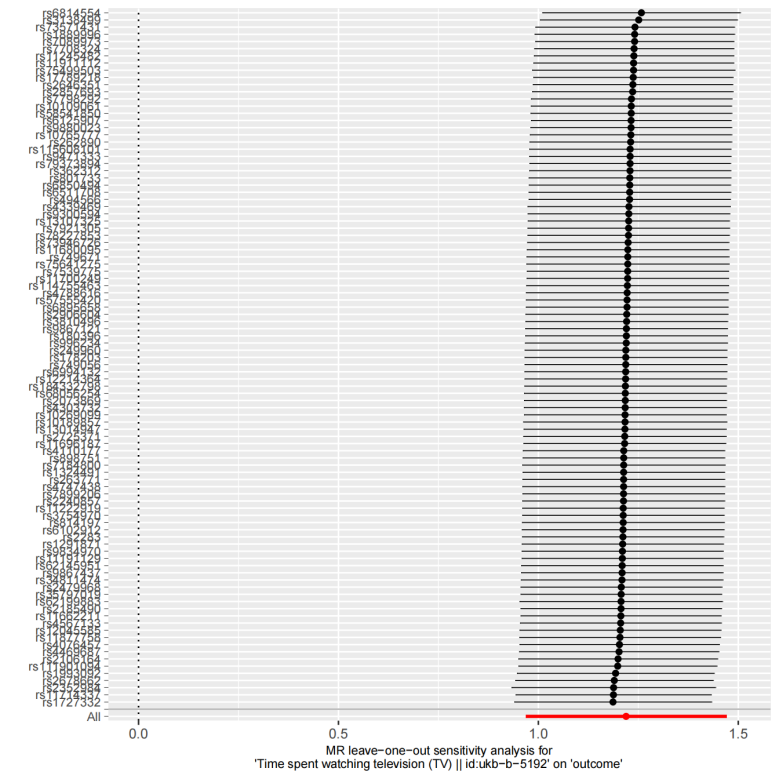 |

**Fig. S11.** Leave-one-out plot for the effects of digital device use on the risk of attention-deficit/hyperactivity disorder

(A) Length of mobile phone use, (B) Time spent using computer, (C) Plays computer games, and (D) Time spent watching television

| 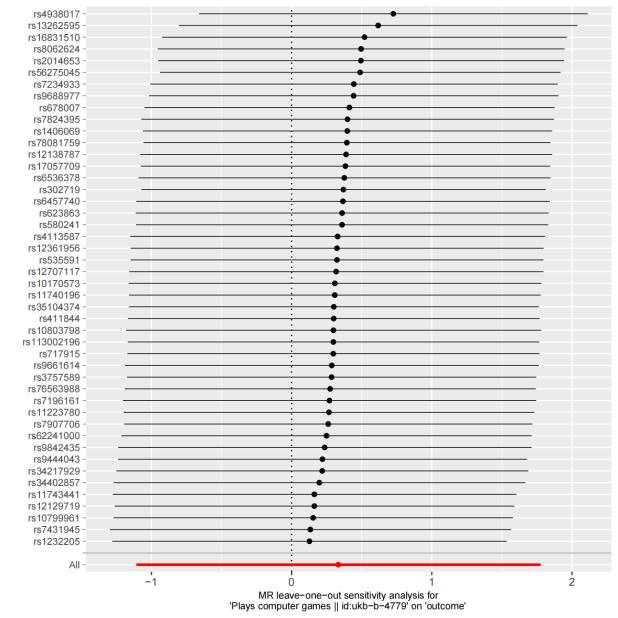  **D**  **B**  **A** | 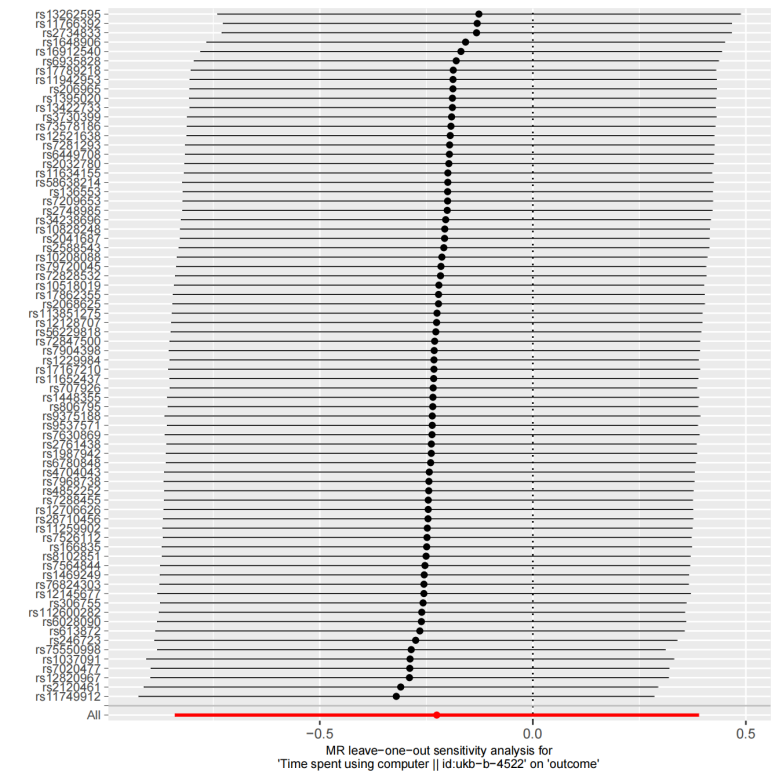 |
| --- | --- |
| 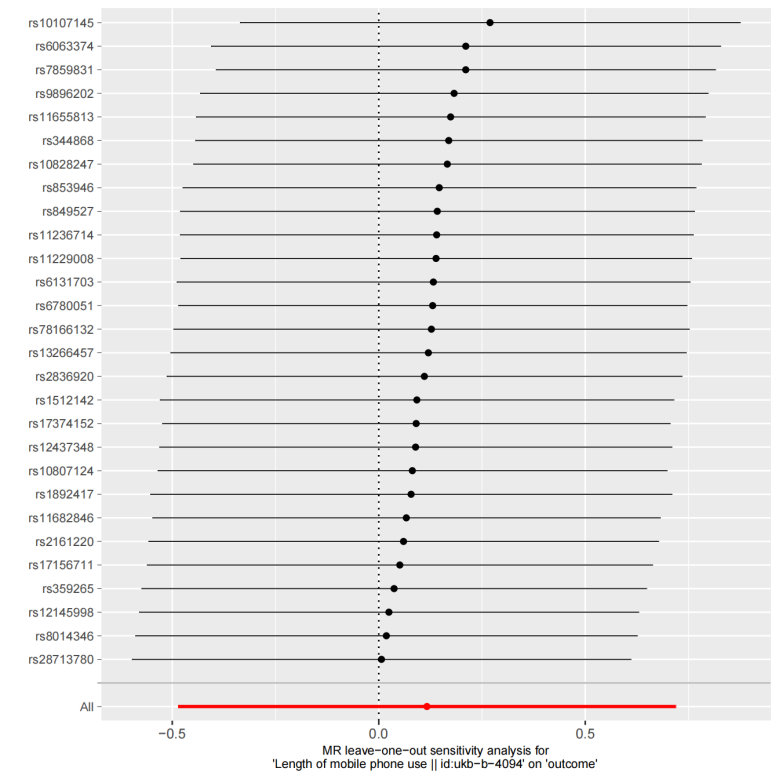  **C** | 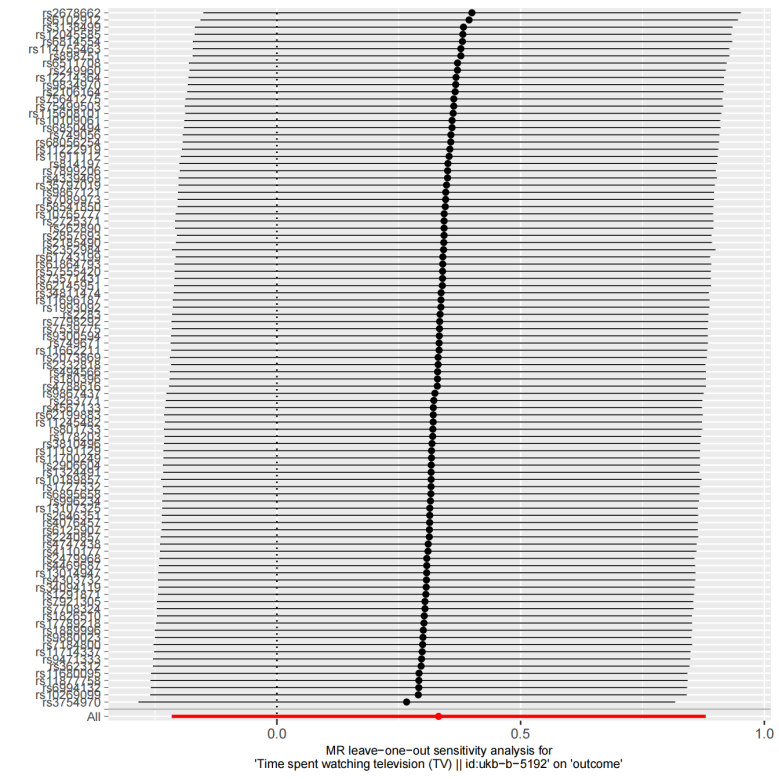 |

**Fig. S12.** Leave-one-out plot for the effects of digital device use on the risk of anxiety disorder

(A) Length of mobile phone use, (B) Time spent using computer, (C) Plays computer games, and (D) Time spent watching television

| 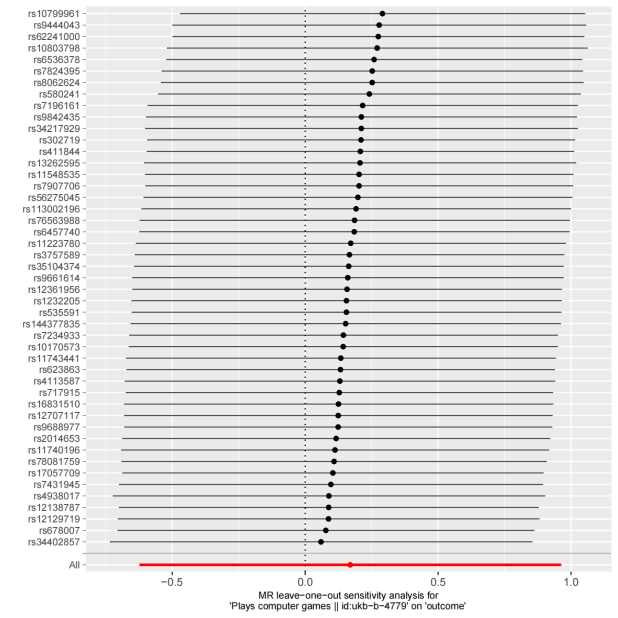  **D**  **B**  **A** | 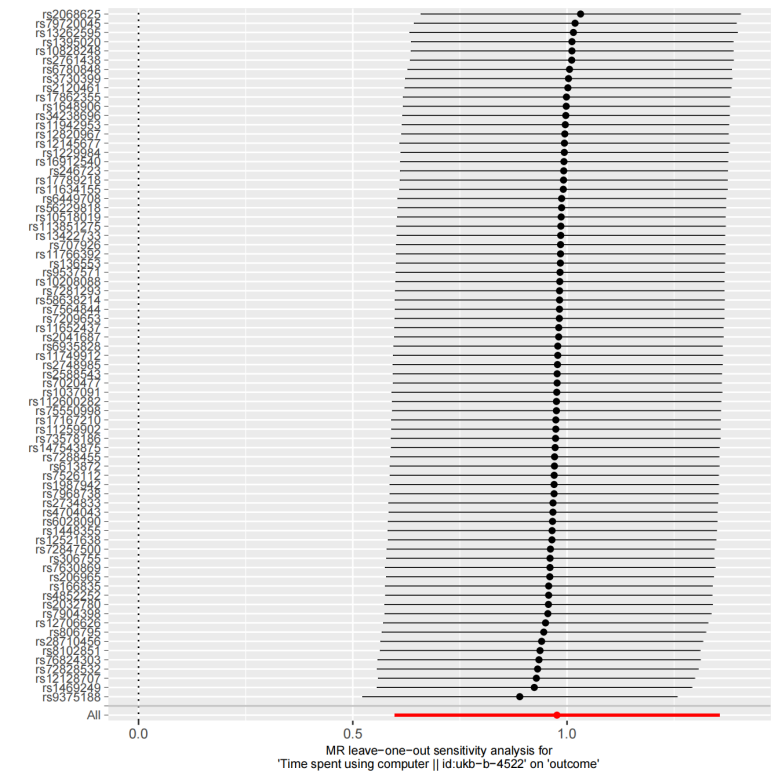 |
| --- | --- |
| 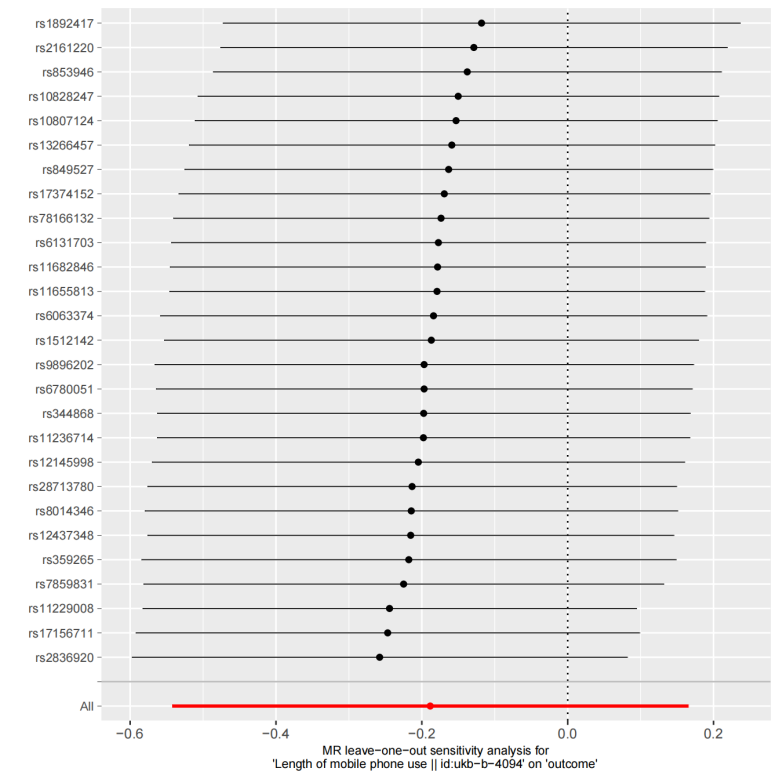  **C** | 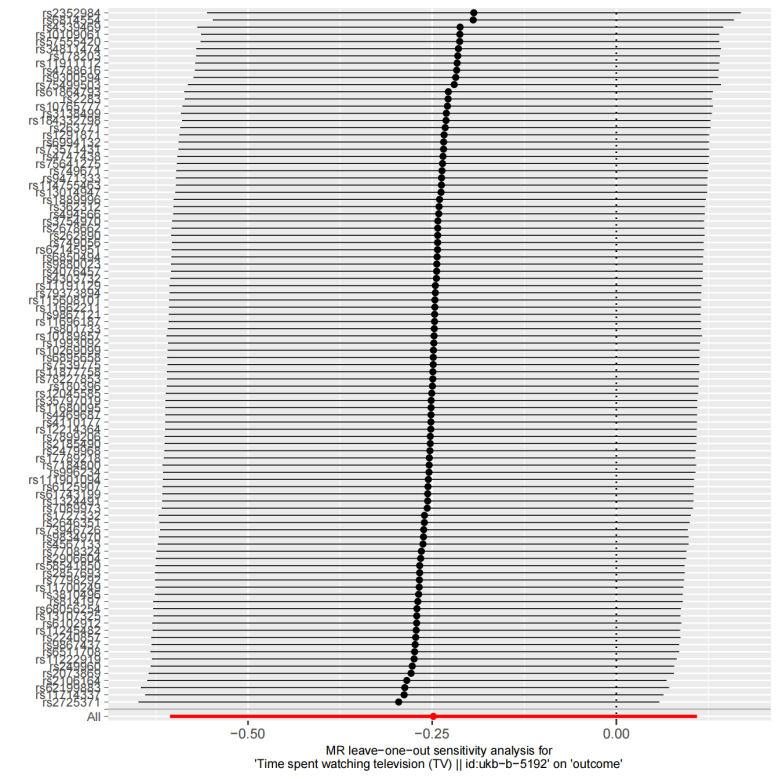 |

**Fig. S13.** Leave-one-out plot for the effects of digital device use on the risk of autism spectrum disorder

(A) Length of mobile phone use, (B) Time spent using computer, (C) Plays computer games, and (D) Time spent watching television

| 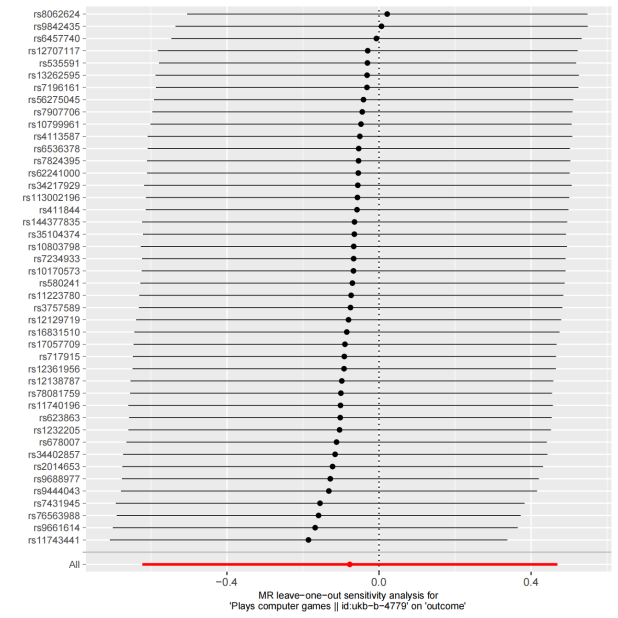  **D**  **B**  **A** | 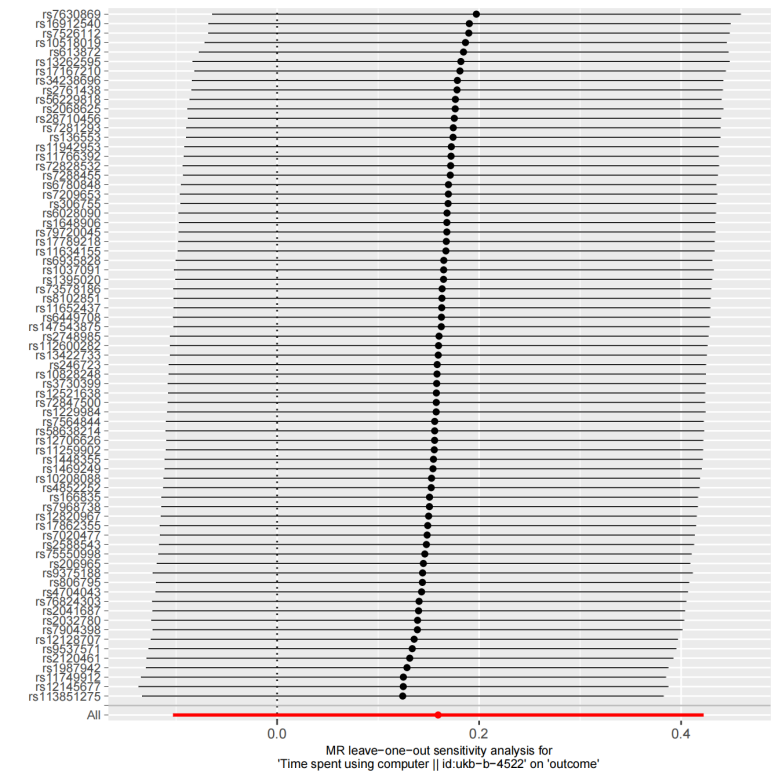 |
| --- | --- |
| 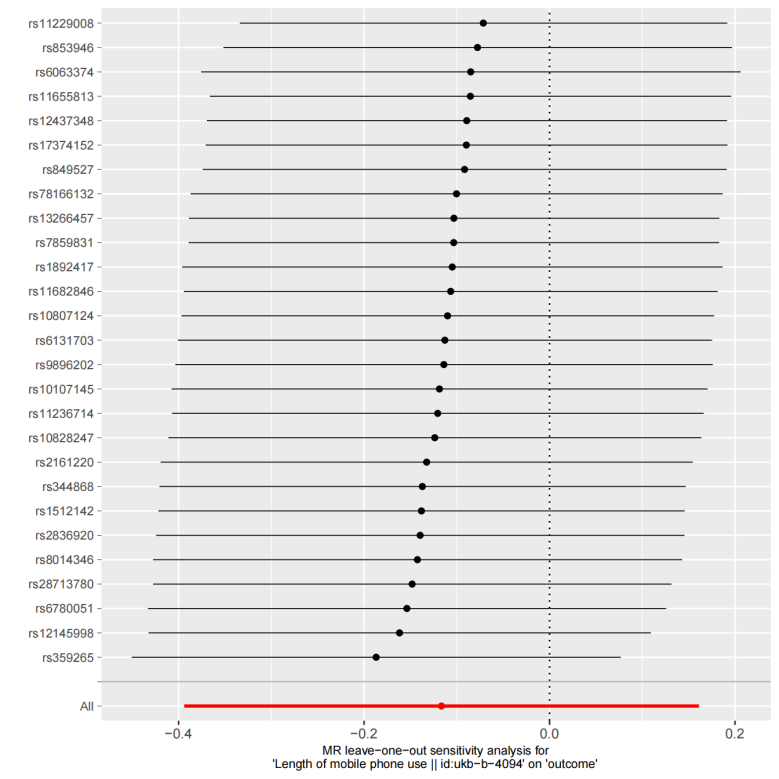  **C** | 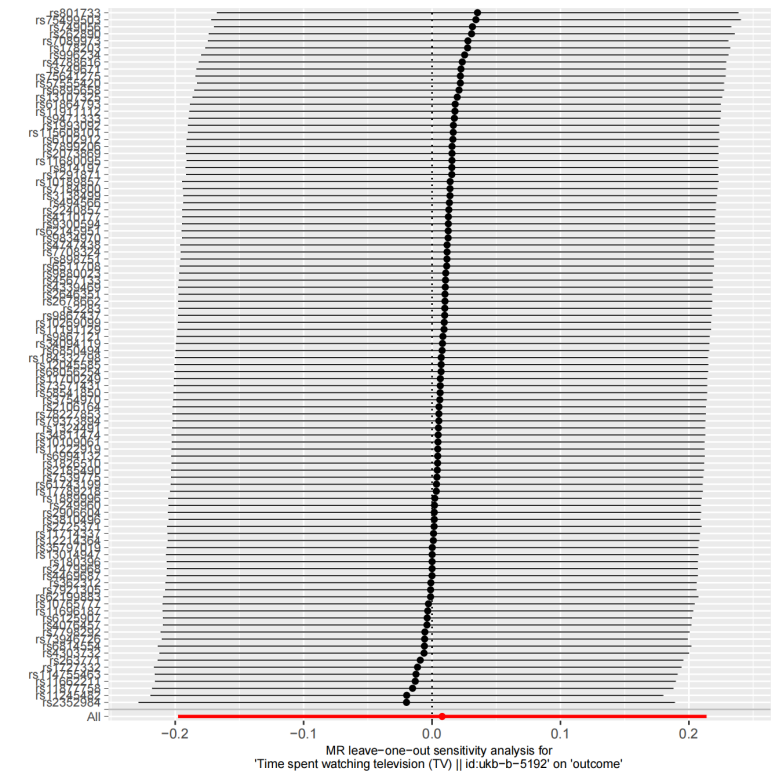 |

**Fig. S14.** Leave-one-out plot for the effects of digital device use on the risk of major depression disorder

(A) Length of mobile phone use, (B) Time spent using computer, (C) Plays computer games, and (D) Time spent watching television

| 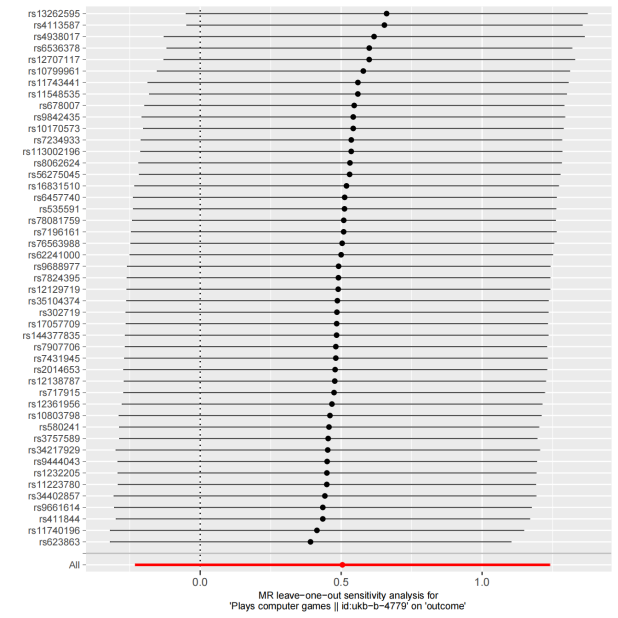  **D**  **B**  **A** | 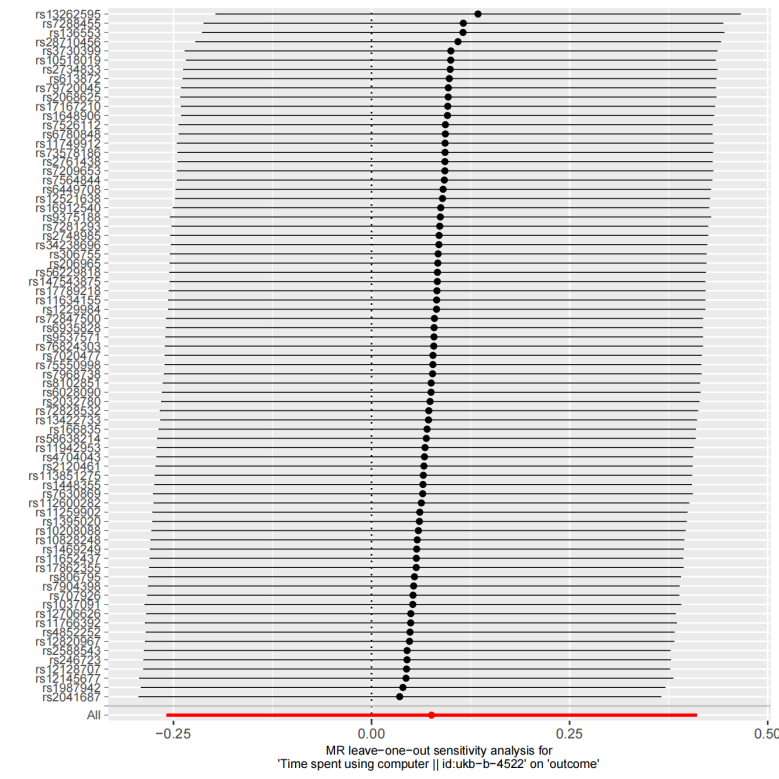 |
| --- | --- |
| 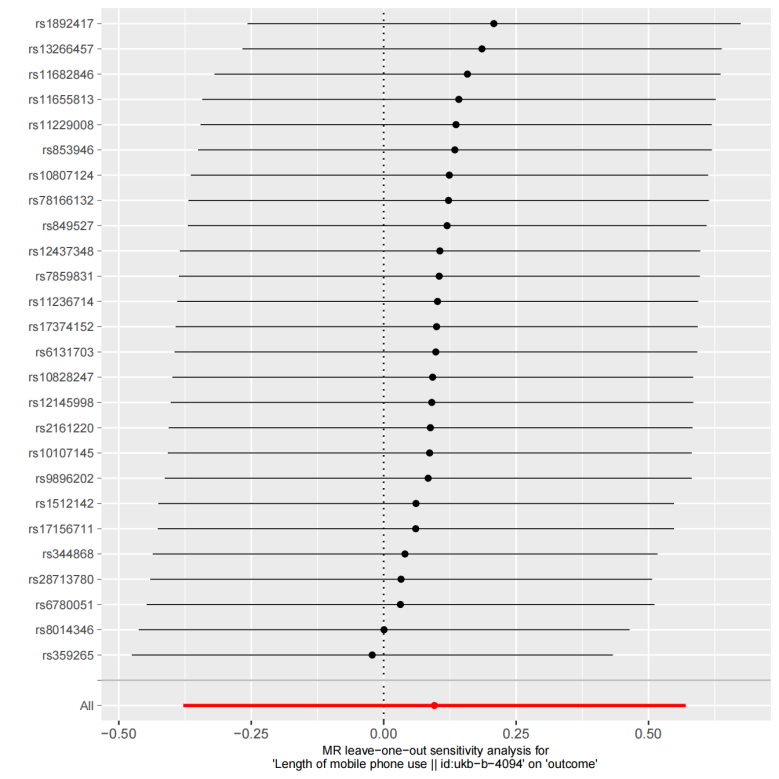  **C** | 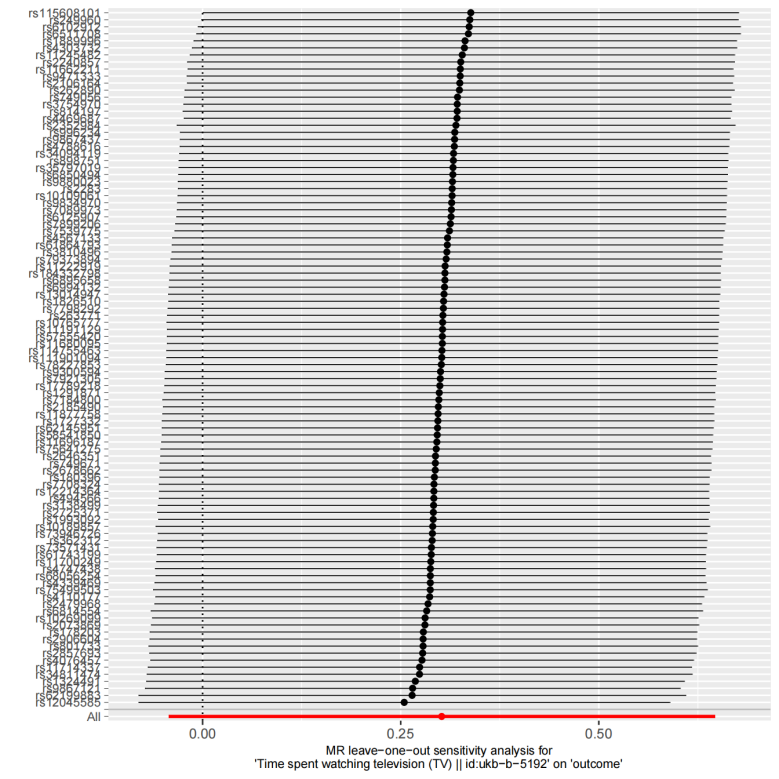 |

**Fig. S15.** Leave-one-out plot for the effects of digital device use on the risk of post traumatic stress disorder

Length of mobile phone use, (B) Time spent using computer, (C) Plays computer games, and (D) Time spent watching television

| 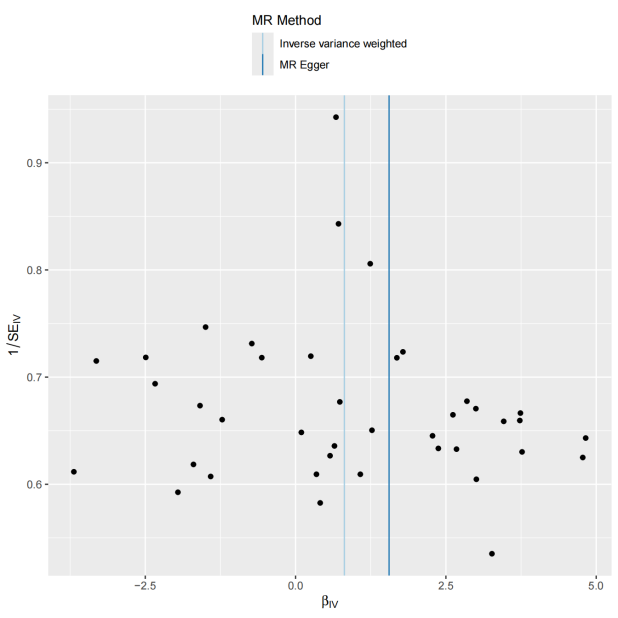  **A** | 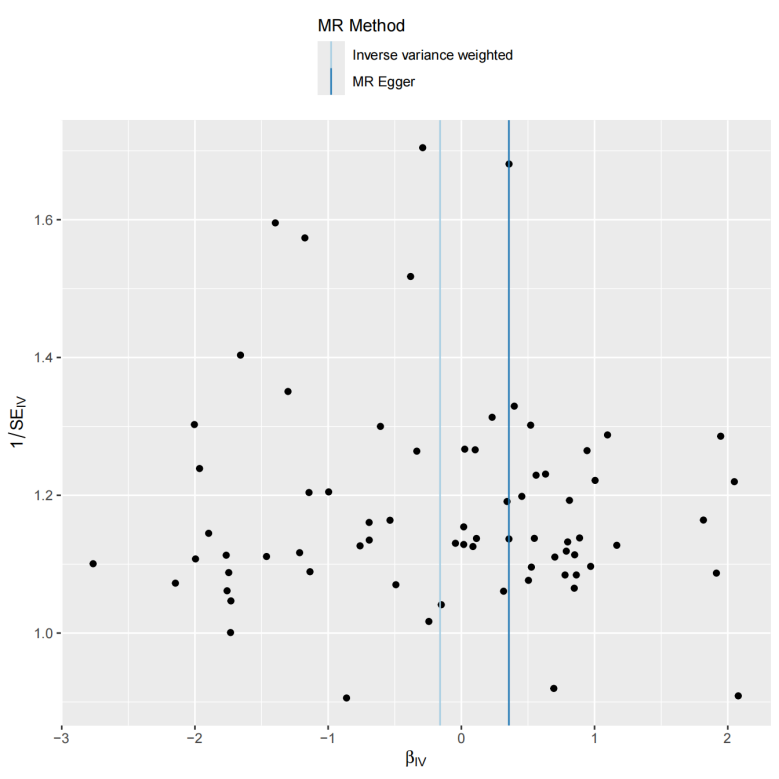  **B** |
| --- | --- |
| 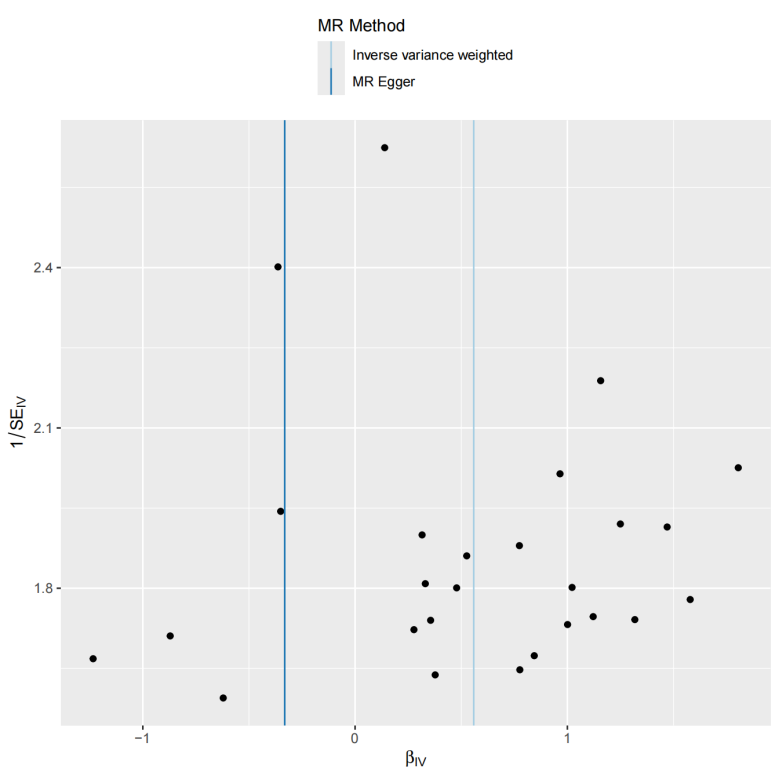  **C** | 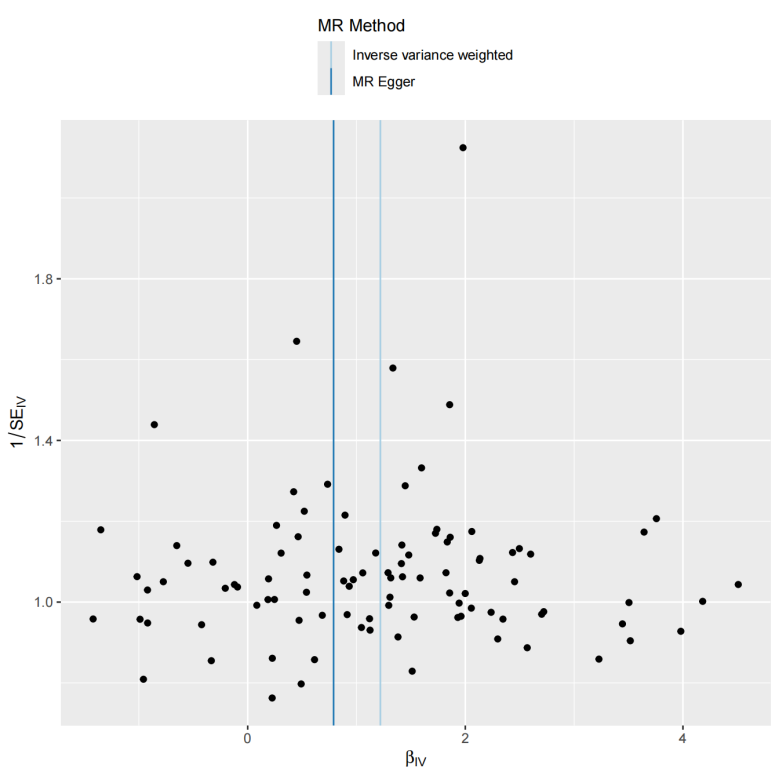  **D** |

**Fig. S16.** Funnel plots for the effects of digital device use on the risk of attention-deficit/hyperactivity disorder

(A) Length of mobile phone use, (B) Time spent using computer, (C) Plays computer games, and (D) Time spent watching television

| 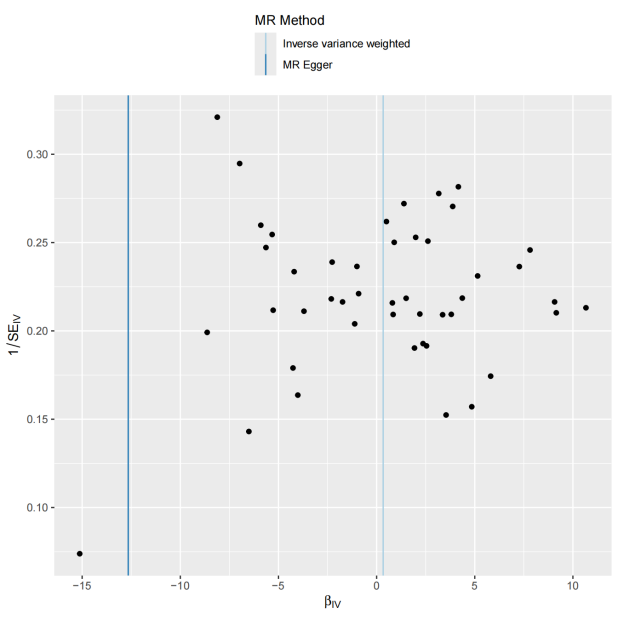  **A** | 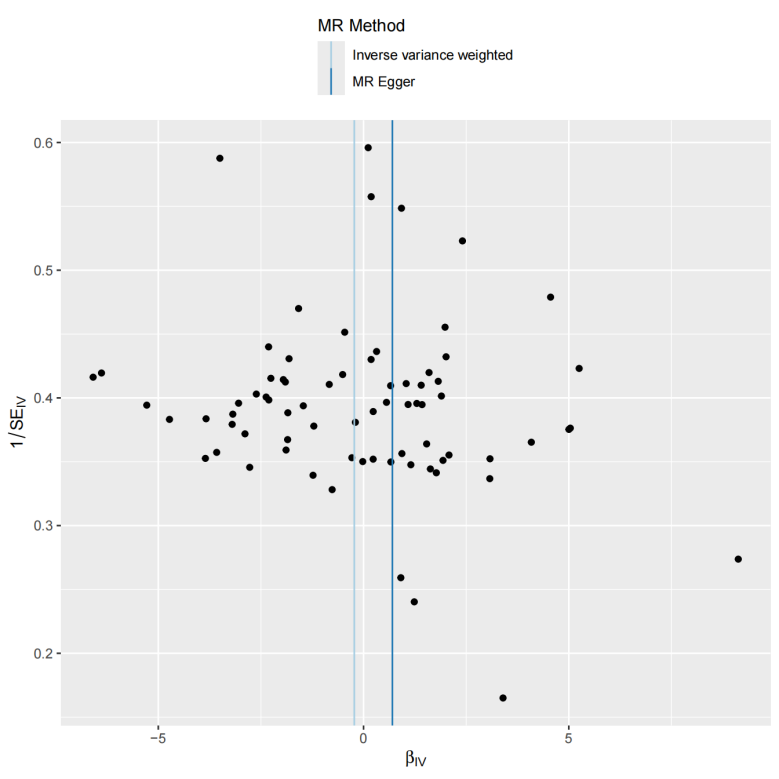  **B** |
| --- | --- |
| 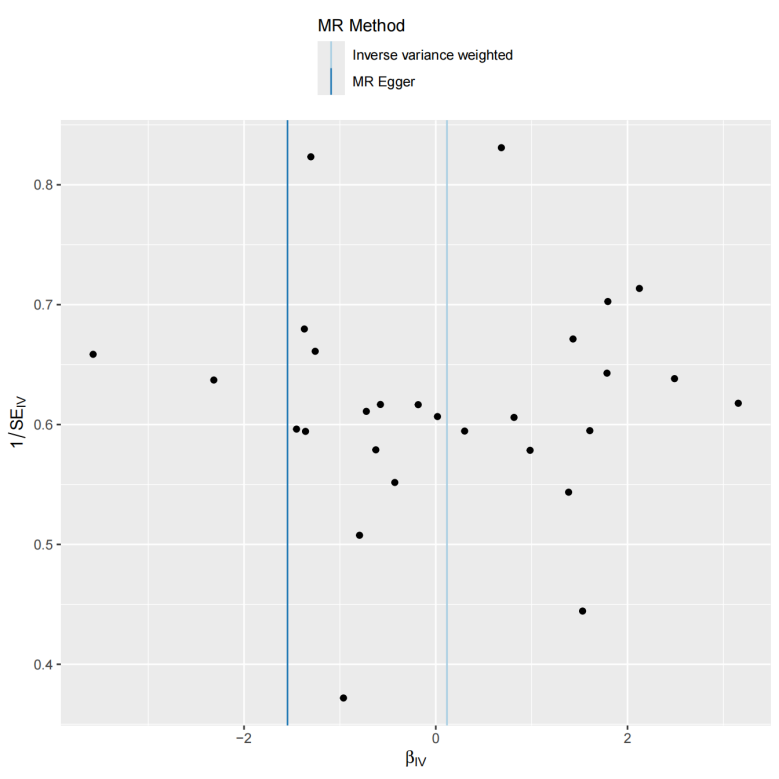  **C** | 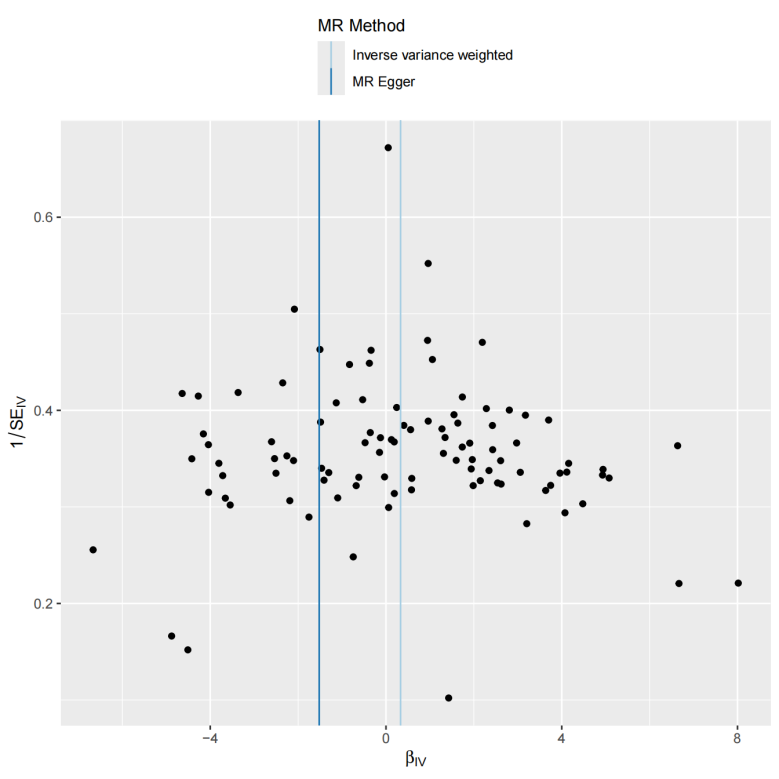  **D** |

**Fig. S17.** Funnel plots for the effects of digital device use on the risk of anxiety disorder

(A) Length of mobile phone use, (B) Time spent using computer, (C) Plays computer games, and (D) Time spent watching television

| 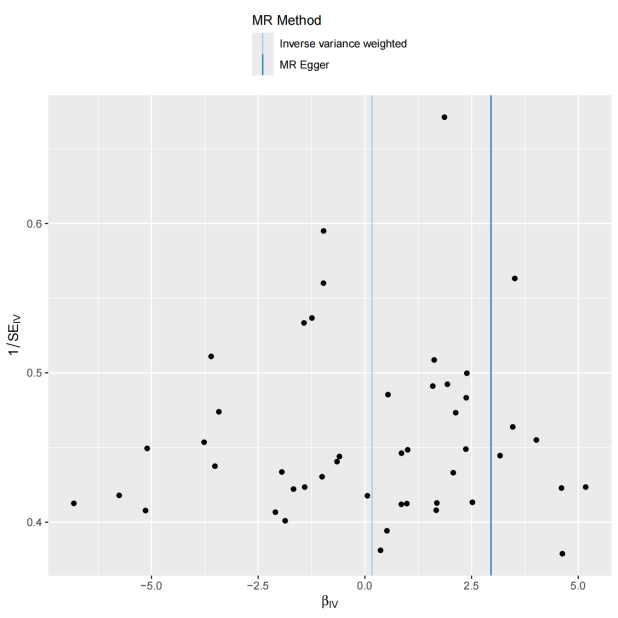  **A** | 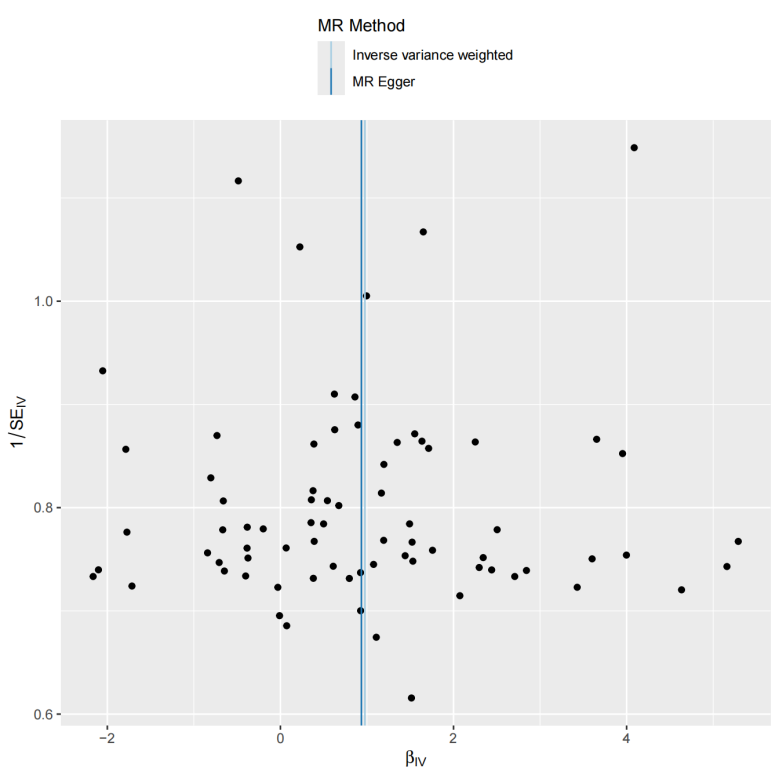  **B** |
| --- | --- |
| 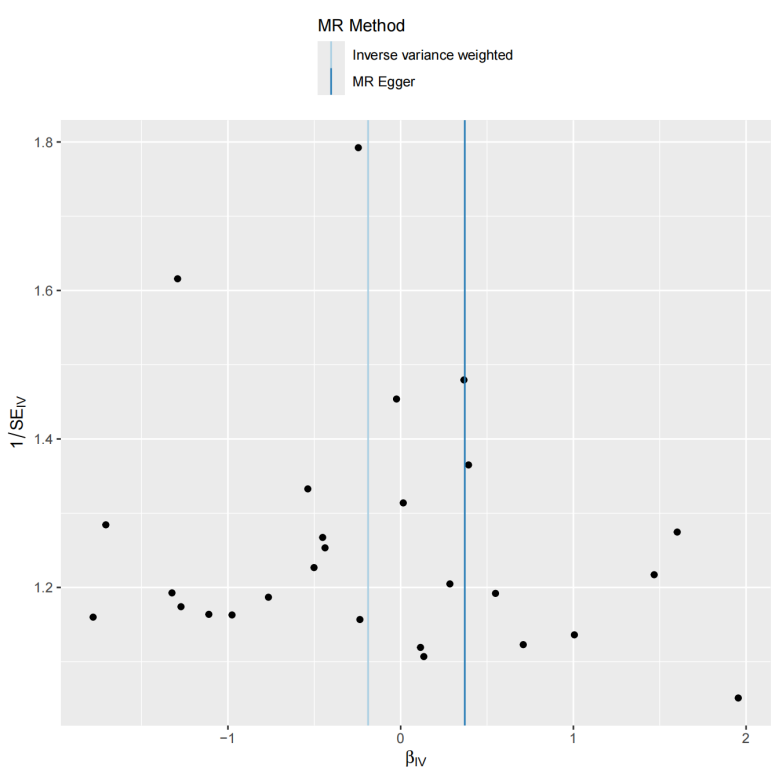  **C** | 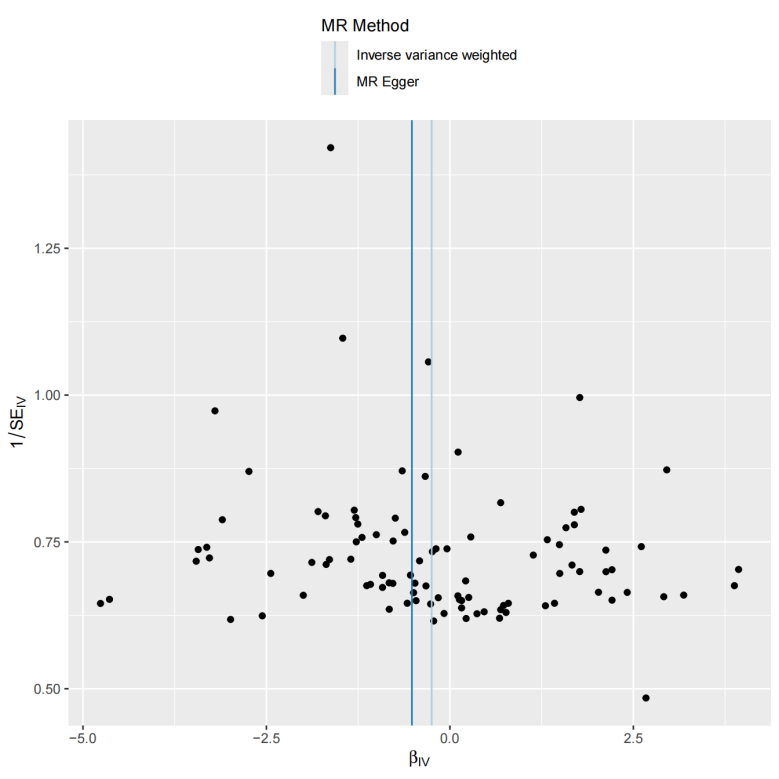  **D** |

**Fig. S18.** Funnel plots for the effects of digital device use on the risk of autism spectrum disorder

(A) Length of mobile phone use, (B) Time spent using computer, (C) Plays computer games, and (D) Time spent watching television

| 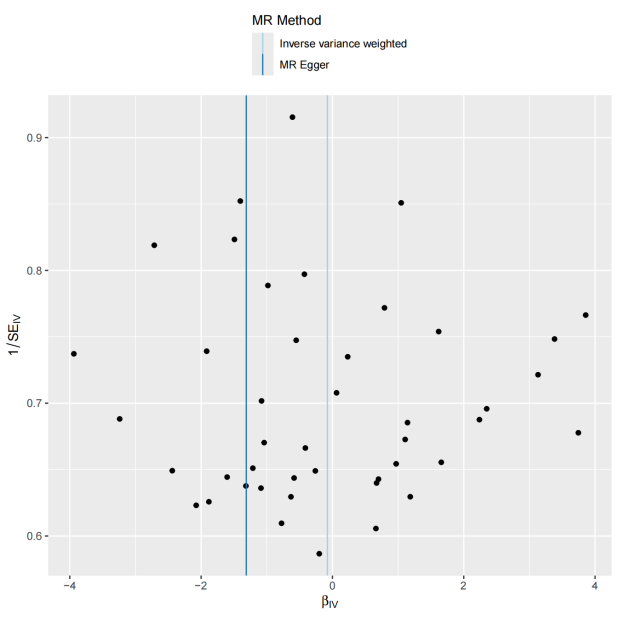  **A** | 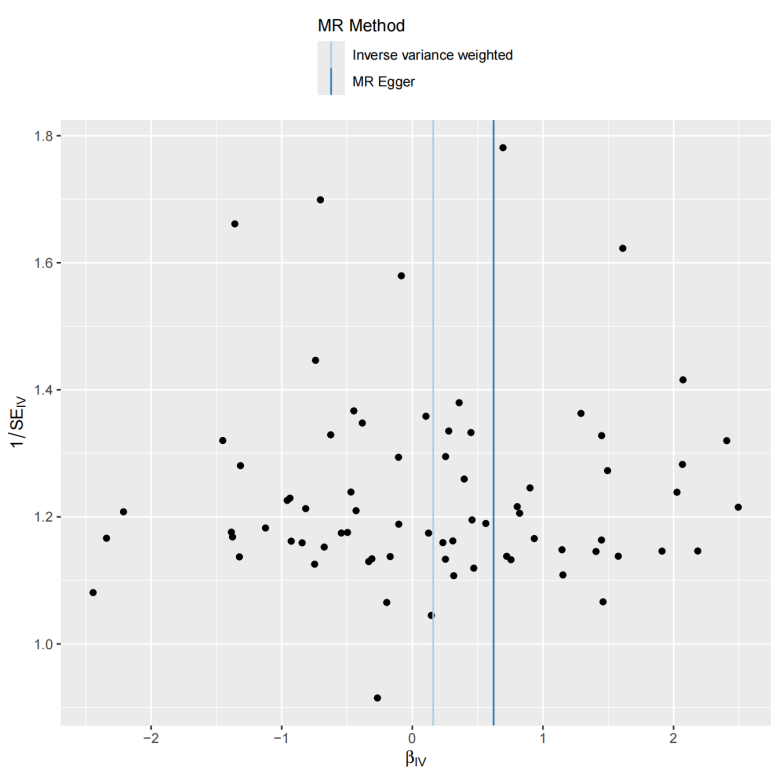  **B** |
| --- | --- |
| 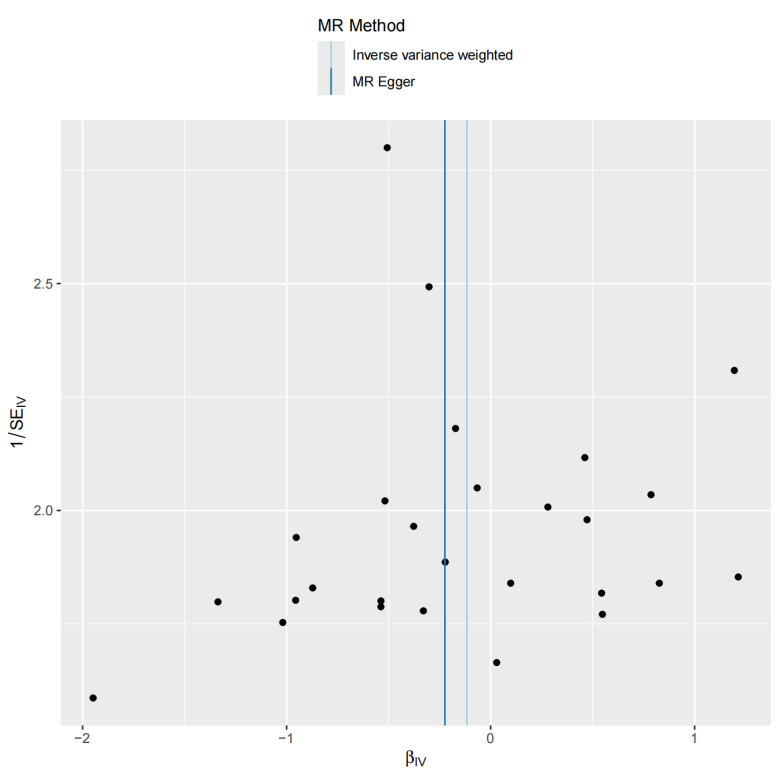  **C** | 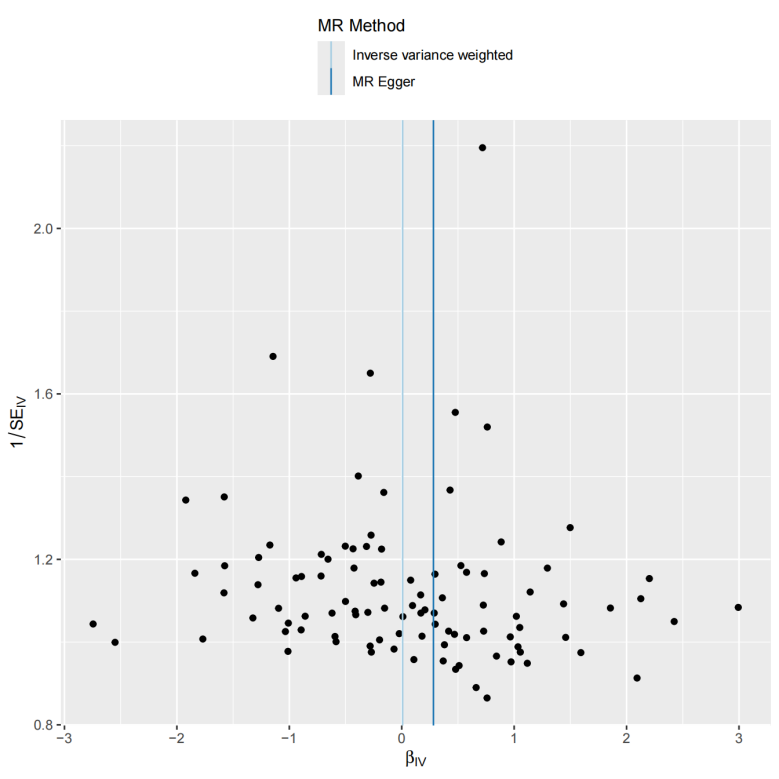  **D** |

**Fig. S19.** Funnel plots for the effects of digital device use on the risk of major depression disorder

(A) Length of mobile phone use, (B) Time spent using computer, (C) Plays computer games, and (D) Time spent watching television

| 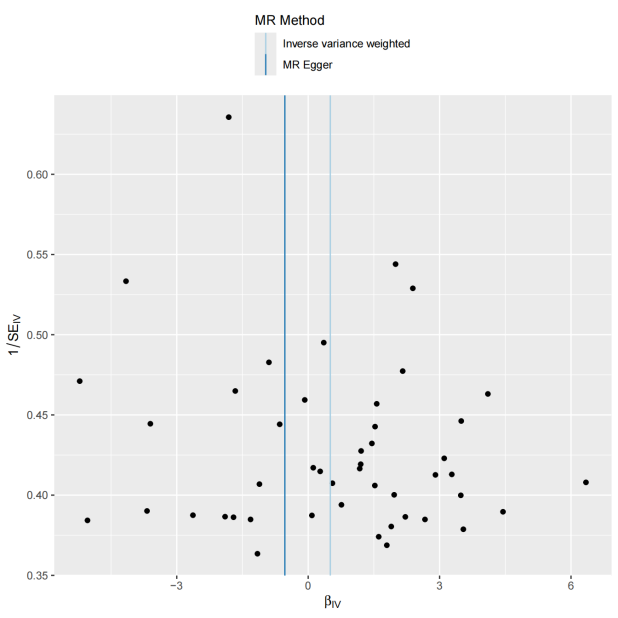  **A** | 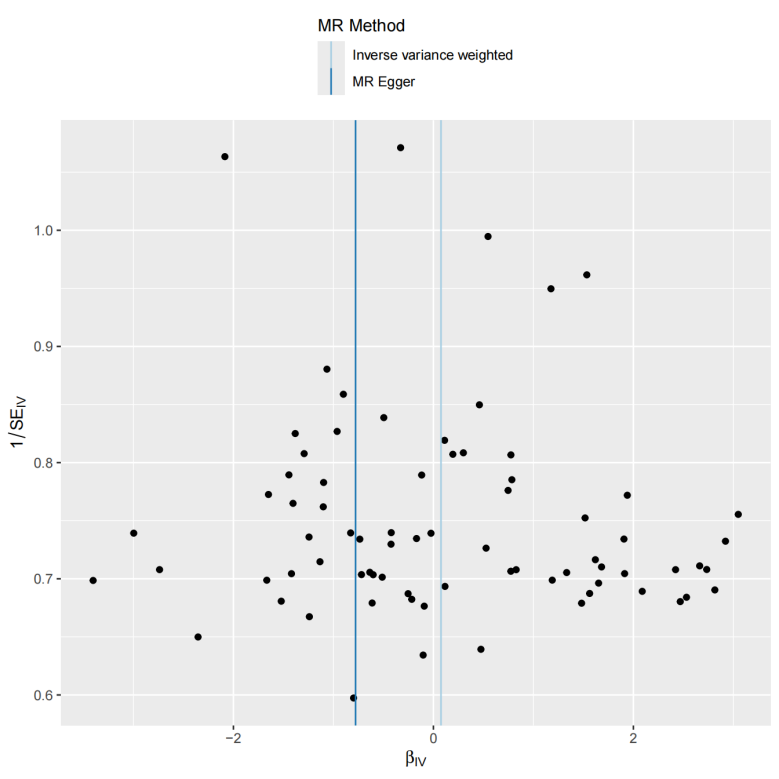  **B** |
| --- | --- |
| 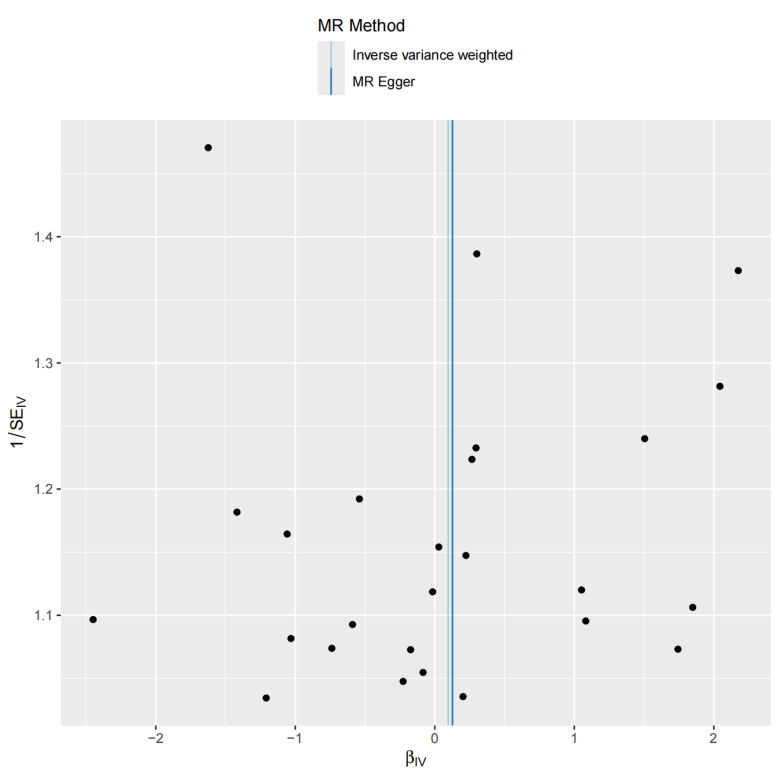  **C** | 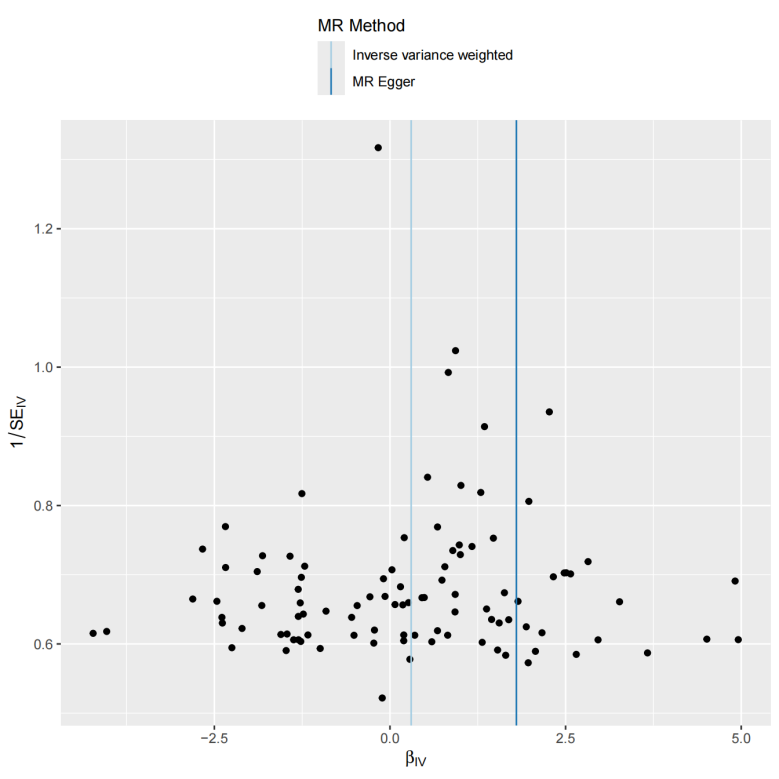  **D** |

**Fig. S20.** Funnel plots for the effects of digital device use on the risk of post traumatic stress disorder

(A) Length of mobile phone use, (B) Time spent using computer, (C) Plays computer games, and (D) Time spent watching television

**Table S9.** The causal effect of the five psychiatric disorders on digital device use in the mendelian randomization study

| **Exposure** | **Outcome** | **Method** | **Number of SNVs** | **Beta (95% CI)** | ***P-value*** | ***P-FDR*** |
| --- | --- | --- | --- | --- | --- | --- |
| ADHD on digital devices use | | |  |  |  |  |
| ADHD | Length of mobile phone use | MR Egger | 19 | -0.002 (-0.189, 0.186) | 0.984 | 0.997 |
|  |  | Weighted median | 19 | 0.014 (-0.02, 0.049) | 0.416 | 0.801 |
|  |  | Simple mode | 19 | 0.024 (-0.053, 0.101) | 0.548 | 0.801 |
|  |  | Weighted mode | 19 | 0.028 (-0.045, 0.102) | 0.459 | 0.801 |
| ADHD | Time spent using computer | MR Egger | 20 | 0.02 (-0.116, 0.155) | 0.778 | 0.889 |
|  |  | Weighted median | 20 | -0.016 (-0.039, 0.007) | 0.178 | 0.756 |
|  |  | Simple mode | 20 | -0.038 (-0.104, 0.027) | 0.264 | 0.756 |
|  |  | Weighted mode | 20 | -0.029 (-0.081, 0.024) | 0.298 | 0.756 |
| ADHD | Plays computer games | MR Egger | 22 | 0.012 (-0.049, 0.073) | 0.706 | 0.869 |
|  |  | **Weighted median** | **22** | **0.028 (0.017, 0.040)** | **< 0.001** | **< 0.001** |
|  |  | Simple mode | 22 | 0.036 (0.014, 0.058) | 0.005 | 0.133 |
|  |  | Weighted mode | 22 | 0.033 (0.009, 0.058) | 0.015 | 0.300 |
| ADHD | Time spent watching television | MR Egger | 18 | 0.034 (-0.071, 0.140) | 0.536 | 0.801 |
|  |  | **Weighted median** | **18** | **0.036 (0.016, 0.056)** | **< 0.001** | **< 0.001** |
|  |  | Simple mode | 18 | 0.053 (0.008, 0.098) | 0.034 | 0.389 |
|  |  | Weighted mode | 18 | 0.044 (0.010, 0.078) | 0.021 | 0.320 |
| AD on digital devices use | |  |  |  |  |  |
| AD | Length of mobile phone use | MR Egger | 16 | 0.025 (-0.016, 0.066) | 0.257 | 0.756 |
|  |  | Weighted median | 16 | -0.009 (-0.024, 0.006) | 0.225 | 0.756 |
|  |  | Simple mode | 16 | -0.022 (-0.052, 0.007) | 0.163 | 0.756 |
|  |  | Weighted mode | 16 | -0.02 (-0.054, 0.013) | 0.255 | 0.756 |
| AD | Time spent using computer | MR Egger | 16 | -0.003 (-0.026, 0.020) | 0.822 | 0.889 |
|  |  | Weighted median | 16 | -0.002 (-0.011, 0.006) | 0.584 | 0.801 |
|  |  | Simple mode | 16 | -0.008 (-0.022, 0.006) | 0.285 | 0.756 |
|  |  | Weighted mode | 16 | -0.005 (-0.019, 0.010) | 0.538 | 0.801 |
| AD | Plays computer games | MR Egger | 16 | -0.003 (-0.019, 0.014) | 0.743 | 0.874 |
|  |  | Weighted median | 16 | -0.003 (-0.008, 0.003) | 0.295 | 0.756 |
|  |  | Simple mode | 16 | -0.005 (-0.016, 0.006) | 0.377 | 0.777 |
|  |  | Weighted mode | 16 | -0.002 (-0.013, 0.008) | 0.663 | 0.864 |
| AD | Time spent watching television | MR Egger | 16 | 0.008 (-0.012, 0.028) | 0.426 | 0.801 |
|  |  | Weighted median | 16 | 0.005 (-0.002, 0.013) | 0.140 | 0.740 |
|  |  | Simple mode | 16 | 0.005 (-0.006, 0.016) | 0.345 | 0.767 |
|  |  | Weighted mode | 16 | 0.006 (-0.005, 0.016) | 0.296 | 0.756 |
| ASD on digital devices use | |  |  |  |  |  |
| ASD | Length of mobile phone use | MR Egger | 46 | 0.018 (-0.022, 0.058) | 0.390 | 0.780 |
|  |  | Weighted median | 46 | -0.007 (-0.025, 0.012) | 0.490 | 0.801 |
|  |  | Simple mode | 46 | -0.008 (-0.054, 0.038) | 0.737 | 0.874 |
|  |  | Weighted mode | 46 | -0.009 (-0.051, 0.034) | 0.689 | 0.864 |
| ASD | Time spent using computer | MR Egger | 44 | 0 (-0.026, 0.026) | 0.992 | 0.997 |
|  |  | Weighted median | 44 | 0.010 (-0.002, 0.023) | 0.113 | 0.740 |
|  |  | Simple mode | 44 | 0.010 (-0.018, 0.037) | 0.495 | 0.801 |
|  |  | Weighted mode | 44 | 0.010 (-0.017, 0.037) | 0.475 | 0.801 |
| ASD | Plays computer games | MR Egger | 48 | 0.012 (-0.001, 0.025) | 0.081 | 0.720 |
|  |  | Weighted median | 48 | 0.007 (0.001, 0.014) | 0.024 | 0.320 |
|  |  | Simple mode | 48 | 0.008 (-0.006, 0.022) | 0.256 | 0.756 |
|  |  | Weighted mode | 48 | 0.007 (-0.006, 0.021) | 0.281 | 0.756 |
| ASD | Time spent watching television | MR Egger | 45 | 0.006 (-0.015, 0.027) | 0.561 | 0.801 |
|  |  | Weighted median | 45 | 0.005 (-0.005, 0.016) | 0.312 | 0.756 |
|  |  | Simple mode | 45 | 0.020 (-0.007, 0.047) | 0.148 | 0.740 |
|  |  | Weighted mode | 45 | 0.020 (-0.005, 0.045) | 0.123 | 0.740 |
| MDD on digital devices use | |  |  |  |  |  |
| MDD | Length of mobile phone use | MR Egger | 62 | -0.001 (-0.062, 0.059) | 0.970 | 0.997 |
|  |  | Weighted median | 62 | 0.003 (-0.022, 0.028) | 0.818 | 0.889 |
|  |  | Simple mode | 62 | -0.013 (-0.074, 0.048) | 0.674 | 0.864 |
|  |  | Weighted mode | 62 | -0.007 (-0.061, 0.047) | 0.799 | 0.889 |
| MDD | Time spent using computer | MR Egger | 60 | -0.022 (-0.057, 0.014) | 0.233 | 0.756 |
|  |  | Weighted median | 60 | 0.013 (-0.003, 0.029) | 0.104 | 0.740 |
|  |  | Simple mode | 60 | 0.018 (-0.022, 0.058) | 0.379 | 0.777 |
|  |  | Weighted mode | 60 | 0.017 (-0.020, 0.053) | 0.368 | 0.777 |
| MDD | Plays computer games | MR Egger | 62 | -0.008 (-0.032, 0.016) | 0.530 | 0.801 |
|  |  | Weighted median | 62 | 0.005 (-0.004, 0.014) | 0.290 | 0.756 |
|  |  | Simple mode | 62 | -0.004 (-0.028, 0.021) | 0.772 | 0.889 |
|  |  | Weighted mode | 62 | 0.011 (-0.010, 0.032) | 0.309 | 0.756 |
| MDD | Time spent watching television | MR Egger | 62 | -0.009 (-0.043, 0.024) | 0.591 | 0.801 |
|  |  | Weighted median | 62 | 0.005 (-0.009, 0.019) | 0.521 | 0.801 |
|  |  | Simple mode | 62 | 0 (-0.032, 0.032) | 0.997 | 0.997 |
|  |  | Weighted mode | 62 | 0.002 (-0.027, 0.030) | 0.908 | 0.969 |
| PTSD on digital devices use | |  |  |  |  |  |
| PTSD | Length of mobile phone use | MR Egger | 34 | 0.010 (-0.025, 0.046) | 0.580 | 0.801 |
|  |  | Weighted median | 34 | 0.002 (-0.017, 0.022) | 0.808 | 0.889 |
|  |  | Simple mode | 34 | 0.016 (-0.028, 0.060) | 0.487 | 0.801 |
|  |  | Weighted mode | 34 | 0.015 (-0.028, 0.057) | 0.505 | 0.801 |
| PTSD | Time spent using computer | MR Egger | 35 | 0 (-0.024, 0.025) | 0.970 | 0.997 |
|  |  | Weighted median | 35 | 0.002 (-0.010, 0.015) | 0.726 | 0.874 |
|  |  | Simple mode | 35 | 0.008 (-0.019, 0.034) | 0.576 | 0.801 |
|  |  | Weighted mode | 35 | 0.005 (-0.019, 0.029) | 0.691 | 0.864 |
| PTSD | Plays computer games | MR Egger | 35 | -0.006 (-0.017, 0.006) | 0.334 | 0.763 |
|  |  | Weighted median | 35 | 0.007 (0, 0.014) | 0.059 | 0.590 |
|  |  | Simple mode | 35 | 0.012 (-0.004, 0.028) | 0.139 | 0.740 |
|  |  | Weighted mode | 35 | 0.007 (-0.007, 0.022) | 0.327 | 0.763 |
| PTSD | Time spent watching television | MR Egger | 32 | 0.006 (-0.016, 0.027) | 0.603 | 0.804 |
|  |  | Weighted median | 32 | 0.010 (-0.002, 0.022) | 0.090 | 0.720 |
|  |  | Simple mode | 32 | 0.007 (-0.014, 0.029) | 0.506 | 0.801 |
|  |  | Weighted mode | 32 | 0.011 (-0.009, 0.032) | 0.279 | 0.756 |

**Note:** Abbreviations: ADHD, attention-deficit/hyperactivity disorder; AD, Anxiety disorder; ASD, autism spectrum disorder; CI, confidence interval; MDD, major depression disorder; PTSD, post traumatic stress disorder; SNV, single-nucleotide variant

**Table S10.** Test on pleiotropy and heterogeneity for the selected SNVs related to psychiatric disorders used as instruments in analysis of effect on digital device use

| **Exposure** | **Outcome** | **Pleiotropy** | | |  | **Heterogeneity (IVW)** | | |
| --- | --- | --- | --- | --- | --- | --- | --- | --- |
|  |  | **MR-Egger intercept** | **SE** | ***P-*value** |  | ***Cochran’s Q*** | ***df*** | ***P-*value** |
| ADHD | Length of mobile phone use | 0.0014 | 0.006 | 0.819 |  | 40.719 | 18 | 1.66E-03 |
| ADHD | Time spent using computer | -0.0017 | 0.004 | 0.711 |  | 56.127 | 19 | 1.56E-05 |
| ADHD | Plays computer games | 0.0006 | 0.002 | 0.772 |  | 50.936 | 21 | 2.70E-04 |
| ADHD | Time spent watching television | -0.0003 | 0.004 | 0.934 |  | 43.716 | 17 | 3.77E-04 |
| AD | Length of mobile phone use | -0.0053 | 0.004 | 0.150 |  | 21.48 | 15 | 1.22E-01 |
| AD | Time spent using computer | 0.0002 | 0.002 | 0.924 |  | 12.166 | 15 | 6.66E-01 |
| AD | Plays computer games | 0.0002 | 0.001 | 0.880 |  | 22.347 | 15 | 9.90E-02 |
| AD | Time spent watching television | -0.0004 | 0.002 | 0.801 |  | 9.318 | 15 | 8.60E-01 |
| ASD | Length of mobile phone use | -0.002 | 0.002 | 0.291 |  | 80.958 | 45 | 8.06E-04 |
| ASD | Time spent using computer | 0.0013 | 0.001 | 0.306 |  | 77.522 | 43 | 9.75E-04 |
| ASD | Plays computer games | -0.0006 | 0.001 | 0.351 |  | 70.054 | 47 | 1.62E-02 |
| ASD | Time spent watching television | -0.0004 | 0.001 | 0.666 |  | 65.295 | 44 | 2.02E-02 |
| MDD | Length of mobile phone use | 0.0011 | 0.002 | 0.541 |  | 102.205 | 61 | 7.47E-04 |
| MDD | Time spent using computer | 0.0021 | 0.001 | 0.049 |  | 85.987 | 59 | 1.25E-02 |
| MDD | Plays computer games | 0.0008 | 0.001 | 0.281 |  | 123.635 | 61 | 3.79E-06 |
| MDD | Time spent watching television | 0.0009 | 0.001 | 0.363 |  | 100.061 | 61 | 1.20E-03 |
| PTSD | Length of mobile phone use | -0.0011 | 0.002 | 0.620 |  | 41.431 | 33 | 1.49E-01 |
| PTSD | Time spent using computer | 0.0001 | 0.001 | 0.965 |  | 51.348 | 34 | 2.85E-02 |
| PTSD | Plays computer games | 0.0018 | 0.001 | 0.015 |  | 40.635 | 34 | 2.01E-01 |
| PTSD | Time spent watching television | 0.0003 | 0.001 | 0.816 |  | 43.932 | 31 | 6.18E-02 |

**Note:** Abbreviations: ADHD, attention-deficit/hyperactivity disorder; AD, Anxiety disorder; ASD, autism spectrum disorder; IVW, the inverse variance weighted; MDD, major depression disorder; MR, Mendelian randomization; PTSD, post traumatic stress disorder; SE, standard error; SNV, single-nucleotide varian

| 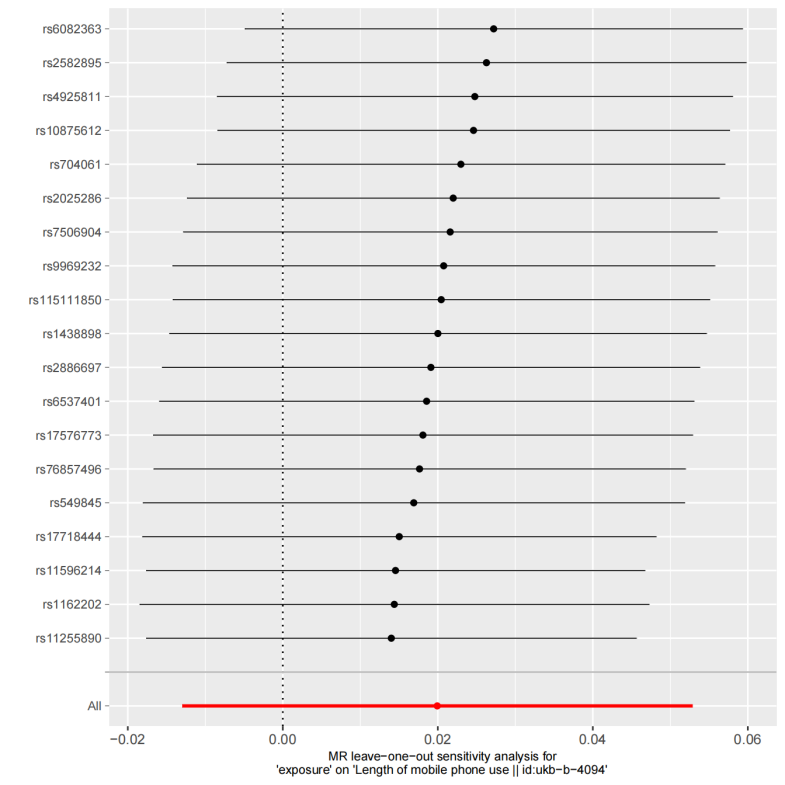  **A**  **B** | 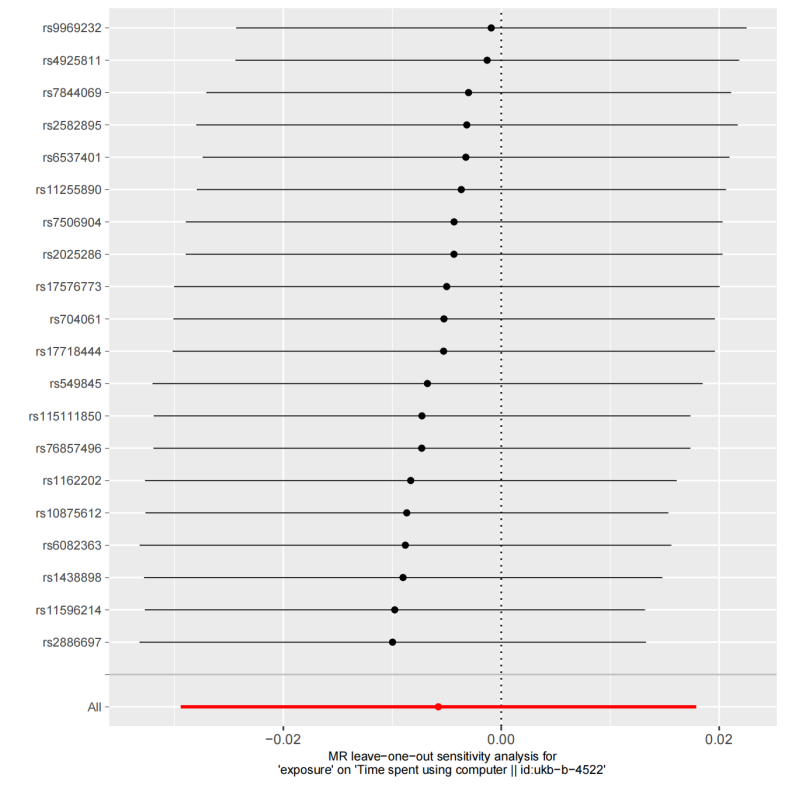 |
| --- | --- |
| 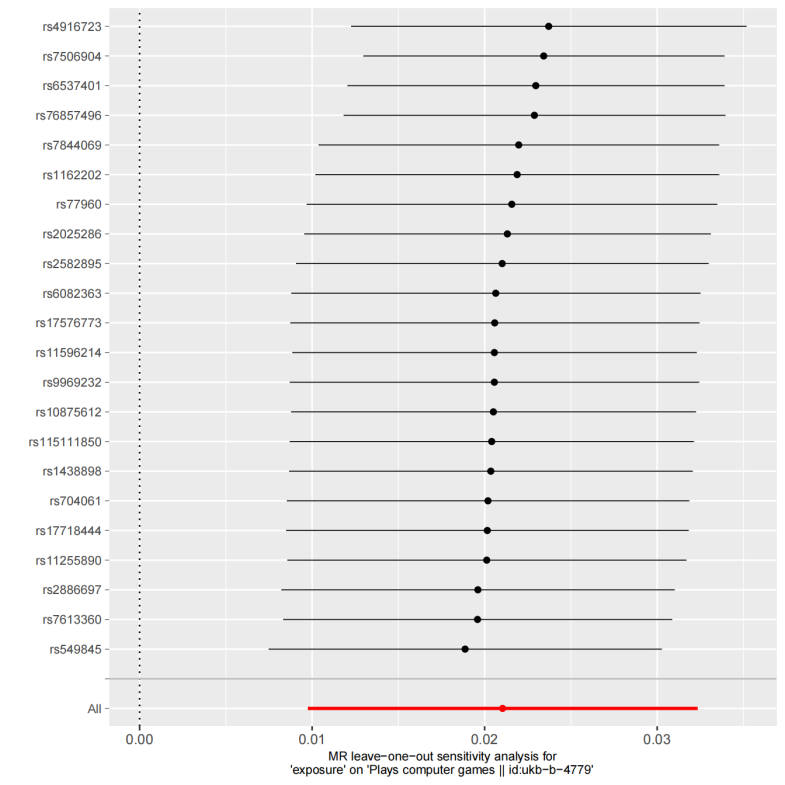  **D**  **C** | 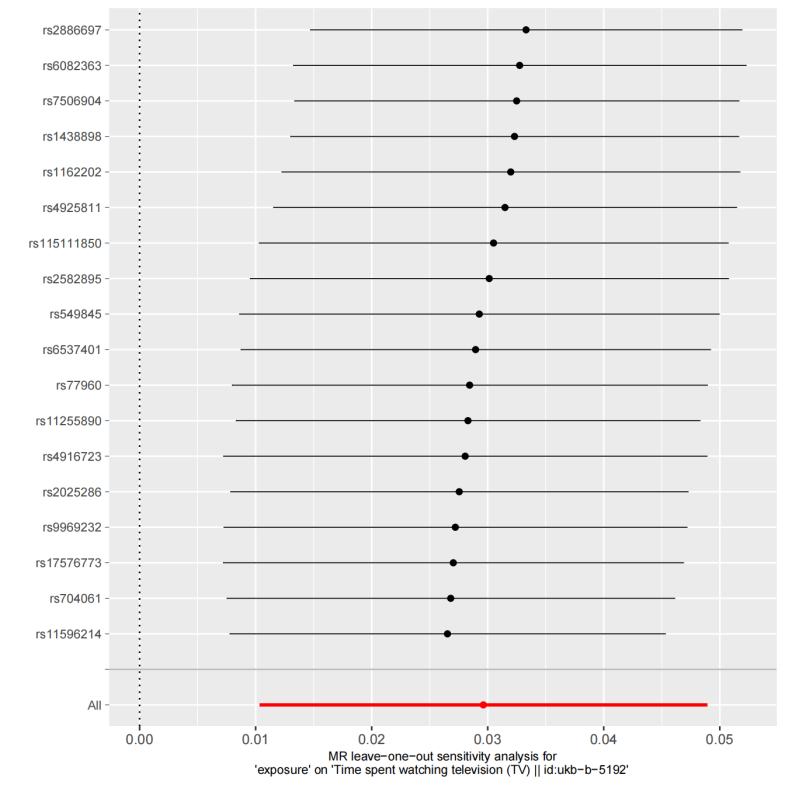 |

**Fig. S21.** Leave-one-out plot for the effects of attention-deficit/hyperactivity disorder on digital device use

(A) Length of mobile phone use, (B) Time spent using computer, (C) Plays computer games, and (D) Time spent watching television

| 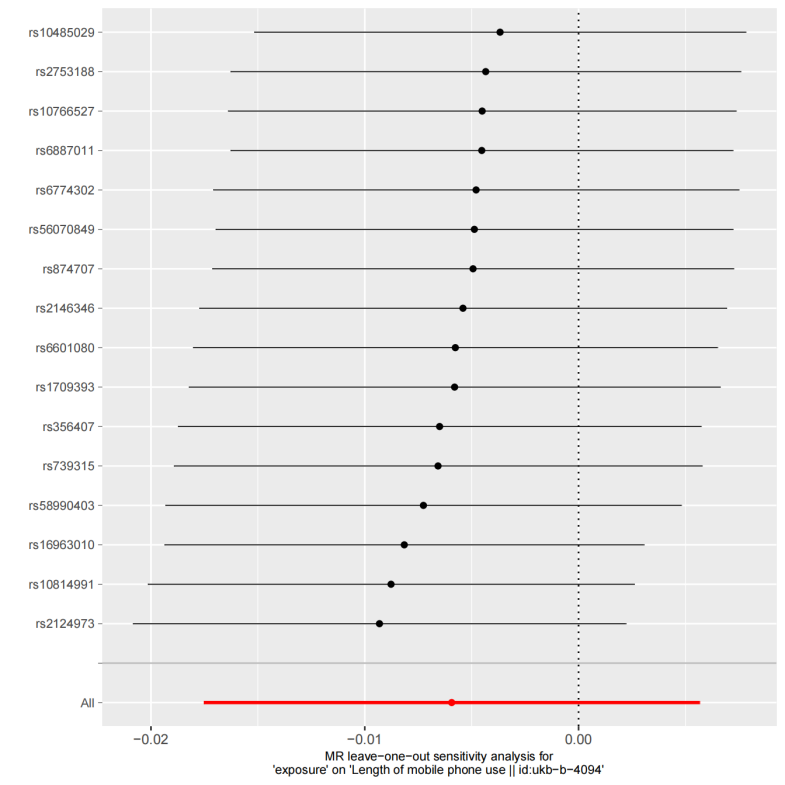  **D**  **B**  **A** | 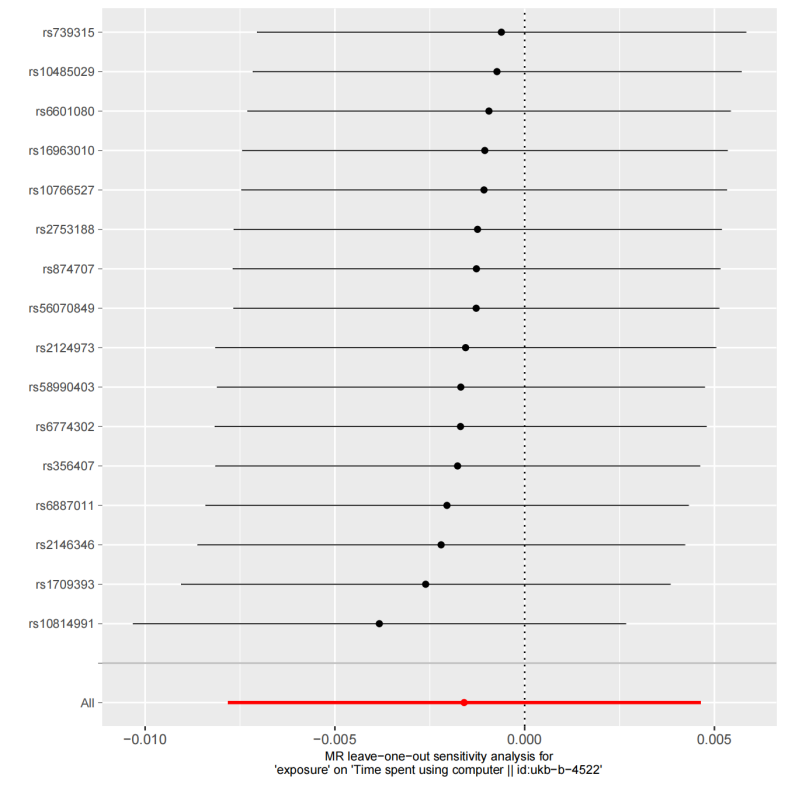 |
| --- | --- |
| 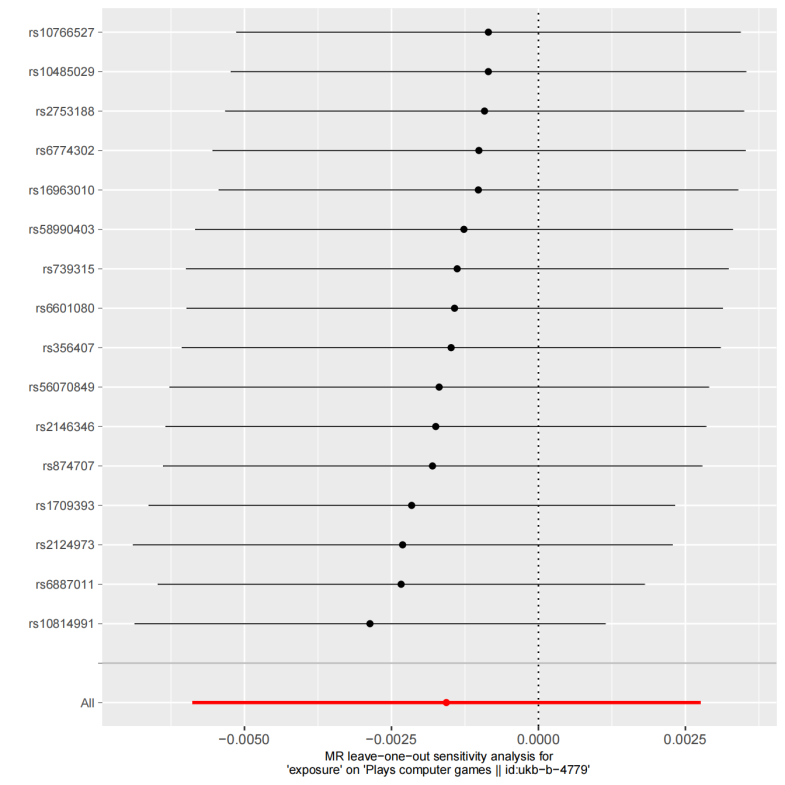  **C** | 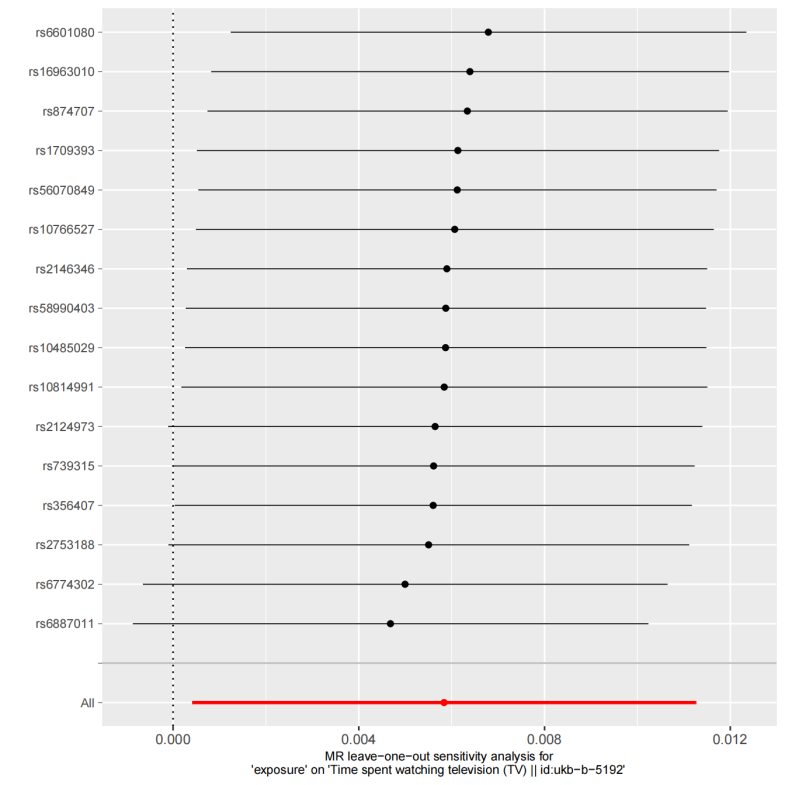 |

**Fig. S22.** Leave-one-out plot for the effects of anxiety disorder on digital device use

(A) Length of mobile phone use, (B) Time spent using computer, (C) Plays computer games, and (D) Time spent watching television

| 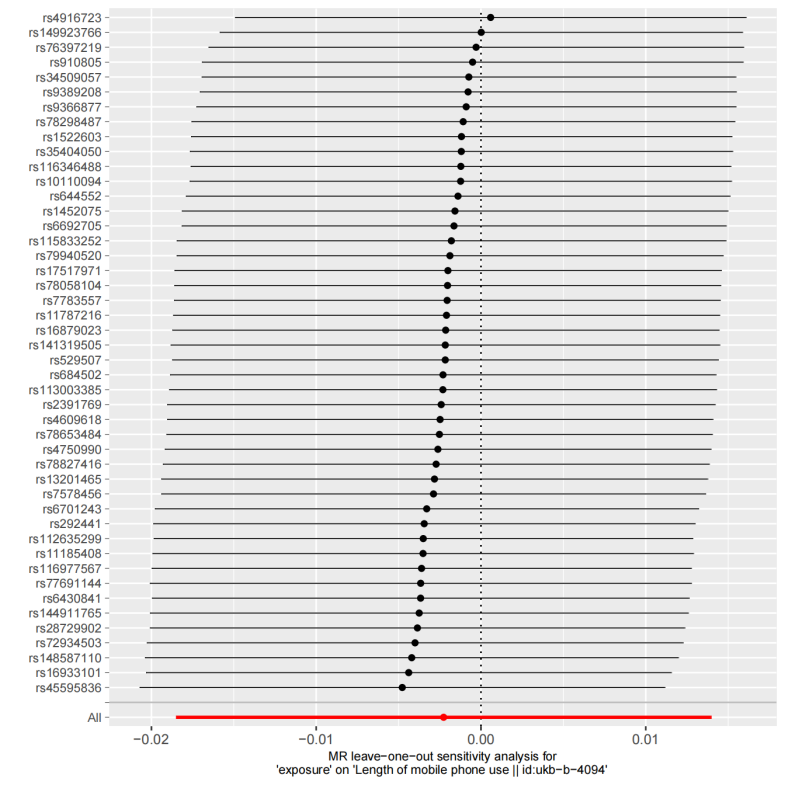  **D**  **B**  **A** | 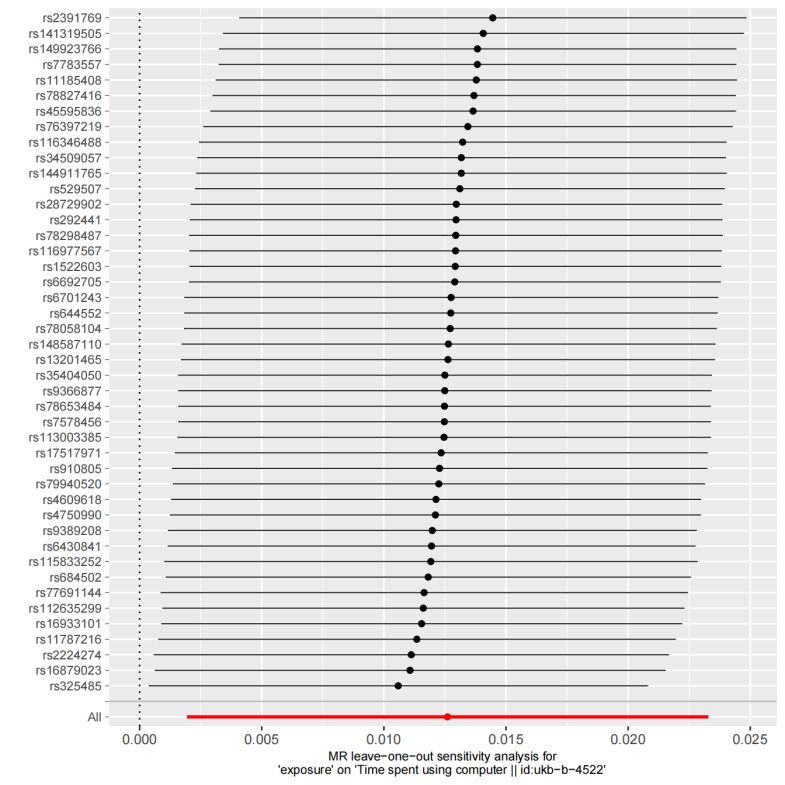 |
| --- | --- |
| 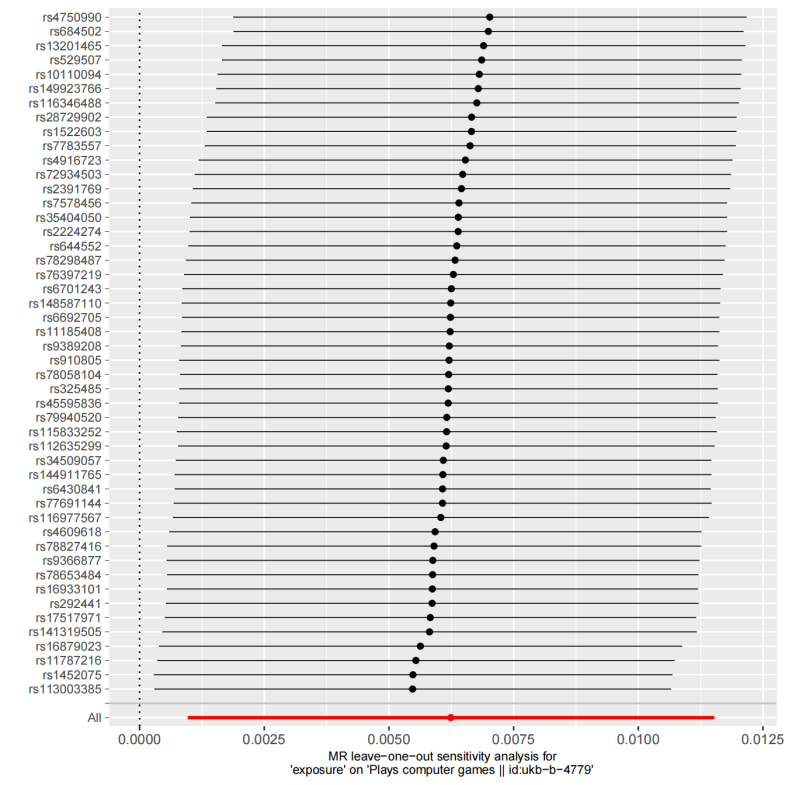  **C** | 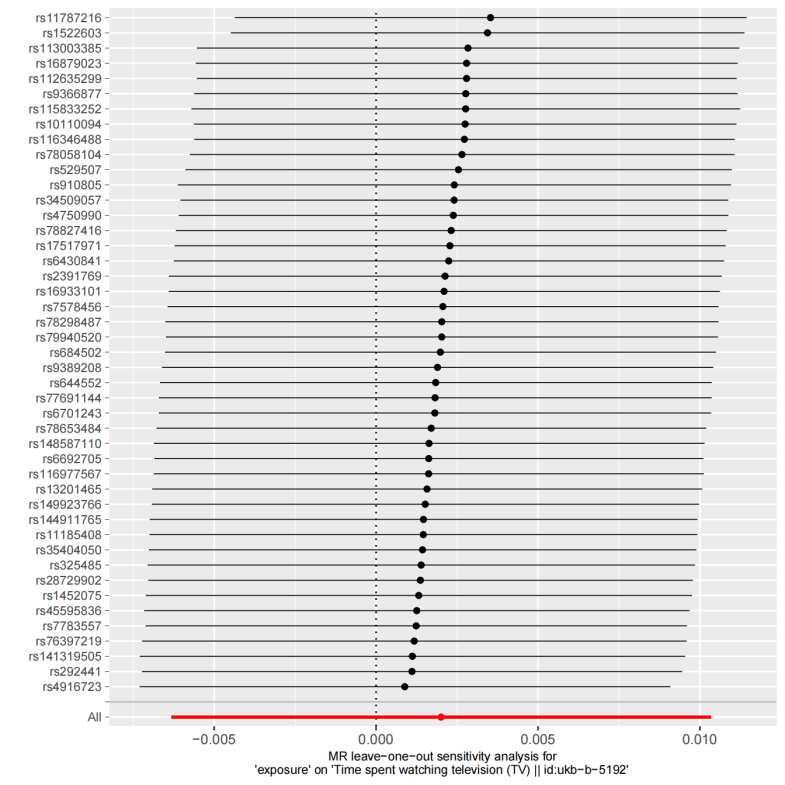 |

**Fig. S23.** Leave-one-out plot for the effects of autism spectrum disorder on digital device use

(A) Length of mobile phone use, (B) Time spent using computer, (C) Plays computer games, and (D) Time spent watching television

| 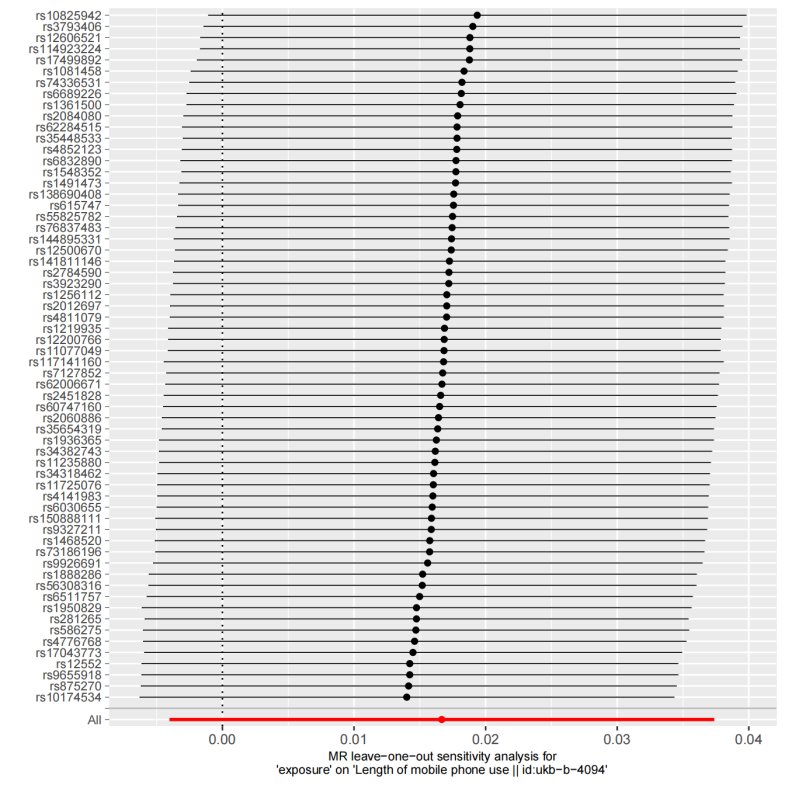  **D**  **B**  **A** | 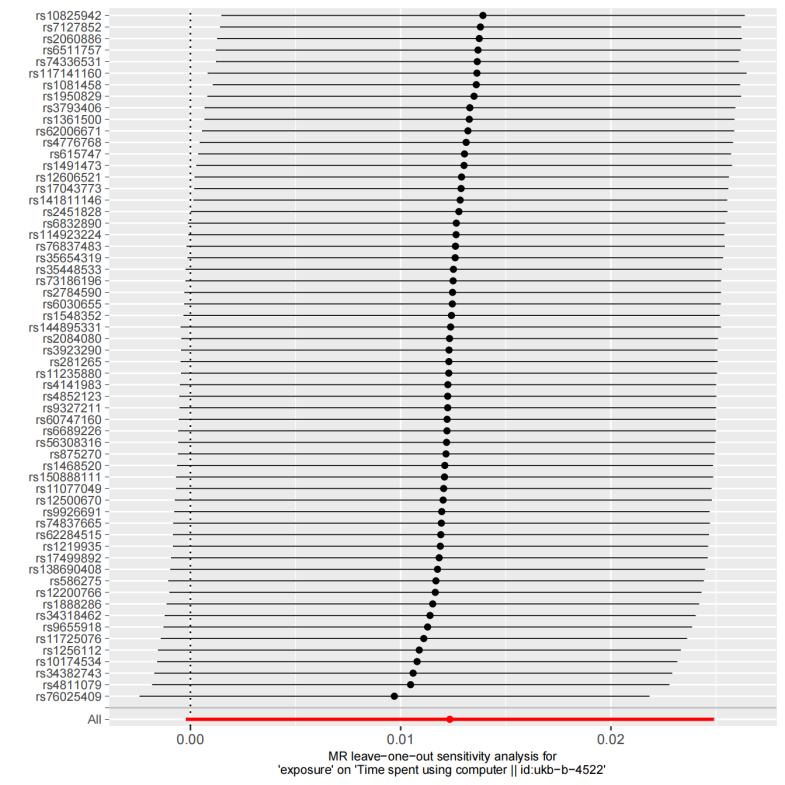 |
| --- | --- |
| 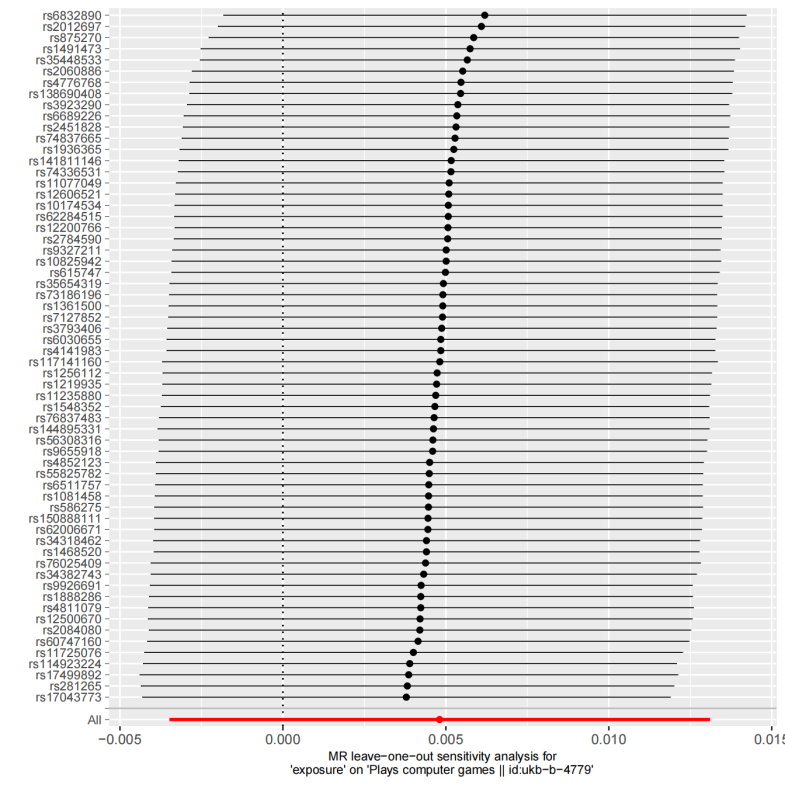  **C** | 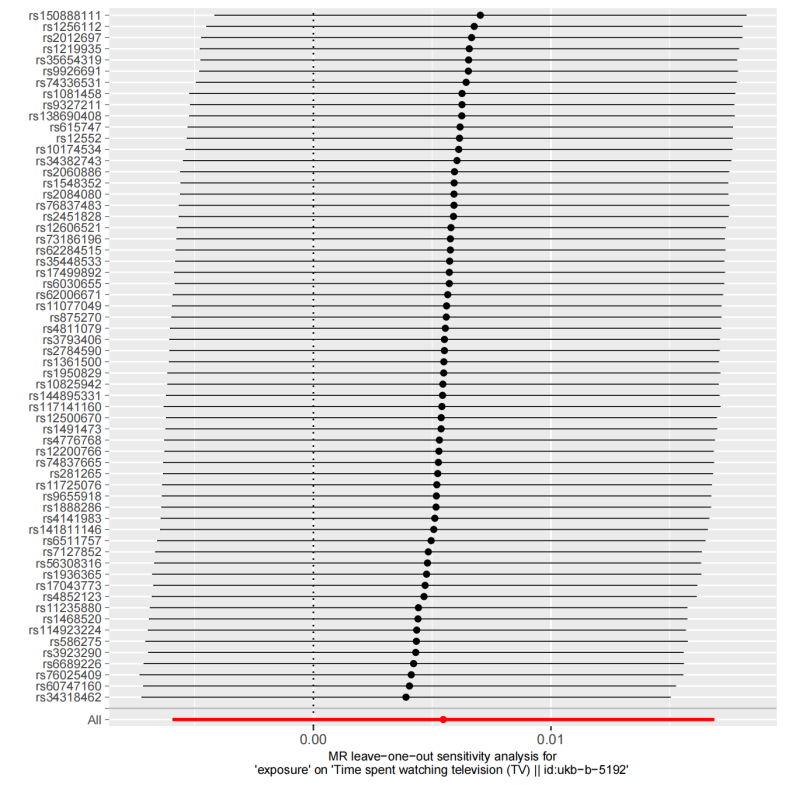 |

**Fig. S24.** Leave-one-out plot for the effects of major depression disorder on digital device use

(A) Length of mobile phone use, (B) Time spent using computer, (C) Plays computer games, and (D) Time spent watching television

| 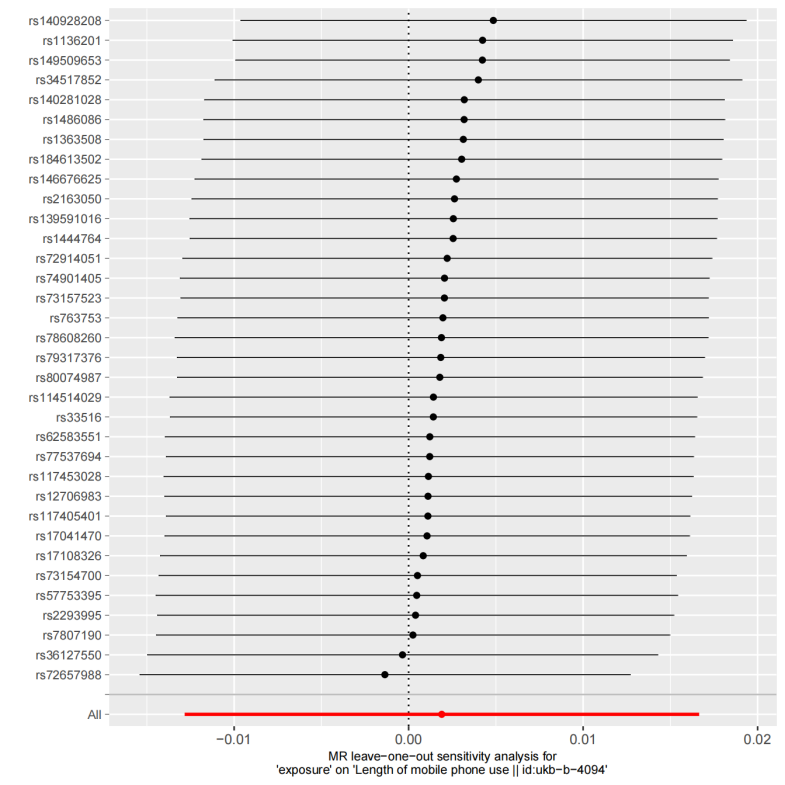  **D**  **B**  **A** | 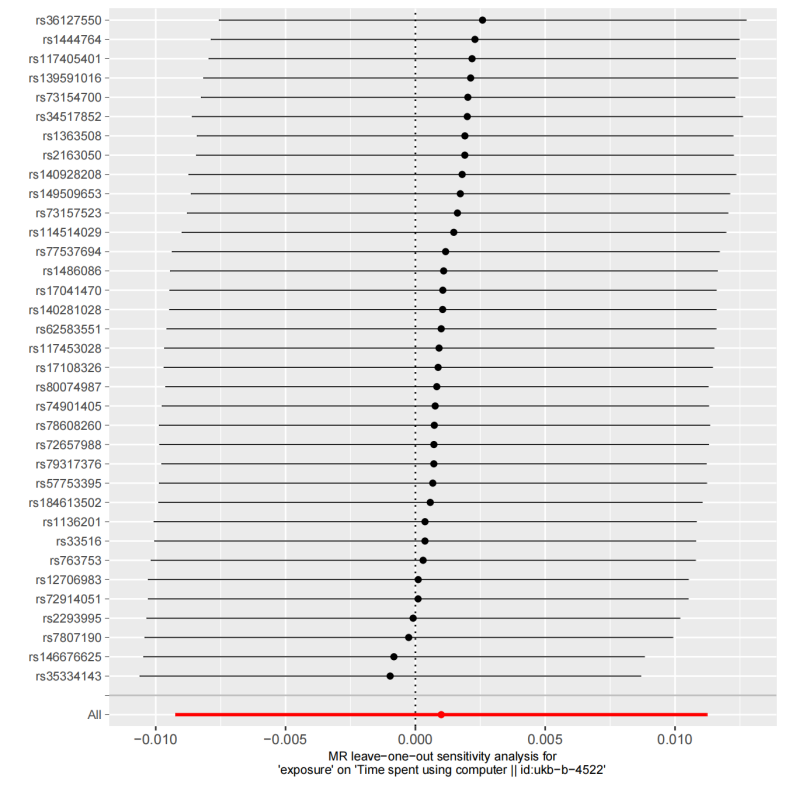 |
| --- | --- |
| 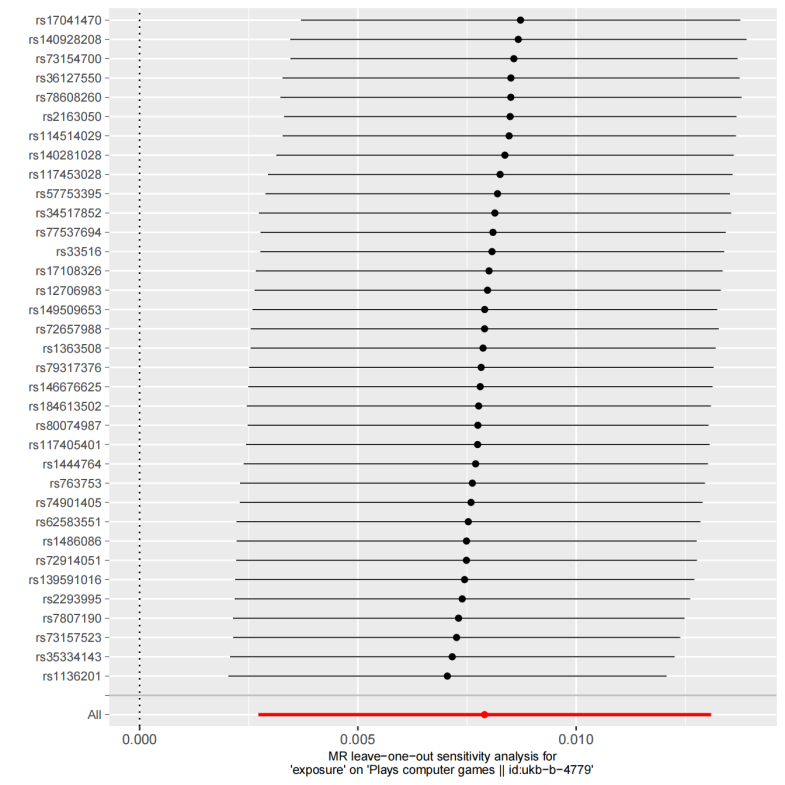  **C** | 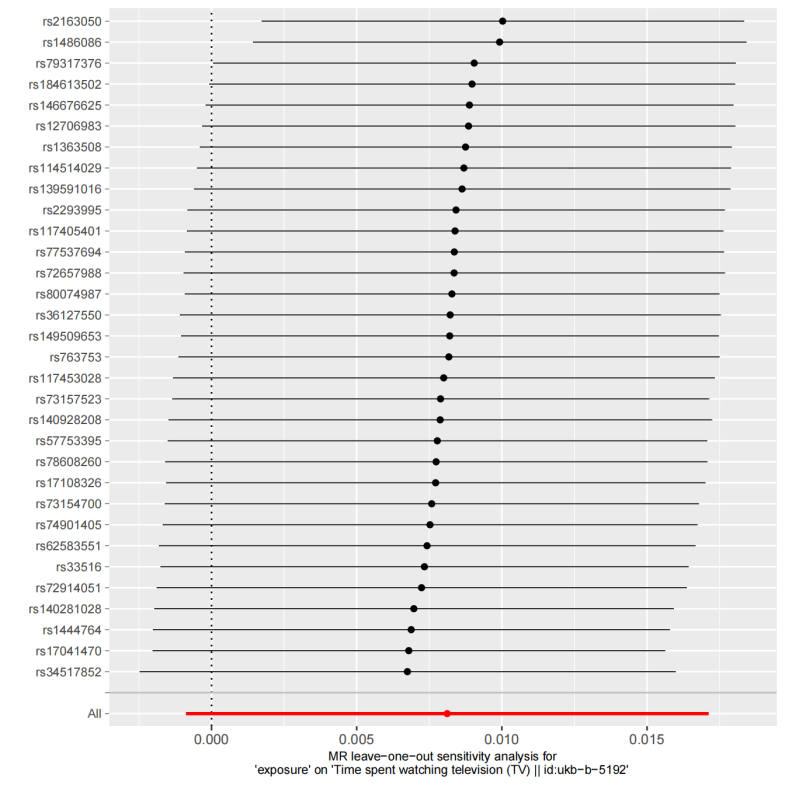 |

**Fig. S25.** Leave-one-out plot for the effects of post traumatic stress disorder on digital device use

1. Length of mobile phone use, (B) Time spent using computer, (C) Plays computer games, and (D) Time spent watching television
